# Deep learning directed synthesis of fluid ferroelectric materials

**DOI:** 10.1039/d6mh01249c

**Published:** 2026-09-11

**Authors:** Charles Parton-Barr, Stuart R. Berrow, Calum J. Gibb, Jordan Hobbs, Wanhe Jiang, Caitlin O’Brien, Will C. Ogle, Helen F. Gleeson, Richard J. Mandle

**Affiliations:** a School of Physics and Astronomy, University of Leeds Leeds LS29JT UK r.mandle@leeds.ac.uk; b School of Chemistry, University of Leeds Leeds LS29JT UK; c School of Applied Mathematics, University of Leeds Leeds LS29JT UK

## Abstract

We present a data-to-molecule deep-learning workflow for the targeted design of organic fluid ferroelectrics and validate it through the synthesis and experimental characterisation of machine-learning generated candidate materials. Fluid ferroelectrics, a class of liquid crystals that exhibit switchable long-range polar order, offer opportunities in ultrafast electro-optic technologies, responsive soft matter, and next-generation energy materials. Without well-validated design rules, development of new fluid ferroelectric materials remains challenging. We curate a comprehensive dataset of all known longitudinally polar liquid-crystal materials and train graph neural networks to predict ferroelectric nematic transition temperatures. A graph variational autoencoder generates *de novo* structures which are filtered by an ensemble of classifiers and regressors to identify candidates with predicted ferroelectric nematic behaviour and accessible transition temperatures. Integration with a computational retrosynthesis engine and a digitised chemical inventory narrows the design space to a synthesis-ready longlist. Eleven machine-learning-selected candidates were synthesised and characterised, with one molecule exhibiting a modulated antiferroelectric phase. Extrapolated ferroelectric nematic transitions were obtained, compared against model predictions, and used to augment the original dataset. Further iteration using graph neural networks and XGBoost models produced a second ranked set of candidates; chemical synthesis yields fluid ferroelectric materials through a practical, closed loop-workflow.

## Abstract 2

A closed-loop deep-learning workflow predicts, screens and synthesises thirteen new molecules, yielding two novel fluid ferroelectrics that exhibit the N_F_ phase.

## Graphical abstract

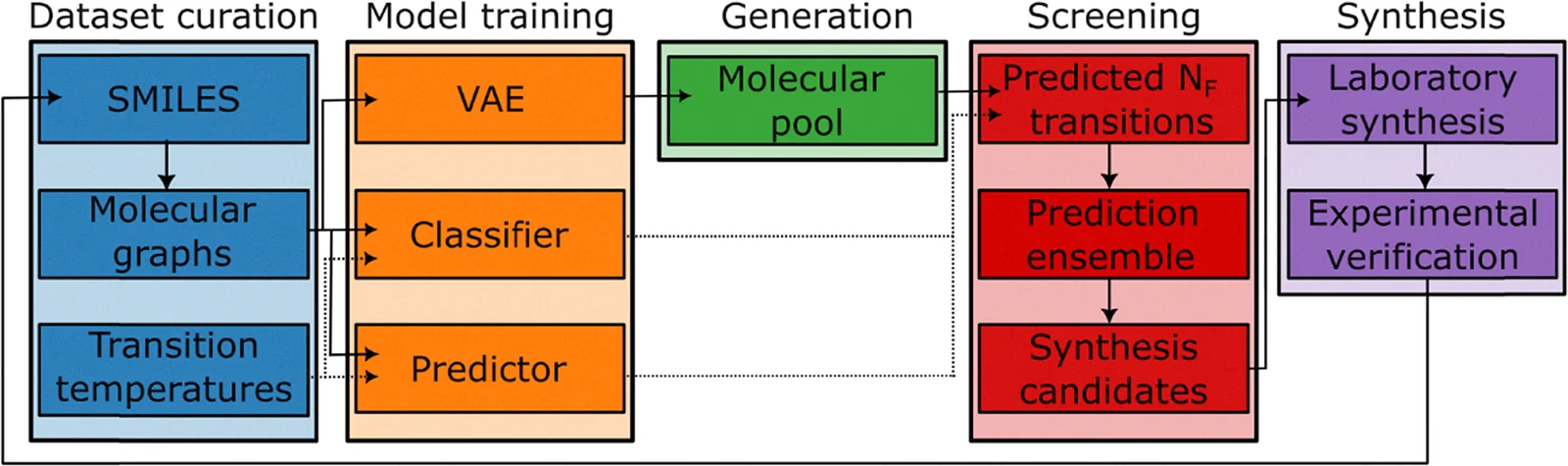

**New concepts**

This work establishes a closed-loop, data-to-molecule methodology for the discovery of liquid crystalline materials, using ferroelectric nematics as a demanding test case. The fluid ferroelectric nematic phase emerges from subtle, collective interactions that are poorly captured by simple molecular descriptors, and general structure–property rules remain underdeveloped. We integrate generative deep learning and graph-based property prediction with experimental synthesis and characterisation into a single discovery pipeline. Candidate structures are generated computationally, filtered by predictive models and synthetic accessibility, and then realised experimentally. Unlike conventional property prediction studies that terminate with candidate ranking, this work closes the loop: synthesised molecules are used as feedback data to retrain the models and generating refined candidate sets. This central concept of the work extends machine-learning materials discovery beyond descriptor-based prediction towards experimentally grounded, synthetically feasible molecular design. It also provides insight into model applicability, showing how predictive performance degrades as generated molecular structures diverge from the training domain.

## Introduction

The design of molecular materials requires domain specific expertise and understanding of the complex, often multidimensional relationship between chemical structure and macroscopic properties.^1^ The vastness of chemical space means that discovering new molecular materials through exploratory synthesis is inherently slow.^2^ Significant experimental effort is required to iteratively expand from known structures, a process prone to the encoding of human bias. Advances in computational power and algorithmic efficiency make deep learning an ever more practical approach for extracting structure–property relationships from large molecular datasets.^3^ Coupled to generative models capable of designing new molecular structures,^4^ this enables the data-driven exploration of chemical space and opens a new paradigm in the design of molecular materials. The end point of molecule design is physical realisation, and to best achieve this, the generative workflow output must be guided by a set of requirements.^5^ The molecules need to be realistically synthesisable, property prediction must be rigorously justified, expert human feedback integrated, and analysis of predictions should elucidate structural property relations.

Approaches for generating novel molecules can be broadly classified into rule-based systems and generative machine-learning models. Rule-based approaches construct new molecules by recombining predefined structural fragments or applying encoded chemical reactions.^6^ In contrast, generative models learn a latent representation of chemical space from training data, sampling this latent space to propose new chemical structures.^7^ Deep generative models have been developed using recurrent neural networks,^8^ variational autoencoders,^9^ and generative adversarial networks;^10^ each learning the implicit molecular syntax (*e.g.* from SMILES strings) and producing novel candidate structures. Recently, molecular graph neural networks have been combined with variational autoencoder architectures to generate new graph structures.^11^ While these methods have been successfully and widely applied in the context of drug discovery,^12^ protein structure,^13^ and inorganic/solid-state materials,^14^ they are yet to significantly impact the design of soft materials.^15^

**Fig. 1:**
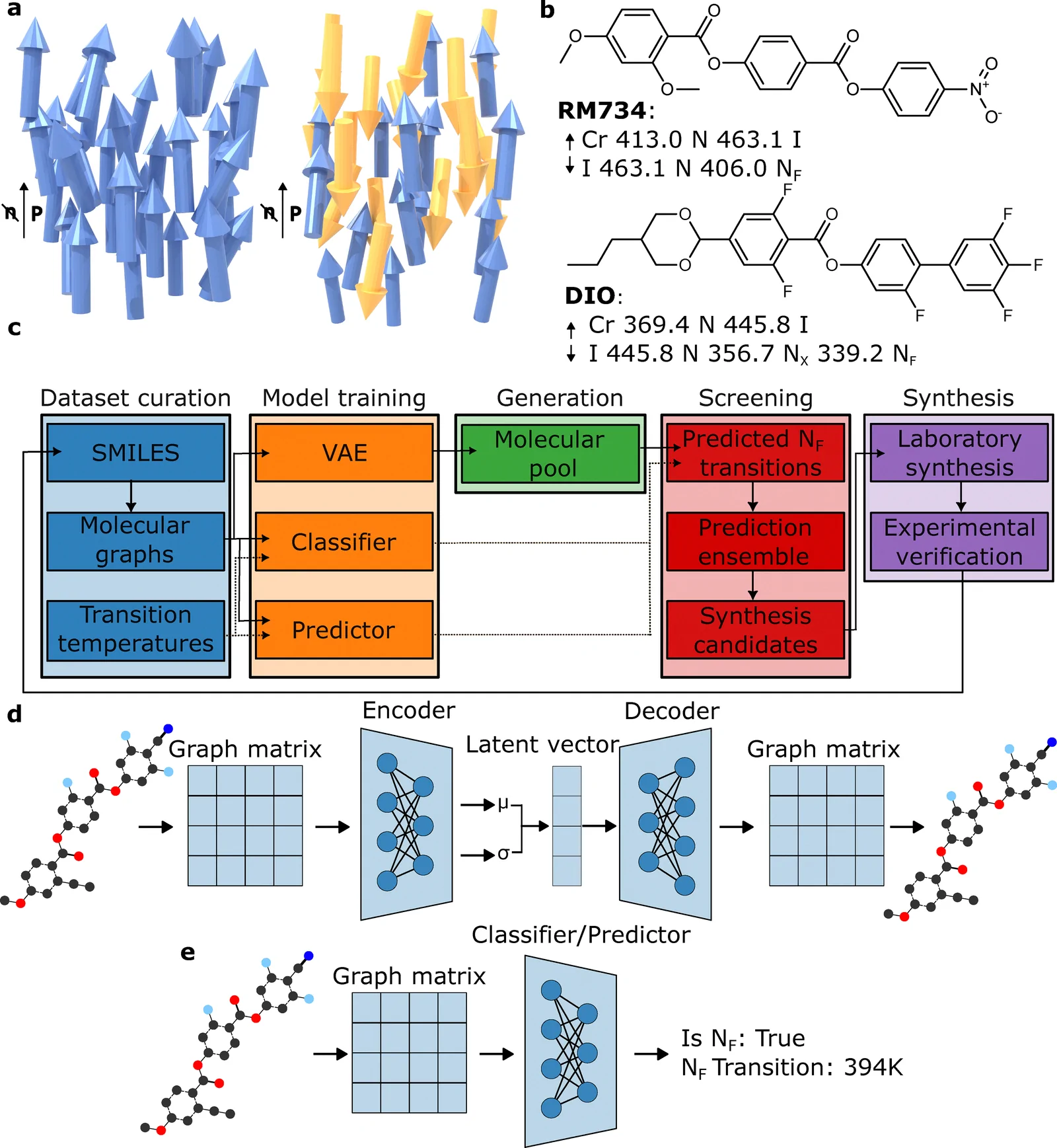
(a) A schematic representation of the ferroelectric nematic (N_F_) phase (right), in which molecular dipoles align collectively to produce macroscopic polar order and lack inversion symmetry. (b) Representative N_F_ materials RM734 and DIO, with molecular structures, transition temperatures in K. (c) Overview of the generative–predictive workflow for data-driven discovery of N_F_ materials, including dataset curation, model training, molecular generation, screening, and experimental validation. (d) Variational autoencoder architecture used for molecular generation. (e) Neural-network classifier and property prediction model architectures.

Liquid crystals provide a demanding testbed for molecular design: their bulk properties emerge from molecular structure through subtle and collective interactions that are not captured by simple molecular descriptors. Liquid crystals are soft phases of matter that combine degrees of orientational and positional order with fluidity, giving rise to states of matter (mesophases) with distinct symmetry and physical properties.^16–18^ Amongst these phases is the recently discovered ferroelectric nematic (N_F_) phase (Fig. 1a) which long range polar order arising from the parallel arrangement of molecular dipoles yields bulk spontaneous polarisation comparable to solid state ferroelectrics.^19^ This can be compared to a conventional nematic phase in which the molecules do not possess any dipole moment ordering and inversion symmetry is preserved.

The formation of liquid crystal phases and their specific mesophase type depends on a delicate balance of molecular shape and electronic structure, which impact on collective molecular organisation. Small structural modifications can give rise to significant differences in phase behaviour, making traditional structure–property heuristics unreliable. Only a small number of structural motifs are currently known to support the N_F_ phase, notably DIO,^20^ RM734^21,22^ (Fig. 1b) and derivatives thereof.^23–26^ This narrow chemical space stems from an absence of design rules for the structural-property relationship that causes the spontaneous polarity to emerge.^27–30^ Identifying new structural motifs capable of stabilising polar order therefore presents a significant challenge in the molecular design of such materials.

Machine learning studies in liquid crystal science have focused on property prediction or classification rather than molecular design. Examples include models for the classification of ferroelectric nematic materials,^31^ predicting clearing point (isotropisation) temperatures,^32,33^ phase transitions in bent-core type materials,^34^ dielectric properties,^35,36^ prediction of helical twisting power,^37^ phase sequence prediction^38^ or stability range,^39^ lyotropic phase behaviour,^40^ image-based frameworks for phase identification *via* optical texture analysis,^41,42^ hydrodynamic properties *via* director field dynamics,^43^ and optimisation of LCD backlight technology.^44^ Each of these studies illustrates the promise of data-driven methods in liquid crystal research but none address generative molecular design with explicit synthesis of new materials, which is the focus of the present work.

Here we introduce a generative-predictive framework for small-molecule design and apply it to the discovery of new ferroelectric nematic materials. This workflow (Fig. 1c) generates candidate structures and predicts N_F_ behaviour and transition temperatures, providing a data-driven approach to exploring the chemical space associated with this state of matter. We validate the framework by synthesis and characterisation of model-generated candidates; these form an *ex silico* testing set, which is used to further train and fine-tune machine learning models. We perform this complete workflow for two cycles; the first synthesised set exhibit only virtual N_F_ phases, whereas the second gives materials which exhibit the N_F_ phase in their neat state. The complete workflow establishes an experimental benchmark for data-driven materials discovery. A complete set of synthesised materials along with their transition temperatures can be found in Fig. S90.

## Methods

### Data setup

We curated a dataset comprising >500 small-molecule liquid crystal materials which includes all reported N_F_ materials, up to October 2025 and a further >200 materials from unpublished in-house measurements. Each entry consists of the molecular structure as SMILES and experimentally measured phase transition temperatures. To avoid biasing the model towards N_F_ behaviour the dataset was balanced with structurally similar non-N_F_ materials.

Smiles strings were converted to molecular graphs using RDKIT. In the graph representation, each molecule is represented by a node feature matrix, *V*. (atom descriptors) and an edge matrix, *E*. (bond connectivity). These graph representations were annotated with transition temperatures and used as inputs for deep-learning models (Fig. 1(d and e)). A more complete description of the featurisation methodology is given in section 5 of the SI. For the second iteration of the workflow, the scikit-fingerprint package is used to compute fingerprint representations of molecules.^45^

### Model training

We employ three deep learning architectures: (a) a graph variational autoencoder (VAE) for generation of *de novo* structures (b) a binary classifier to predict the presence or absence of N_F_ behaviour; (c) a scalar predictor (regression model) for predicting N_F_ transition temperatures (Fig. 1e).

The VAE is a modified MGVAE architecture^11^ and learns a probabilistic latent representation of molecular structure. The encoder maps molecular graphsnto a continuous latent space, and the decoder reconstructs graphs from latent vectors (*i.e.* the inverse process), enabling the generation of novel molecules.

For classification and regression graph neural networks transform molecular graphs in scalar outputs corresponding to phase assignment or N_F_ transition temperature. Our model library (Fig. S1–S9) includes classifiers and regressors built on the following message-passing layers, GATConv,^46^ GATv2Conv,^47^ GINConv,^48^ GCNConv,^49^ GatedGraphConv,^50^ TransformerConv.^51^

We additionally train XGBoost^52^ classifiers and regressors on fingerprint representations. In the second iteration of the workflow, their predictions are combined with those of the GNNs, producing a cross-model consensus filtering that includes distinct model architectures.

Given the modest size of our dataset (∼700 molecules) repeated *k*-fold cross-validation was used for model training and performance evaluation. Hyperparameter settings for the training of models can be found in the SI.

### Generation

After training the VAE model was used to generate novel molecular structures by sampling its latent space and decoding the resulting vectors into molecular graphs. Because the latent space derives from experimentally characterised liquid crystal molecules, the generated structures occupy a similar chemical manifold (“dataset-like”) while also allowing exploration of training set's chemical space.

The decoded molecular graphs were converted into canonical SMILES strings; after filtering any duplicates this yields a pool of novel molecules occupying the learned N_F_ liquid crystal chemical manifold, ∼30 000 novel structures overall.

### Screening

We construct an ensemble of classifiers by selecting the three most accurate models from across four different graph neural network architectures. Each classifier simply returns a Boolean value indicating the presence or absence of the N_F_ phase. An analogous ensemble of predictors was selected in the same manner, choosing for the lowest root mean square error (RMSE) of N_F_ transition temperature predictions.

Molecules for which the ensemble of classifiers demonstrated high consensus were subsequently evaluated using the ensemble of predictors, with the mean predicted N_F_ transition temperature as a ranking criterion. This yields the generated pool of materials.

From the screened pool we selected candidate molecules representing both high predicted likelihood of N_F_ behaviour and low-probability negative controls. Computational retrosynthesis tools^53^ allied to our digitised chemical inventory and vendor catalogues allowed us to identify longlist of synthetically accessible targets. This machine generated longlist was then reviewed and prioritised by a team of human synthetic chemists.

### Synthesis

All compounds were synthesised using standard organic chemistry techniques in conventional laboratory glassware, either open to air or under an atmosphere of dry nitrogen. All chemical reactions were ultimately subject to purification by flash chromatography over silica gel using a Combiflash NextGen 300+ system (Teledyne Isco) with an appropriate solvent gradient. Final materials were filtered through 0.2-micron PTFE filters to remove particulates. Yields refer to isolated, spectroscopically homogenous material. Chemical characterisation was made by NMR spectroscopy (^1^H,^13^C and ^19^F) and high-resolution mass spectrometry (HRMS), all materials were confirmed to have purity of >99% (peak area) by reverse phase HPLC. Full synthetic details are given in sections 8–10 of the SI (Fig. S26–S89). Synthesised materials were studied by polarised optical microscopy (POM) and differential scanning calorimetry (DSC). Transition temperatures for the virtual polar phases were extrapolated from mixture studies with DIO as the host (∼90 wt%) and the material in question as the guest (∼10 wt%). In total, we synthesised a total of 13 novel compounds as part of this work with eleven produced in cycle one and two in cycle two.

## Results

To evaluate how the generative model explores chemical space relative to the experimental dataset, we compared distributions of selected structural descriptors (Fig. S10). The generated molecules exhibit broader variability in size and ring count, while retaining similar overall carbon distributions. Nitrogen-containing structures are less common in the generated set, whereas fluorination increases modestly. Oxygen counts shift toward simpler structures, suggesting the model samples lower-polarity motifs while retaining ester functionality. Substructure analysis (SMARTS-based) indicates reduced frequency of specific motifs that are over-represented in the training set (*e.g.*, multiple difluorobenzene rings), alongside emergence of new functionalities such as ketones. Collectively, these patterns indicate that the generative model learns the dominant structural trends in liquid-crystal materials while exploring adjacent chemical space rather than memorising training examples. Our classifier and predictor ensembles are comprised of four different types of graph convolutional layers and we identify the most accurate models for each architecture *via* hyperparameter searching (Tables S1 and S2).

**Fig. 2:**
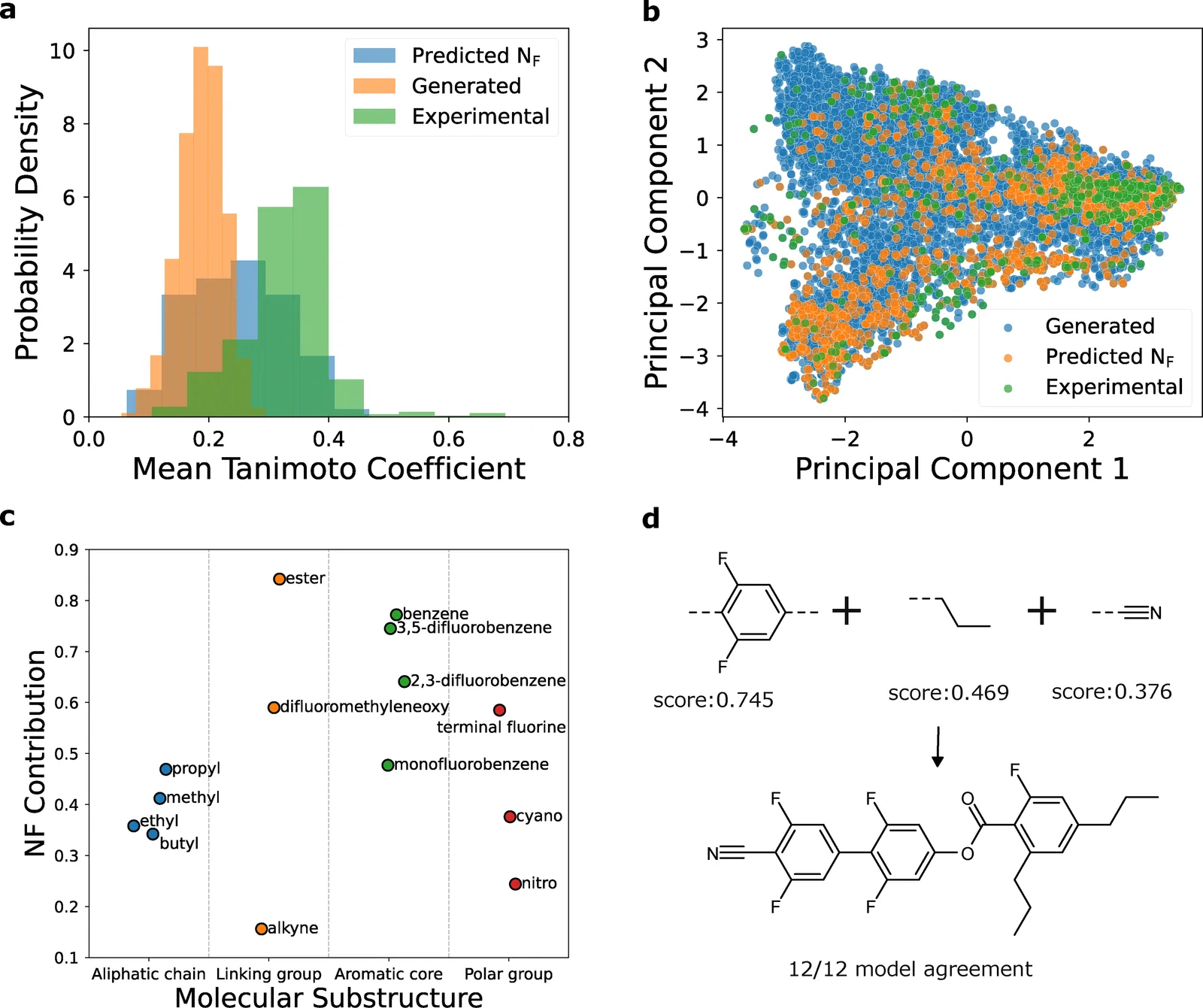
Analysing the model decisions in classifying a material as N_F_. (a) The Tanimoto coefficients comparing the molecule similarity within the three datasets. (b) Visualisation of the structural similarity of the total three datasets. (c) Subgraph masking explanation scoring of the contribution to the N_F_ phase for molecular substructures present in the generated molecules. (d) Contribution-guided design coupled with scaffold analysis of the dataset.

To assess whether the model explores new chemical space, we compared molecular similarity distributions across the experimental dataset, the generated pool, and the predicted N_F_ subset using Tanimoto coefficients of Morgan fingerprints (Fig. 2a). As expected, generated structures are more diverse than the experimental set, while predicted N_F_ candidates cluster closer to experimental N_F_ molecules, indicating that the model prioritises plausible N_F_ like scaffolds while expanding chemical diversity beyond known motifs.

Principal component analysis (PCA) of molecular fingerprints further shows that generated and predicted N_F_ molecules populate regions adjacent to (but extending beyond) the experimentally explored N_F_ space (Fig. 2b), confirming that the generative model proposes novel yet chemically realistic structures.

To gain insight into model decisions, we applied the substructure-masking explanation method (SME)^54^ to quantify the influence of functional groups on N_F_ predictions (Fig. 2c). Removing specific fragments and recomputing predictions yields a per-fragment contribution score, identifying structural features that promote or suppress N_F_ behaviour. Combining high-contribution motifs provides a rational, interpretable route to designing candidate molecules (Fig. 2d), complementing chemical intuition.

### Synthesis and experimental validation of *de novo* compounds

**Fig. 3:**
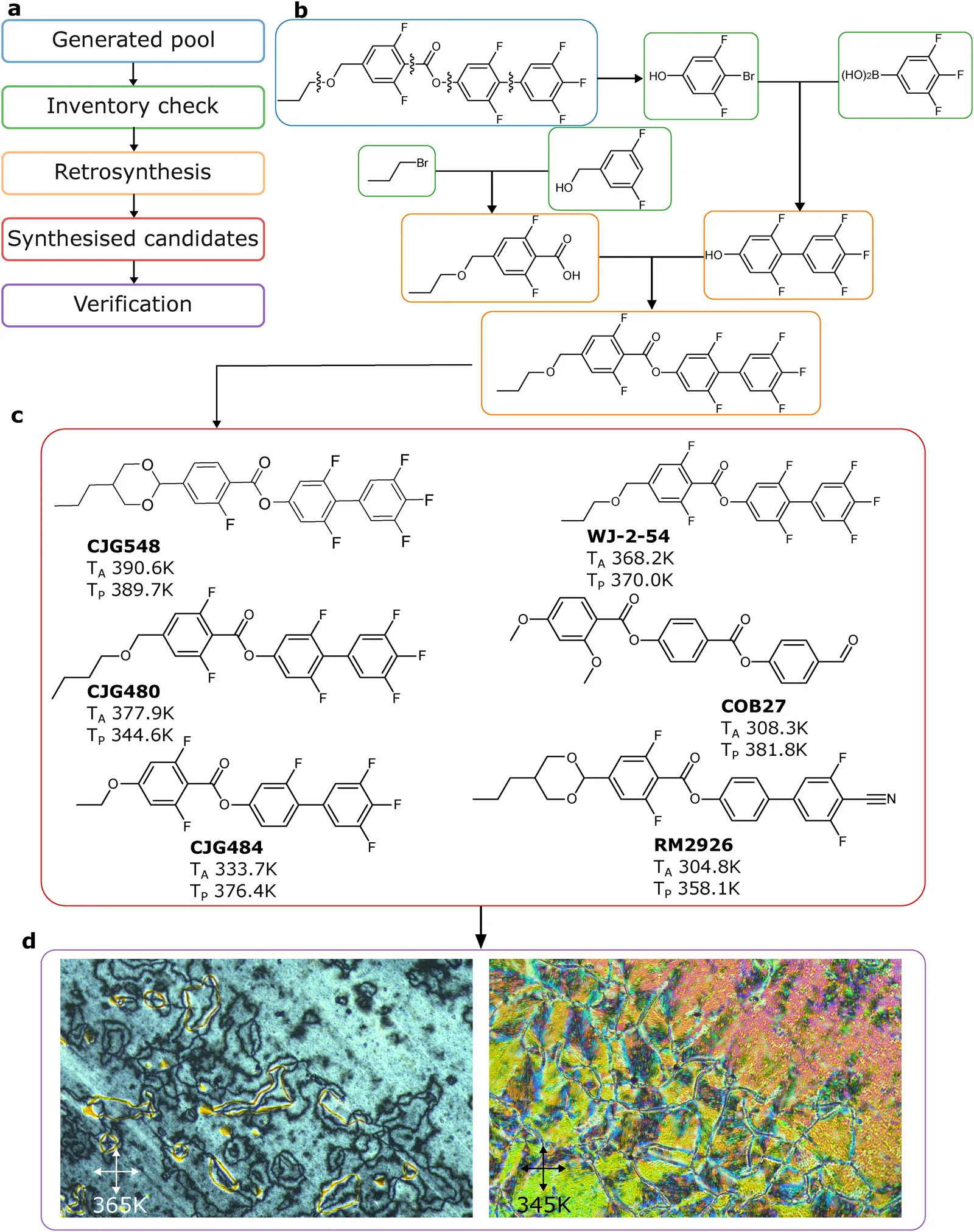
From machine learning predictions to experimental verification. (a) The stages of the workflow that takes the molecules from generated to physical materials. (b) Retrosynthesis coupled with a laboratory stock inventory assists in the identification of readily synthesisable molecules within the generated pool. (c) Synthesised N_F_ structures and their extrapolated (*T*_A_) and predicted (*T*_P_) N_F_ transition temperatures. (d) Once synthesised, POM analysis of 90 wt% DIO: 10 wt% guest mixtures are used to extrapolate N_F_ transition temperatures.

To select synthetically tractable targets from the generated pool we employed AiZynthFinder^53^ to identify feasible retrosynthetic routes. The required precursors were cross referenced with our in-house digitised chemical inventory (Fig. 3b), yielding a list of materials which could be rapidly accessible. Synthesis candidates were subsequently reviewed by synthetic chemists to confirm feasibility. From this process, we synthesised 11 previously unreported materials in this campaign. Later, we synthesise two additional materials based on a second generated pool. Materials were obtained in >99% purity (peak area) as judged by C18-HPLC analysis (where feasible); full synthetic details and chemical characterisation data are given in the SI to this article. We utilise extrapolated *T*_NF_ values to remove the explicit dependency on melting point and supercooling range for the 11 materials in the first generation. The structures of a selection of these synthesised N_F_ candidates are given in Fig. 3c with their extrapolated actual transition temperatures (*T*_A_; K) and predicted transition temperatures (*T*_P_; K). The resulting dataset provides a direct test of the generative-predictive pipeline. The molecules can be assessed and used to augment the original experimental dataset, providing further structural data from which the models can learn from in further iterations of this process, thus continuing to guide molecular discovery. Molecules with a high chemical similarity to existing N_F_ materials (CJG548, CJG480, CJG484, WJ-2-54) have extrapolated N_F_ transition temperatures within ∼40 K of the predicted values. Of note, CJG548 exhibits the modulated antiferroelectric phase (N_X_) phase, confirmed through the current response technique (Fig. S91). There is a growth in error as the structures increasingly differ from experimental dataset culminating in a maximum discrepancy between predicted and actual *T*_NF_ of 73 K (COB27). Full details of extrapolated N_F_ transitions for the complete set of synthesised molecules can be found in the SI (Fig. S11–S19). The novel structural and transitional data is then added to the N_F_ dataset to supplement further predictions; we synthesise two of these materials, and find them to exhibit the N_F_ phase in their neat state.

### Implementation of a closed loop workflow

In the previous section, the eleven synthesis candidates functioned as an experimental probe of the GNN ensemble predictions, these revealing a performance gap between model predictions and experimentally realised transition temperatures. We elected to augment the N_F_ dataset with an additional 200 materials taken from the literature^55–61^ published after our initial cutoff date (October 2025), as well as the 11 *de novo* materials synthesised during the first cycle and additional in-house measurements. At this point, the dataset comprises ∼1000 entries. We then perform a second iteration of the model training pipeline, implementing several methodological additions. Firstly, we further partitioned the data to include a held-out test set against which the *in silico* performance of the GNN models was evaluated, allowing us to perform Optuna^62^ hyperparameter searches tested against metrics of the validation folds. Secondly, we trained XGBoost classifiers and regressors to predict the N_F_ transition temperature. Third, ensembles were trained for the six GNN architectures (GAT, GATv2, GCN, GIN, GatedGraph and TransformerConv) and XGBoost models with the latter being assembled from selection of molecular fingerprint representations (Table S3). The fingerprints used in the XGBoost ensembles are given Table S3 of the SI. The ensemble sizes for the GNNs were increased from the first iteration (30 models for each architecture *vs.* a 12-model selection) to reduce the variance of the predictions. Scaffold splitting of the dataset was introduced to reduce the structural data leakage between the training, validation and testing splits and from this scaffold-trained classifiers and regressors were produced for each architecture.

**Fig. 4:**
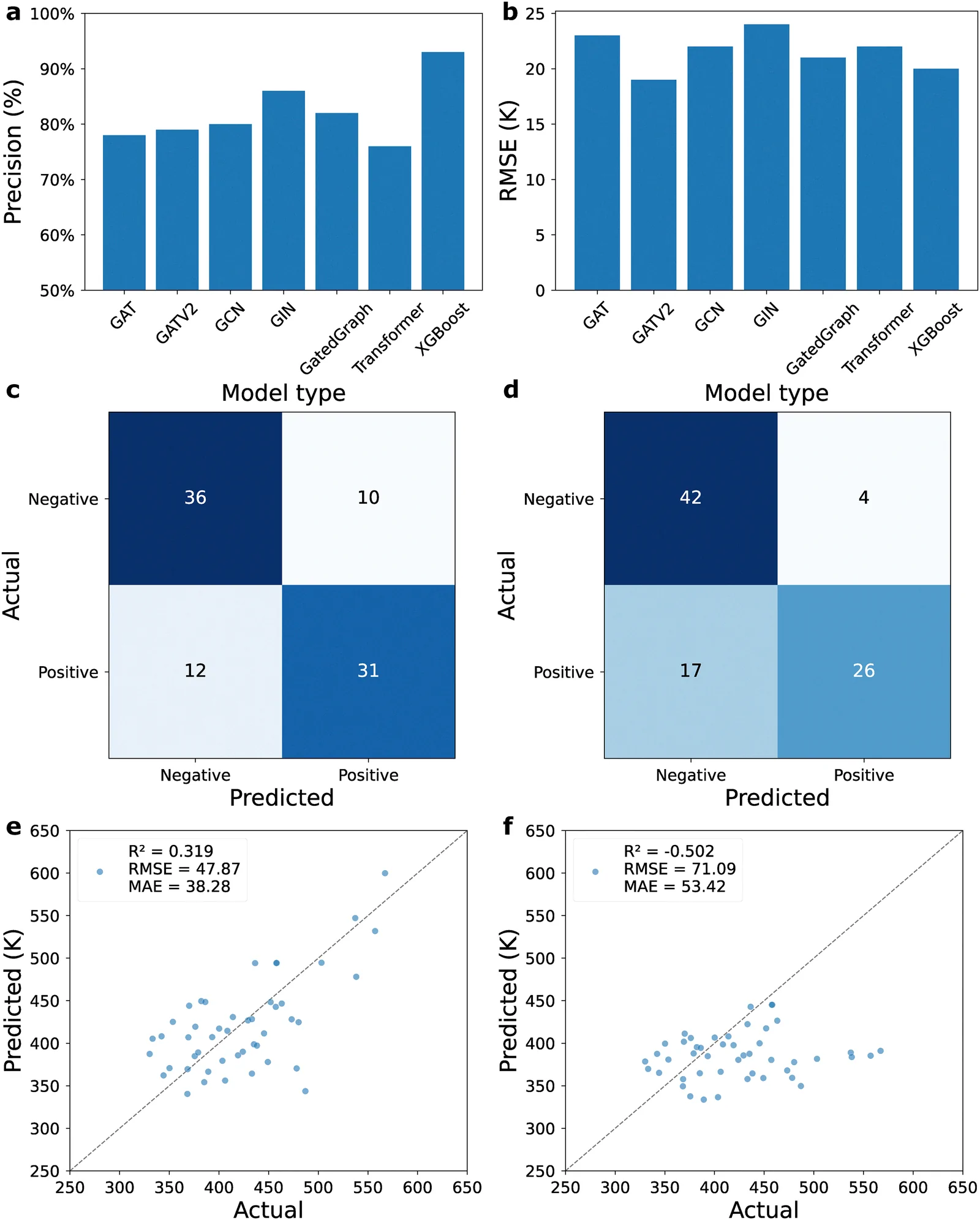
Model performance analysis for the second iteration through the NFML workflow. Classifier precision (a) and regressor RMSE (b) metrics for random split GNNs and XGBoost models. Confusion matrices for (c) GATv2 scaffold and (d) XGBoost scaffold classifiers. Predicted *vs.* actual plots for (e) GATv2 scaffold and (f) XGBoost scaffold regressors.

Fig. 4a and b present the precision and RMSE for the GNN models trained on a random split of the dataset. Classifiers were evaluated through their precision and recall metrics. All random split models possess a precision of above 75% and a recall above 90%. The random split XGBoost models have a greater precision for classification tasks whereas the GATv2 model performs the best for RMSE. We consider the precision to be the more significant metric for assessing the viability of synthesis candidates as a false positive is a waste of resources. The false negative reduces the predicted candidate pool, but given this is many tens of thousands of molecules in size we deem this acceptable.

Fig. 4c and d compare the confusion matrices of the scaffold split trained (c) GATv2 and (d) XGBoost classifiers. The XGBoost classifier has a greater precision, 86% *vs.* 75% respectively, but predicts fewer actual positives than the GATv2 classifier. Demonstrated by the XGBoost classifier possessing a recall of 60% *vs.* the GATv2 classifier's 72%. Fig. 4e and f compare the predicted *vs.* actual figures for the (e) GATv2 and (f) XGBoost regressors. For a *T*_NF_ of over 500 K the XGBoost regressor fails to give accurate predictions, whereas the GATv2 regressor can more accurately predict on the same dataset. Scaffold models except for GATv2 and XGBoost are discarded for possessing a recall performance of <60%. All scaffold regressors except for GATv2 were discarded from predictions ensembles due to negative *R*^2^ values.

The scaffold-*vs.*-random comparison along with the experimental results shows that random split models overstate their generalisability to unseen data. GATv2 and XGBoost are the only classifier models that show an acceptable level of performance degradation when trained on scaffold splits of the data, suggesting that these architectures are better able to capture the structural features that govern N_F_ phase formation. These results show that the split strategy and model architecture should be considered when assessing a molecule's suitability for synthesis. Confusion matrices and predicted *vs.* actual plots for all model architectures and splitting types can be found in section 6 of the SI (Fig. S20–S23).

Two sets of prediction candidates filtered by molecules with accessible retrosynthetic routes are presented in section 7 of the SI (Fig. S24 and S25). Set A is constructed from the predictions of all random split model architectures and rely on a cross-architecture consensus for the validation of their predictions. Set B is constructed from models trained on a scaffold split of the data with XGBoost and GATv2 classifiers and the GATv2 regressor being selected for use based on the analysis of their performance metrics performed above. The sets were constructed by evaluating the total votes each molecule receives and filtered into a final set of NF candidates filtered by strong consensus (60% ensemble confidence) to yield a tractable final pool of molecules. These molecules were then passed to regressors to predict their range of N_F_ transition temperatures. The predicted candidates that survive this filtering are therefore consistent across model choice and molecular representation. All molecules within these retrosynthesis-filtered sets possess a Tanimoto similarity to the training dataset of above 0.6, indicating that there's a moderate structural similarity to the existing N_F_ dataset and there is confidence in the structures falling within the applicability domain of the models. Candidate sets without retrosynthesis filtering can be found in the GitHub repository linked at the end of this manuscript.

**Fig. 5:**
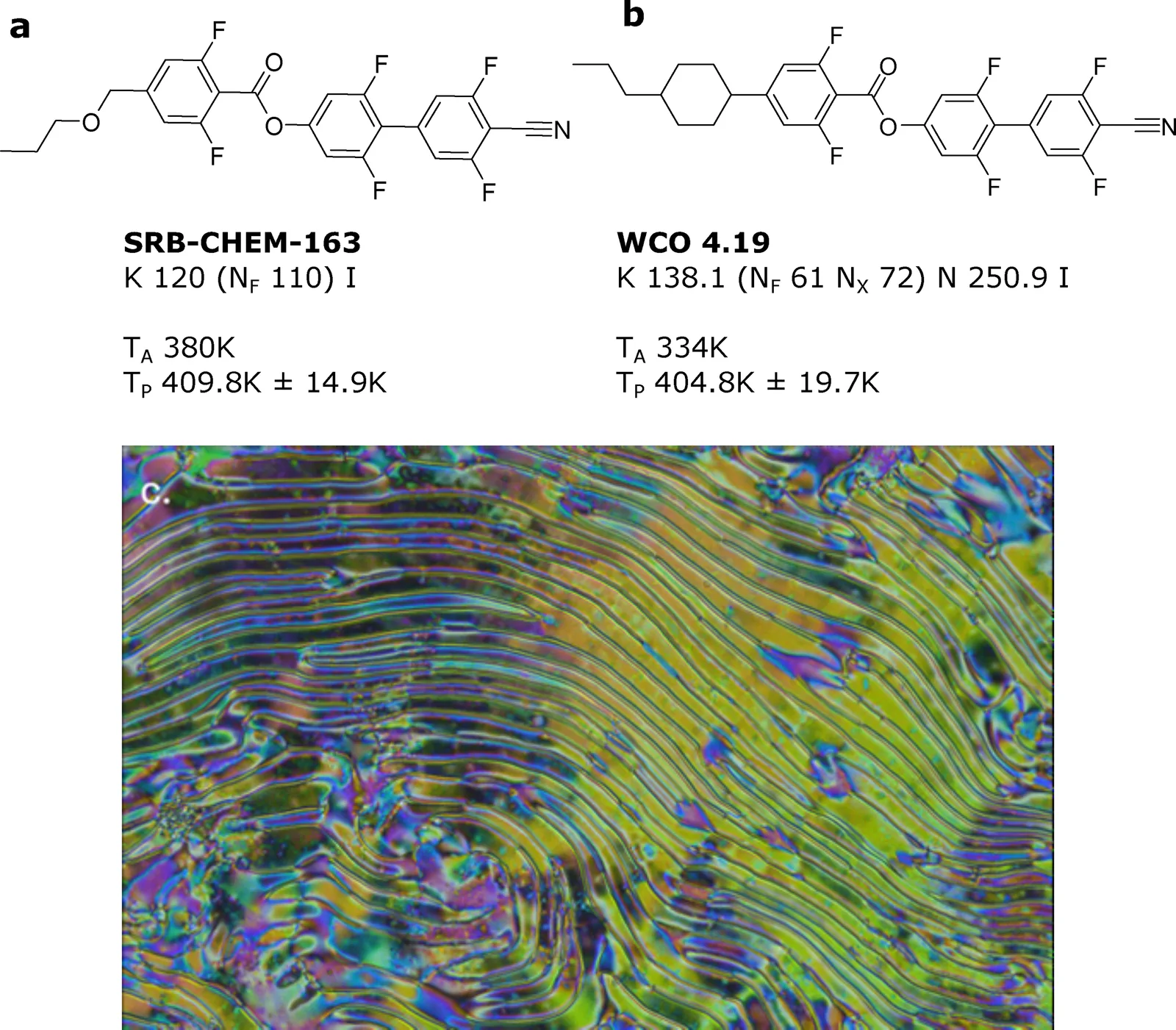
Structures of synthesised materials SRB-CHEM-163 (a) and WCO 4.19 (b) from the second pool of generated candidates. Molecular structures are annotated with their experimental phase sequence for the neat material (in °C), the actual N_F_ transition temperatures (*T*_A_, in K), and the predicted N_F_ transition temperature and the standard deviation of the ensemble predictions (*T*_P_, in K). We show a POM image of the N_F_ phase of SRB-CHEM-163 at 96 °C, taken between untreated glass coverslips. Candidates were ultimately selected by consensus filtering between the scaffold-split trained GATv2 and XGBoost classifiers, while the N_F_ transition temperatures were predicted by an ensemble of GATv2 regressors.

Fig. 5a and b presents the synthesised candidate molecules that were selected from set B, along with their predicted N_F_ transition temperatures (and the standard deviation across ensemble predictions) and their experimentally measured transition temperatures. Both materials exhibit the N_F_ phase, with the cyclohexane material also exhibiting N_X_ and apolar nematic phases. These molecules were selected based on both synthetic accessibility and the large model agreement (30/30 of the GATv2 scaffold classifiers predict an N_F_ phase). The experimental transition temperature for the N_F_ phase is somewhat lower than the prediction for both materials. Representative optical textures observed during POM are given in Fig. 5.

## Conclusion

A generative-predictive framework for data-driven design and experimental validation of small molecules is presented. By integrating molecular graph learning, ensemble prediction, retrosynthetic filtering, and laboratory synthesis, this provides a practical end-to-end approach for accelerating materials discovery.

Applied to the discovery of ferroelectric nematic liquid crystals, we use this workflow to propose synthetically accessible candidates which are then synthesised and subjected to experimental verification. Several possess extrapolated N_F_ transitions within reasonable boundaries, demonstrating that machine-learned chemical intuition can successfully guide molecular design in a domain where subtle structural changes govern emergent properties.

The synthesised molecules were added to the training data for a second iteration through the workflow, producing two sets of synthesis candidates. Set A was selected by consensus across random-split trained classifiers and regressors. Set B was selected by consensus between scaffold-split trained GATv2 and XGBoost classifiers with N_F_ predicted by an ensemble of GATv2 regressors. Two molecules were selected from set B based on synthetic accessibility and high confidence rating of the machine learning models, and were found to exhibit the N_F_ phase in their neat form, without recourse to extrapolation of *T*(N_F_).

This work illustrates a generalisable strategy for closed-loop molecular innovation: learn from experimental structure–property data, generate candidates within a realistic chemical manifold, triage using model consensus and retrosynthetic constraints, and validate in the laboratory *via* chemical synthesis and experimental physical characterisation. We expect this approach to extend to other classes of functional soft materials and to accelerate the rational exploration of chemical space. Moreover, the integration of comparable workflows with high throughput synthesis and characterisation offers the prospect of autonomous, machine led, exploration of chemical space.

## Author contributions

The dataset was curated by CPB, JH, CJG, and RJM. CPB and RJM developed the machine learning code used in this work. Chemical synthesis was performed by SRB, COB, CJG, WJ, WCO, RJM. Characterisation of liquid crystalline behaviour was undertaken by (SRB, CPB, CJG, JH, WJ, WCO, COB, RJM). RJM and HFG secured funding. The first draft was written by CPB and RJM, with contributions from all authors.

## Conflicts of interest

There are no conflicts of interest to declare.

## Supplementary Material

MH-013-D6MH01249C-s001

## Data Availability

The data supporting this article have been included as part of the supplementary information (SI). Supplementary information is available. See DOI: https://doi.org/10.1039/d6mh01249c. The code used in this article is available at https://github.com/123cpb/nfml. The data associated with this article is openly available from the University of Leeds Data Repository at https://doi.org/10.5518/1798.
