# Supplementary material for: Deep learning directed synthesis of fluid ferroelectric materials
Source: Mater Horiz. 2026 Sep 11;13(21):10672–82. doi: 10.1039/d6mh01249c (PMC13569083; doi:10.1039/d6mh01249c)
Supplement: Supplementary file 1 [file MH-013-D6MH01249C-s001.pdf]

Supplementary Information (SI) for Materials Horizons.
This journal is © The Royal Society of Chemistry 2026

# Deep learning directed synthesis of fluid ferroelectric materials

Charles Parton-Barr[1], Stuart R. Berrow [1], Calum J. Gibb [2], Jordan Hobbs [1], Wanhe Jiang [1], Caitlin O'Brien [1,2,3], Will C. Ogle [1,2,3], Helen F. Gleeson[1], Richard J. Mandle[1,2,*]

[1]School of Physics and Astronomy, University of Leeds, Leeds, UK, LS29JT
[2]School of Chemistry, University of Leeds, Leeds, UK, LS29JT
[3]School of Applied Mathematics, University of Leeds, Leeds, UK, LS29JT

* r.mandle@leeds.ac.uk

**Contents:**

1. **Model architectures**

2. **Model hyperparameters**
   **2.1. Iteration 1**
   **2.2. Iteration 2**

3. **Substructure distribution histograms**

4. **Mixture transition textures**

5. **Data featurisation**

6. **Iteration 2 performance metrics**

7. **Candidate pools**

8. **Chemical methods**
   **8.1. Chemical Synthesis**
   **8.2. Chemical Characterisation Methods**
   **8.3. Mesophase Characterisation**
   **8.4. General Reaction Protocols**
   **8.5. Synthetic Schemes**

9. **Chemical Characterisation**
   **9.1. Scheme One**
   **9.2. Scheme Two**
   **9.3. Scheme Three**
   **9.4. Scheme Four**
   **9.5. Scheme Five**

**9.6. Scheme Six**
**9.7. Scheme Seven**
**9.8. Scheme Eight**
**9.9. Scheme Nine**

10. **Analytical Data**
**10.1. Scheme One**
**10.2. Scheme Two**
**10.3. Scheme Three**
**10.4. Scheme Four**
**10.5. Scheme Five**
**10.6. Scheme Six**
**10.7. Scheme Seven**
**10.8. Scheme Eight**
**10.9. Scheme Nine**
**10.10. Scheme Ten**

11. **Candidate structures**

12. **Current response**

# 1. Model Architectures

## 1.1. Variational Autoencoder (VAE)

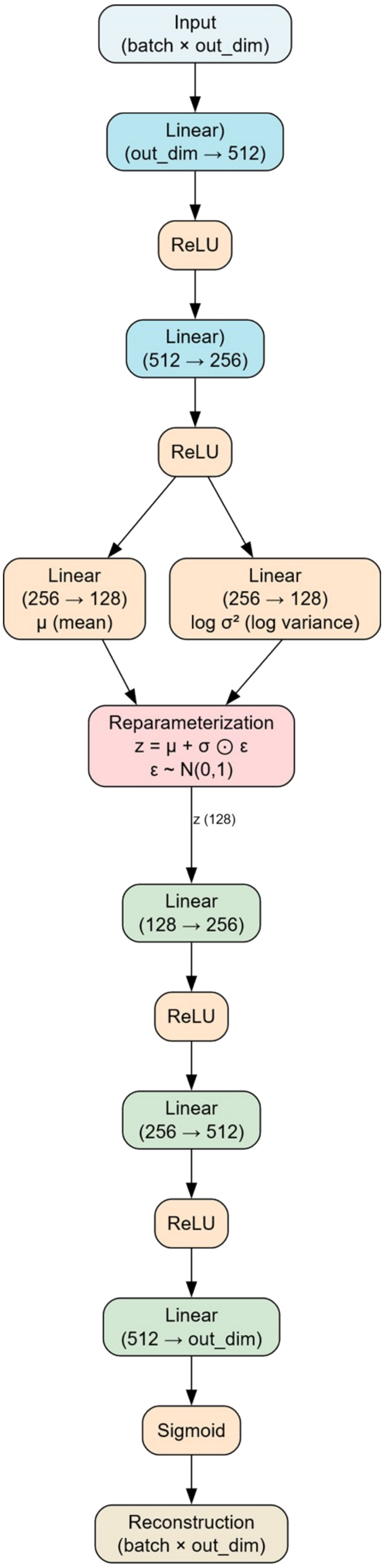


**Figure SI-1:** Schematic representation of the VAE model with example hyperparameters

### 1.2. GATClassifier

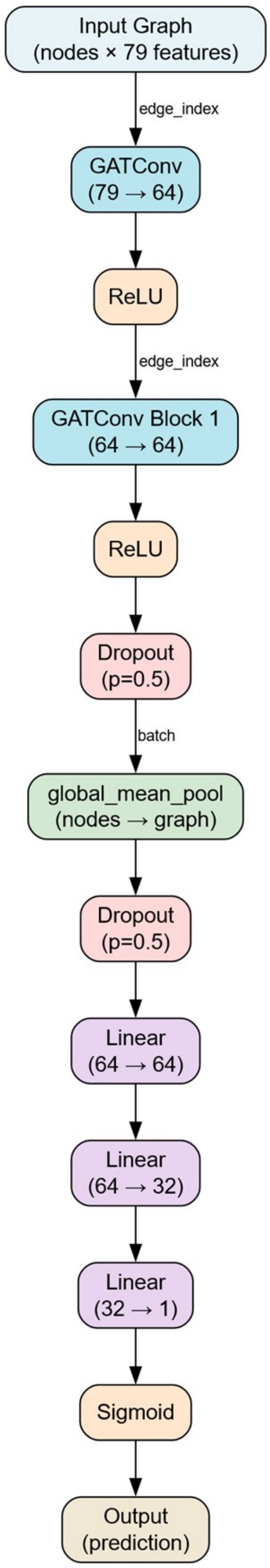


**Figure SI-2:** Schematic representation of the GATClassifier model architecture with example hyperparameters.

## 1.3. GATV2 Classifier

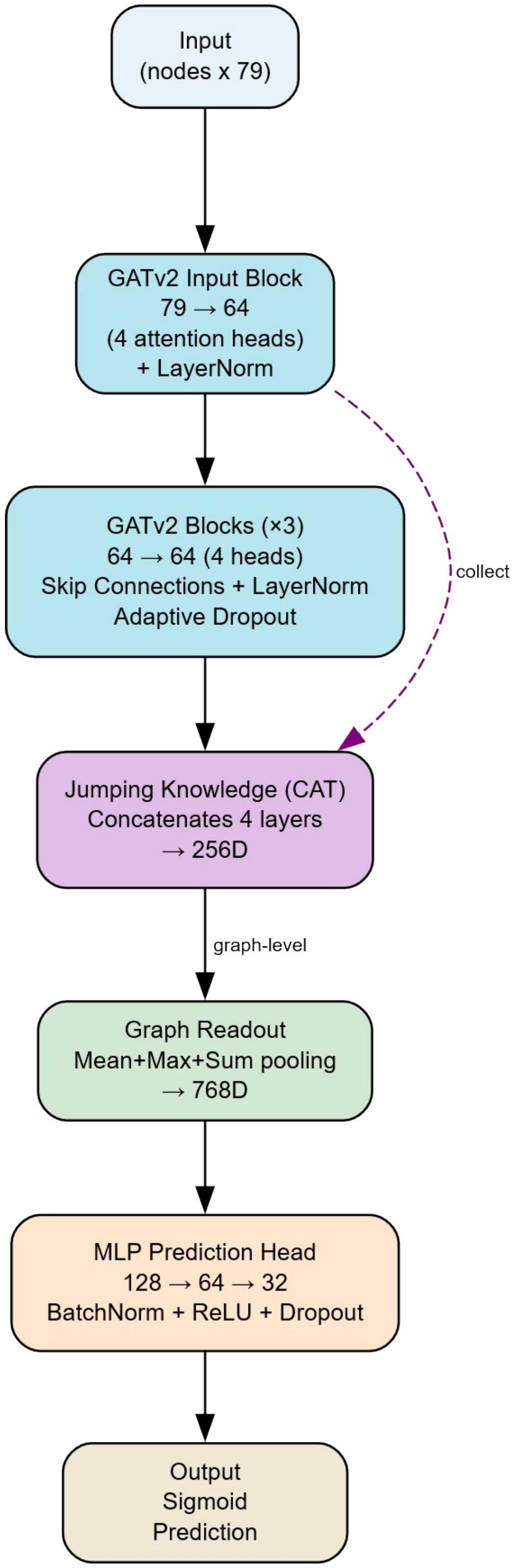


**Figure SI-3:** Schematic representation of the GATv2 Classifier model architecture with example hyperparameters. The architecture has been simplified for layout purposes.

## 1.4. GINClassifier

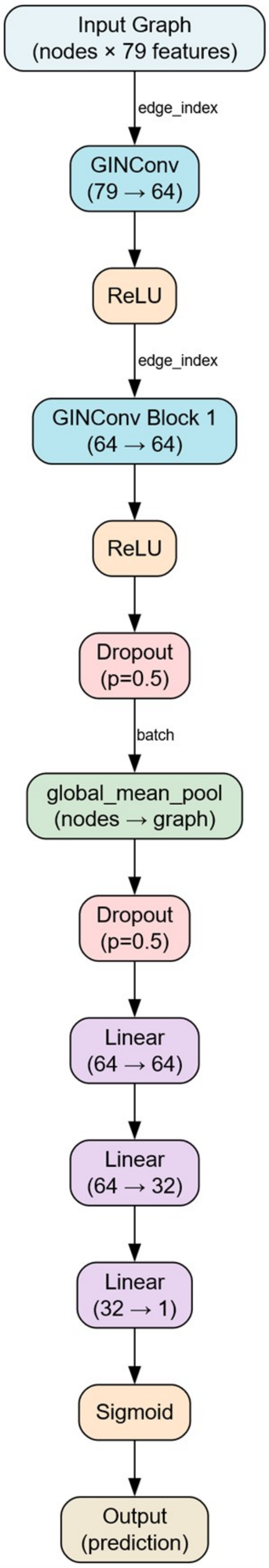


**Figure SI-4**: Schematic representation of the GINClassifier model architecture with example hyperparameters

### 1.5. GCNClassifier

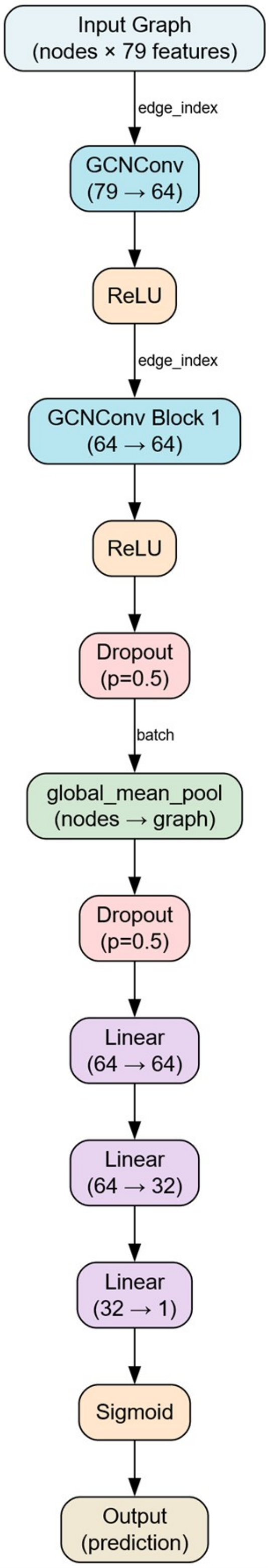


**Figure SI-5:** Schematic representation of the GCNClassifier model architecture with example hyperparameters

## 1.6. GATPredictor

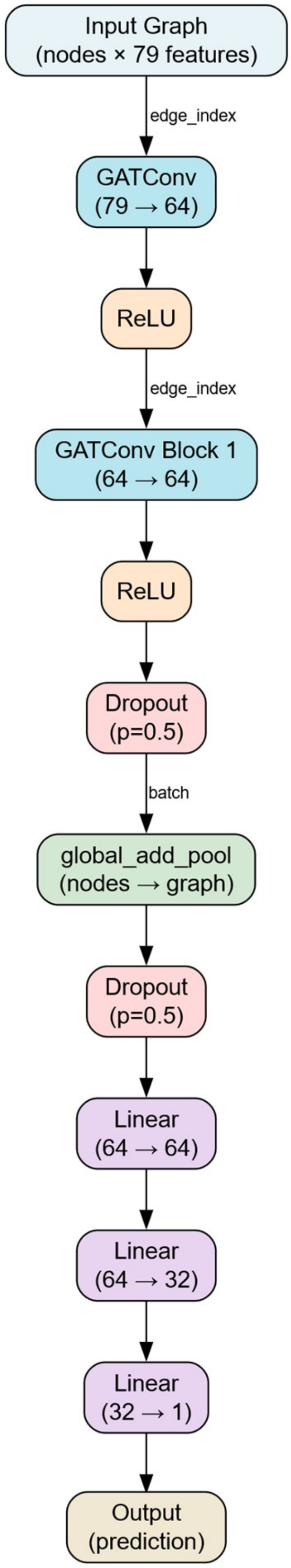


**Figure SI-6** Schematic representation of the GATPredictor model architecture with example hyperparameters

## 1.7. GATV2 Predictor

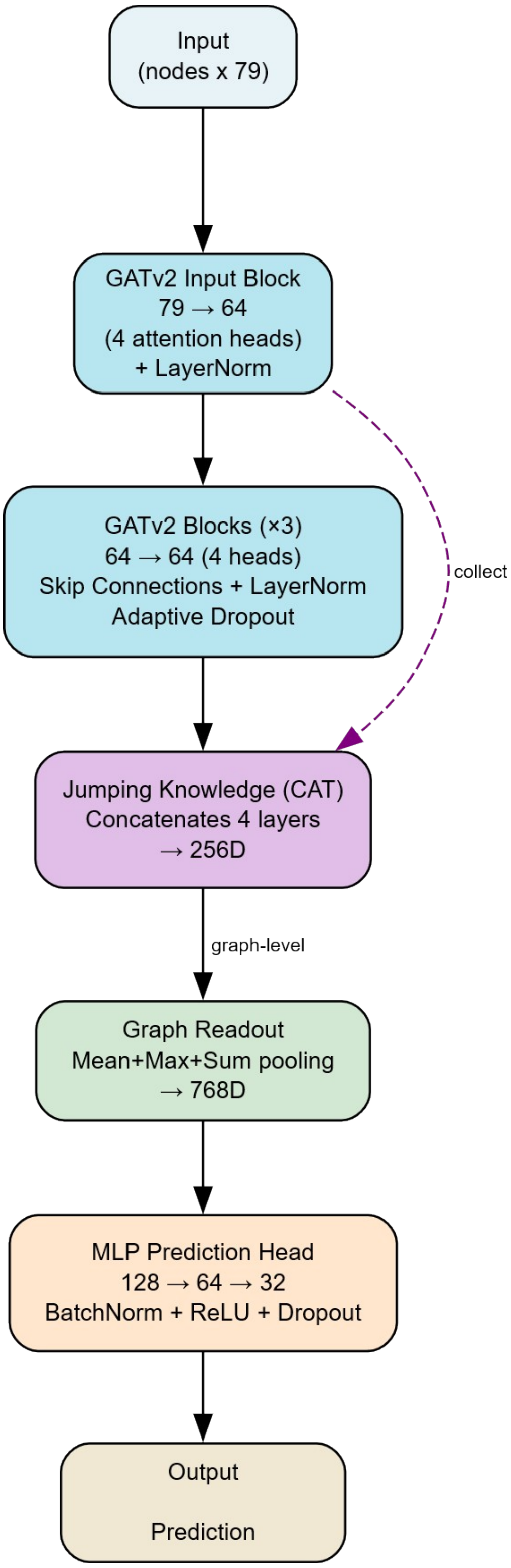


**Figure SI-7:** Schematic representation of the GATV2 predictor model architecture with example hyperparameters. The architecture has been simplified for layout purpose

### 1.8. GINPredictor

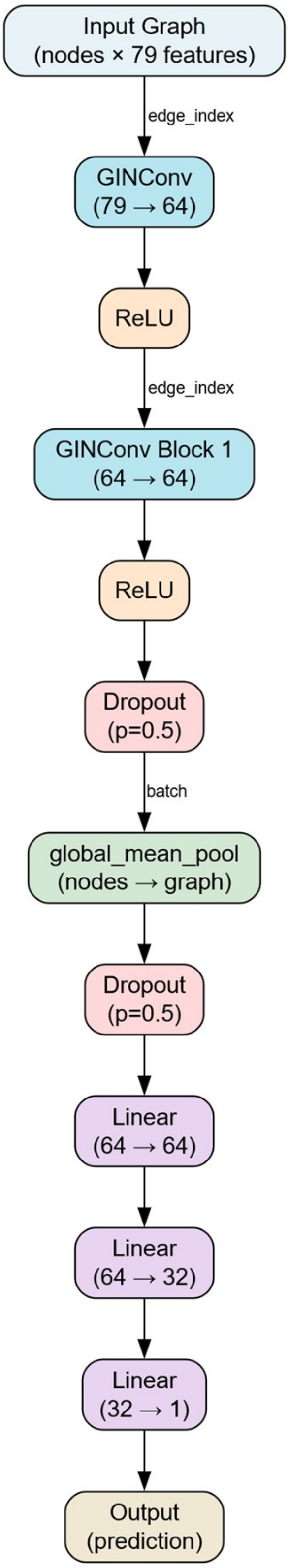


**Figure SI-8:** Schematic representation of the GINPredictor model architecture with example hyperparameters

### 1.9. GCNPredictor

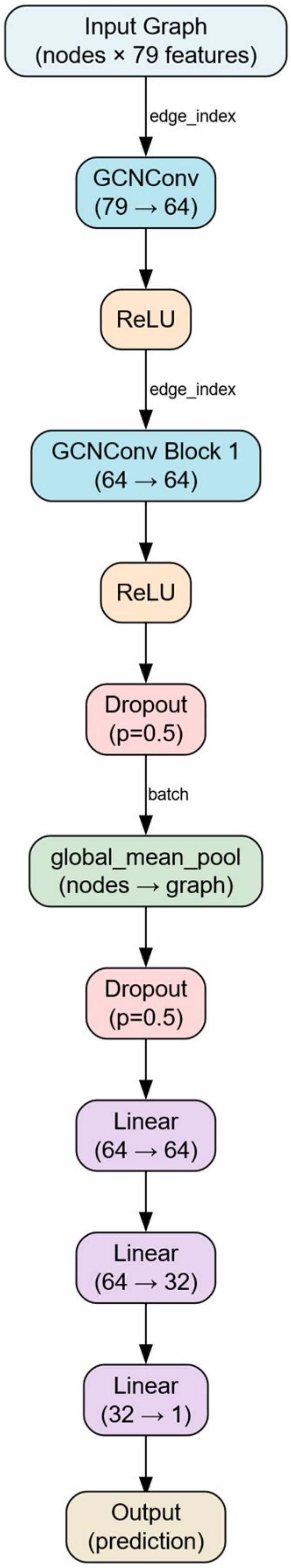


**Figure SI-9:** Schematic representation of the GCNpredictor model architecture with example hyperparameters

## 2. Model hyperparameters

### 2.1. Iteration 1

| Model | Hyperparameters |
|---|---|
| 1 | Model type: Classifier<br>Convolutional blocks: 3<br>Dropout: 0.5<br>Convolutional layer hidden dimension: 256<br>Learning rate: 0.001<br>Batch size: 32<br>Epochs: 50<br>k-fold splits: 5<br>number of repeats: 3 |
| 2 | Model type: Classifier<br>Convolutional blocks: 10<br>Dropout: 0.25<br>Convolutional layer hidden dimension: 256<br>Learning rate: 0.001<br>Batch size: 16<br>Epochs: 50<br>k-fold splits: 5<br>number of repeats: 3 |
| 3 | Model type: Classifier<br>Convolutional blocks: 10<br>Dropout: 0.25<br>Convolutional layer hidden dimension: 256<br>Learning rate: 0.001<br>Batch size: 32<br>Epochs: 50<br>k-fold splits: 5<br>number of repeats: 3 |
| 4 | Model type: EnhancedClassifier<br>Convolutional blocks: 3<br>Dropout: 0.25<br>Convolutional layer hidden dimension: 256<br>Learning rate: 0.001<br>Batch size: 32<br>Epochs: 50<br>k-fold splits: 5<br>number of repeats: 3 |
| 5 | Model type: EnhancedClassifier<br>Convolutional blocks: 3<br>Dropout: 0.25 |

| | |
|---|---|
| | Convolutional layer hidden dimension: 512<br>Learning rate: 0.001<br>Batch size: 16<br>Epochs: 50<br>k-fold splits: 5<br>number of repeats: 3 |
| 6 | Model type: EnhancedClassifier<br>Convolutional blocks: 3<br>Dropout: 0.25<br>Convolutional layer hidden dimension: 512<br>Learning rate: 0.001<br>Batch size: 32<br>Epochs: 50<br>k-fold splits: 5<br>number of repeats: 3 |
| 7 | Model type: GCNClassifier<br>Convolutional blocks: 3<br>Dropout: 0.25<br>Convolutional layer hidden dimension: 512<br>Learning rate: 0.001<br>Batch size: 32<br>Epochs: 50<br>k-fold splits: 5<br>number of repeats: 3 |
| 8 | Model type: GCNClassifier<br>Convolutional blocks: 3<br>Dropout: 0.25<br>Convolutional layer hidden dimension: 256<br>Learning rate: 0.001<br>Batch size: 16<br>Epochs: 50<br>k-fold splits: 5<br>number of repeats: 3 |
| 9 | Model type: GCNClassifier<br>Convolutional blocks: 3<br>Dropout:0.25<br>Convolutional layer hidden dimension: 512<br>Learning rate:0.001<br>Batch size: 16<br>Epochs: 50<br>k-fold splits: 5<br>number of repeats: 3 |
| 10 | Model type: GINClassifier |

| | |
|---|---|
| | Convolutional blocks: 3<br>Dropout: 0.5<br>Convolutional layer hidden dimension: 256<br>Learning rate: 0.001<br>Batch size: 32<br>Epochs: 25<br>k-fold splits: 5<br>number of repeats: 3 |
| 11 | Model type: GINClassifier<br>Convolutional blocks: 3<br>Dropout: 0.5<br>Convolutional layer hidden dimension: 512<br>Learning rate: 0.001<br>Batch size: 32<br>Epochs: 50<br>k-fold splits: 5<br>number of repeats: 3 |
| 12 | Model type: GINClassifier<br>Convolutional blocks: 3<br>Dropout: 0.5<br>Convolutional layer hidden dimension: 512<br>Learning rate: 0.001<br>Batch size: 32<br>Epochs:50<br>k-fold splits:3<br>number of repeats: 5 |

**Table SI-1:** Hyperparameters of the twelve classifier-type models that make up our iteration 1 voting ensemble

| Model | Hyperparameters |
|---|---|
| 1 | Model type: Predictor<br>Convolutional blocks: 3<br>Dropout: 0.25<br>Convolutional layer hidden dimension: 256<br>Learning rate: 0.001<br>Batch size:16<br>Epochs: 50<br>k-fold splits: 5<br>number of repeats: 3 |
| 2 | Model type: Predictor<br>Convolutional blocks: 3<br>Dropout: 0.25<br>Convolutional layer hidden dimension: 512<br>Learning rate: 0.001<br>Batch size: 32<br>Epochs: 50<br>k-fold splits: 5<br>number of repeats: 3 |
| 3 | Model type: Predictor<br>Convolutional blocks: 3<br>Dropout: 0.5<br>Convolutional layer hidden dimension: 512<br>Learning rate: 0.001<br>Batch size: 32<br>Epochs: 50<br>k-fold splits: 5<br>number of repeats: 3 |
| 4 | Model type: EnhancedPredictor<br>Convolutional blocks: 3<br>Dropout: 0.25<br>Convolutional layer hidden dimension: 256<br>Learning rate: 0.001<br>Batch size: 16<br>Epochs: 50<br>k-fold splits: 5<br>number of repeats: 3 |
| 5 | Model type: EnhancedPredictor<br>Convolutional blocks: 3<br>Dropout: 0.25<br>Convolutional layer hidden dimension: 256<br>Learning rate: 0.001<br>Batch size: 32 |

| | |
|---|---|
| | Epochs: 50<br>k-fold splits: 5<br>number of repeats: 3 |
| 6 | Model type: EnhancedPredictor<br>Convolutional blocks: 3<br>Dropout: 0.25<br>Convolutional layer hidden dimension: 512<br>Learning rate: 0.001<br>Batch size: 32<br>Epochs: 50<br>k-fold splits: 5<br>number of repeats: 3 |
| 7 | Model type: GCNPredictor<br>Convolutional blocks: 10<br>Dropout: 0.25<br>Convolutional layer hidden dimension: 256<br>Learning rate: 0.001<br>Batch size: 16<br>Epochs: 50<br>K-fold splits: 5<br>Number of repeats: 3 |
| 8 | Model type: GCNPredictor<br>Convolutional blocks: 3<br>Dropout: 0.25<br>Convolutional layer hidden dimension: 512<br>Learning rate: 0.001<br>Batch size: 16<br>Epochs: 50<br>k-fold splits: 5<br>Number of repeats: 3 |
| 9 | Model type: GCNPredictor<br>Convolutional blocks: 3<br>Dropout: 0.25<br>Convolutional layer hidden dimension: 512<br>Learning rate: 0.001<br>Batch size: 16<br>Epochs: 50<br>k-fold splits: 5<br>number of repeats: 3 |
| 10 | Model type: GINPredictor<br>Convolutional blocks: 3<br>Dropout: 0.5<br>Convolutional layer hidden dimension: 512 |

| | |
|---|---|
| | Learning rate: 0.001<br>Batch size: 32<br>Epochs: 50<br>k-fold splits: 5<br>number of repeats: 3 |
| 11 | Model type: GINPredictor<br>Convolutional blocks: 3<br>Dropout: 0.25<br>Convolutional layer hidden dimension: 256<br>Learning rate: 0.001<br>Batch size: 16<br>Epochs: 50<br>k-fold splits: 5<br>number of repeats: 3 |
| 12 | Model type: GINPredictor<br>Convolutional blocks: 3<br>Dropout: 0.25<br>Convolutional layer hidden dimension: 256<br>Learning rate:0.001<br>Batch size:32<br>Epochs: 50<br>k-fold splits: 5<br>number of repeats: 3 |

**Table SI-2**: Hyperparameters of the twelve predictor-type models that make up our mean ensemble

### 2.2. Iteration 2

| | |
|---|---|
| GATv2_classifier_random | Model type: GATv2Classifier<br>Convolutional blocks: 4<br>Split type: random<br>Dropout: 0.06453371686984193<br>Convolutional layer hidden dimension: 256<br>Learning rate: 0.0002963783947778693<br>Batch size:128<br>Epochs: 134<br>k-fold splits: 5<br>number of repeats: 3 |
| GATv2_classifier_scaffold | Model type: GATv2Classifier<br>Convolutional blocks: 7<br>Split type: scaffold<br>Dropout: ": 0.08572640535698162<br>Convolutional layer hidden dimension: 512<br>Learning rate: 0.00014598173992939992 |

| | |
|---|---|
| | Batch size:32<br>Epochs: 77<br>k-fold splits: 5<br>number of repeats: 1 |
| GATv2_regression_random | Model type: GATv2Predictor<br>Convolutional blocks: 3<br>Split type: random<br>Dropout: 0.050648010737235766<br>Convolutional layer hidden dimension: 256<br>Learning rate: 0.001326755059733017<br>Batch size:128<br>Epochs: 164<br>k-fold splits: 5<br>number of repeats: 3 |
| GATv2_regression_scaffold | Model type: GATv2Classifier<br>Convolutional blocks: 3<br>Split type: scaffold<br>Dropout: 0.07262193184426281<br>Convolutional layer hidden dimension: 2048<br>Learning rate: 0.0005627555836364237<br>Batch size:128<br>Epochs: 1345<br>k-fold splits: 5<br>number of repeats: 1 |
| GAT_classification_random | Model type: GATClassifier<br>Convolutional blocks: 4<br>Split type: random<br>Dropout: 0.36560519439636197<br>Convolutional layer hidden dimension: 512<br>Learning rate: 0.0019720784929833294<br>Batch size:128<br>Epochs: 178<br>k-fold splits: 5<br>number of repeats: 3 |
| GAT_regression_random | Model type: GATClassifier<br>Convolutional blocks: 2<br>Split type: random<br>Dropout: 0.12851254578153898<br>Convolutional layer hidden dimension: 128<br>Learning rate: 0.0027288673346892948<br>Batch size:64<br>Epochs: 134<br>k-fold splits: 5 |

| | |
|---|---|
| | number of repeats: 3 |
| GG_classification_random | Model type: GatedGraphClassifier<br>Convolutional blocks: 2<br>Split type: random<br>Dropout: 0.3719301907573434<br>Convolutional layer hidden dimension: 256<br>Learning rate: 0.00011685814088177345<br>Batch size:16<br>Epochs: 297<br>k-fold splits: 5<br>number of repeats: 3 |
| GG_predictor_random | Model type: GatedGraphPredictor<br>Convolutional blocks: 2<br>Split type: random<br>Dropout: 0.29667032373742863<br>Convolutional layer hidden dimension: 256<br>Learning rate: 0.00046414581776362673<br>Batch size:32<br>Epochs: 211<br>k-fold splits: 5<br>number of repeats: 3 |
| GCN_classification_random | Model type: GCNClassifier<br>Convolutional blocks: 3<br>Split type: random<br>Dropout: 0.36856148280182427<br>Convolutional layer hidden dimension: 256<br>Learning rate: 0.0020097134871543154<br>Batch size:16<br>Epochs: 222<br>k-fold splits: 5<br>number of repeats: 3 |
| GCN_predictor_random | Model type: GCNPredictor<br>Convolutional blocks: 2<br>Split type: random<br>Dropout: 0.21407488272172256<br>Convolutional layer hidden dimension: 2048<br>Learning rate: 0.0015880323955553737<br>Batch size:32<br>Epochs: 278<br>k-fold splits: 5<br>number of repeats: 3 |
| GIN_classification_random | Model type: GINClassifier<br>Convolutional blocks: 2 |

| | |
|---|---|
| | Split type: random<br>Dropout: 0.33620310886845617<br>Convolutional layer hidden dimension: 2048<br>Learning rate: 0.0002963783947778693<br>Batch size:16<br>Epochs: 193<br>k-fold splits: 5<br>number of repeats: 3 |
| GIN_predictor_random | Model type: GINPredictor<br>Convolutional blocks: 2<br>Split type: random<br>Dropout: 0.18981454545142928<br>Convolutional layer hidden dimension: 2048<br>Learning rate: 0.00013120667432220057<br>Batch size:32<br>Epochs: 254<br>k-fold splits: 5<br>number of repeats: 3 |
| Transformer_classifier_random | Model type: TransformerClassifier<br>Convolutional blocks: 2<br>Split type: random<br>Dropout: 0.07820827682277663<br>Convolutional layer hidden dimension: 1024<br>Learning rate: 0.0003861495044875059<br>Batch size:16<br>Epochs: 162<br>k-fold splits: 5<br>number of repeats: 3 |
| Transformer_regressor_rando<br>m | Model type: TransformerPredictor<br>Convolutional blocks: 2<br>Split type: random<br>Dropout: 0.10148251352352801<br>Convolutional layer hidden dimension: 256<br>Learning rate: 0.0008401731273741588<br>Batch size:32<br>Epochs: 235<br>k-fold splits: 5<br>number of repeats: 3 |
| XGBoost_classifier_random | Model type: GATv2Classifier<br>Split type: random<br>{<br>"mordred": {<br>"fp_type": "mordred", |

| | |
|---|---|
| | "n_estimators": 1500,<br>"max_depth": 10,<br>"learning_rate": 0.031486170398999376,<br>"subsample": 0.5582330181548657,<br>"colsample_bytree": 0.693347452454977,<br>"min_child_weight": 1,<br>"gamma": 3.5976471689023346,<br>"reg_alpha": 0.0014175360348497963,<br>"reg_lambda": 0.014462580415499563<br>},<br>"map": {<br>"fp_type": "map",<br>"n_estimators": 1300,<br>"max_depth": 7,<br>"learning_rate": 0.08485430190997417,<br>"subsample": 0.7778245492737748,<br>"colsample_bytree": 0.8746696788598094,<br>"min_child_weight": 2,<br>"gamma": 1.1685285928180547,<br>"reg_alpha": 0.07732776208662087,<br>"reg_lambda": 0.003824234652671362<br>},<br>"atom_pair": {<br>"fp_type": "atom_pair",<br>"n_estimators": 1300,<br>"max_depth": 12,<br>"learning_rate": 0.039633884790200496,<br>"subsample": 0.6658084625171566,<br>"colsample_bytree": 0.9155220309089538,<br>"min_child_weight": 1,<br>"gamma": 1.514055060779356,<br>"reg_alpha": 2.111593329069601e-06,<br>"reg_lambda": 5.91851873119353e-07<br>},<br>"layered": {<br>"fp_type": "layered",<br>"n_estimators": 300,<br>"max_depth": 8,<br>"learning_rate": 0.08432952262963654,<br>"subsample": 0.849471335172441,<br>"colsample_bytree": 0.6407220063216659,<br>"min_child_weight": 1,<br>"gamma": 0.3144416035463985, |

| | |
|---|---|
| | "reg_alpha": 4.677451066046656e-06,<br>"reg_lambda": 2.782542042341084e-06<br>},<br>"rdkit": {<br>"fp_type": "rdkit",<br>"n_estimators": 1500,<br>"max_depth": 9,<br>"learning_rate": 0.05560026540900335,<br>"subsample": 0.7073338793838971,<br>"colsample_bytree": 0.8230826480029119,<br>"min_child_weight": 1,<br>"gamma": 0.02520464715498605,<br>"reg_alpha": 4.835183307728258e-07,<br>"reg_lambda": 5.161725451432342e-07<br>}<br>} |
| XGBoost_classifier_scaffold | Model type: XGBoost<br>Split type: Scaffold<br>{<br>"atom_pair": {<br>"fp_type": "atom_pair",<br>"n_estimators": 500,<br>"max_depth": 12,<br>"learning_rate": 0.2252676609591457,<br>"subsample": 0.8914245301735767,<br>"colsample_bytree": 0.9227362849274916,<br>"min_child_weight": 5,<br>"gamma": 0.17155659300385515,<br>"reg_alpha": 4.012475167509174e-06,<br>"reg_lambda": 7.061523689928079e-07<br>},<br>"estate": {<br>"fp_type": "estate",<br>"n_estimators": 500,<br>"max_depth": 8,<br>"learning_rate": 0.2581435651469605,<br>"subsample": 0.5121468703862474,<br>"colsample_bytree": 0.7545536011908952,<br>"min_child_weight": 10,<br>"gamma": 2.928675493341906,<br>"reg_alpha": 0.0008302818959886782,<br>"reg_lambda": 0.00025296745746954314<br>}, |

| | |
|---|---|
| | "autocorr": {<br>"fp_type": "autocorr",<br>"n_estimators": 500,<br>"max_depth": 3,<br>"learning_rate": 0.16192202099242461,<br>"subsample": 0.5878883162992878,<br>"colsample_bytree": 0.8012978303987651,<br>"min_child_weight": 7,<br>"gamma": 0.2899015059505158,<br>"reg_alpha": 0.006131395879664587,<br>"reg_lambda": 0.0033148885862714582<br>},<br>"mordred_3d": {<br>"fp_type": "mordred_3d",<br>"n_estimators": 1500,<br>"max_depth": 8,<br>"learning_rate": 0.26267206761930645,<br>"subsample": 0.5202521009243568,<br>"colsample_bytree": 0.5658158564399262,<br>"min_child_weight": 7,<br>"gamma": 3.9741467802626844,<br>"reg_alpha": 0.0021846357624013147,<br>"reg_lambda": 1.220676651751883e-06<br>},<br>"layered": {<br>"fp_type": "layered",<br>"n_estimators": 1300,<br>"max_depth": 11,<br>"learning_rate": 0.24074448556600725,<br>"subsample": 0.6408954049802805,<br>"colsample_bytree": 0.7071581300464582,<br>"min_child_weight": 7,<br>"gamma": 1.5480074057483273,<br>"reg_alpha": 7.153871966850925e-05,<br>"reg_lambda": 0.002319740413099216<br>}<br>} |
| XGBoost_regressor_random | Model type: xgboost<br>Split type: scaffold<br>{<br>"atom_pair": {<br>"fp_type": "atom_pair",<br>"n_estimators": 1400, |

| | |
|---|---|
| | "max_depth": 7,<br>"learning_rate": 0.01139029138018265,<br>"subsample": 0.6187762647689145,<br>"colsample_bytree": 0.5163423366099305,<br>"min_child_weight": 7,<br>"gamma": 0.0047902841587389566,<br>"reg_alpha": 3.448864897914892e-08,<br>"reg_lambda": 1.1684157039695513<br>},<br>"map": {<br>"fp_type": "map",<br>"n_estimators": 2000,<br>"max_depth": 4,<br>"learning_rate": 0.029154010688616866,<br>"subsample": 0.5064826179376595,<br>"colsample_bytree": 0.9284484509260027,<br>"min_child_weight": 2,<br>"gamma": 0.010154210459249465,<br>"reg_alpha": 0.16391342146504914,<br>"reg_lambda": 0.0707713891409971<br>},<br>"mordred": {<br>"fp_type": "mordred",<br>"n_estimators": 700,<br>"max_depth": 3,<br>"learning_rate": 0.022829069171836226,<br>"subsample": 0.853411781767488,<br>"colsample_bytree": 0.6992139228895411,<br>"min_child_weight": 2,<br>"gamma": 0.09274179346019618,<br>"reg_alpha": 0.022430192324124215,<br>"reg_lambda": 0.011924065914937148<br>},<br>"mordred_3d": {<br>"fp_type": "mordred_3d",<br>"n_estimators": 1100,<br>"max_depth": 3,<br>"learning_rate": 0.02496670085732168,<br>"subsample": 0.5044885725127056,<br>"colsample_bytree": 0.9731535218925407,<br>"min_child_weight": 5,<br>"gamma": 4.96855661647963e-05,<br>"reg_alpha": 0.046245713871923326, |

| | |
|---|---|
| | "reg_lambda": 6.5655449961715275<br>},<br>"autocorr": {<br>"fp_type": "autocorr",<br>"n_estimators": 1600,<br>"max_depth": 4,<br>"learning_rate": 0.09147304911466263,<br>"subsample": 0.5154845690832526,<br>"colsample_bytree": 0.9715306684890168,<br>"min_child_weight": 10,<br>"gamma": 0.002048768404310189,<br>"reg_alpha": 0.4150146349409657,<br>"reg_lambda": 2.3384875158828744<br>}<br>} |

**Table SI-3:** Hyperparameters of the model ensebles that contribute to iteration 2 predictions

## 3. Substructure distribution histograms

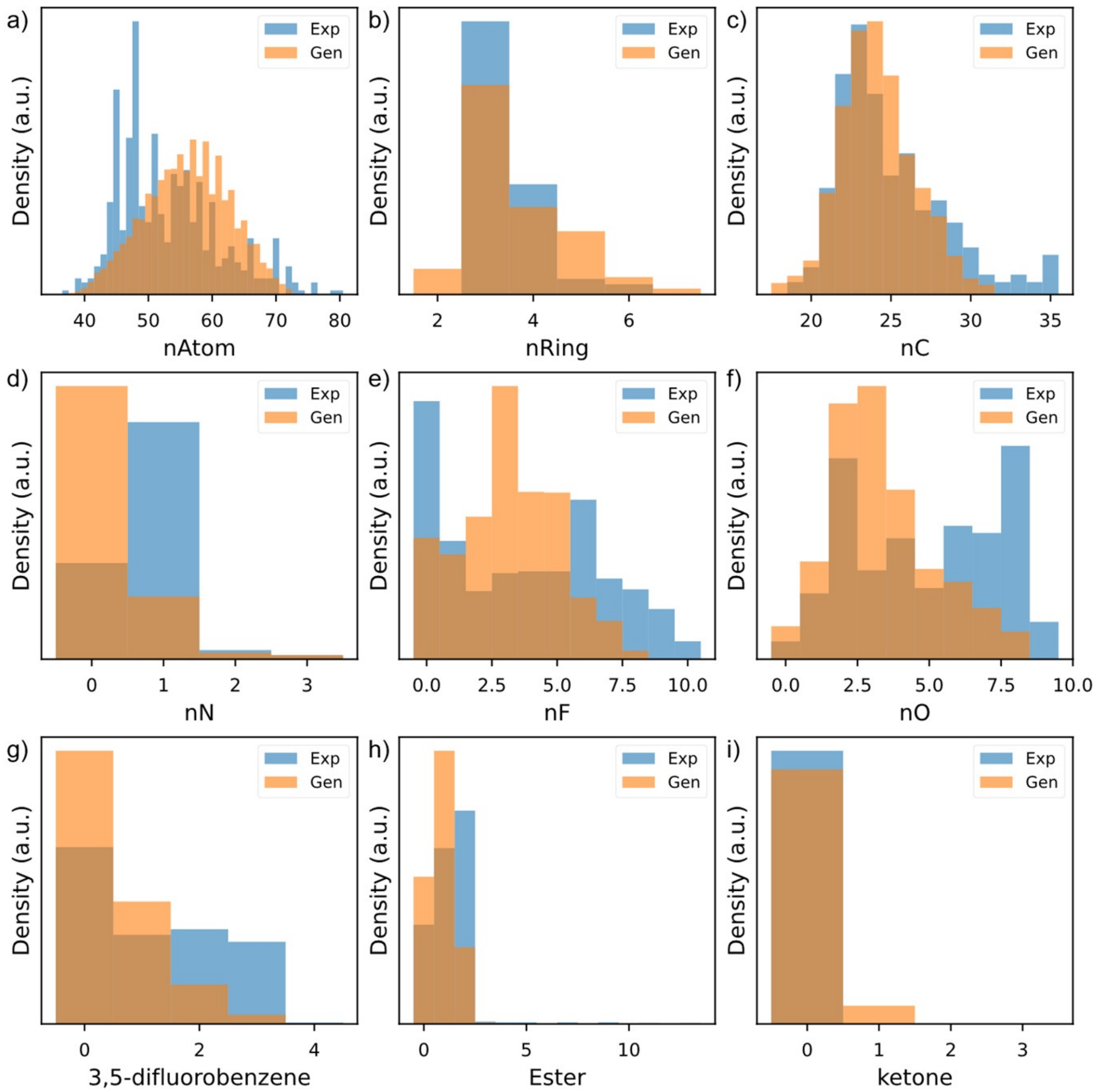


**Figure SI-10:** Comparing the per molecule probability distributions of descriptor and substructure counts for the experimental and generated dataset. A) number of atoms. B) number of rings. C) number of carbon atoms. D) number of nitrogen atoms. E) number of fluorine atoms. F) number of oxygen atoms. g) number of 3,5-difluorobenzene substructures. h) number of ester substructures. i) number of ketone substructures.

## 4. Mixture transition textures

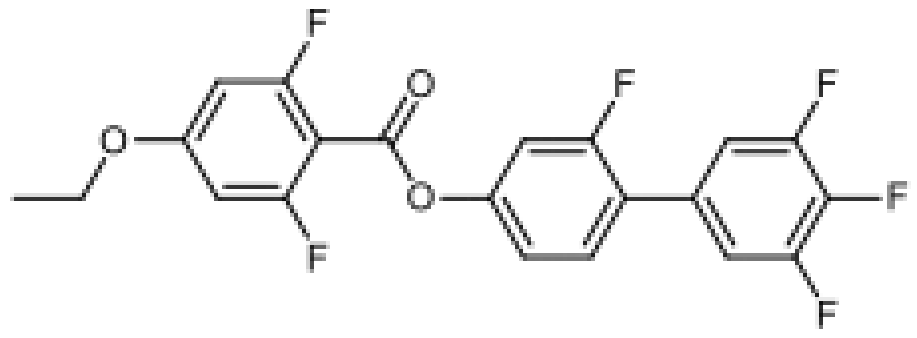

CJG484

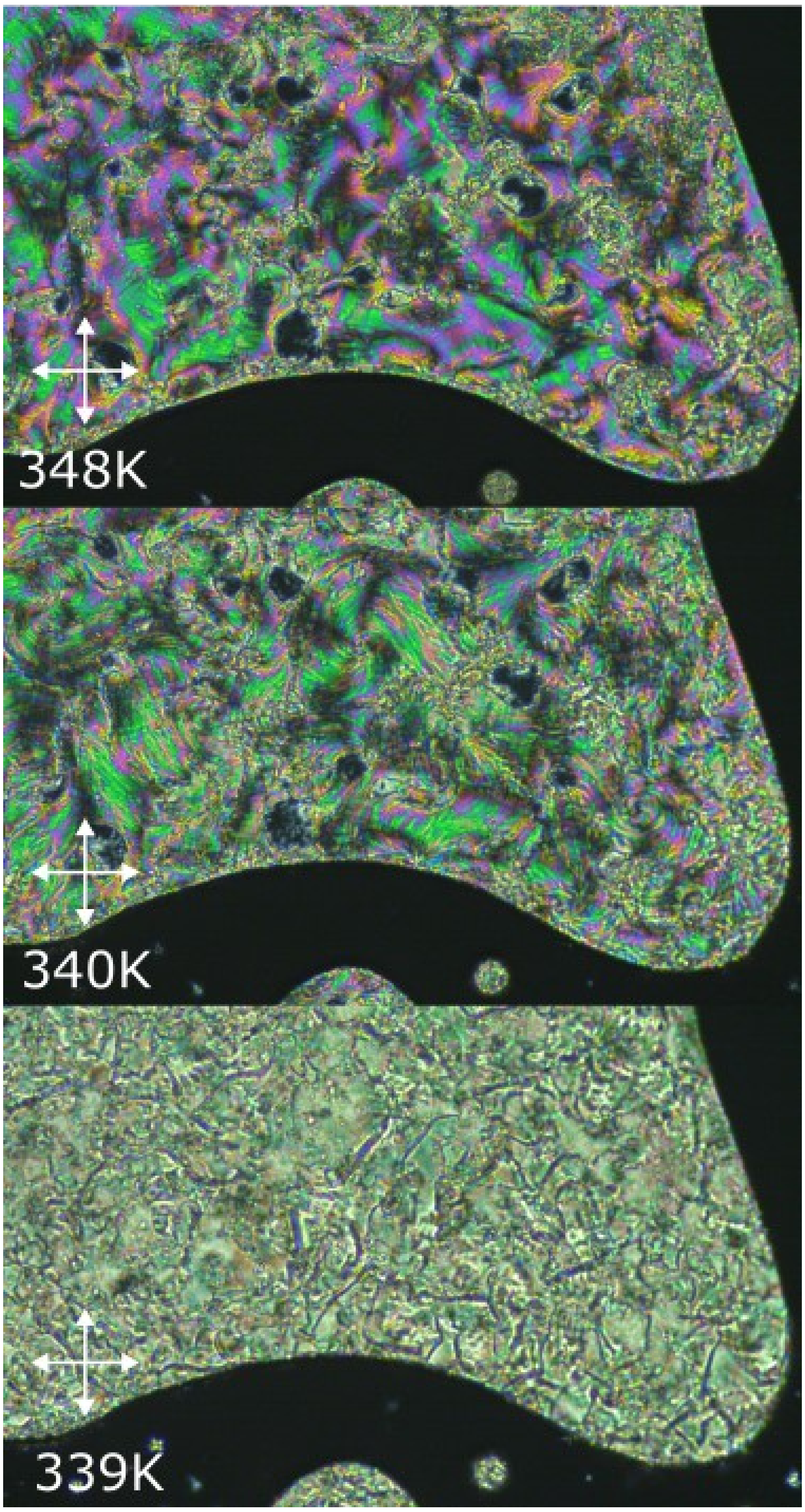


**Figure SI-11:** POM textures of a 90% wt DIO : 10% wt CJG484 mixture undergoing a transition to the $N_F$ phase. The extrapolated transition temperature of CJG484 is calculated at 333K

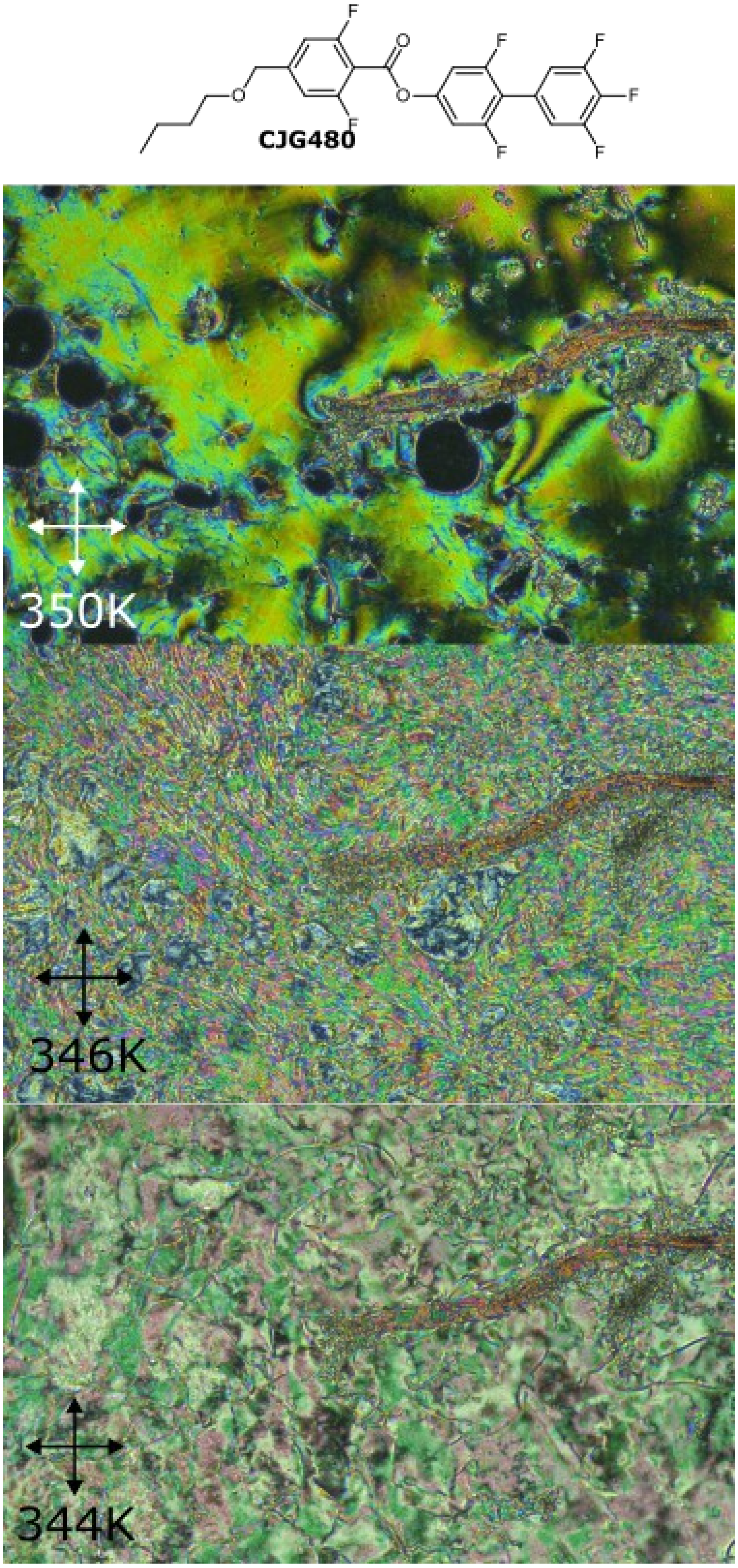


**Figure SI-12**: POM textures of a 90% wt DIO : 10% wt CJG480 mixture undergoing a transition to the $N_F$ phase. The extrapolated transition temperature of CJG480 is calculated at 377K

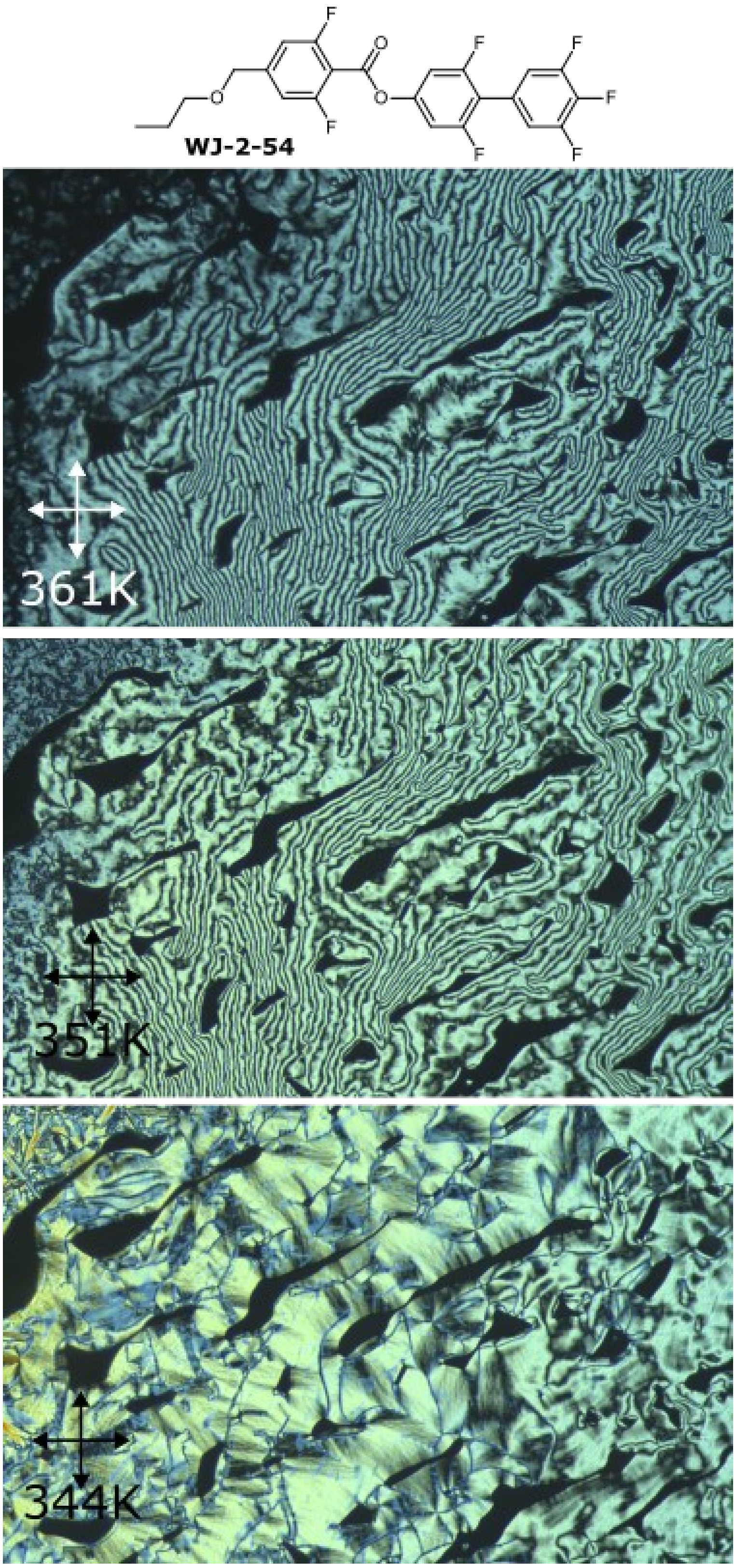


**Figure SI-13**: POM textures of a 90% wt DIO : 10% wt WJ-2-54 mixture undergoing a transition to the $N_F$ phase. The extrapolated transition temperature of WJ-2-54 is calculated at 370K

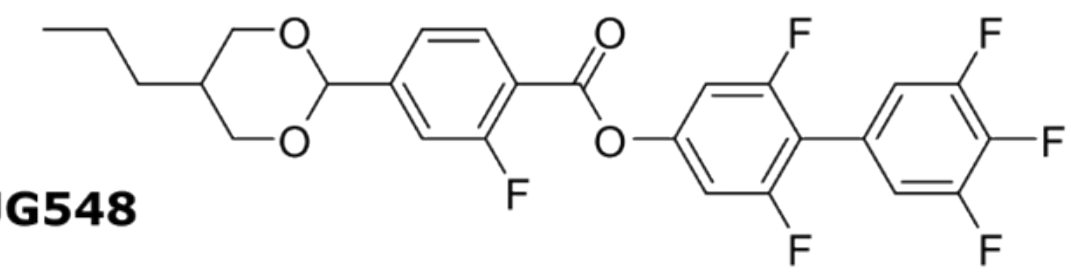

**CJG548**

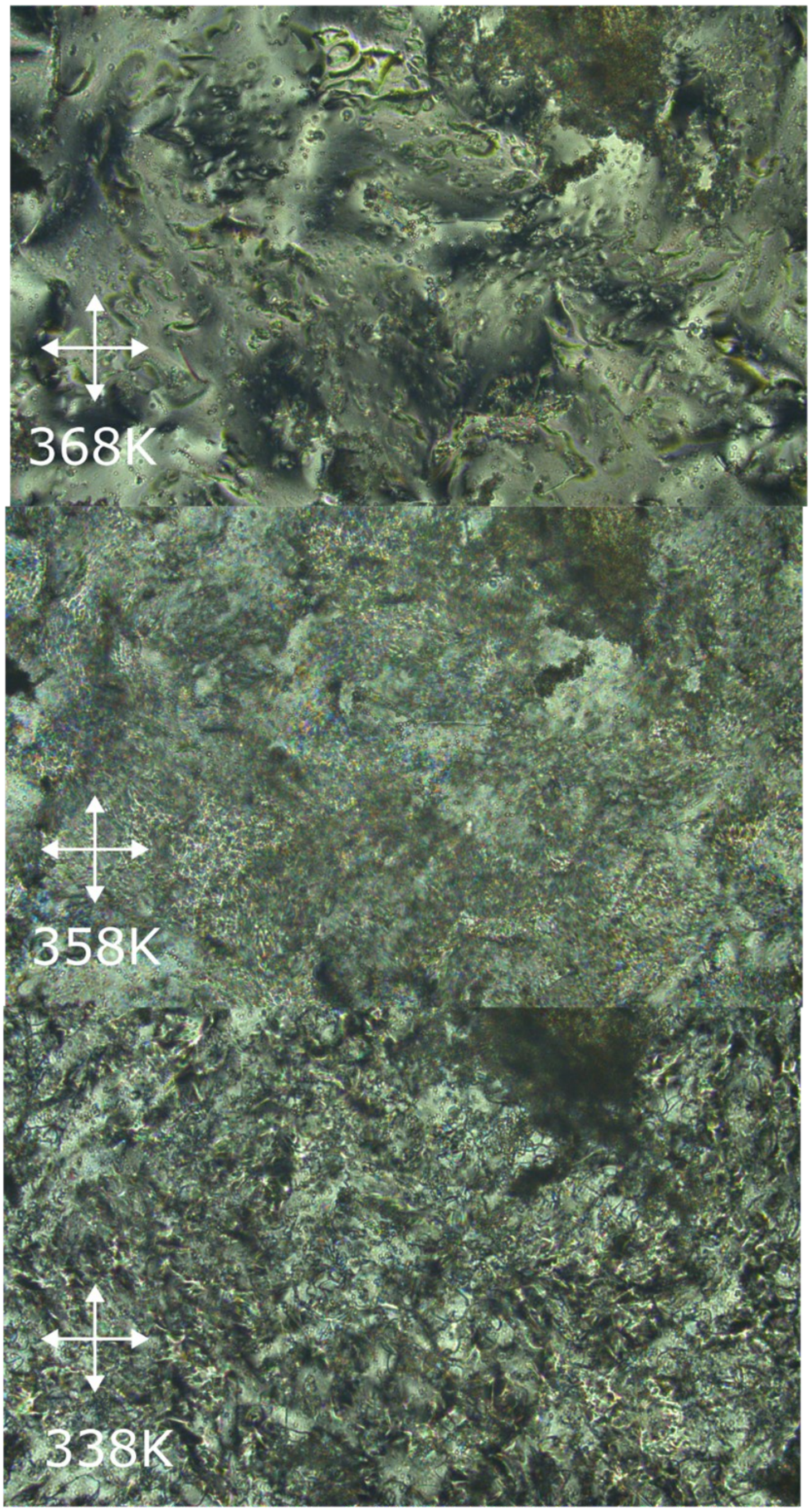


**Figure SI-14**: POM textures of a 90% wt DIO : 10% wt CJG548 mixture undergoing a transition to the $N_F$ phase. The extrapolated transition temperature of CJG548 is calculated at 390K

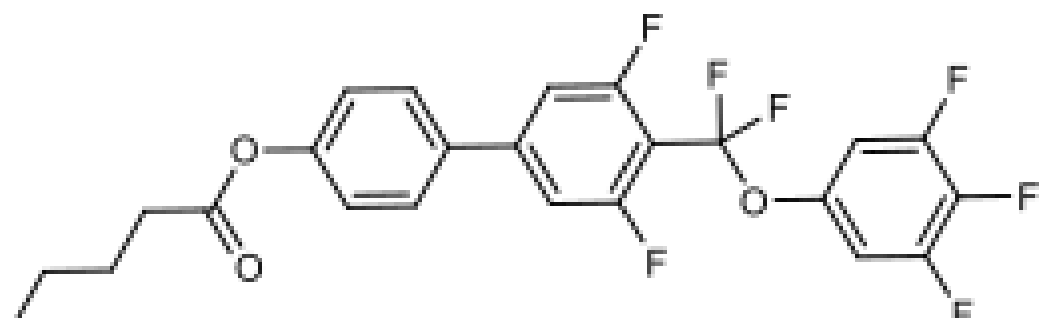


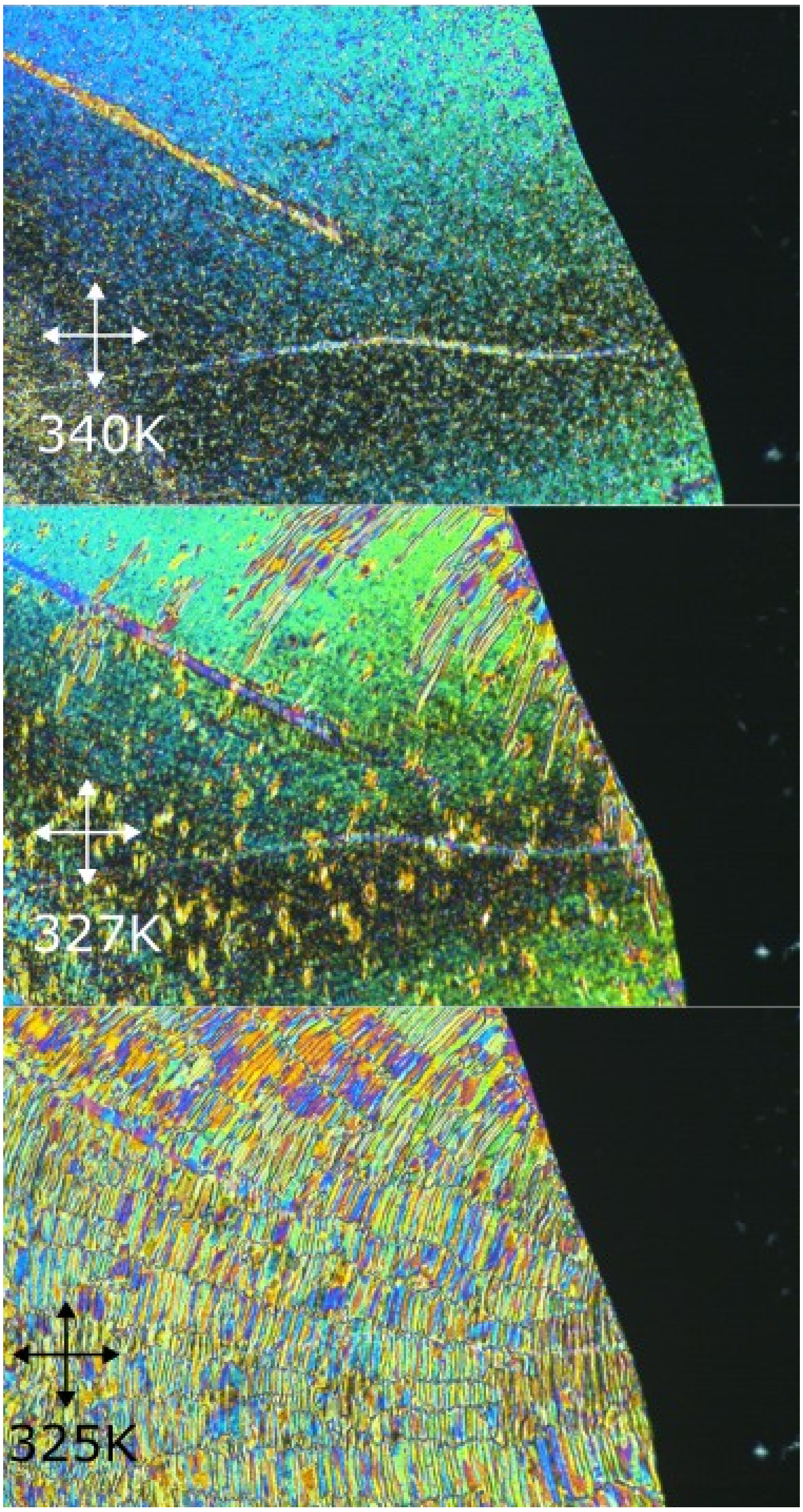


**Figure SI-15**: POM textures of a 90% wt DIO : 10% wt SRB-CHEM-082 mixture undergoing a transition to the $N_F$ phase. The extrapolated transition temperature of SRB-CHEM-082 is calculated at 280K

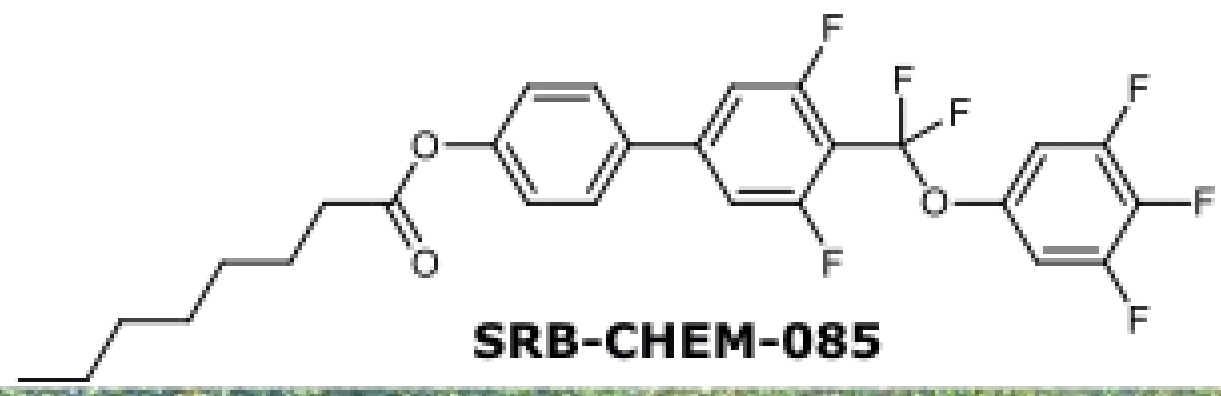

**SRB-CHEM-085**

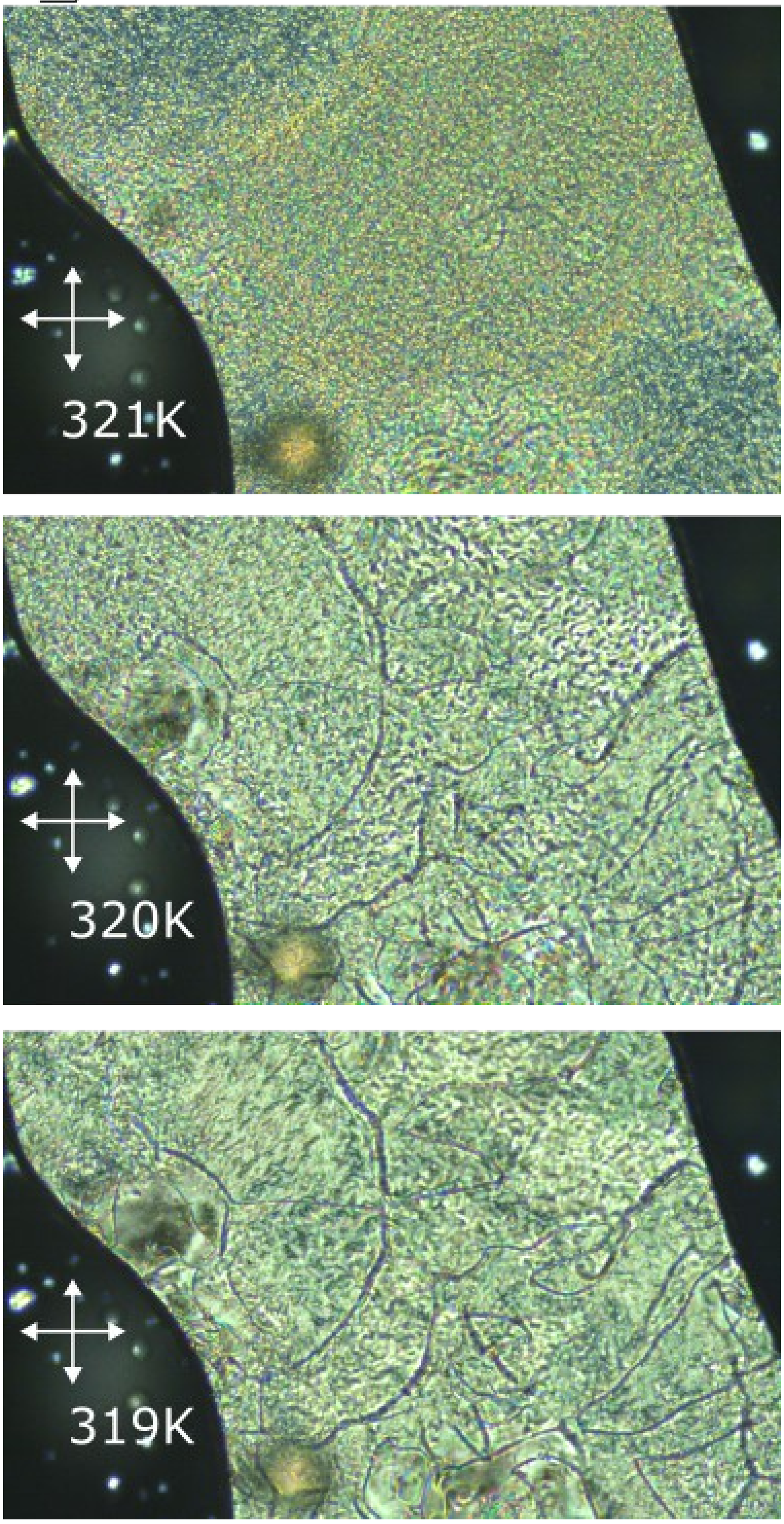


**Figure SI-16**: POM textures of a 90% wt DIO : 10% wt SRB-CHEM-085 mixture undergoing a transition to the $N_F$ phase. The extrapolated transition temperature of SRB-CHEM-085 is calculated at 200K

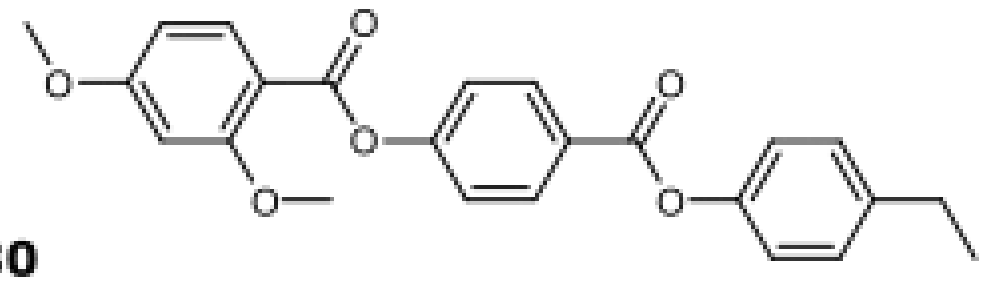

COB30

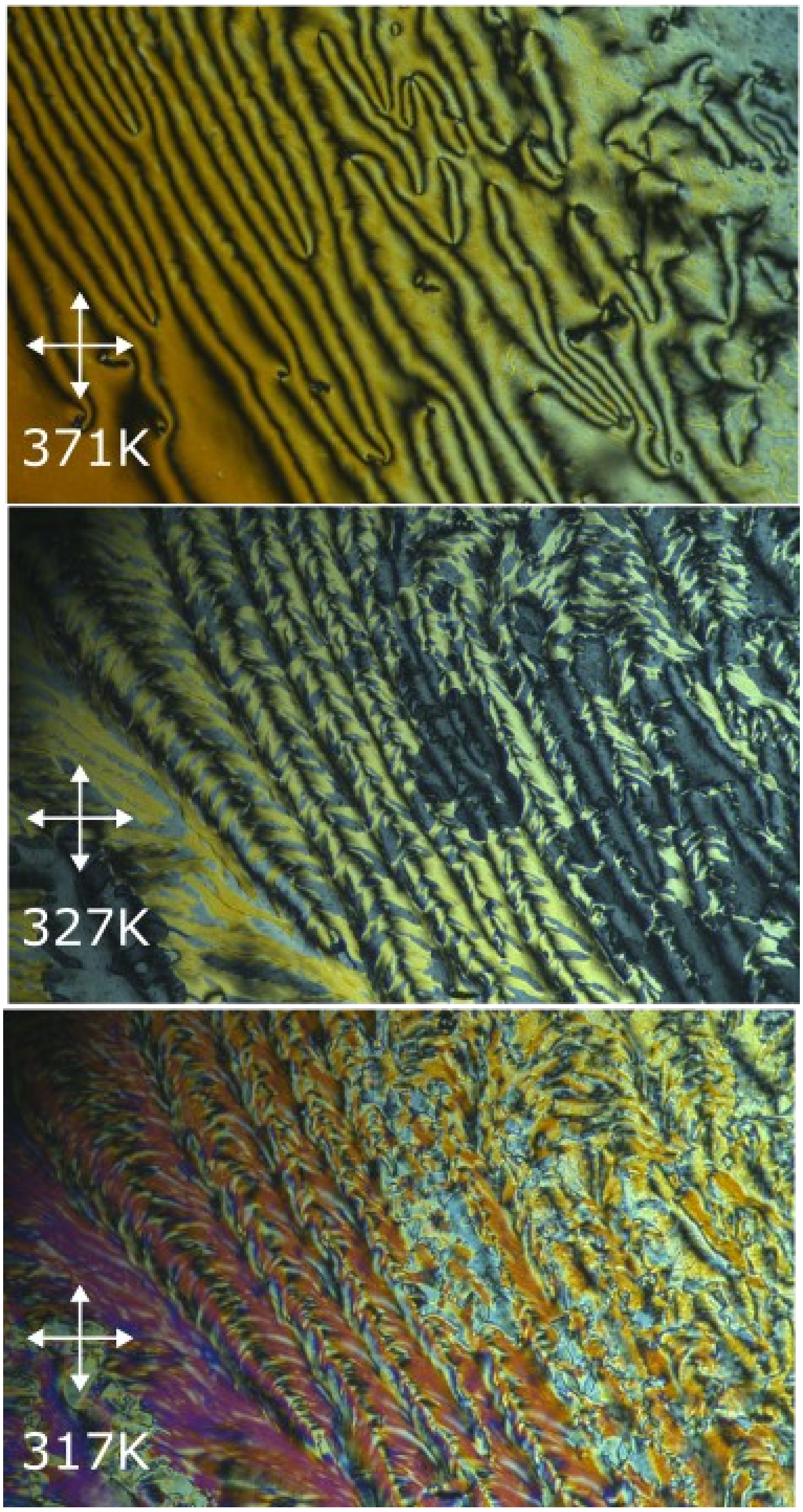


**Figure SI-17:** POM textures of a 90% wt DIO : 10% wt COB30 mixture undergoing a transition to the $N_F$ phase. The extrapolated transition temperature of COB30 is calculated at 180K

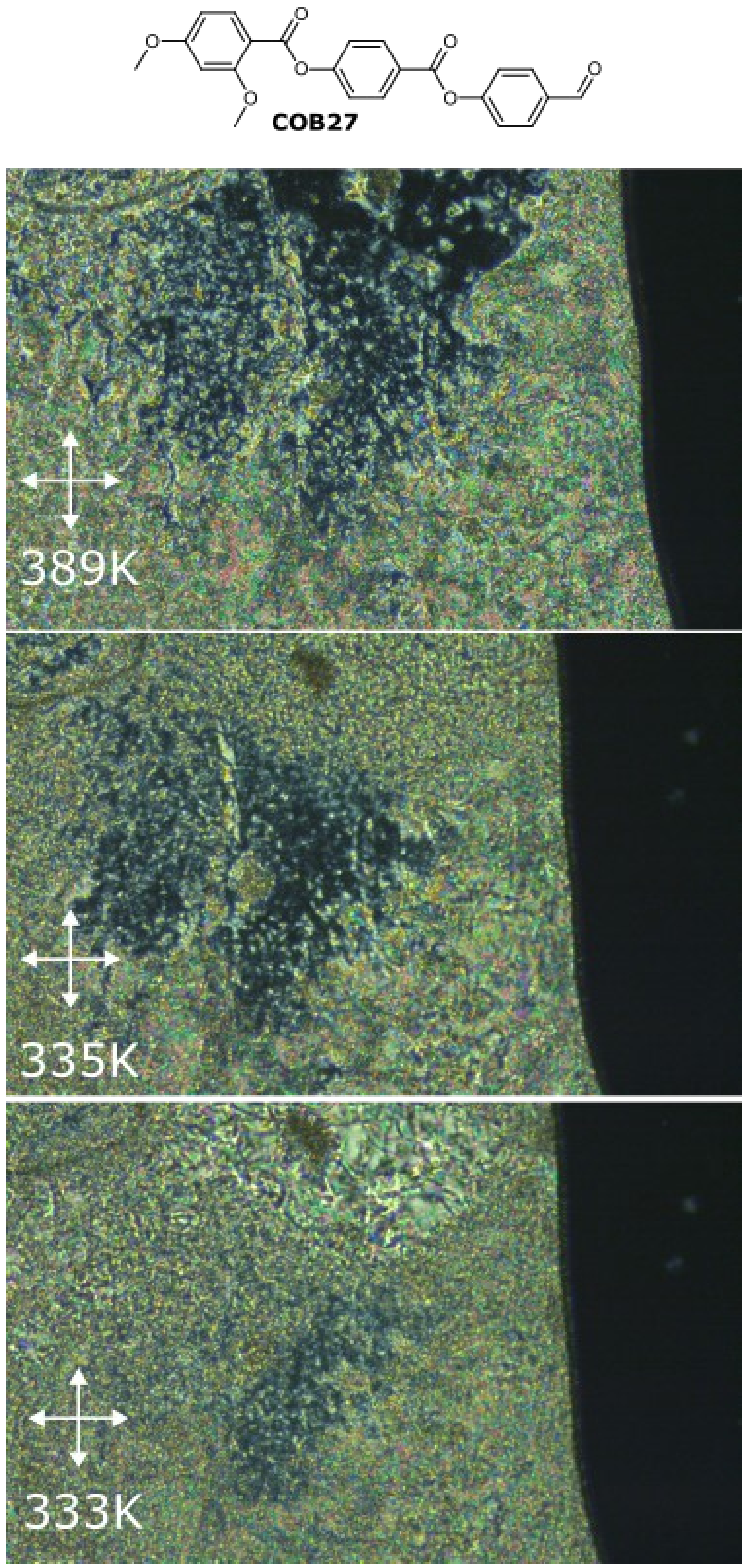


**Figure SI-18:** POM textures of a 90% wt DIO : 10% wt COB27 mixture undergoing a transition to the $N_F$ phase. The extrapolated transition temperature of COB27 is calculated at 308K

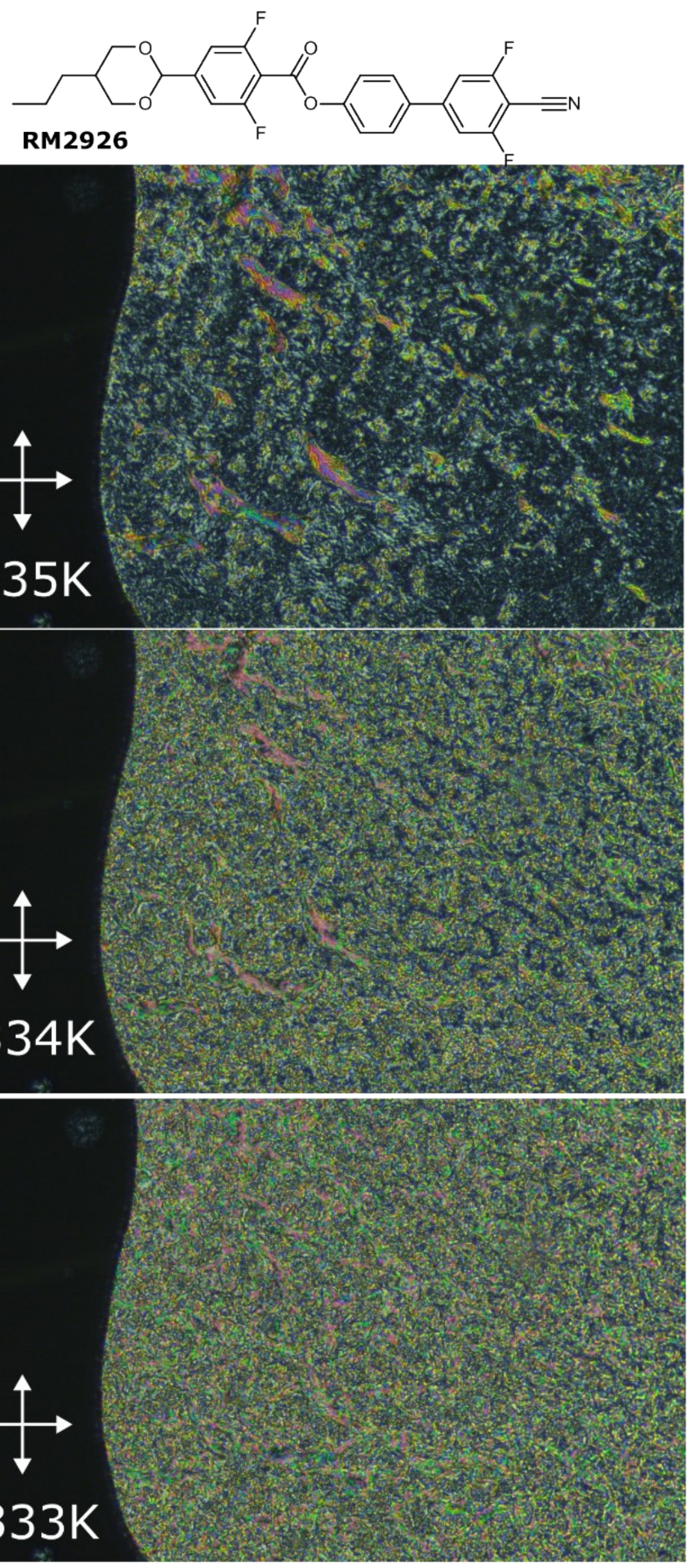


**Figure SI-19:** POM textures of a 90% wt DIO : 10% wt RM2926 mixture undergoing a transition to the $N_F$ phase. The extrapolated transition temperature of COB27 is calculated at 308K

Three distinct featurisation approaches used in this work. Graph representations are constructed from molecular SMILES strings using RDKit and python code to determine their node and edge matrices. Nodes represent the heavy atoms of a molecule and the edge matrix its covalent bonds. Hydrogen atoms are considered implicitly and encoded as a node feature. The node feature vectors are built from one hot encodings of atom types from a list of permitted atoms (liquid crystals are

organic molecules but additional atoms were included for generalisation purposes), the degree of heavy atom neighbours, the atoms formal charge, its hybridisation and the implicit hydrogen count. Ring membership and aromaticity are denoted by a binary value. The atomic mass, Van der Waals radius, and covalent radius are normalised by scaling constants. The constructed feature vector has a dimension of 79 components. The bond feature vector is constructed from a one hot encoding of the bond type and its stereochemistry, and binary values for its conjugation and ring membership. The connectivity is calculated from the RDKit adjacency matrix and converted to the COO format

Fingerprints were computed using the scikit-fingerprints library [1]. Through the course of hyperparameter searching all fingerprints within the library were available to be considered.

The featurisation of the VAE input is taken from the code published in the MGCVAE paper [2]. The nodes of each molecule is represented by a hot encoded tensor of shape ($N_{max}$ , $A$) where $N_{max}$ is the maximum number of molecules, a constant defined in the training script,  and $A$ is atom type encoded from coders that create a feature vector from every atom type in the training dataset. Each row indicates the element of atom I with rows beyond the molecule size assigned zero values to indicate no atoms. Bonds are encoded as a one hot encoded tensor of shape $(N_{max}, N_{max} \cdot |B|)$ where $B$ is the number of bond types in the dataset. The final tensor for each molecule concatenates the atom, bond and a length tensor of shape ($N_{max}$, 1)

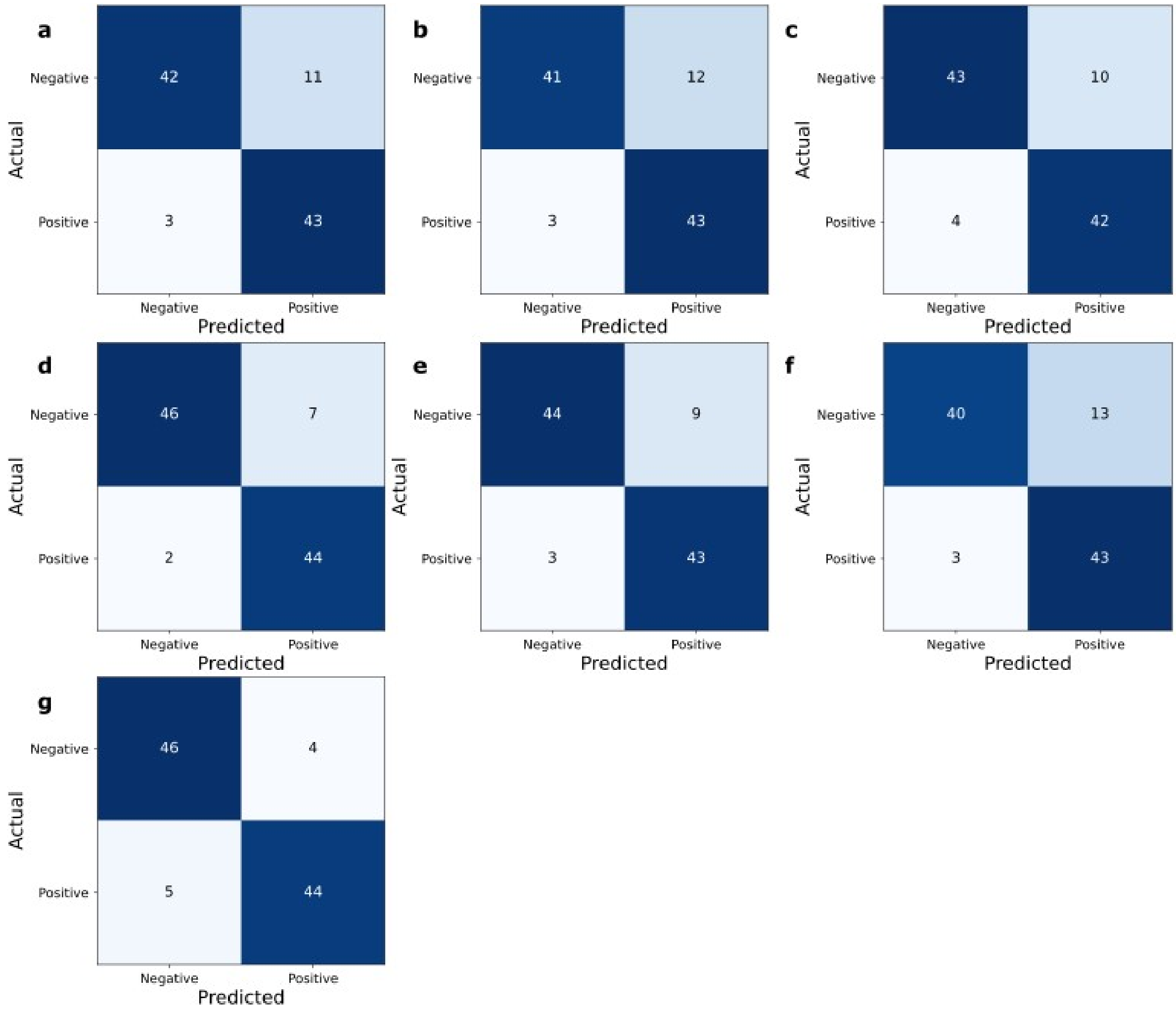


**Figure SI-20:** Confusion matrices of the random split trained a) GAT2, b) GAT, c) GCN, d) GIN, e) GG, f) TransformerConv, g) XGBoost classifiers

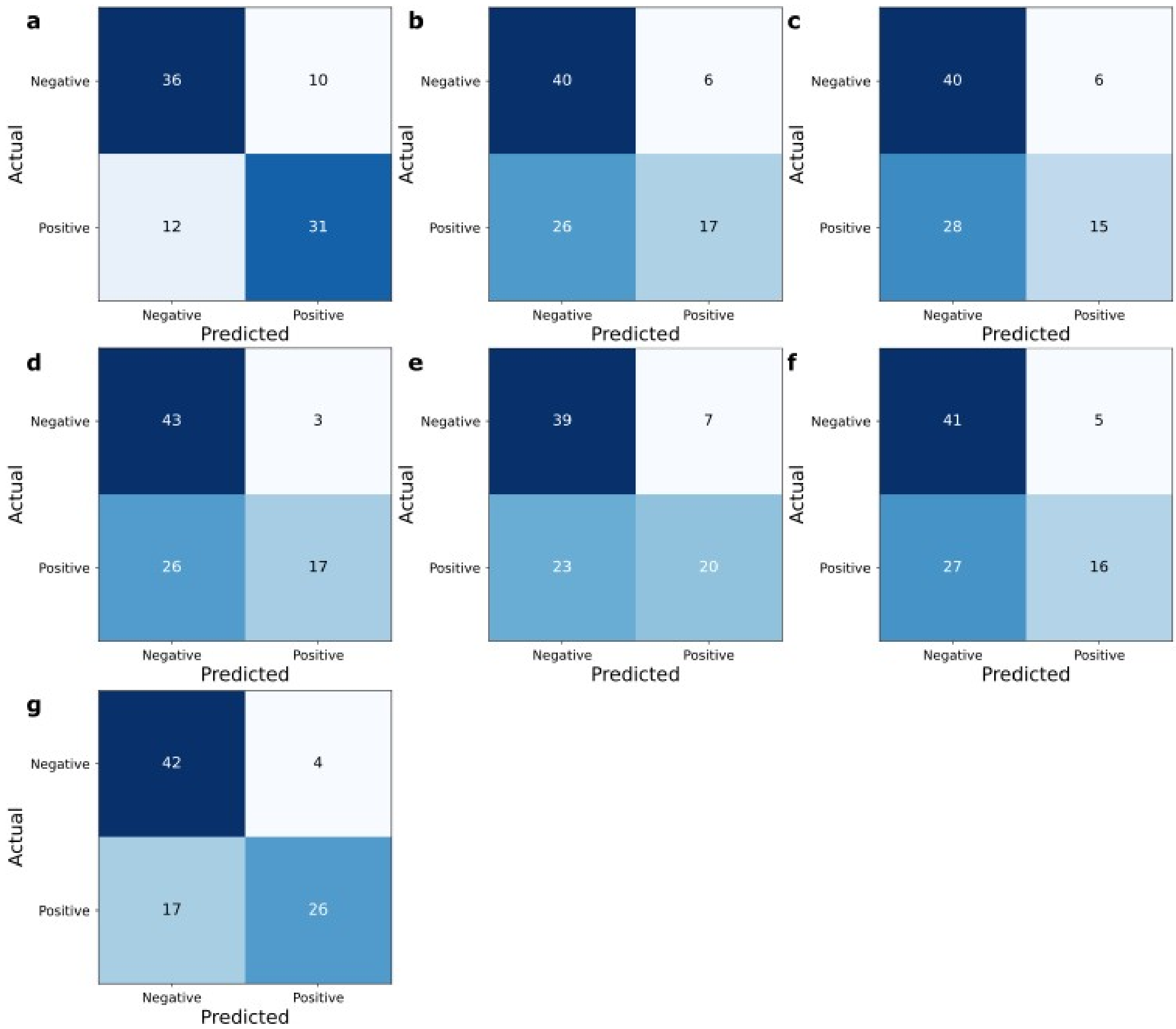


**Figure SI-21:** Confusion matrices of the scaffold split trained a) GAT2, b) GAT, c) GCN, d) GIN, e) GG, f) TransformerConv, g) XGBoost classifiers

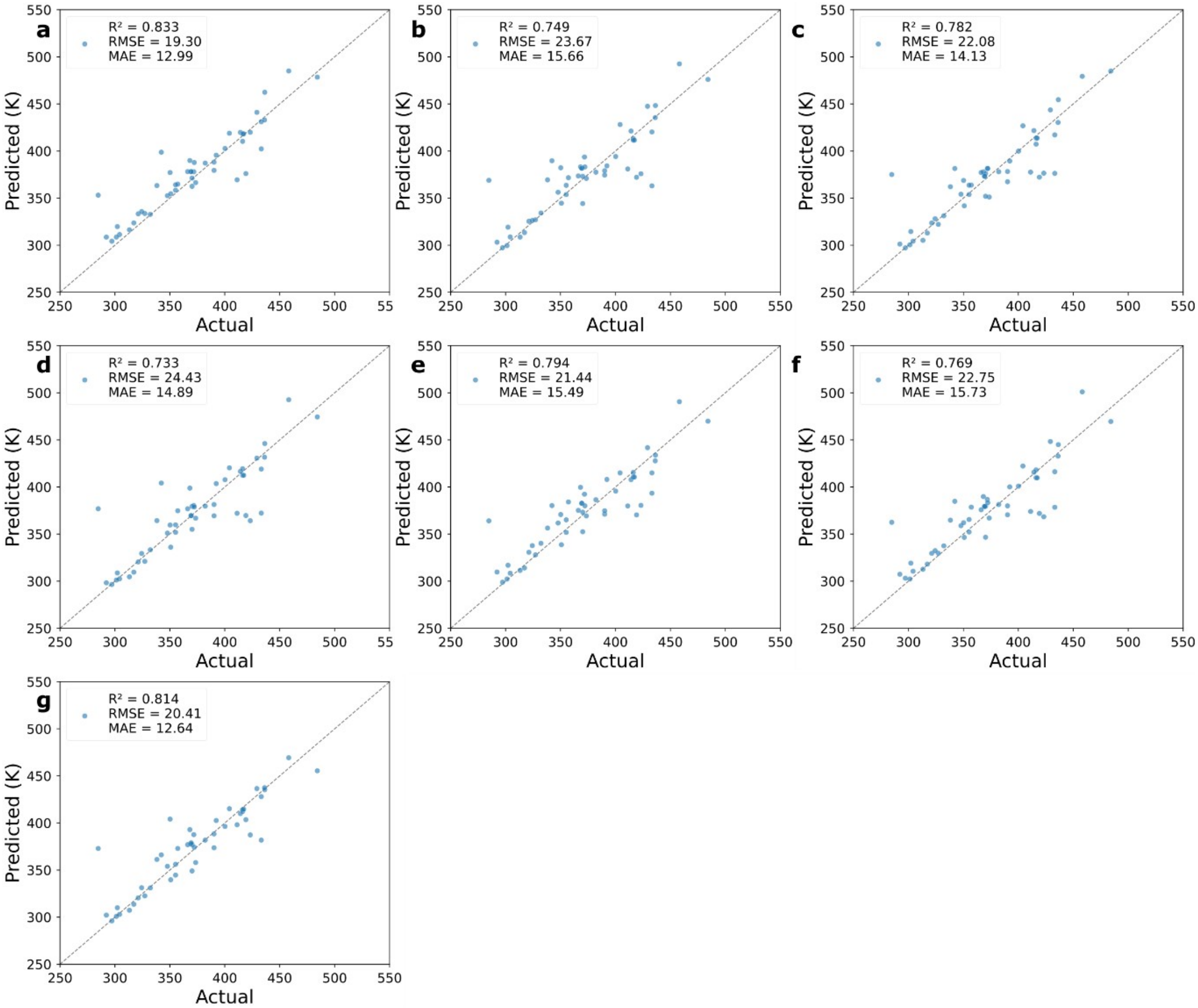


**Figure SI-22:** Predicted vs actual plots of the random split trained a) GAT2, b) GAT, c) GCN, d) GIN, e) GG, f) TransformerConv, g) XGBoost regressors

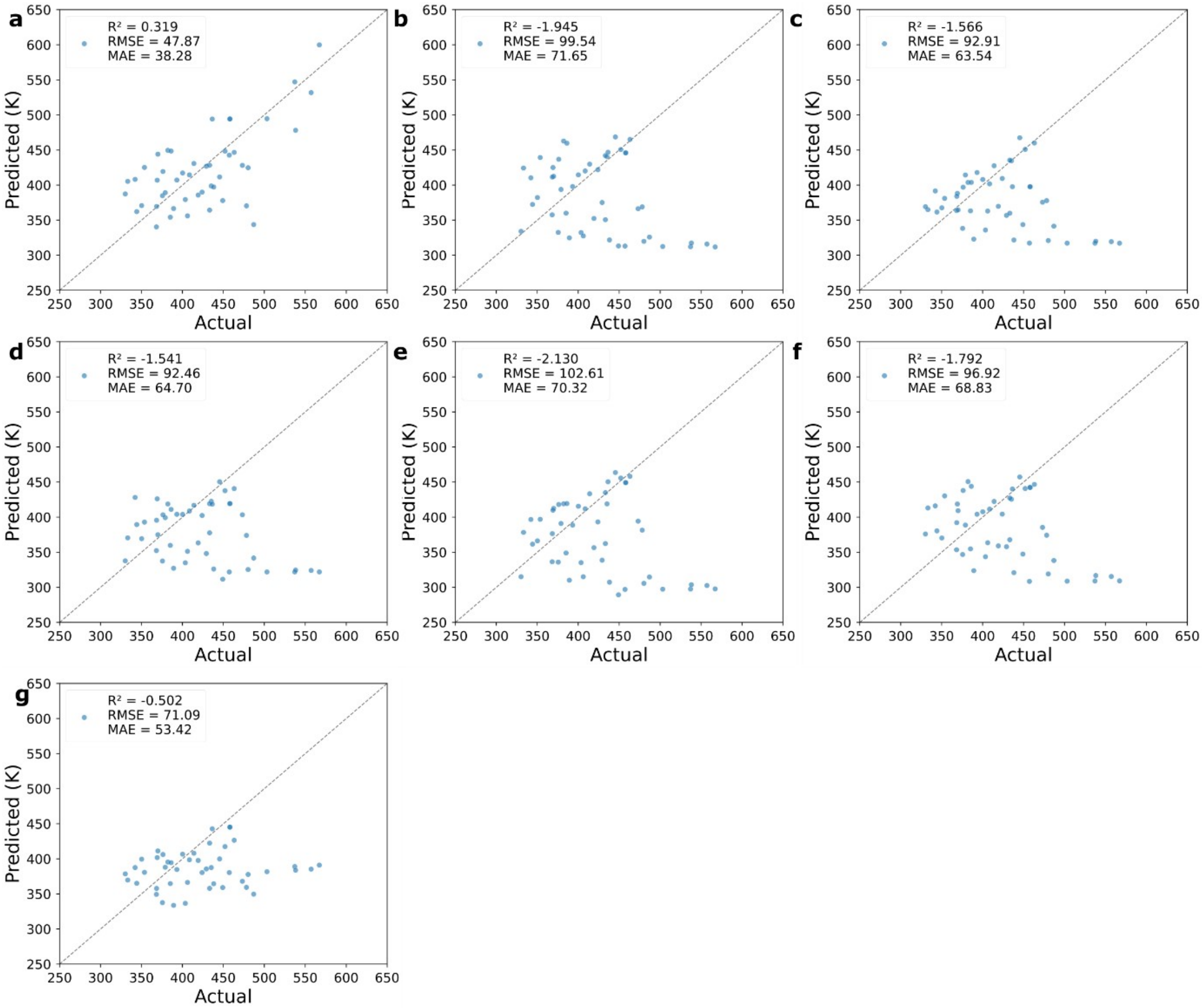


**Figure SI-23:** Predicted vs actual plots of the scaffold split trained a) GAT2, b) GAT, c) GCN, d) GIN, e) GG, f) TransformerConv, g) XGBoost regressors

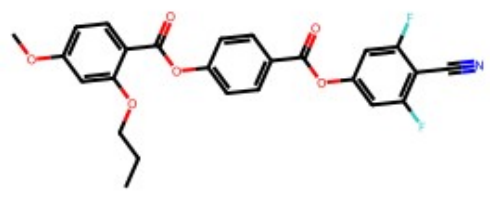

CCCOc1cc(OC)ccc1C(=O)Oc1ccc(C(=O)Oc2cc(F)c(C#N)c(F)c2)cc1
XGBoost (375.3) Transformer (394.1)

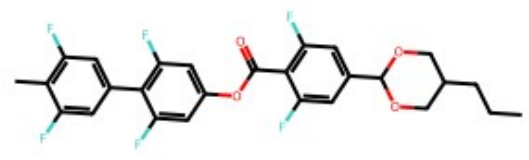

CCCC1COC(c2cc(F)c(C(=O)Oc3cc(F)c(-c4cc(F)c(C)c(F)c4)c(F)c3)c(F)c2)OC1
GAT (382.6) GG (416.2)

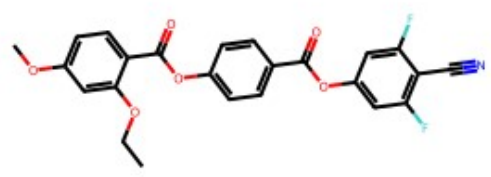

CCOc1cc(OC)ccc1C(=O)Oc1ccc(C(=O)Oc2cc(F)c(C#N)c(F)c2)cc1
XGBoost (389.5) GIN (407.1)

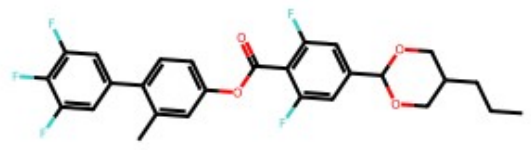

CCCC1COC(c2cc(F)c(C(=O)Oc3ccc(-c4cc(F)c(F)c(F)c4)c(C)c3)c(F)c2)OC1
GG (343.3) GIN (361.8)

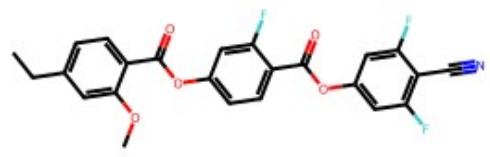

CCc1ccc(C(=O)Oc2ccc(C(=O)Oc3cc(F)c(C#N)c(F)c3)c(F)c2)c(OC)c1
GIN (377.1) GAT2 (410.3)

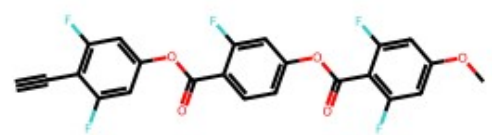

C#Cc1c(F)cc(OC(=O)c2ccc(OC(=O)c3c(F)cc(OC)cc3F)cc2F)cc1F
GAT (424.6) GG (469.2)

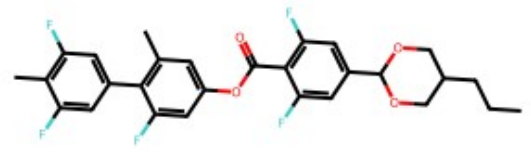

CCCC1COC(c2cc(F)c(C(=O)Oc3cc(C)c(-c4cc(F)c(C)c(F)c4)c(F)c3)c(F)c2)OC1
Transformer (363.3) GAT2 (386.8)

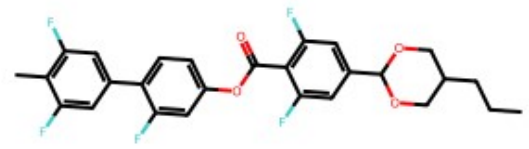

CCCC1COC(c2cc(F)c(C(=O)Oc3ccc(-c4cc(F)c(C)c(F)c4)c(F)c3)c(F)c2)OC1
GCN (347.0) GG (375.3)

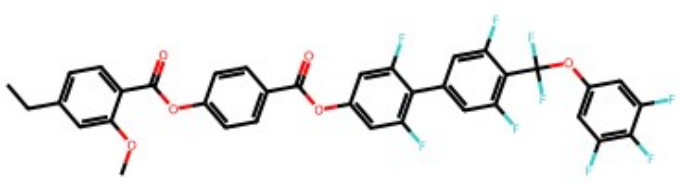

CCc1ccc(C(=O)Oc2ccc(C(=O)Oc3cc(F)c(-c4cc(F)c(C(F)(F)Oc5cc(F)c(F)c(F)c5)c(F)c4)c(F)c3)cc2)c(OC)c1
GIN (436.9) GAT2 (471.3)

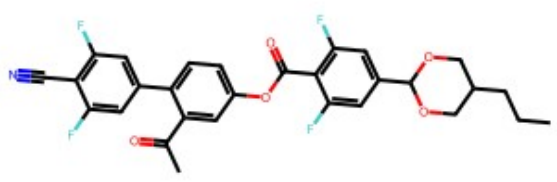

CCCC1COC(c2cc(F)c(C(=O)Oc3ccc(-c4cc(F)c(C#N)c(F)c4)c(C(C)=O)c3)c(F)c2)OC1
GIN (356.8) GG (378.0)

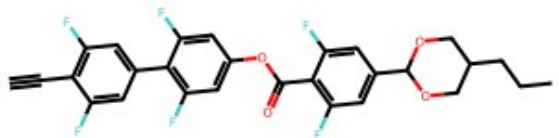

C#Cc1c(F)cc(-c2c(F)cc(OC(=O)c3c(F)cc(C4OCC(CCC)CO4)cc3F)cc2F)cc1F
GAT (376.4) XGBoost (422.1)

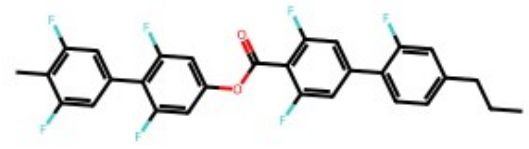

CCCc1ccc(-c2cc(F)c(C(=O)Oc3cc(F)c(-c4cc(F)c(C)c(F)c4)c(F)c3)c(F)c2)c(F)c1
GCN (390.8) XGBoost (414.5)

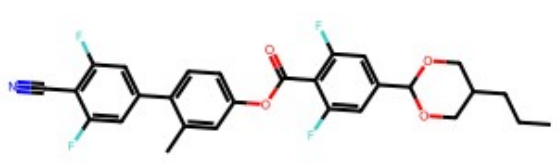

CCCC1COC(c2cc(F)c(C(=O)Oc3ccc(-c4cc(F)c(C#N)c(F)c4)c(C)c3)c(F)c2)OC1
GG (357.1) GIN (368.9)

**Figure SI-24:** The candidate pool of molecules validated by a consensus across the seven random split trained molecule and a predicted retrosynthetic route. The min-max temperature range along with model responsible for the prediction is also shown

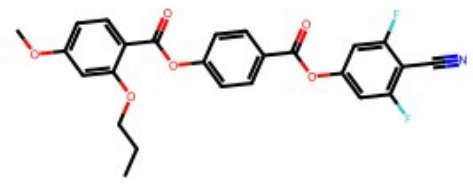

CCCOc1cc(OC)ccc1C(=O)Oc1ccc(C(=O)Oc2cc(F)c(C#N)c(F)c2)cc1
371.5 392.1

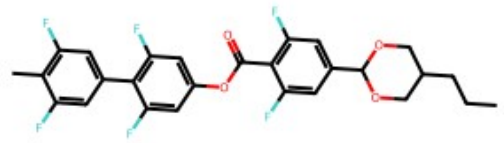

CCCC1COC(c2cc(F)c(C(=O)Oc3cc(F)c(-c4cc(F)c(C)c(F)c4)c(F)c3)c(F)c2)OC1
381.0 411.5

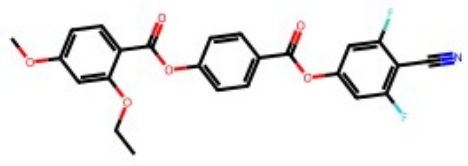

CCOc1cc(OC)ccc1C(=O)Oc1ccc(C(=O)Oc2cc(F)c(C#N)c(F)c2)cc1
392.2 410.2

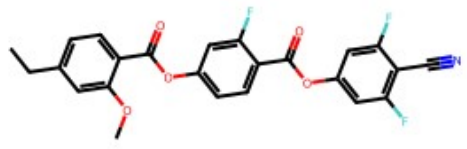

CCc1ccc(C(=O)Oc2ccc(C(=O)Oc3cc(F)c(C#N)c(F)c3)c(F)c2)c(OC)c1
394.7 414.6

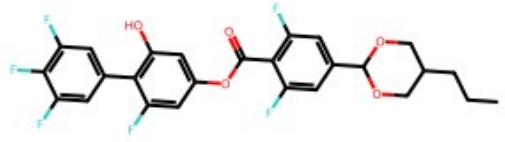

CCCC1COC(c2cc(F)c(C(=O)Oc3cc(O)c(-c4cc(F)c(F)c(F)c4)c(F)c3)c(F)c2)OC1
341.1 380.7

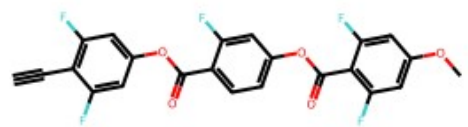

C#Cc1c(F)cc(OC(=O)c2ccc(OC(=O)c3c(F)cc(OC)cc3F)cc2F)cc1F
443.2 469.0

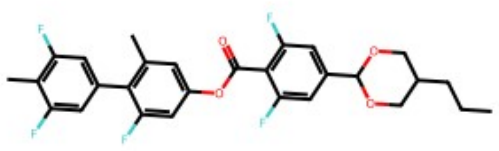

CCCC1COC(c2cc(F)c(C(=O)Oc3cc(C)c(-c4cc(F)c(C)c(F)c4)c(F)c3)c(F)c2)OC1
351.6 381.6

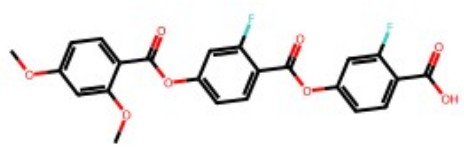

COc1ccc(C(=O)Oc2ccc(C(=O)Oc3ccc(C(=O)O)c(F)c3)c(F)c2)c(OC)c1
380.6 417.2

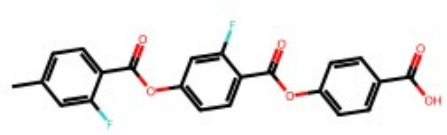

Cc1ccc(C(=O)Oc2ccc(C(=O)Oc3ccc(C(=O)O)cc3)c(F)c2)c(F)c1
372.4 423.7

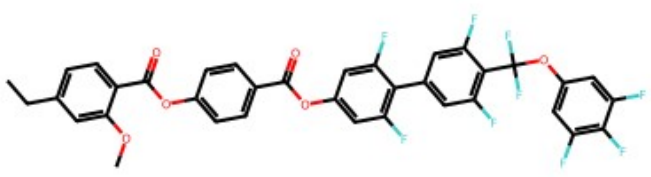

CCc1ccc(C(=O)Oc2ccc(C(=O)Oc3cc(F)c(-c4cc(F)c(C(F)(F)Oc5cc(F)c(F)c(F)c5)c(F)c4)c(F)c3)cc2)c(OC)c1
448.6 479.2

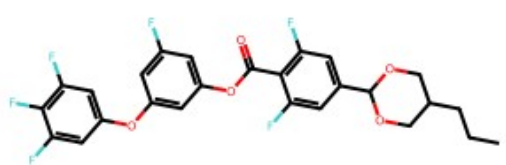

CCCC1COC(c2cc(F)c(C(=O)Oc3cc(F)cc(Oc4cc(F)c(F)c(F)c4)c3)c(F)c2)OC1
365.8 392.4

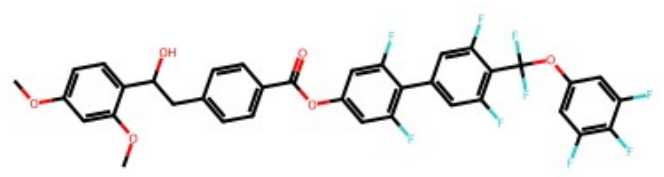

COc1ccc(C(O)Cc2ccc(C(=O)Oc3cc(F)c(-c4cc(F)c(C(F)(F)Oc5cc(F)c(F)c(F)c5)c(F)c4)c(F)c3)cc2)c(OC)c1
421.4 450.1

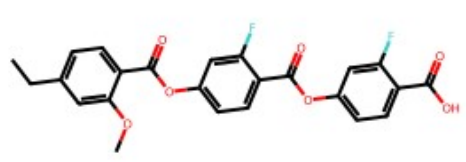

CCc1ccc(C(=O)Oc2ccc(C(=O)Oc3ccc(C(=O)O)c(F)c3)c(F)c2)c(OC)c1
352.3 390.8

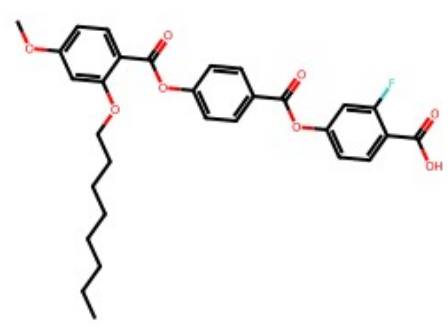

CCCCCCCCOc1cc(OC)ccc1C(=O)Oc1ccc(C(=O)Oc2ccc(C(=O)O)c(F)c2)cc1
295.3 320.3

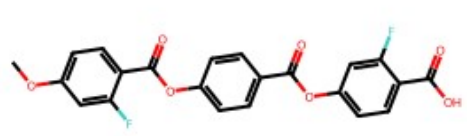

COc1ccc(C(=O)Oc2ccc(C(=O)Oc3ccc(C(=O)O)c(F)c3)cc2)c(F)c1
358.2 410.8

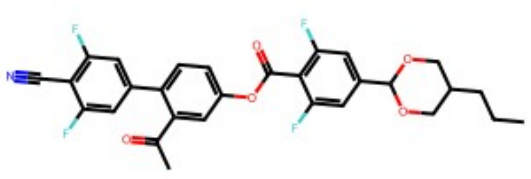

CCCC1COC(c2cc(F)c(C(=O)Oc3ccc(-c4cc(F)c(C#N)c(F)c4)c(C(C)=O)c3)c(F)c2)OC1
347.8 384.0

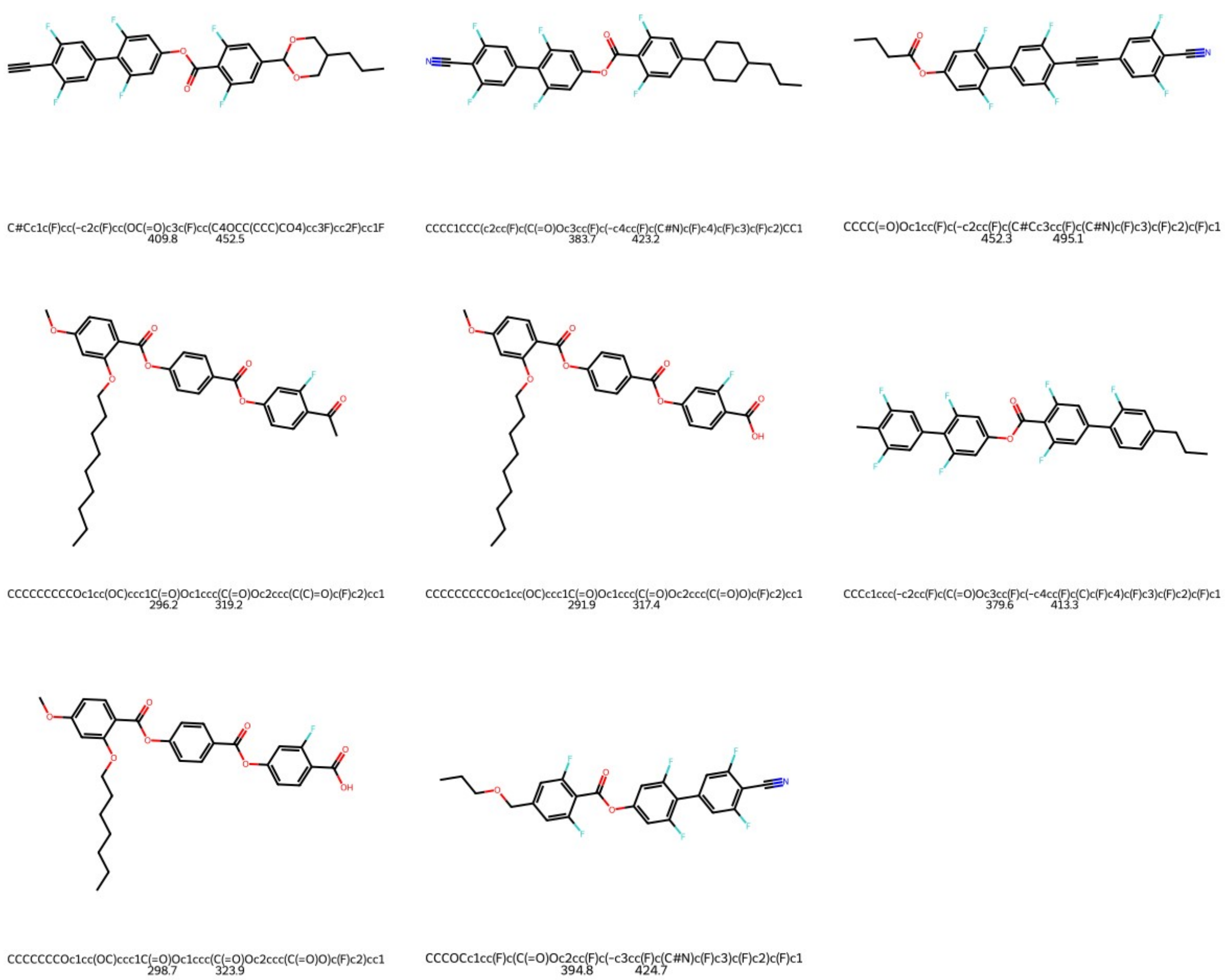


**Figure SI-25:** The candidate set produced by scaffold split GATv2 and XGBoost classifiers with 60% ensemble agreement and a valid retrosynthetic route. The range of their predicted NF transition (K) is given as the standard deviation of the ensemble predictions from their mean prediction.

### 8.1. Chemical Synthesis

Chemicals were purchased from commercial suppliers (Fluorochem, Apollo Scientific, Merck, ChemScene, Ambeed) and used as received. Solvents were purchased from Merck and used without further purification. Anhydrous solvents were obtained by percolation through activate alumina in a standard solvent purification system. Reactions were performed in standard laboratory glassware at ambient temperature and atmosphere (unless noted otherwise) and were monitored by TLC with an appropriate eluent and visualised with 254 nm light. Chromatographic purification was performed using a Combiflash NextGen 300+ System (Teledyne Isco) with a silica gel stationary phase and a binary solvent gradient as the mobile phase, with detection

made in the 200-800 nm range. Chromatographed materials were subjected to re-crystallisation from an appropriate solvent system.

## 8.2. Chemical Characterisation Methods

The structures of intermediates and final products were determined using $^{1}H$, $^{13}C\{^{1}H\}$, and $^{19}F$ NMR spectroscopy. NMR spectroscopy was performed using either a Bruker Avance III HDNMR spectrometer operating at 400 MHz, 100.5 MHz or 376.4 MHz ($^{1}H$, $^{13}C\{^{1}H\}$ and $^{19}F$, respectively) or  a Bruker AV4 NEO 11.75T spectrometer operating at 500 MHz, 125.5 MHz or 470.5 MHz ($^{1}H$, $^{13}C\{^{1}H\}$ and $^{19}F$, respectively).High resolution mass spectrometry data (HRMS) was collected using a Bruker MaXis Impact spectrometer with a positive ESI source (VIP-HESI) or a Bruker Impact QqTOF instrument acquired with positive DIP APCI source. In both, the sample was introduced *via* direct infusion as solution in acetonitrile. HPLC analysis was performed using an Agilent 1290 Infinity II system fitted with a poroshell 120 ec-C18 column running a $H_2O$:MeCN gradient.

## 8.3. Mesophase Characterisation

Phase transition temperatures and associated enthalpies of transition were determined by differential scanning calorimetry (DSC) using a TA instruments Q2000 heat flux calorimeter with a liquid nitrogen cooling system for temperature control. Samples were measured with 10 ℃ $min^{-1}$ heating and cooling rates. The transition temperatures and enthalpy values reported are averages obtained for duplicate runs. Phase transition temperatures were measured on cooling cycles for consistency between monotropic and enantiotropic phase transitions, while crystal melts were obtained on heating. Phase identification by polarised optical microscopy (POM) was performed using a Leica DM 2700 P polarised optical microscope equipped with a Linkam TMS 92 heating stage. Samples were studied sandwiched between two untreated glass coverslips.

## 8.4. General Methods:

**General Method A – Steglich esterification:**

A round bottom flask or reaction vial were charged with carboxylic acid (1.2 mol eqv), phenol (1 mol eqv), EDC.HCl (1.5 mol eqv) and DMAP (2 mol%). The solids were suspended in DCM and stirred vigorously until the reaction was complete (1-48h) as judged by TLC. The reaction mass was dry loaded onto celite and purified by flash chromatography over silica with a suitable solvent gradient. The chromatographed material was recrystalised from a suitable solvent to afford the target material.

**General Method B – Carboxylation:**

An oven dried flask was cooled under an atmosphere of dry nitrogen, charged with the compound to be lithiated (1 mol eqv), anhydrous THF (~ 0.2 M), and cooled to -78 °C with an external $CO_2$/acetone bath. A solution of n-butyl lithium (1.6 M in hexane, 1.2 mol eqv.) was added dropwise. Once the addition was complete, the solution was allowed to stir for 0.5h. Separately, a stream of $CO_2$ gas was dried by bubbling through neat $H_2SO_4$ and was introduced to the reaction with vigorous stirring for 20 minutes. The cooling bath was then removed, and the reaction mixture allowed to warm to ambient temperature over the course of 2h. The solution was acidified with 2M HCl. The organic layer was separated and retained, the aqueous was washed with ethyl acetate and discarded. The combined organics were dried over $MgSO_4$, and concentrated *in vacuo*. The resulting material was used without further purification.

**General Method C – Suzuki-Miyaura Cross Coupling:**

A biphasic mixture of THF and aqueous 2M sodium carbonate (1:1) was degassed by sparging with dry nitrogen whilst vigorously stirring. To this was added aryl halide (1 mol eqv), boronate species (> 1 mol eqv), and finally Pd-XPHOS-G3 (~ 1 mol%). The reaction was heated under reflux under an atmosphere of dry nitrogen, and monitored by TLC. Once complete, the reaction was cooled to ambient temperature. The organic layer was separated and retained, the aqueous layer was washed with EtOAc (x2) and discarded. The combined organics were washed with saturated aqueous sodium chloride, and dried over $MgSO_4$. The volatiles were removed in vacuo; the crude material was purified by flash chromatography and/or recrystalisation as appropriate.

## 8.5. Synthetic Schemes

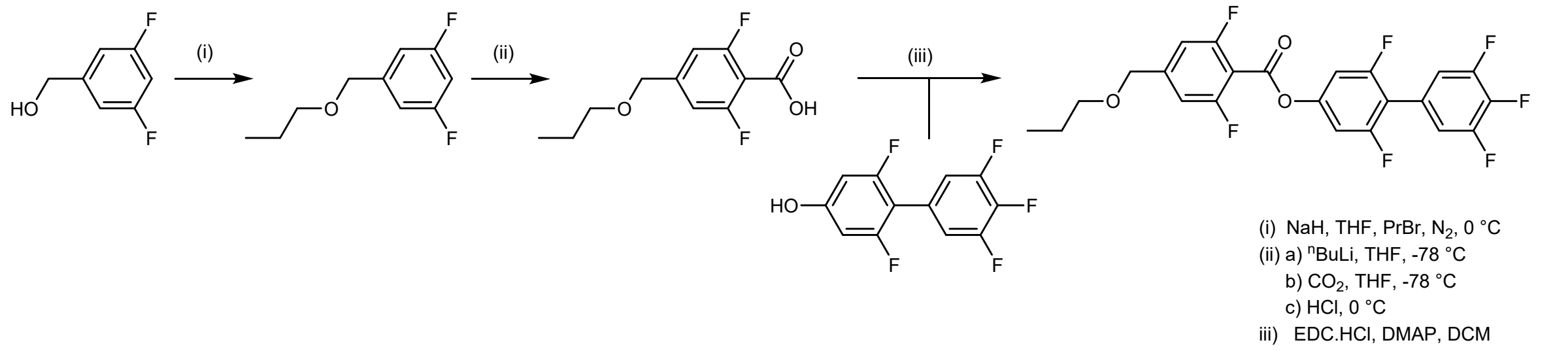


## Scheme 1

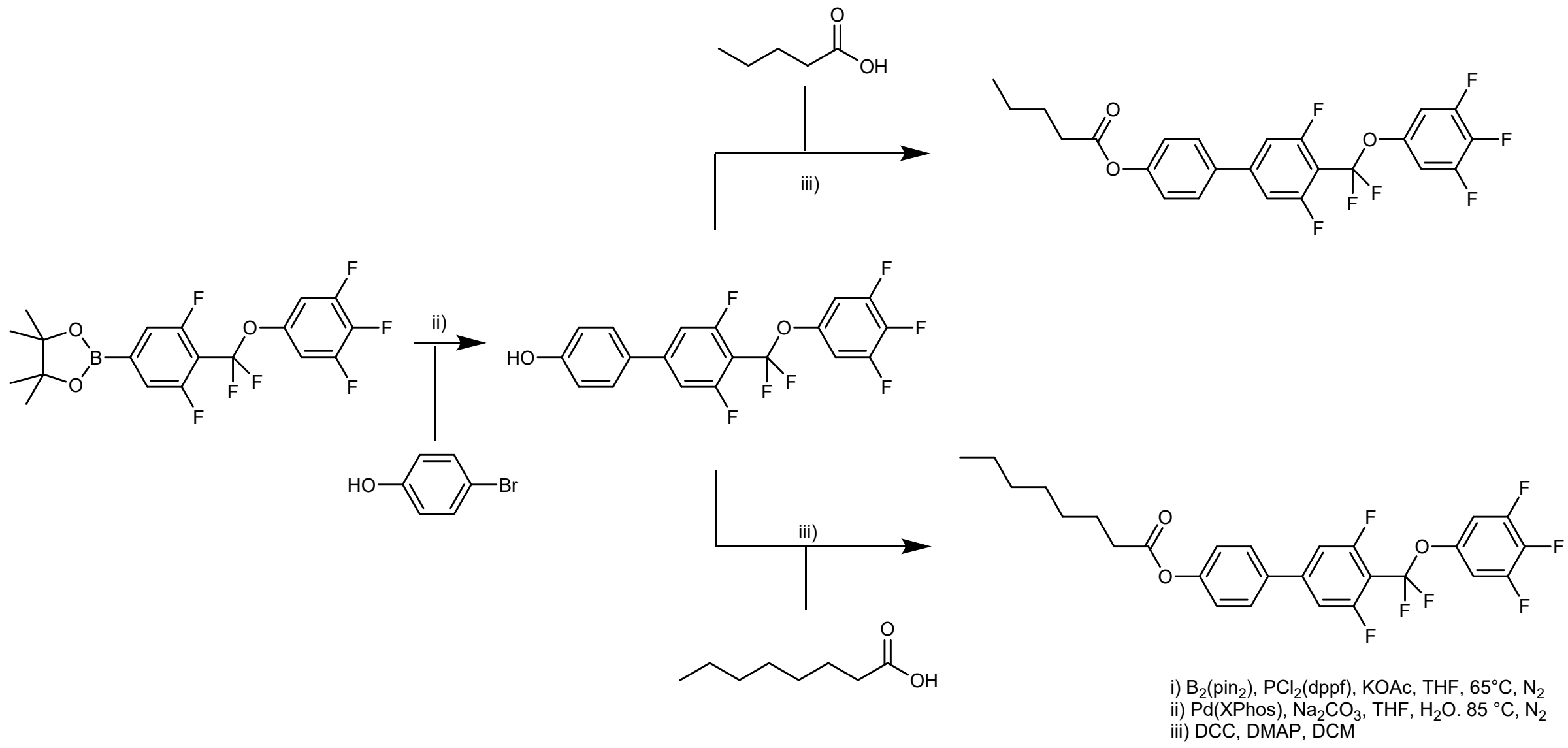


## Scheme 2

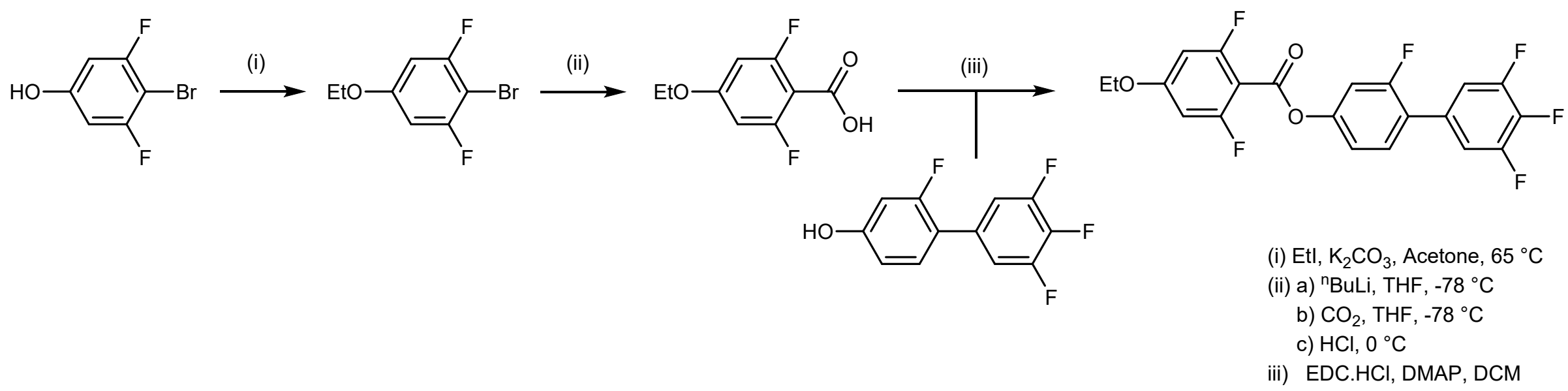


## Scheme 3

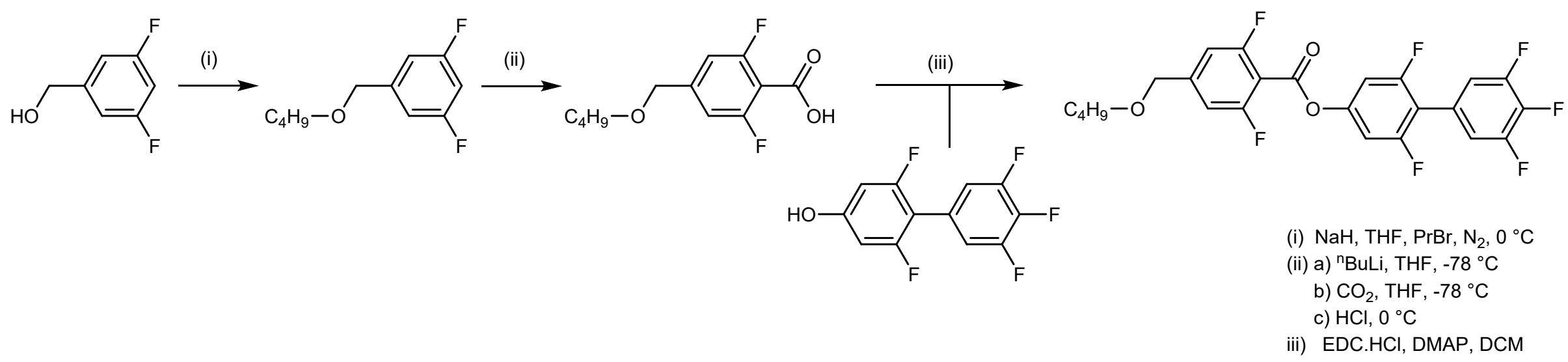


**Scheme 4**

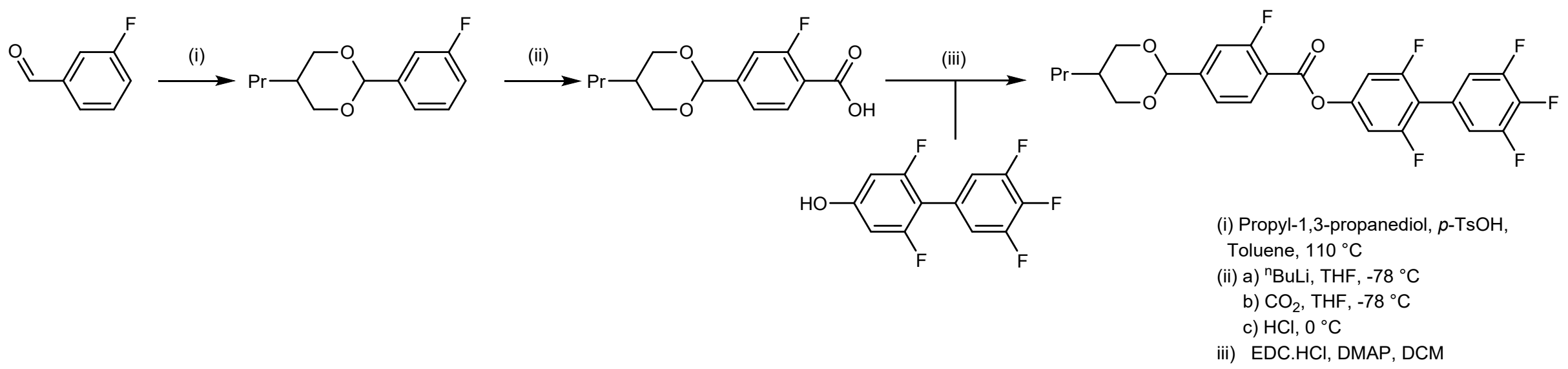


**Scheme 5**

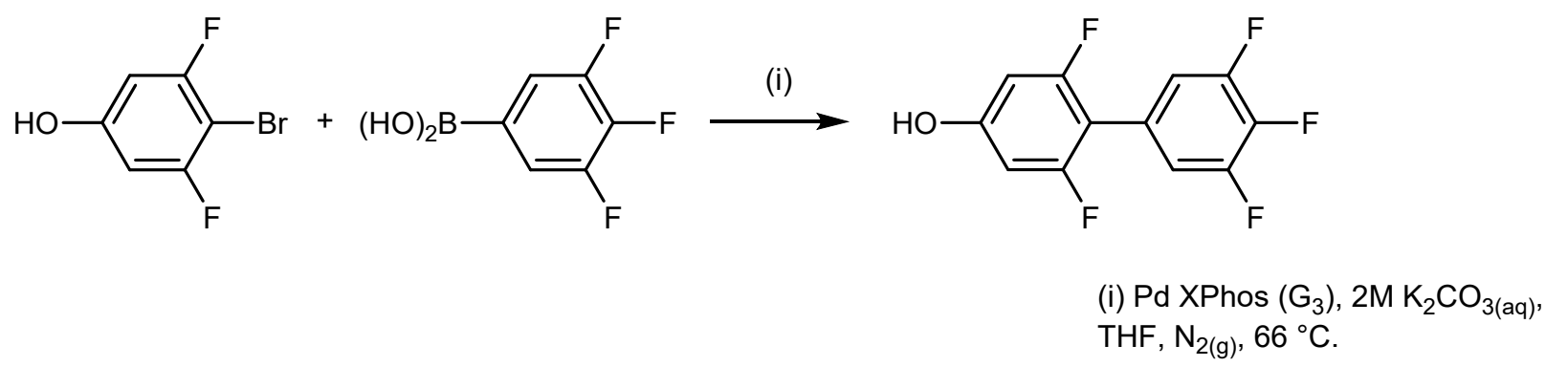


**Scheme 6**

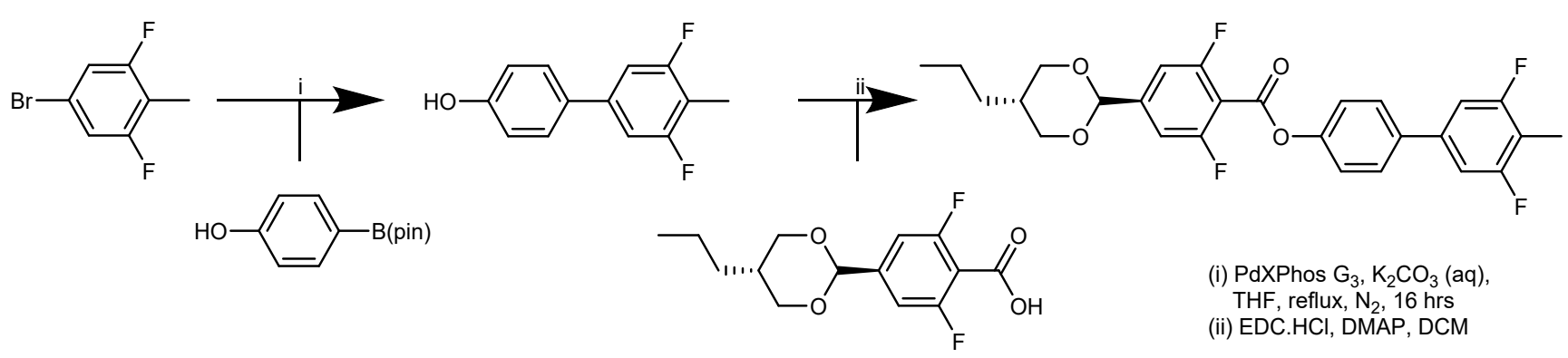


**Scheme 7**

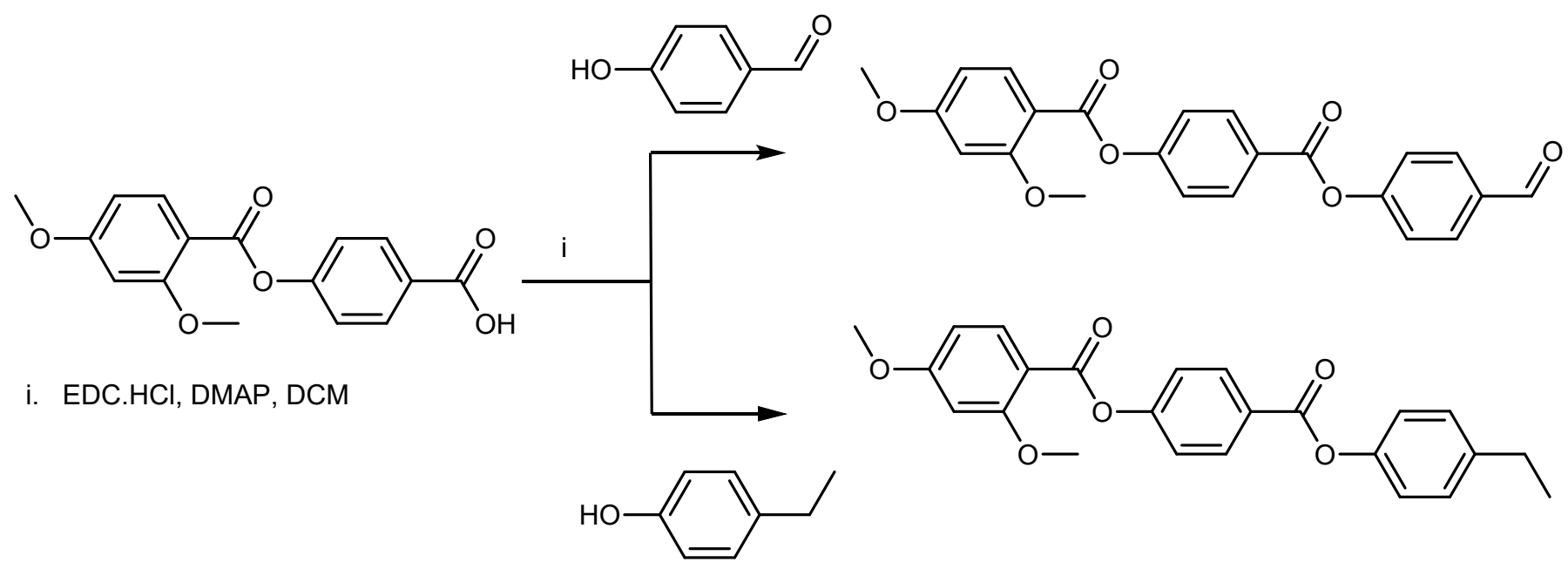


**Scheme 8**

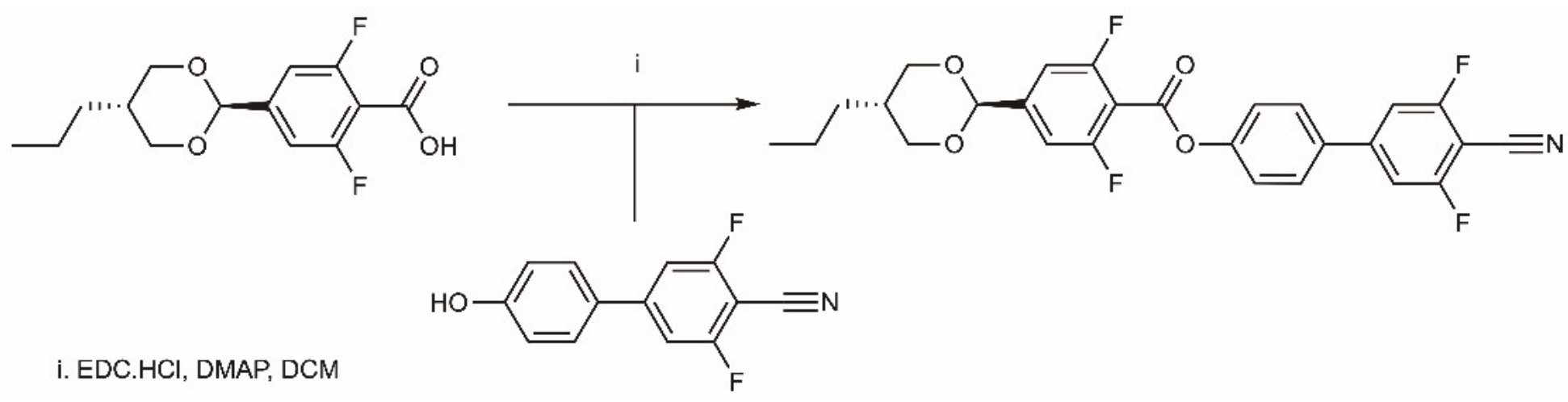


**Scheme 9**

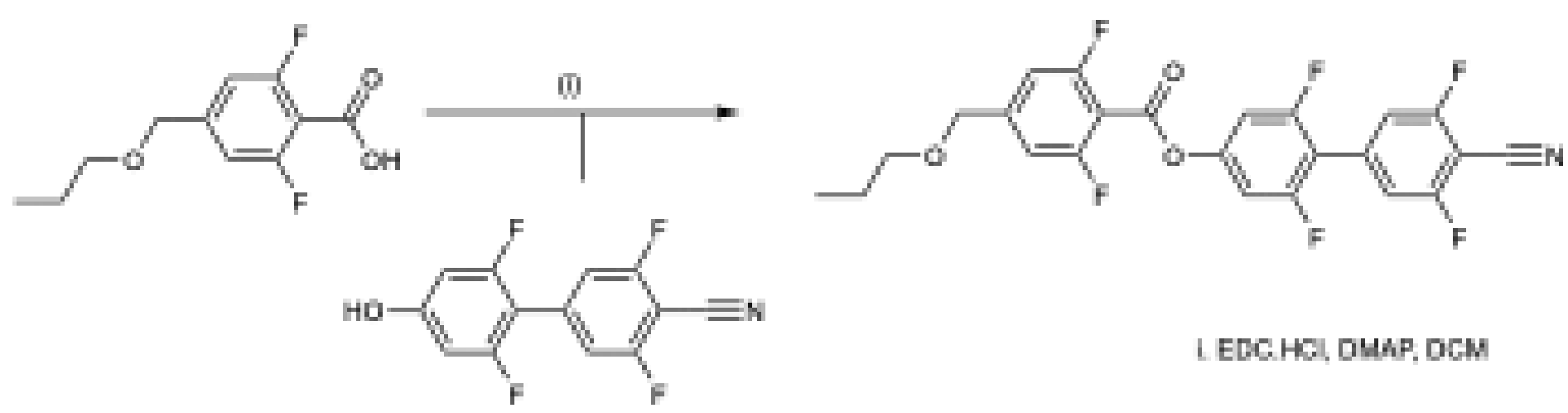


**Scheme 10**

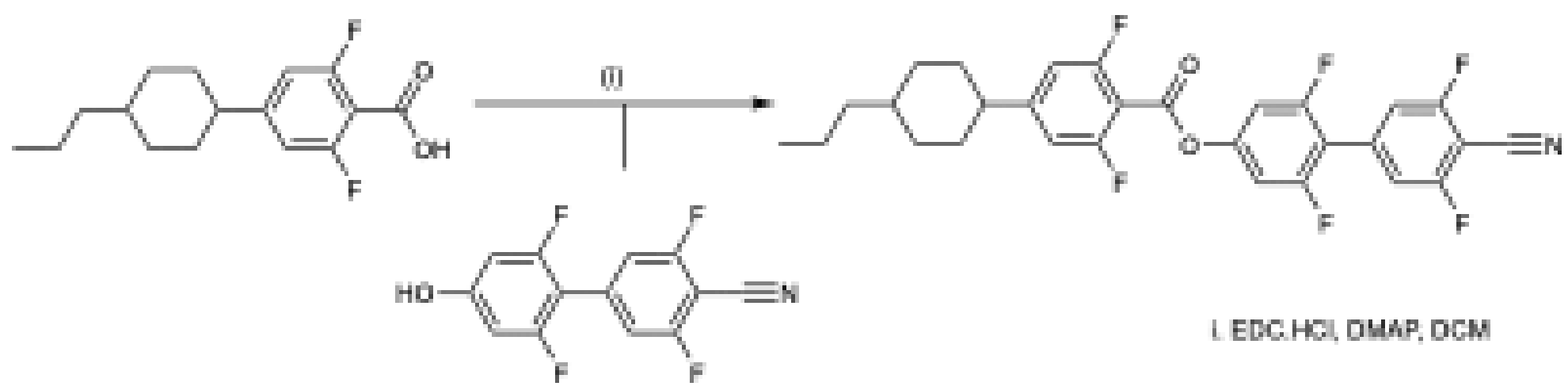


**Scheme 11**

## 9. Chemical Characterisation

### 9.1. Scheme 1

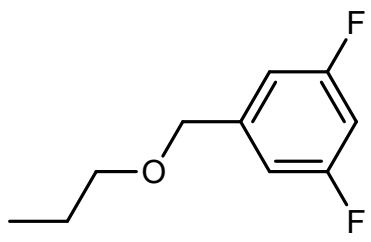


**IDX7a / WJ-2-41 / 3,5-difluorobenzyl propyl ether**

An oven dried flask was cooled under an atmosphere of dry nitrogen, and subsequently charged with THF (280 ml) and sodium hydride (1.72 g, 43 mmol, 1.5 mol eqv.). The suspension was stirred with cooling *via* an ice bath. 3,5-Difluorobenzyl alcohol (4.13 g, 28.7 mmol, 1 mol eqv.) was added dropwise, and the reaction stirred for one hour. 1-Bromopropane (24.4 g, 200 mmol, 7 mol eqv) was added dropwise. The reaction was monitored by TLC until completion. Once complete, the reaction was quenched by slow addition of water. The reaction was diluted with ethyl acetate, the organic layer was extracted and set aside. The aqueous layer was washed with ethyl acetate and discarded. The combined organics were dried over $MgSO_4$ and concentrated *in vacuo*. Chromatography of the crude material with a gradient of DCM/hexanes afforded the title compound as a colourless liquid.

| | |
|---|---|
| Yield: | 3.14 g, 59% |
| $R_F$: | 0.7 (DCM: hexane=1:1) |
| $^1$H NMR (400 MHz): | 6.87(m, 2H,Ar-**H**), 6.70 (tt, *J* = 9.0 Hz, *J* = 2.4 Hz, 1H,Ar-**H**), 4.47 (s, 2H,O-$\mathbf{CH_2}$-Ar), 3.45 (t, *J* = 6.7 Hz, 2H,O-$\mathbf{CH_2}$-$CH_2$-$CH_3$), 1.65 (m, 2H,O-$CH_2$-$\mathbf{CH_2}$-$CH_3$), 0.96 (t, *J* = 7.4 Hz, 3H,O-$CH_2$-$CH_2$-$\mathbf{CH_3}$). |
| $^{13}$C{$^1$H} NMR (101 MHz): | 163.23 (dd, *J* = 248.3, *J* = 12.6 Hz), 143.16 (t, *J* = 8.8 Hz), 109.89 (dd, J=6.7,18.6 Hz), 102.76 (t, *J* = 25.2 Hz), 72.66, 71.68, 23.06, 10.72. |
| $^{19}$F NMR (376 MHz): | -110.15 (t, *J* = 8.0 Hz Ar-F) |

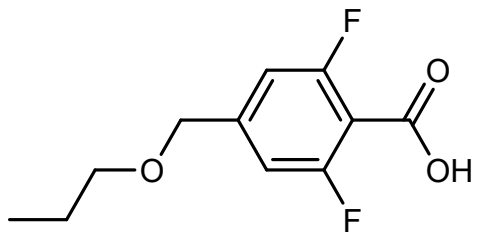


**IDX7b / WJ-2-52 / 2,6-difluoro-2-(propan-1-yloxy)benzoic acid**

Quantities used: 3,5-difluorobenzyl propyl ether (1.65 g, 8.9 mmol, 1 mol eqv), anhydrous THF (100 ml), n-butyl lithium (1.6 M in hexane, 10.6 mmol, 1.2 mol eqv.). General procedure B was followed. The resulting material was used without further purification.

| | |
|---|---|
| Yield: | 1.92 g, 94 % (pale yellow solid) |
| $R_F$: | 0.0 (DCM) |
| $^1$H NMR (400 MHz): | 11.45 (s, 1H,-COO**H**), 6.94 (m, 2H,Ar-**H**), 4.51 (s, 2H, O-C**H**$_2$-Ar), 3.46 (t, *J* = 6.6 Hz, 2H,CH$_3$-CH$_2$-C**H**$_2$-O), 1.64 (m, 2H,CH$_3$-C**H**$_2$-CH$_2$-O), 0.92 (t, *J* = 7.4 Hz, 3H,C**H**$_3$-CH$_2$-CH$_2$-O). |
| $^{13}$C{$^1$H} NMR (101 MHz): | 166.20, 161.40 (dd, *J* = 259.2, *J* = 5.9 Hz), 146.74 (t, *J* = 9.7 Hz), 110.39 (dd, *J* = 23.5, *J* =3.0 Hz), 108.37 (t, *J* = 16.4 Hz), 72.79, 70.87, 22.74, 10.42. |
| $^{19}$F NMR (376 MHz): | -108.26 (d, *J* = 9.9 Hz). |

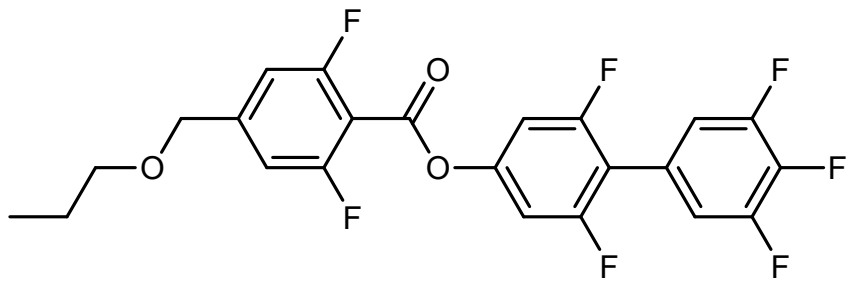


**IDX7/ WJ-2-54 / (2,3',4',5',6-pentafluoro-[1,1'-biphenyl]-4-yl)methyl 2,6-difluoro-4-(propoxymethyl)benzoate**

Quantities used: 2,6-difluoro-2-(propan-1-yloxy)benzoic acid (141 mg, 0.614 mmol, 1.2 mol eqv.), 4-(3,4,5-trifluorophenyl)-3,5-difluorophenol (133 mg, 0.511 mmol, 1 mol eqv.), EDC.HCl (147 mg, 0.77 mmol, 1.5 mol eqv.), DMAP (18.7 mg, 0.153 mmol, 30 mol%), DCM (5 ml). The reaction was performed according to general procedure A. The crude material was purified by flash chromatography over silica gel with a gradient of hexane/DCM, followed by recrystallization from n-hexane.

| | |
|---|---|
| Yield: | 120 mg (50%) |
| $R_F$: | 0.8 (DCM/hexane, 1:1) |
| $^1$H NMR (400 MHz): | 7.12 (m, *J* = 7.6 Hz, 2H,Ar-**H**), 7.02 (m, 4H,Ar-**H**), 4.55 (s, 2H, O-C**H**$_2$-Ar), 3.49 (t, *J* = 6.6 Hz, 2H,CH$_3$-CH$_2$-C**H**$_2$-O-), 1.68 (m, 2H,CH$_3$-C**H**$_2$-CH$_2$-O-), 0.98 (t, *J* = 7.4 Hz, 3H,C**H**$_3$-CH$_2$-CH$_2$-O-). |
| $^{13}$C{$^1$H} NMR (101 MHz): | 162.70 (dd, *J* = 259.5, *J* = 6.4 Hz),159.06, 158.75 (dd, *J* = 250.4, *J* = 8.1 Hz), 151.18 (ddd, *J* = 250.1, *J* = 10.3, *J* = 4.3 Hz), 150.92 (t, *J* = 14.4 Hz), 147.96 (t, *J* = 9.7 Hz), 139.91 (dt, *J* = 253.5, *J* = 14.8 Hz), 124.36 (td, *J* = 8.6, *J* = 5.7 Hz), 115.27 – 114.55 (m), 113.95 (t, *J* = 18.1 Hz), 110.49 (dd, *J* = 22.8, 3.3 Hz), 107.74 (t, *J* = 16.5 Hz), 106.64 (m), 73.01, 71.06, 23.01, 10.67. |
| $^{19}$F NMR (471 MHz): | -108.36 (d, *J* = 9.8 Hz), -112.20 (d, *J* = 8.3 Hz), -134.31 (dd, *J* = 20.7, 8.1 Hz), -159.93 (tt, *J* = 20.7, *J* = 6.5 Hz). |
| HRMS (APCI+): | 473.0979 (calcd. For $C_{23}H_{16}F_7O_3$: 473.0982, err: 0.6 ppm; M+H) |
| Assay (HPLC, C18): | 99.39% (254 nm , 5.176 min) |

## 9.2. Scheme Two

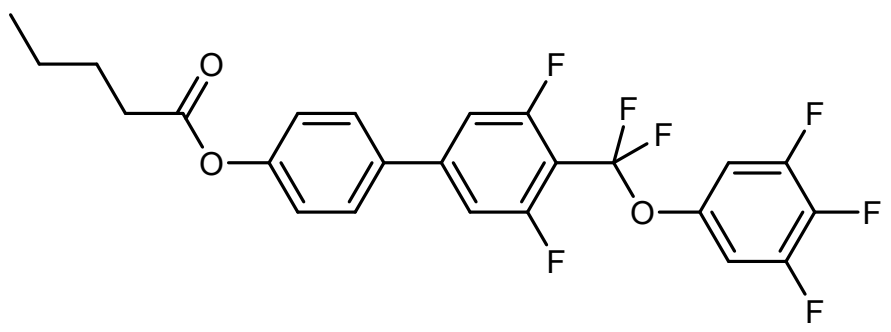

**IDX2538 / SRB-CHEM-082 / 4-hydroxy-(3',5'-difluoro-4'-(difluoro(3,4,5-trifluorophenoxy)methyl)-1,1'-biphenyl)yl pentanoate**

Quantities used: 4-Hydroxy-(3',5'-difluoro-4'-(difluoro(3,4,5-trifluorophenoxy)methyl)-1,1'-biphenyl) (0.402 g, 1 mmol), [3]valeric acid (0.122 g, 1.2 mmol), DCC (0.25 g, 1.2 mmol) and DMAP (12 mg, 0.1 mmol), DCM (10 ml). General procedure A was followed, with the exception that DCC was used in place of EDC.HCl. The crude material was purified via flash column chromatography over silica, using a gradient of hexane to dichloromethane as eluent. The resulting solid was then recrystallized from 2-propanol, to yield the product as colourless crystals.

| | |
|---|---|
| Yield: | 0.36 g, (73 %) |
| $R_F$: | 0.59 (DCM/Hexane, 1:1) |
| $^1$H NMR (400 MHz): | 7.59 (dt, *J* = 8.6, *J* = 2.8 Hz, 2H, Ar-**H**), 7.28 – 7.17 (m, 4H, Ar-**H**)*, 7.07 – 6.95 (m, 2H, Ar-**H**)*, 2.63 (t, *J* = 7.5 Hz, 2H, R-C**H**$_2$-COOAr), 1.79 (m, 2H, R-C**H**$_2$-CH$_2$-CH$_3$), 1.50 (m, *J* = 7.6 Hz, 2H, R-CH$_2$-C**H**$_2$-CH$_3$), 1.02 (t, *J* = 7.4 Hz, 3H, CH$_2$-C**H**$_3$). (*Overlapping Peaks) |
| $^{13}$C{$^1$H} NMR (101 MHz): | 172.13, 160.28 (dd, *J* = 257.5, *J* = 10.1 Hz), 151.73, 151.02 (ddd, *J* = 251.2, *J* = 10.5, *J* = 5.1 Hz), 146.12 (t, *J* = 10.5 Hz), 144.92 – 144.41 (m), 138.24 (dt, *J* = 251.3, *J* = 15.5 Hz), 128.09, 122.50, 120.26 (t, *J* = 265.4 Hz), 117.61, 110.97 (dd, *J* = 24.1, *J* = 3.0 Hz), 108.82 – 108.01 (m), 107.45 (dd, *J* = 16.2, *J* = 6.5 Hz), 34.11, 26.96, 22.26, 13.72. |
| $^{19}$F NMR (471 MHz): | -61.66 (t, *J* = 26.2 Hz, 2F, O-C**F**$_2$-Ar), -110.17 (td, *J* = 26.2, *J* = 11.1 Hz, 2F, Ar-**F**), -132.48 (dd, *J* = 20.8, *J* = 8.6 Hz, 2F, Ar-**F**), -163.17 (tt, *J* = 21.0, *J* = 6.0 Hz, 1F, Ar-**F**). |
| HRMS (ESI+): | 487.1142 ([M+H]$^+$): calcd. For $C_{24}H_{18}F_7 0_3$: 487.1139, err = -0.6 ppm) |
| Assay (HPLC, C18): | 99.78% (254 nm, 5.774 min) |
| m.p.: | 63.1 °C |

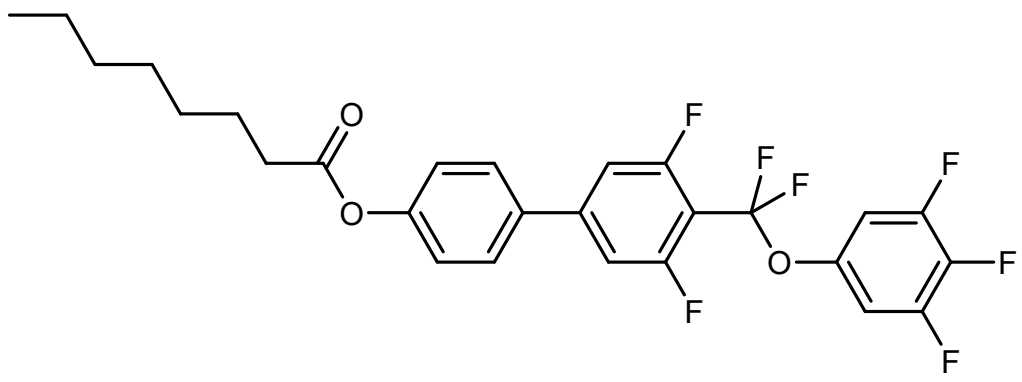

**IDX1646 / SRB-CHEM-085 / 4-hydroxy-(3’,5’-difluoro-4’-(difluoro(3,4,5-trifluorophenoxy)methyl)-1,1’-biphenyl)yl octanoate**

Quantities used: 4-Hydroxy-(3’,5’-difluoro-4’-(difluoro(3,4,5-trifluorophenoxy)methyl)-1,1’-biphenyl) (0.402 g, 1 mmol), ), [3] octanoic acid (0.173 g, 1.2 mmol), DCC (0.25 g, 1.2 mmol), DMAP (12 mg, 0.1 mmol), DCM (10 ml). General procedure A was followed, with the exception that DCC was used in place of EDC.HCl. The crude material was purified via flash column chromatography over silica, using a gradient of hexane to dichloromethane as eluent. The resulting solid was then recrystallized from 2-propanol, to yield the product as colourless crystals.

| | |
|---|---|
| Yield: | 0.30 g, (56 %) |
| $R_F$: | 0.65 (DCM/Hexane, 1:1) |
| $^1$H NMR (400 MHz): | 7.50 (dt, *J* = 8.7, *J* = 2.8 Hz, 2H, Ar-**H**), 7.17 – 7.08 (m, 4H, Ar-**H**)*, 6.96 – 6.87 (m, 2H, Ar-**H**)*, 2.52 (t, *J* = 7.5 Hz, 2H, RC**H**$_2$-COOAr), 1.70 (m, , 2H, R-C**H**$_2$-R), 1.42 – 1.17 (m, 8H, R-C**H**$_2$-R)*, 0.83 (t, *J* = 6.8 Hz, 3H, R-C**H**$_3$). (*Overlapping Peaks) |
| $^{13}$C{$^1$H} NMR (101 MHz): | 172.14, 160.26 (dd, *J* = 257.7, *J* = 5.2 Hz), 151.74, 151.03 (ddd, *J* = 254.8, *J* = 10.3, *J* = 5.2 Hz), 146.12 (t, *J* = 10.6 Hz), 144.91 – 144.39 (m), 138.46 (dt, *J* = 250.5, 14.7 Hz), 134.96, 128.10, 122.50, 120.26 (t, *J* = 265.2 Hz), 117.62, 110.98 (dd, *J* = 24.1, *J* = 3.0 Hz), 108.71-108.0 (m), 107.45 (dd, *J* = 16.3, 6.4 Hz), 34.40, 31.67, 29.08, 28.93, 24.91, 22.61, 14.07. |
| $^{19}$F NMR (471 MHz): | -61.66 (t, *J* = 26.2 Hz, 2F, O-C**F**$_2$-Ar), -110.16 (td, *J* = 26.2, *J* = 11.2 Hz, 2F, Ar-**F**), -132.47 (dd, *J* = 20.6, *J* = 8.7 Hz, 2F, Ar-**F**), -163.16 (tt, *J* = 20.9, 6.2 Hz, 1F, Ar-**F**). |
| HRMS (ESI+): | 529.1615 ($[M+H]^+$): calcd. For $C_{27}H_{24}F_7O_3$: 529.1608, err = -1.3 ppm) |
| Assay (HPLC, C18): | 99.16% (254 nm, 6.291 min) |
| m.p.: | 64.8 °C |

## 9.3. Scheme Three

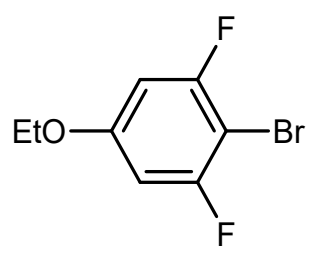


**IDX323 / CJG 412 / 2-bromo-5-ethoxy-1,3-diflurobenzene**

4 bromo-3,5-diflurophenol (4.16 g 20 mmol, 1 mol eqv.) was dissolved in 100 mL of acetone. Potassium carbonate (4.14 g, 30 mmol, 1.5 mol eqv.) was added, and the reaction stirred for 30 mins before iodo ethane (3.43 g, 1.77 mL, 22 mmol, 1.1 mol eqv.) was added in one portion and the reaction was heated under reflux until complete - as judged by the consumption of the phenolic reagent by TLC (circa 24 h). The reaction was then filtered, the filtrate washed with acetone, the volatiles removed *in vacuo*, before being purification by flash chromatography using a gradient of DCM:hexanes. The chromatographed product was then recrystallised from EtOH to afford the title compound as a white solid.

| | |
|---|---|
| Yield: (White solid) | 3.98 g, 84 % |
| $R_F$ (Hexanes): | 0.10 |
| $^1$H NMR (400 MHz): | 6.48 (ddd, *J* = 13.2 Hz, *J* = 10.0 Hz, *J* = 8.2 Hz, 2H, Ar-**H**), 3.96 (d, *J* = 7.0 Hz, 2H, O-C$\mathbf{H_2}$-$CH_3$), 1.40 (t, *J* = 7.0 Hz, 3H, $CH_2$-C$\mathbf{H_3}$). |
| $^{13}$C{$^1$H} NMR (101 MHz): | 160.24 (dd, *J* = 246.5 Hz, *J* = 7.1 Hz), 159.60 (t, *J* = 12.9 Hz), 99.08 (dd, *J* = 26.4 Hz, *J* = 2.9 Hz), 88.05 (t, *J* = 25.0 Hz), 64.24, 14.39. |
| $^{19}$F NMR (376 MHz): | -105.23 (d, *J* = 9.5 Hz, 2F. Ar-**F**). |

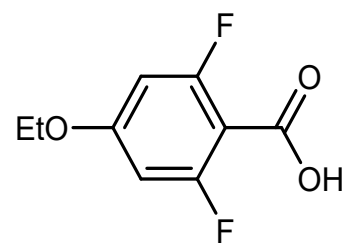

**IDX323 / CJG 483 / 4-ethoxy-2,6-diflurobenzoic acid**

Quantities used: 2-bromo-5-ethoxy-1,3-diflurobenzene (3.2 g, 13.5 mmol, 1 mol eqv.), anhydrous THF (100 mL), n-butyl lithium (1.6 M in hexane, 12.65 mL, 20.25 mmol, 1.5 mol eqv.) General procedure B was followed. The crude material was re-crystallised from EtOH to afford the title compound as a crystalline white solid.

| | |
|---|---|
| Yield: | 2.21 g, 81 % |
| $R_F$ (DCM): | 0.00 |
| $^1$H NMR (400 MHz, DMSO): | 13.40 (s, 1H, Ar-COO**H**), 7.00 – 6.68 (m, 2H, Ar-**H**), 4.10 (q, *J* = 7.0 Hz, 2H, O-C**H$_2$**-CH$_3$), 1.33 (t, *J* = 7.0 Hz, 3H, CH$_2$-C**H$_3$**). |
| $^{13}$C{$^1$H} NMR (101 MHz, DMSO): | 162.64 – 162.19 (m)*, 161.63 (dd, *J* = 251.8, *J* = 10.3 Hz), 104.10 (t, *J* = 18.4 Hz), 100.36 – 98.98 (m), 65.08, 14.67. *Overlapping signals. |
| $^{19}$F NMR (376 MHz, DMSO): | -109.46 (d, *J* = 11.7 Hz, 2F, Ar-**F**). |

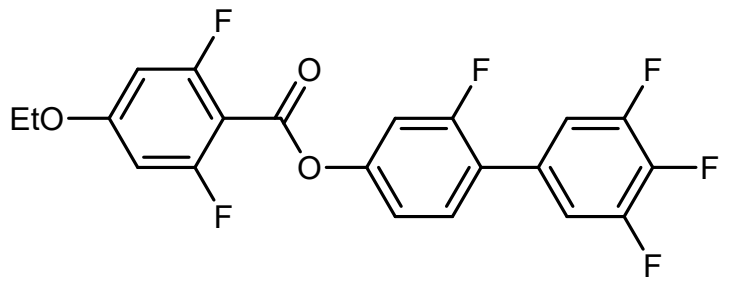


**IDX 232 / CJG 484 / 2,3',4',5'-tetrafluoro-[1,1'-biphenyl]-4-yl 4-ethoxy-2,6-difluorobenzoate:** Quantities used: 4-ethoxy-2,6-diflurobenzoic acid (130 mg, 0.6 mmol, 1.2 mol eqv.), 4-(3,4,5-trifluorophenyl)-3-fluorophenol (121 mg, 0.50 mmol, 1 mol eqv.), EDC.HCl (143 mg, 0.75 mmol, 1.5 mol eqv.), DMAP (5 mg, 0.05 mmol, 30 mol%), DCM (5 ml). The reaction was performed according to general procedure A. The crude material was purified by flash chromatography over silica gel with a gradient of hexane/DCM, followed by recrystallization from EtOH to afford a white solid.

| | |
|---|---|
| Yield: | 307 mg, 72 % |
| $R_F$ (DCM): | 0.90 |
| $^1$H NMR (400 MHz): | 7.42 (t, *J* = 8.7 Hz, 1H, Ar-**H**), 7.21 – 7.10 (m, 4H, Ar-**H**)*, 6.54 (d, *J* = 10.5 Hz, 2H, Ar-**H**), 4.09 (q, *J* = 7.0 Hz, 2H, O-C**H$_2$**-CH$_3$), 1.46 (t, *J* = 7.0 Hz, 3H, CH$_2$-C**H$_3$**). *Overlapping signals. |
| $^{13}$C{$^1$H} NMR (101 MHz): | 164.05 – 163.55 (m)*, 163.00 (dd, *J* = 258.3 Hz, 8.4 Hz), 159.42 (t, *J* = 2.9 Hz), 159.36 (d, *J* = 251.3 Hz), 151.20 (ddd, *J* = 250.4 Hz, 10.3 Hz, 4.5 Hz), 151.18 (d, *J* = 10.9 Hz), 140.76, 138.24 (t, *J* = 15.3 Hz), 135.51 (t, *J* = 10.1 Hz), 131.03 – 130.82 (m), 130.56 (d, *J* = 4.0 Hz), 124.09 (d, *J* = 11.4 Hz), 118.26 (d, *J* = 3.8 Hz), 113.49 – 112.93 (m), 110.85, 110.59, 101.48 (t, *J* = 15.5 Hz), 99.28 (dd, *J* = 26.1, 3.1 Hz), 64.83, 14.39. *Overlapping signals. |
| $^{19}$F NMR (376 MHz): | -106.16 (d, *J* = 11.6 Hz, 2F, Ar-**F**), -114.60 (t, *J* = 9.7 Hz, 1F, Ar-**F**), -134.24 (dd, *J* = 20.6, 8.7 Hz, 2F, Ar-**F**), -161.29 (tt, *J* = 20.7, 6.6 Hz, 1F, Ar-**F**). |

## 6.4. Scheme 4

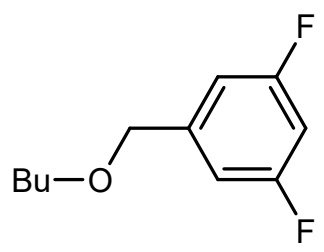


**CJG 411 / 3,5-difluorobenzyl butyl ether**

An oven-dried flask was cooled under an atmosphere of dry nitrogen, and subsequently charged with anhydrous THF (100 ml) and sodium hydride (60%, 3.00 g, 75 mmol, 1.5 mol eqv.). The suspension was stirred with cooling *via* an ice bath. 3,5-Difluorobenzyl alcohol (7.20 g, 50.0 mmol, 1 mol eqv.) was added dropwise, and the reaction was left to stir for one hour. 1-Bromobutane (8.2 g, 60 mmol, 1.1 mol eqv) was added dropwise. The reaction was monitored by TLC until completion. Once complete, the reaction was quenched by slow addition of water. The reaction was diluted with EtOAc, and the organic layer was extracted 2x 20 mL EtOAc. The combined organics were dried over $MgSO_4$ and concentrated *in vacuo*. Chromatography of the crude material with a gradient of DCM/hexanes afforded the title compound as a colourless oil.

| | |
|---|---|
| Yield: | 6.02 g, 62 % |
| $R_F$ (DCM): | 0.40 |
| $^1$H NMR (400 MHz): | 6.93 – 6.84 (m, 2H, Ar-**H**), 6.71 (tt, *J* = 9.0 Hz, *J* = 2.5 Hz, 1H, Ar-**H**), 4.48 (s, 2H, Ar-C**H₂**-O), 3.51 (t, *J* = 6.5 Hz, 2H, O-C**H₂**-CH₂), 1.64 (m, 2H, CH₂-C**H₂**-CH₂), 1.45 (m, 2H, CH₂-CH₂-C**H₃**), 0.96 (t, *J* = 7.4 Hz, 3H, CH₂-C**H₃**). |
| $^{13}$C{$^1$H} NMR (101 MHz): | 163.10 (dd, *J* = 248.3 Hz, *J* = 12.6 Hz), 143.15 (t, *J* = 8.8 Hz), 109.95 – 109.42 (m), 102.52 (t, *J* = 25.4 Hz), 71.52, 70.61, 31.78, 19.35, 13.81. |
| $^{19}$F NMR (376 MHz): | -110.17 (t, *J* = 8.0 Hz, F, Ar-**F**). |

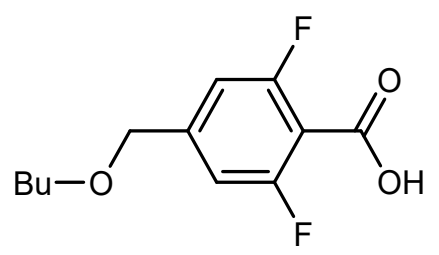

**CJG 448 / 2,6-difluoro-2-(butyl-1-yloxy)benzoic acid**

Quantities used: 3,5-Difluorobenzyl butyl ether (1.30 g, 6 mmol, 1 mol eqv), anhydrous THF (50 mL) n-butyl lithium (1.6 M in hexane, 5.63 mL 9 mmol, 1.5 mol eqv.) was added dropwise. General procedure B was followed. The resulting material was used without further purification.

| | |
|---|---|
| Yield: | 1.30 g, 89 % |
| $R_F$ (DCM): | 0.00 |
| $^1$H NMR (400 MHz) | 7.12 (d, *J* = 8.9 Hz, 2H, Ar-**H**), 4.50 (s, 2H, Ar-C$\mathbf{H_2}$-O), 3.45 (t, *J* = 6.5 Hz, 2H, O-C$\mathbf{H_2}$-$CH_2$), 1.54 (m, 2H, $CH_2$-C$\mathbf{H_2}$-$CH_2$), 1.35 (m, 2H, $CH_2$-C$\mathbf{H_2}$-$CH_3$), 0.89 (t, *J* = 7.4 Hz, 3H, $CH_2$-C$\mathbf{H_3}$). |
| $^{19}$F NMR (376 MHz): | -111.97 (d, *J* = 9.7 Hz, 2F, Ar-**F**). |

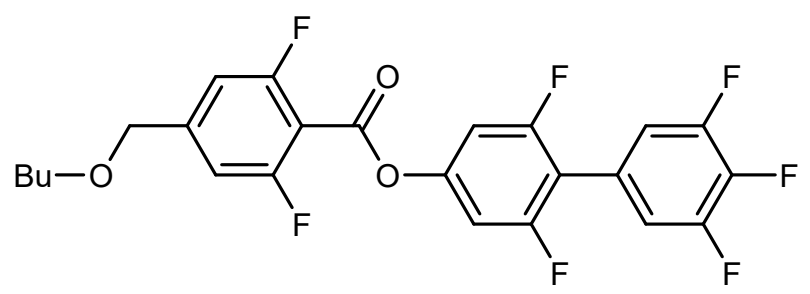

**IDX 59 / CJG 480 / 2,3',4',5',6-pentafluoro-[1,1'-biphenyl]-4-yl 4-(butoxymethyl)-2,6-difluorobenzoate**

Quantities used: 2,6-difluoro-2-(Butyl-1-yloxy)benzoic acid (383 mg, 1.65 mmol, 1.2 mol eqv.), 4-(3,4,5-trifluorophenyl)-3,5-difluorophenol (390 mg, 1.50 mmol, 1 mol eqv.), EDC.HCl (501 mg, 2.63 mmol, 1.5 mol eqv.), DMAP (18 mg, 0.15 mmol, 30 mol%), DCM (5 ml). The reaction was performed according to general procedure A. The crude material was purified by flash chromatography over silica gel with a gradient of hexane/DCM, followed by recrystallization from MeCN to give a white solid.

| | |
|---|---|
| Yield: | 510 mg, 70 % |
| $R_F$ (DCM): | 0.85 |
| $^1$H NMR (400 MHz) | 7.17 – 7.08 (m, 2H, Ar-**H**), 7.07 – 6.98 (m, 4H, Ar-**H**)*, 4.54 (s, 2H, Ar-C**H$_2$**-O), 3.53 (t, *J* = 6.5 Hz, 2H, O-C**H$_2$**-CH$_2$), 1.64 (p, *J* = 6.7 Hz, 2H, CH$_2$-C**H$_2$**-CH$_2$), 1.44 (h, *J* = 7.5 Hz, 2H, CH$_2$-C**H$_2$**-CH$_3$), 0.96 (t, *J* = 7.4 Hz, 3H, CH$_2$-C**H$_3$**).*Overlapping Signals |
| $^{13}$C{$^1$H} NMR (101 MHz) | 161.29 (dd, *J* = 259.5 Hz, *J* = 5.8 Hz), 159.98, 159.58 (dd, *J* = 250.4 Hz, *J* = 8.5 Hz), 151.25 (ddd, *J* = 250.2 Hz, *J* = 10.2 Hz, *J* = 4.0 Hz), 150.77 (t, *J* = 14.3 Hz), 147.80 (t, *J* = 9.7 Hz), 139.44 (dt, *J* = 254.5 Hz, *J* = 14.6 Hz), 124.44 – 124.01 (m), 113.87 (t, *J* = 17.5 Hz), 110.39 (dd, *J* = 22.7 Hz, 3.2 Hz), 107.65 (t, *J* = 16.5 Hz), 106.90 – 106.24 (m), 71.06, 71.01, 31.73, 19.35, 13.89. |
| $^{19}$F NMR (376 MHz) | -108.37 (d, *J* = 10.2 Hz, 2F, Ar-**F**), -112.22 (d, *J* = 8.8 Hz, 2F, Ar-**F**), -134.33 (dd, *J* = 20.6, *J* = 8.3 Hz, 2F, Ar-**F**), -159.96 (tt, *J* = 20.9, *J* = 6.5 Hz, 1F, Ar-**F**). |

## 6.5. Scheme 5

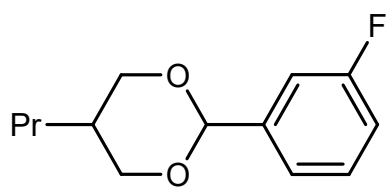


**CJG 348 / 2-(3-fluorophenyl)-5-propyl-1,3-dioxane**

An oven dried flask, fitted with a dean-stark apparatus, was cooled under an atmosphere of dry nitrogen and charged with 3-fluorobenzaldehyde (13.87 g, 110 mmol, 1.1 mol eqv.), propyl-1,3 propanediol (12 g, 100 mmol, 1 mol eqv.), para-toluenesulfonic acid (3.8 g 20 mmol, 0.2 mol eqv.) and 150 mL of anhydrous toluene. The reaction mixture was heated to reflux, with any evolved water being removed at regular intervals, until no further water was captured within the dean-stark apparatus (circa 48 h). The reaction was cooled to room temperature and concentred *in vacuo* before being purified by flash chromatography using a gradient of EtOAc:hexanes. The chromatographed material was then recrystallised from MeOH to afford the title compound as a white solid.

Yield: 12.1 g, 54 %

Spectral data was in keeping with literature values.**[2]**

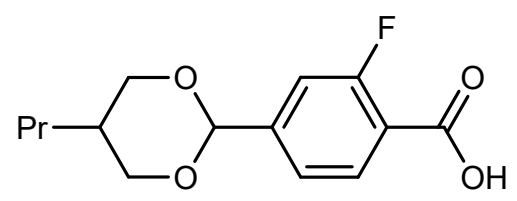


**CJG 358 / 2-fluoro-4-(5-propyl-1,3-dioxan-2-yl)benzoic acid**

Quantitues used: 2-(3-fluorophenyl)-5-propyl-1,3-dioxane (12.0 g, 54 mmol, 1 mol eqv), anhydrous THF (50 mL), n-butyl lithium (1.6 M in hexane, 41.0 mL 65 mmol, 1.5 mol eqv.). General procedure B was followed. The resulting material was purified by recrystallisation from hexane: toluene (5:1) to afford the title compound as an off white solid.

| | |
|---|---|
| Yield: | (Off-white solid) 13.3 g, 92 % |
| $R_F$ (DCM): | 0.00 |

Spectral data was in keeping with literature values. **[3]**

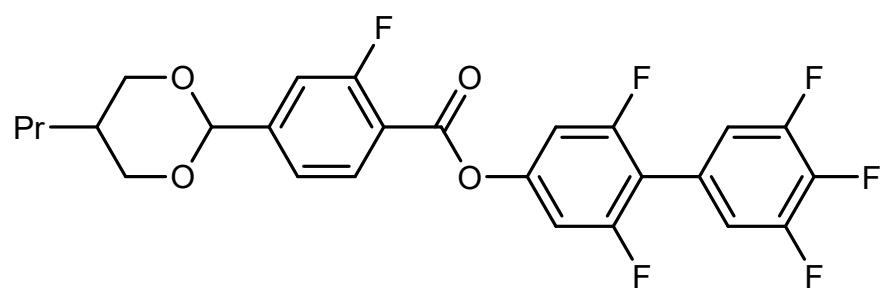

**IDX 519 / CJG 548 / 2,3',4',5',6-pentafluoro-[1,1'-biphenyl]-4-yl 2-fluoro-4-(5-propyl-1,3-dioxan-2-yl)benzoate**

Quantities used: 2-fluoro-4-(5-propyl-1,3-dioxan-2-yl)benzoic acid (161 mg, 0.6 mmol, 1.2 mol eqv.), 4-(3,4,5-trifluorophenyl)-3,5-difluorophenol (130 mg, 0.5 mmol, 1.0 mol eqv.), EDC.HCl (147 mg, 0.75 mmol, 1.5 mol eqv.), DMAP (6 mg, 0.05 mmol, 10 mol%), DCM (5 ml). The reaction was performed according to general procedure A. The crude material was purified by flash chromatography over silica gel with a gradient of hexane/DCM, followed by recrystallization from MeOH to give a white solid.

| | |
|---|---|
| Yield: | 132 mg, 80 % |
| $R_F$ (DCM): | 0.83 |
| $R_F$ (1:1 DCM/hexane): | 0.36 |
| $^1$H NMR (400 MHz): | 8.00 (1H, dd, *J* = 8.4, 7.1 Hz, Ar**H**), 7.37 – 7.29 (2H, m, Ar**H**), 7.06 (2H, ddt, *J* = 8.4, 7.4, 1.1 Hz, Ar**H**), 6.96 – 6.89 (2H, m, Ar**H**), 5.38 (1H, s, Ar-C**H**-$(OCH_2)_2$), 4.23 – 4.16 (2H, m, Ar-CH(OC**H**H)$_2$CH-$C_3H_9$), 3.49 (2H, ddd, *J* = 11.6, 10.4, 1.5 Hz, Ar-CH(OCH**H**)$_2$CH-$C_3H_9$)), 2.15 – 2.03 (1H, m, Ar-CH$(OCH_2)_2$C**H**-$C_3H_9$)), 1.34 – 1.23 (2H, m, Diox-C**H$_2$**-$CH_2$-$CH_3$), 1.08 – 1.00 (2H, m, Diox-$CH_2$-C**H$_2$**-$CH_3$), 0.87 (3H, t, *J* = 7.3 Hz, Diox-$CH_2$-$CH_2$-C**H$_3$**). |
| $^{13}$C{$^1$H} NMR (101 MHz): | 163.38, 161.66 (d, *J* = 4.8 Hz), 161.29, 159.72 (dd, *J* = 250.3, 9.1 Hz), 151.09 (t, *J* = 14.5 Hz), 151.07 (ddd, *J* = 249.6, 10.4, 4.5 Hz), 146.95 (d, *J* = 8.9 Hz), 139.79 (dt, *J* = 253.5, 15.4 Hz), 132.52, 124.28 (td, *J* = 9.8, 5.8 Hz), 122.05 (d, *J* = 4.2 Hz), 116.95 (d, *J* = 9.7 Hz), 115.34 (d, *J* = 23.9 Hz), 115.13 – 114.54 (m), 107.42 – 105.95 (m), 99.40 (d, *J* = 2.1 Hz), 72.65, 33.95, 30.28, 19.55, 14.21. |
| $^{19}$F NMR (376 MHz): | -107.23 - -107.23 (1F, m, Ar**F**), -112.44 (2F, d, *J* = 8.7 Hz, Ar**F**), -134.36 (2F, dd, *J* = 20.6, 8.4 Hz, Ar**F**), -160.02 (1F, tt, *J* = 20.7, 6.8 Hz, Ar**F**) |

## 6.6. Scheme 6

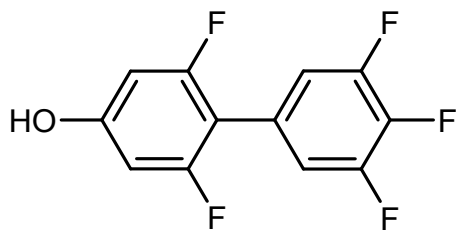

**CJG 290 / 4-(3,4,5-trifluorophenyl)-3,5-difluorophenol**

Quantities used: 4-Bromo-3,5-difluorophenol (10.45 g, 50.0 mmol), 3,4,5-trifluorobenzeneboronic acid (9.67 g, 55.0 mmol) THF (125 ml) 2M aqueous $K_2CO_3$ (50 mL), Pd-XPHOS-G3 (~ 1 mol%). General procedure C was followed. The crude material was purified by flash chromatography using a gradient of hexanes:DCM. The chromatographed material was then re-crystallised from hexanes: toluene (10:1) to afford the product as a white solid.

| | |
|---|---|
| Yield: | (White Solid) 11.96 g, 92 % |
| $R_F$ (DCM): | 0.60 |
| $^1$H NMR (400 MHz, DMSO): | 10.63 (s, 1H, Ar-O**H**), 7.36 – 7.23 (m, 2H, Ar-**H**), 6.59 – 6.51 (m, 2H, Ar-**H**). |
| $^{13}$C{$^1$H} NMR (101 MHz, DMSO): | 206.63, 160.19 (dd, *J* = 245.6 Hz, 10.1 Hz) 160.00 (t, *J* = 15.1 Hz), 150.48 (ddd, *J* = 247.2 Hz, 9.7 Hz, 4.2 Hz), 138.29 (dt, *J* = 250.2 Hz, 15.6 Hz), 126.44 – 125.91 (m), 115.27 (dd, *J* = 21.6 Hz, 4.6 Hz), 106.08 (t, *J* = 18.9 Hz), 100.62 (dd, *J* = 25.0, 2.8 Hz), 100.34 – 99.64 (m). |
| $^{19}$F NMR (376 MHz, DMSO): | -115.06 (d, *J* = 10.5 Hz, 2F, Ar-**F**), -135.84 (dd, *J* = 21.3, 9.5 Hz, 2F, Ar-**F**), -162.47 (tt, *J* = 21.6, 6.8 Hz, 1F, Ar-**F**). |

## 6.6. Scheme 7

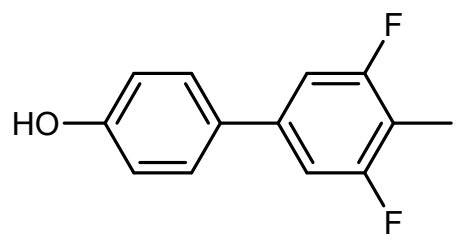


**RM3024 / 2,6-Difluoro-4-(4-hydroxyphenyl)toluene**

Quantities used: 4-Bromo-2,6-difluorotoluene (5.0 g, 24.4 mmol), 4-hydroxybenzene boronic acid pinacol ester (6.2 g, 28 mmol), THF (100 ml), 2M aqueous sodium carbonate (50 ml), Pd-XPHOS-G3 (5 mg). General Procedure C was followed. The crude material was purified by flash chromatography with a gradient of ethyl acetate/hexane to give the title compound as an off white solid.

| | |
|---|---|
| Yield: | 4.3 g, 80.1% |
| $R_F$ (Hexanes): | 0.65 |
| $^1$H NMR (400 MHz): | 9.67 (s, 1H, ArO**H**), 7.53 (ddd, 2H, *J* = 8.9 Hz, *J* = 3.1 Hz, *J* = 2.4 Hz, Ar**H**), 7.33 – 7.22 (m, 2H), 6.84 (ddd, 2H, *J* = 8.6 Hz, *J* = 3.1 Hz, *J* = 2.1 Hz, Ar**H**), 2.14 (t, 3H, *J* = 1.7 Hz, Ar-C**H$_3$**) |
| $^{19}$F NMR (376 MHz): | -114.88 (d, *J* = 8.6 Hz, Ar**F**) |

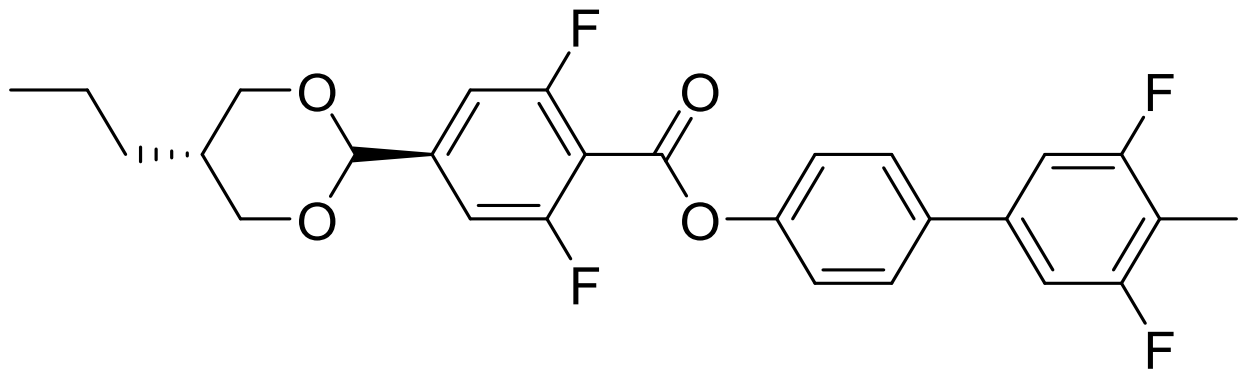

**3',5'-difluoro-4'-methyl-[1,1'-biphenyl]-4-yl 2,6-difluoro-4-(5-propyl-1,3-dioxan-2-yl)benzoate (WCO 4.7 (IDX1013)):** Quantities Used: 2,6-difluoro-4-(5-propyl-1,3-dioxan-2-yl)benzoic acid (231 mg, 0.807 mmol), 3',5'-difluoro-4'-methyl-[1,1'-biphenyl]-4-ol (157 mg, 0.713 mmol), EDC.HCl (339 mg, 1.768 mmol), DMAP (cat. amount), DCM (ca. 5 ml). The reaction was performed according to general procedure A. The crude material was purified by flash chromatography over silica gel with a gradient of hexane/DCM, followed by recrystallization from ethanol to afford the title compound as colourless needles.

Yield: 66 mg, 19 %

$^{1}$H (400 MHz): 7.58 (m, 2H, Ar-**H**), 7.33 m, 2H, Ar-**H**), 7.18 (d, *J* = 8.7 Hz, 2H, Ar-**H**), 7.12 – 7.03 (m, 2H, Ar-**H**), 5.40 (s, 1H, $ArO_2C$-**H**), 4.25 (dd, *J* = 11.8, *J* = 4.8 Hz, 2H, OC-$\mathbf{H_2}$), 3.54 (t, *J* = 11.5 Hz, 2H, OC-$\mathbf{H_2}$), 2.23 (s, 3H, ArC-$\mathbf{H_3}$), 2.20 – 2.07 (m, 1H, CCC-**H**), 1.35 (h, *J* = 7.5 Hz, 2H, C-$\mathbf{H_2}$), 1.11 (q, *J* = 8.3 Hz, 2H, C-$\mathbf{H_2}$), 0.94 (t, *J* = 7.3 Hz, 3H, C-$\mathbf{H_3}$)

$^{13}$C{$^{1}$H} (101 MHz): 162.00 (dd, *J* = 245.9 Hz, *J* = 9.6 Hz), 160.98 (dd *J* = 258.0 Hz, *J* = 5.4 Hz), 159.91, 150.45, 145.35, 139.80, 137.38, 128.12, 122.25, 112.42, 110.31 (dd, *J* = 23.9 Hz, 2.8 Hz), 109.64 (d, *J* = 8.6 Hz), 109.37 (d, *J* = 8.9 Hz), 99.01, 72.72, 34.04, 30.38, 19.67, 14.33, 7.13 (t, *J* = 3.6 Hz)

$^{19}$F NMR(376 MHz): -108.81 (d, *J* = 9.7 Hz, Ar-**F**), -114.52 (d, *J* = 7.6 Hz, Ar-**F**)

HRMS (ESI+): 489.1683 ([M+H], calcd. for ($C_{27}H_{25}F_4O_4$): 489.1683, err = 0.1 ppm)

HPLC: Assay (HPLC, C18): 99.96 % (254 nm, 5.308 min)

## 6.8. Scheme 8

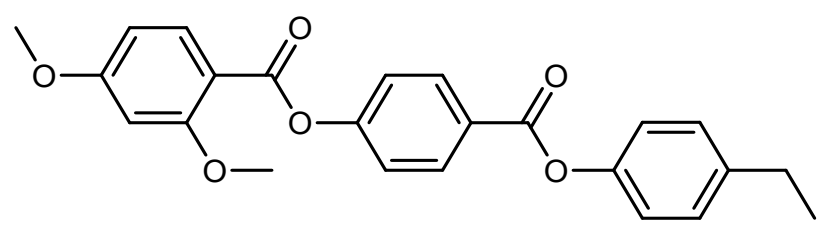

**4-((4-ethylphenoxy)carbonyl)phenyl-2,4-dimethoxybenzoate (COB30 IDX 1014)**

Quantities used: 4-ethylphenol (0.18 g, 1.5 mmol), 4-((2,4-dimethoxybenzoyl)oxy)benzoic acid (0.50 g, 1.65 mmol), **[4]** EDC.HCl (0.40 g, 2.1 mmol), DMAP (cat. amount), DCM (30 mL). The reaction was performed according to general procedure A. The crude material was purified by flash chromatography over silica gel, using a gradient of hexane/ethyl acetate, followed by recrystallisation from acetonitrile to give white crystals.

| | |
|---|---|
| Yield: | 0.39 g (64 %). |
| $R_F$: | 0.55 (DCM) |
| $^{1}$H NMR (400 MHz): | 8.25 (m, 2H, r-**H**), 8.09 (d, 1H, *J* = 8.7 Hz, Ar-**H**), 7.35 (m 2H, Ar-**H**), 7.25 m, 2H, Ar-**H**), 7.12 (m, 2H, Ar-**H**), 6.58 (dd, 1H, *J* = 8.7 Hz, *J* = 2.3 Hz, Ar-**H**), 6.54 (d, 1H, *J* = 2.3 Hz, Ar-**H**), 3.94 (s, 3H, R-O-$\mathbf{CH_3}$), 3.90 (s, 3H, R-O-$\mathbf{CH_3}$), 2.68 (q, 2H, *J* = 7.6 Hz, R-$\mathbf{CH_2}$-$CH_3$), 1.26 (t, 3H, *J* = 7.6 Hz, R-$CH_2$-$\mathbf{CH_3}$). |
| $^{13}$C{$^{1}$H} NMR (101 MHz): | 165.41, 164.90, 163.07, 162.59, 155.52, 148.95, 141.98, 134.76, 131.80, 128.96, 126.95, 122.34, 121.54, 110.76, 105.07, 99.17, 56.19, 55.76, 28.46, 15.73. |

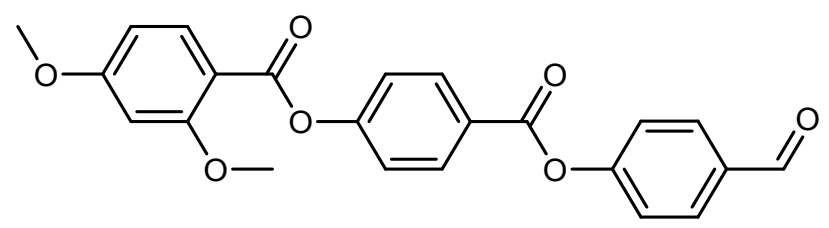

**4-((4-formylphenyloxy)carbonyl)phenyl 2,4-dimethoxybenzoate (COB27 IDX 1407)**

Quantities used: 4-hydroxybenzalehdye (0.18 g, 1.5 mmol), 4-((2,4-dimethoxybenzoyl)oxy)benzoic acid (0.50 g, 1.65 mmol), EDC.HCl (0.40 g, 2.1 mmol), DMAP (cat. amount), DCM (30 mL). The reaction was performed according to general procedure A. The crude material was purified by flash chromatography over silica gel, using a gradient of hexane/ethyl acetate, followed by recrystallisation from acetonitrile to give a white solid.

| | |
|---|---|
| Yield: | 0.09 g (15 %). |
| $R_F$: | 0.20 (DCM) |
| $^1$H NMR (400 MHz): | 10.03 (s, 1H, R-C**H**O), 8.26 (m, 2H, Ar-**H**), 8.10 (d, 1H, *J* = 8.8 Hz, AR-**H**), 7.98 (m, 2H, Ar-**H**), 7.42 (m, 2H, *J* = 8.6 Hz, Ar-**H**), 7.38 (m, 2H, Ar-**H**), 6.58 (dd, 1H, *J* = 8.7, *J* = 2.3 Hz, Ar-**H**), 3.94 (s, 3H, R-O-**$CH_3$**), 3.90 (s, 3H, R-O-**$CH_3$**). |
| $^{13}$C{$^1$H} NMR (101 MHz): | 191.10, 165.50, 164.03, 162.98, 162.65, 155.95, 155.80, 134.77, 134.23, 131.96, 131.43, 126.10, 122.70, 122.57, 110.59, 105.12, 99.17, 56.20, 55.77. |

## 6.9. Scheme 9

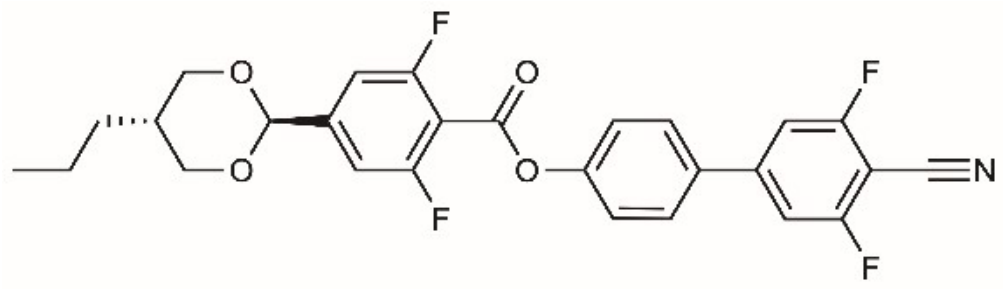

**3',5'-difluoro-4'-cyano-[1,1'-biphenyl]-4-yl 2,6-difluoro-4-(-5-propyl-1,3-dioxan-2-yl)benzoate (RM2926 / IDX2012)):**

Quantities Used: 2,6-difluoro-4-(5-propyl-1,3-dioxan-2-yl)benzoic acid (286 mg, 1 mmol), 3',5'-difluoro-4'-cyano-[1,1'-biphenyl]-4-ol (231 mg, 1 mmol), EDC.HCl (382 mg, 2 mmol), DMAP (5 mg, 0.04 mmol), DCM (10 ml). The reaction was performed according to general procedure A. The crude material was purified by flash chromatography over silica gel with a gradient of hexane/DCM, followed by recrystallization from acetonitrile to afford the title compound as a colourless microcrystalline solid.

Yield: 395 mg, 79 %

$^1$H (500 MHz): 7.59 – 7.51 (2H, m, Ar**H**), 7.36 – 7.29 (2H, m, Ar**H**), 7.22 – 7.16 (2H, m, Ar**H**), 7.15 – 7.07 (2H, m, Ar**H**), 5.33 (1H, s, Ar-C**H**($OCH_2$)$_2$-CH-$CH_2$-),), 4.21 – 4.14 (2H, m, Ar-CH(OCH**H**)$_2$-CH-$CH_2$-), 3.51 – 3.43 (2H, m, Ar-CH(OC**H**H)$_2$-CH-$CH_2$-), 2.07 (1H, ttt, *J* = 11.4, 7.0, 4.6 Hz, Ar-CH($OCH_2$)$_2$-C**H**-$CH_2$-), 1.33 – 1.22 (2H, m, -C**H₂**-$CH_2$-$CH_3$), 1.07 – 0.99 (2H, m, -$CH_2$-C**H₂**-$CH_3$), 0.86 (3H, t, *J* = 7.3 Hz, -$CH_2$-$CH_2$-C**H₃**).

$^{13}$C{$^1$H} (125 MHz): 163.53 (dd, *J* = 261.0, 5.2 Hz), 161.01 (dd, *J* = 258.1, 5.8 Hz), 159.65, 151.77, 148.47 (t, *J* = 9.8 Hz), 145.66 (t, *J* = 9.8 Hz), 135.32, 128.53, 122.82, 110.70 (dd, *J* = 20.4, 3.3 Hz), 110.40 (dd, *J* = 23.6, 3.4 Hz), 109.73 (t, *J* = 17.1 Hz), 109.39, 98.91 (d, *J* = 2.3 Hz), 91.01 (t, *J* = 19.4 Hz), 72.70, 34.02, 30.34, 19.65, 14.31.

$^{19}$F NMR(376 MHz): -103.24 (2F, d, *J* = 9.3 Hz, Ar**F**), -108.62 (2F, d, *J* = 9.9 Hz, Ar**F**),

HPLC: Assay (HPLC, C18): 99.12% (254 nm, 5.1 min)

## 6.10 Scheme 10

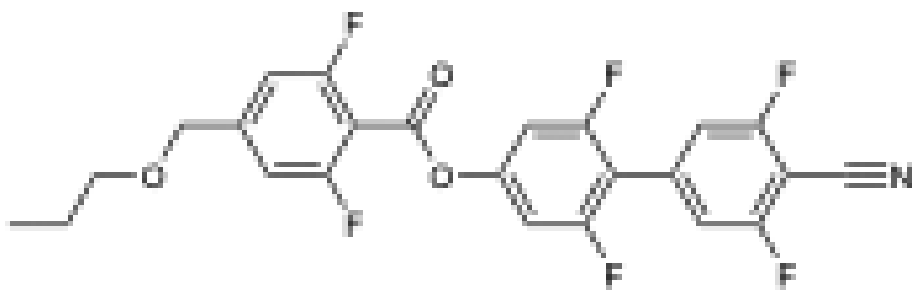

**4'-cyano-2,3',5',6-tetrafluoro-[1,1'-biphenyl]-4-yl 2,6-difluoro-4-(propoxymethyl)benzoate (SRB-CHEM-163 / IDX2.0713)):**

Quantities Used: 2,6-difluoro-4-(propoxymethyl)benzoic acid (230 mg, 1 mmol), 3',5'-difluoro-4'-cyano-[1,1'-biphenyl]-4-ol (134 mg, 0.5 mmol), EDC.HCl (191 mg, 1 mmol), DMAP (5 mg, 0.04 mmol), DCM (10 ml). The reaction was performed according to general procedure A. The crude material was purified by flash chromatography over silica gel with a gradient of hexane/DCM, followed by recrystallization from acetonitrile to afford the title compound as a colourless microcrystalline solid.

Yield: 395 mg, 79 %

$^{1}$H (500 MHz): 7.24 – 7.18 (m, 2H, Ar**H**), 7.09 – 7.01 (m, 4H, Ar**H**), 4.55 (d, *J* = 0.8 Hz, 2H, ArC**H₂**OR), 3.49 (t, *J* = 6.6 Hz, 2H, BnOC$\mathbf{H}_2$R), 1.68 (m, 2H, BnOC$H_2$C$\mathbf{H_2}$C$H_3$), 0.98 (t, *J* = 7.4 Hz, 3H, C$H_2$-C$\mathbf{H_3}$).

$^{13}$C{$^{1}$H} (125 MHz): 164.02 (d, *J* = 5.0 Hz), 162.22 (dd, *J* = 68.9, 5.3 Hz), 160.57 (dd, *J* = 35.4, 6.9 Hz), 158.95 (d, *J* = 2.1 Hz), 158.71 (d, *J* = 8.2 Hz), 151.92 (t, *J* = 14.4 Hz), 148.13 (t, *J* = 9.7 Hz), 136.73 (t, *J* = 10.6 Hz), 114.40 (dq, *J* = 20.9, 2.7 Hz), 113.13 (t, *J* = 17.6 Hz), 110.57 (dd, *J* = 22.4, 3.5 Hz), 107.57 (t, *J* = 16.4 Hz), 107.34 – 106.56 (m), 92.40 (t, *J* = 19.2 Hz), 73.06, 71.09, 23.03, 10.74.

$^{19}$F NMR(376 MHz): -103.63 (d, Ar**F**, *J* = 8.9 Hz), -108.20 (d, Ar**F**, *J* = 10.1 Hz), -111.56 (d, Ar**F**, *J* = 9.4 Hz).

Assay (HPLC, C18): 99.41% (254 nm, 4.2 min)

### 6.11. Scheme 11

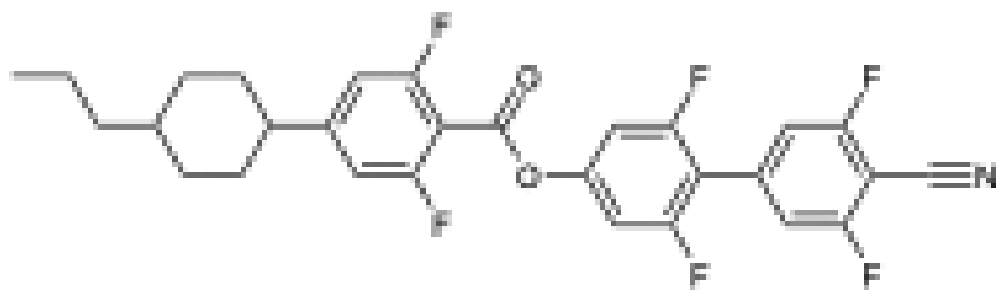

**4'-cyano-2,3',5',6-tetrafluoro-[1,1'-biphenyl]-4-yl 2,6-difluoro-4-(4-propylcyclohexyl)benzoate (WCO 4.19 / IDX2.1471)**

Quantities Used: *trans* 4-(4-propylcyclohexyl)-2,6-difluorobenzoic acid (278 mg, 0.985 mmol), 3',5'-difluoro-4'-cyano-[1,1'-biphenyl]-4-ol (176 mg, 0.659 mmol), EDC.HCl (299 mg, 1.56 mmol), DMAP (5 mg, 0.04 mmol), DCM (20 ml). The reaction was performed according to general procedure A. The crude material was purified by flash chromatography over silica gel with a gradient of hexane/DCM (crude yield, 220 mg), followed by recrystallization from ethanol to afford the title compound as a colourless microcrystalline solid.

Yield: 132 mg (36%)

$^{1}$H (500 MHz): 7.24 – 7.18 (m, 2H, Ar**H**), 7.09 – 6.99 (m, 2H, Ar**H**), 6.96 – 6.85 (m, 2H, Ar**H**), 2.53 (tt, *J* = 12.2, 3.2 Hz, 1H, *trans* Cy**H**-Ar), 1.91 (ddt, *J* = 13.8, 7.3, 2.9 Hz, 2H, Cy**H**), 1.48 – 1.25 (m, 4H), 1.06 (tdd, *J* = 13.0, 11.7, 3.6 Hz, 2H), 0.91 (t, *J* = 7.3 Hz, 3H, -$CH_2$-C$\mathbf{H_3}$).

$^{13}$C{$^{1}$H} (125 MHz): 163.29 (d, *J* = 5.1 Hz, 264.1 Hz), 161.41 (d, *J* = 5.8 Hz, 263.2 Hz), 160.35 (d, *J* = 5.9 Hz), 159.6 (d, *J* = 8.2 Hz, 253.2 Hz), 158.99 (t, *J* = 2.2 Hz), 156.87 (t, *J* = 9.4 Hz), 151.89 (t, *J* = 14.4 Hz), 136.65 (t, *J* = 10.7 Hz), 115.47 – 113.74 (m), 112.90 (t, *J* = 17.6 Hz), 110.90 (dd, *J* = 21.6, 3.2 Hz), 108.97, 107.86 – 106.49 (m), 106.06 (t, *J* = 16.1 Hz), 92.25 (t, *J* = 19.2 Hz), 44.68 (t, *J* = 1.7 Hz), 39.47, 36.80, 33.61, 33.04, 19.98, 14.36.

$^{19}$F NMR(376 MHz): -103.63 (d, *J* = 9.0 Hz, Ar**F**), -108.73 (d, *J* = 10.5 Hz, Ar**F**), -111.66 (d, *J* = 9.5 Hz, **ArF**).

## 10. Analytical Data

### 10.1. Analytical Data for Scheme 1

#### 10.1.1. 3,5-Difluorobenzyl propyl ether

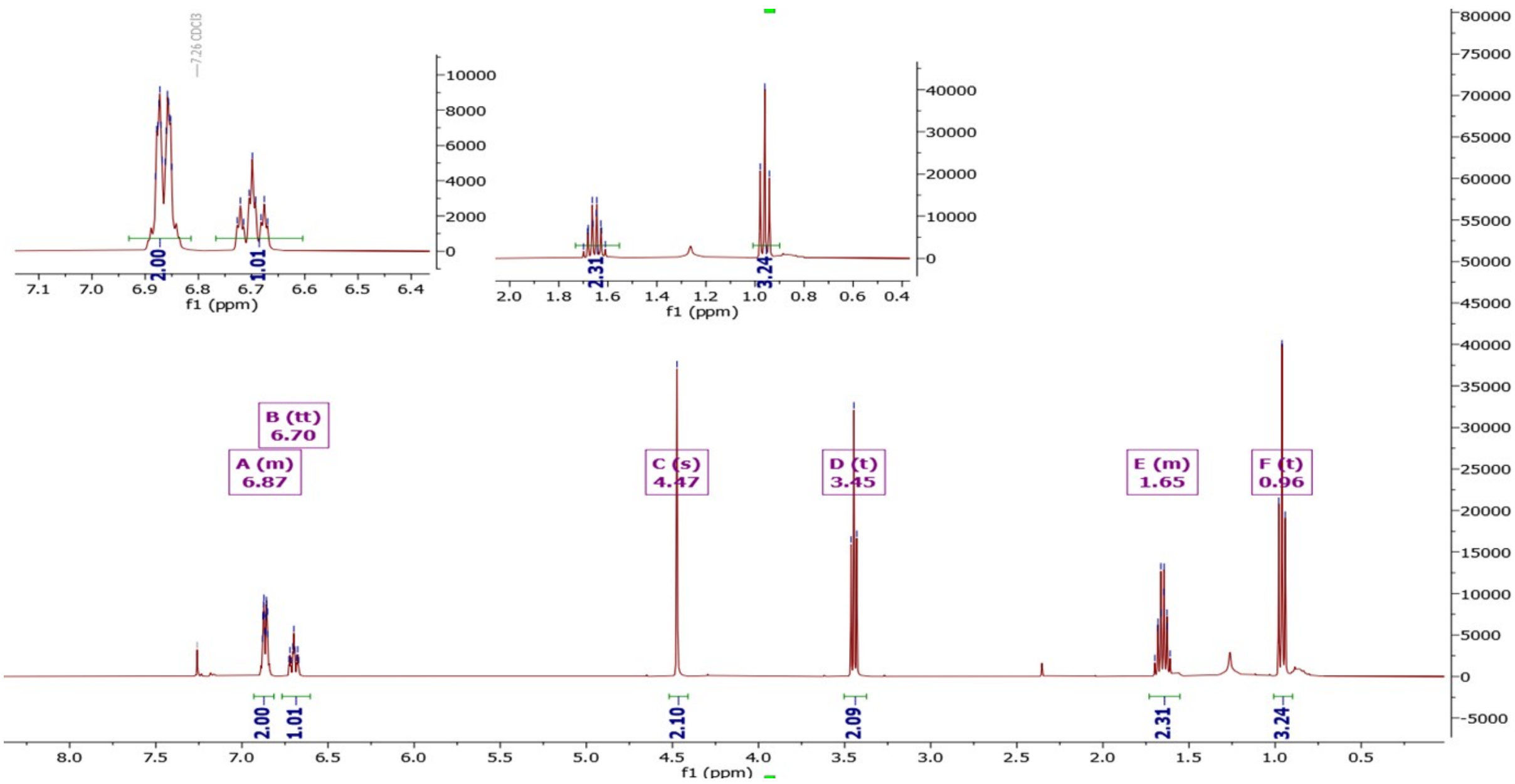


**Figure SI-26:** $^{1}$H NMR of 3,5-difluorobenzyl propyl ether

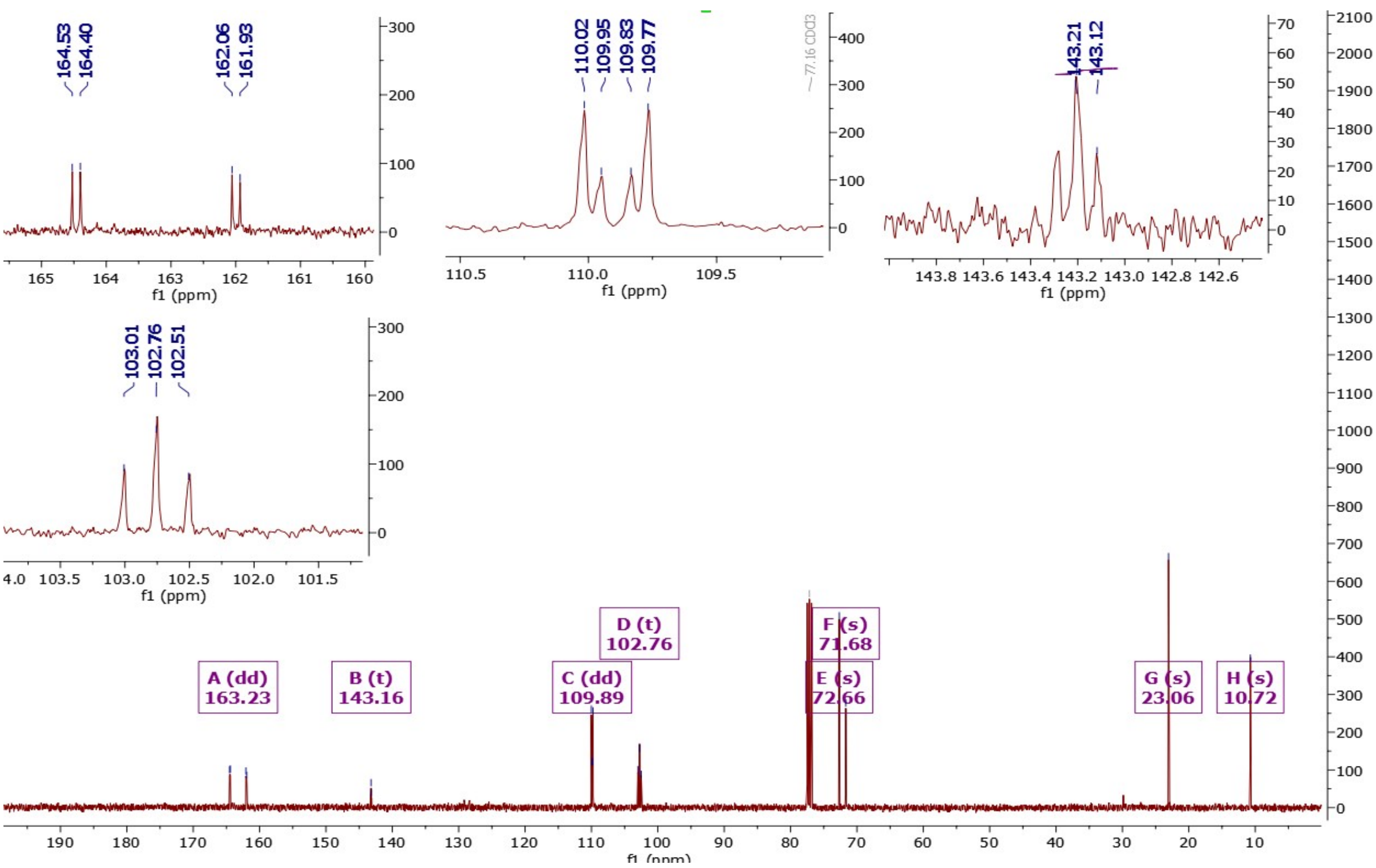


**Figure SI-27:** $^{13}C\{^{1}H\}$ NMR of 3,5-difluorobenzyl propyl ether

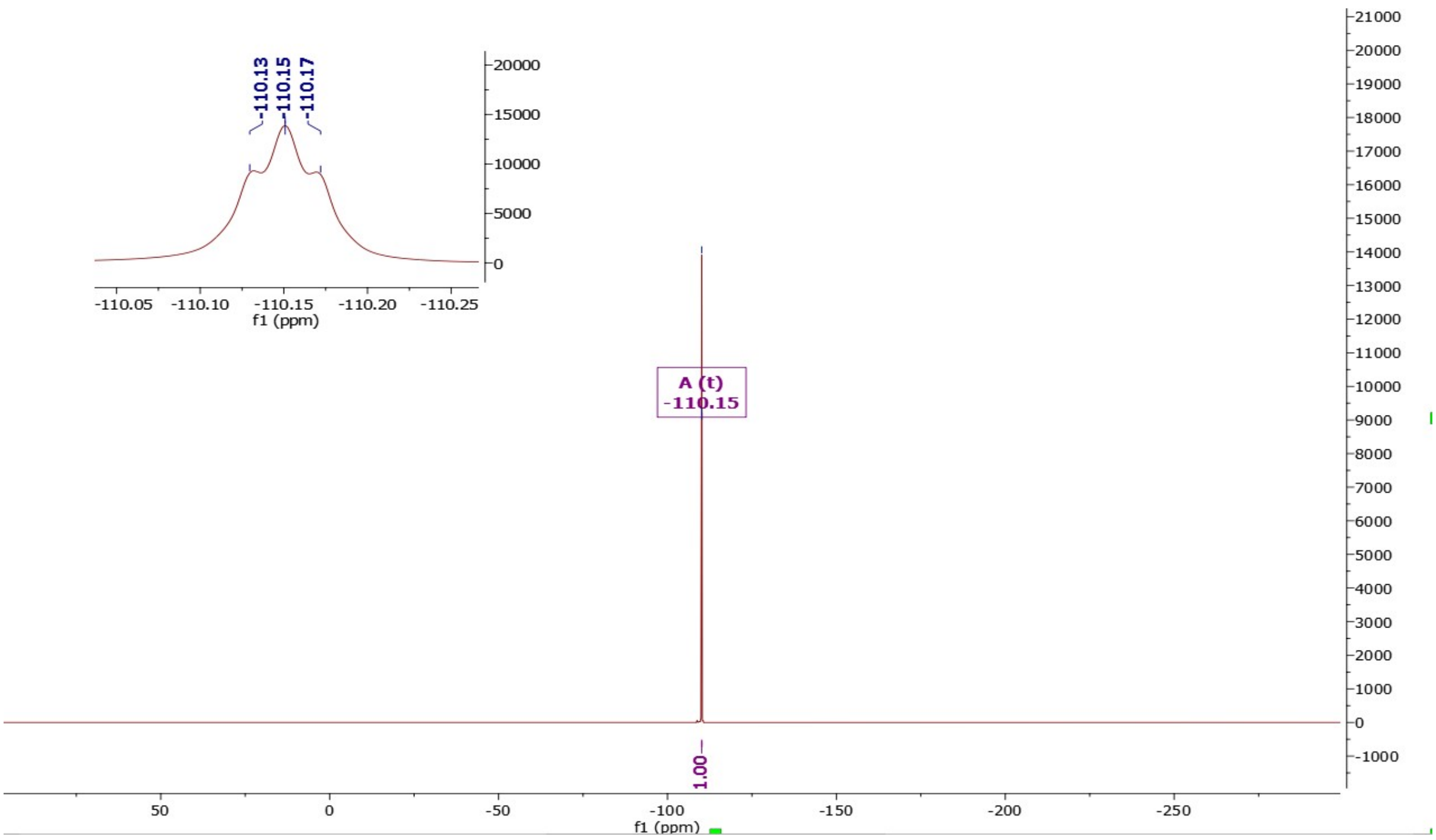


**Figure SI-28:** $^{19}F$ NMR of 3,5-difluorobenzyl propyl ether

### 7.1.2. 2,6-difluoro-2-(propan-1-yloxy)benzoic acid

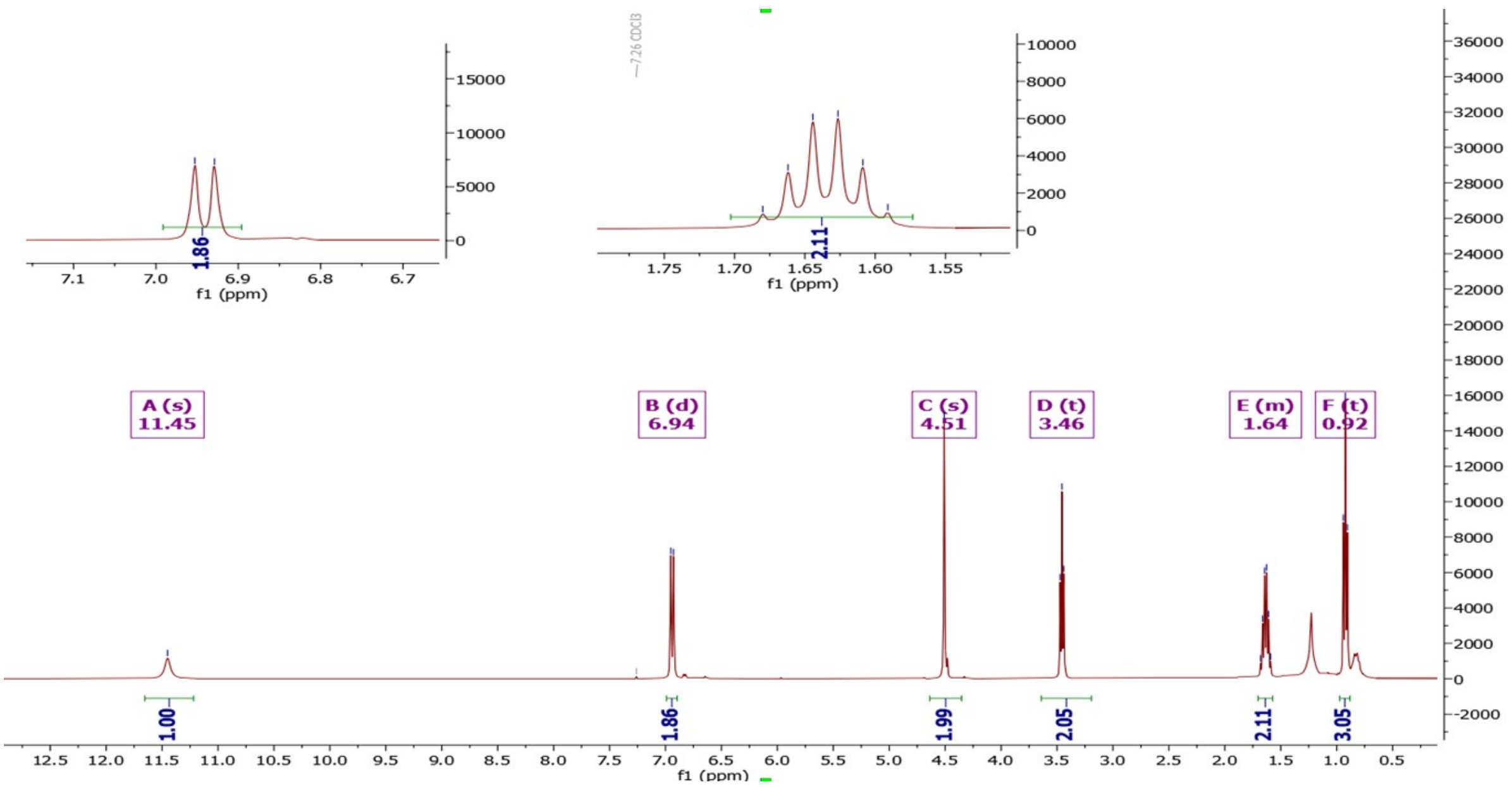


**Figure SI-29:** 1H NMR of 2,6-difluoro-2-(propan-1-yloxy)benzoic acid

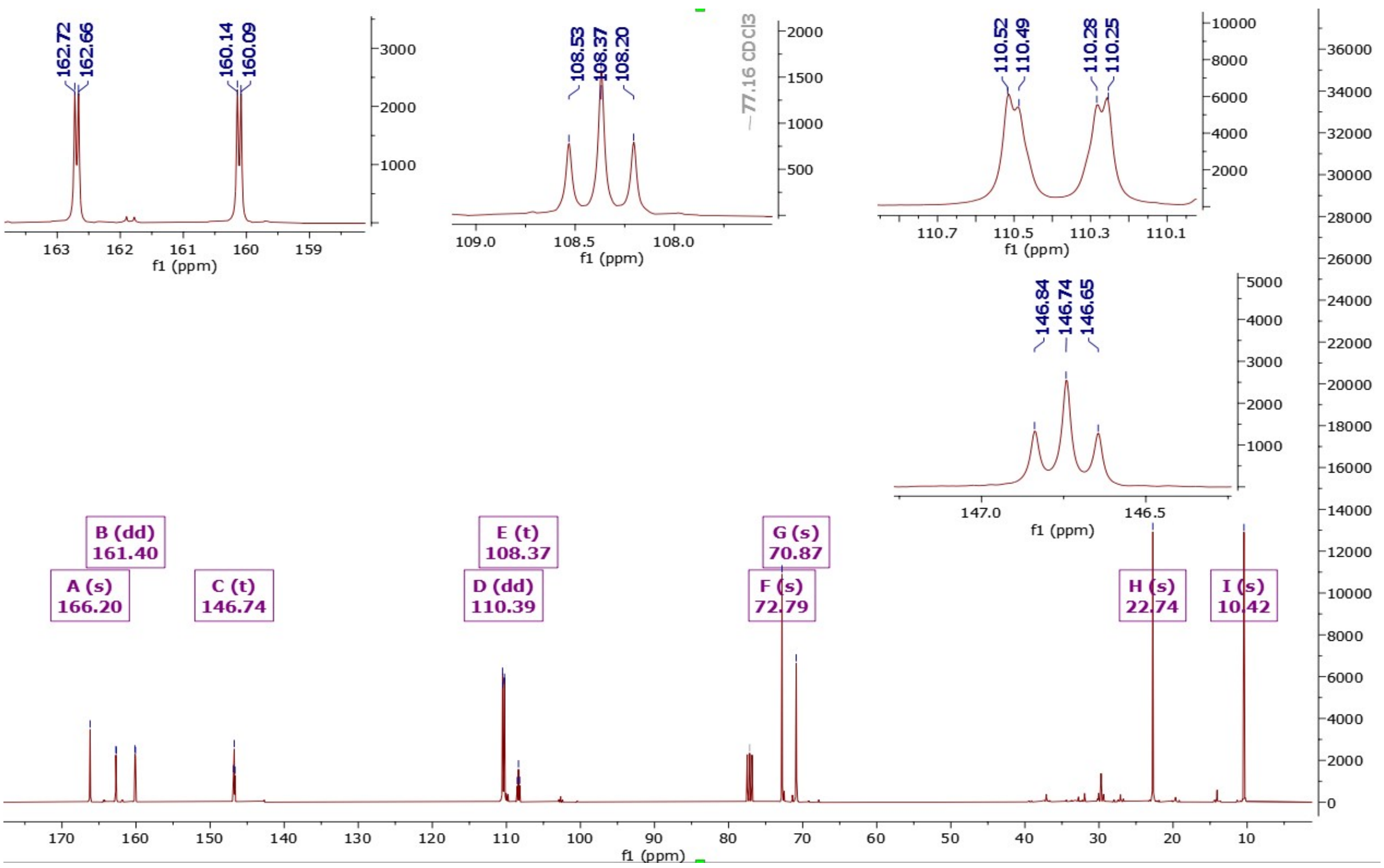


**Figure SI-30:** $^{13}C\{^{1}H\}$ NMR of 2,6-difluoro-2-(propan-1-yloxy)benzoic acid

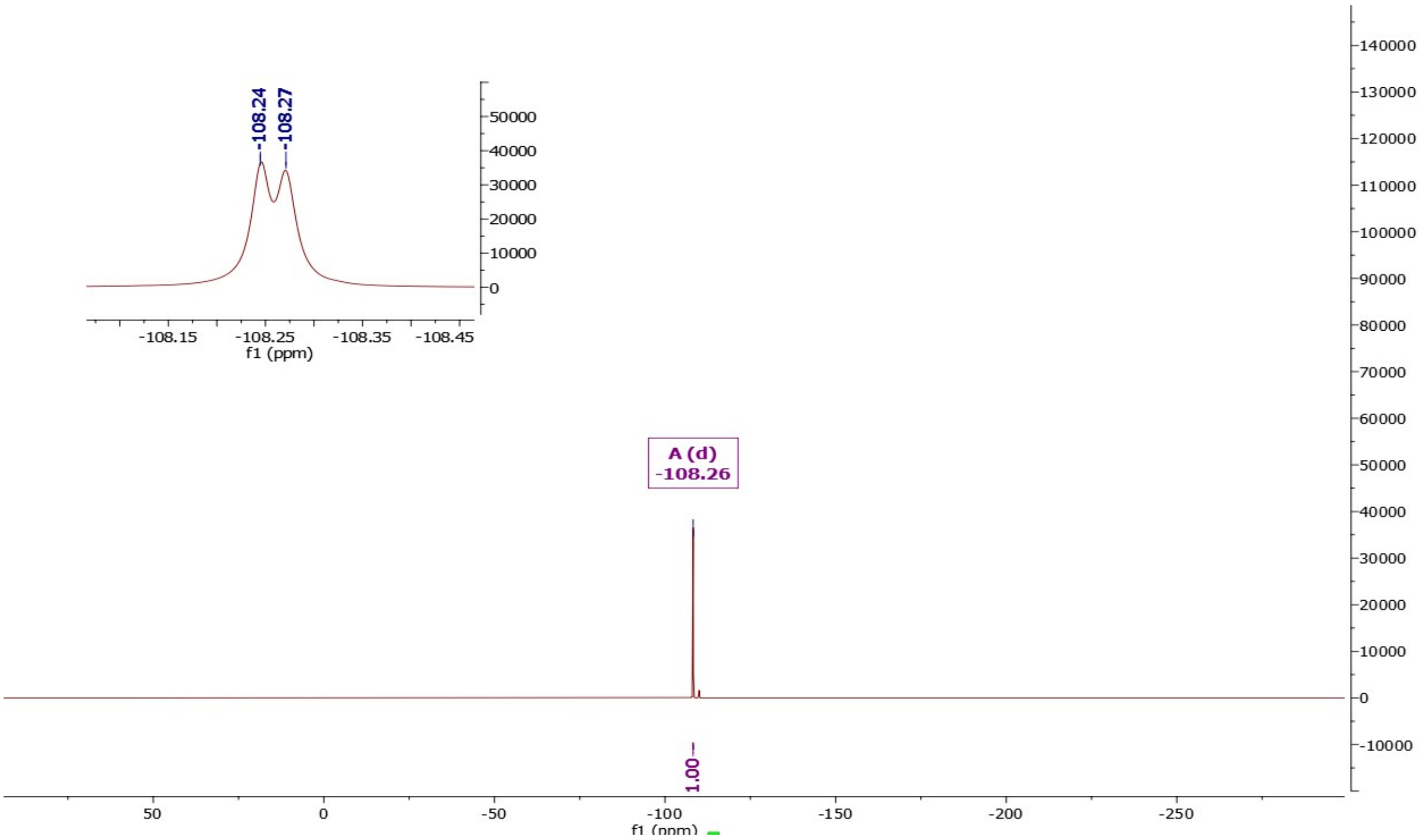


**Figure SI-31:** $^{19}F$ NMR of 2,6-difluoro-2-(propan-1-yloxy)benzoic acid

### 10.1.3. (2,3',4',5',6-pentafluoro-[1,1'-biphenyl]-4-yl)methyl 2,6-difluoro-4-(propoxymethyl)benzoate

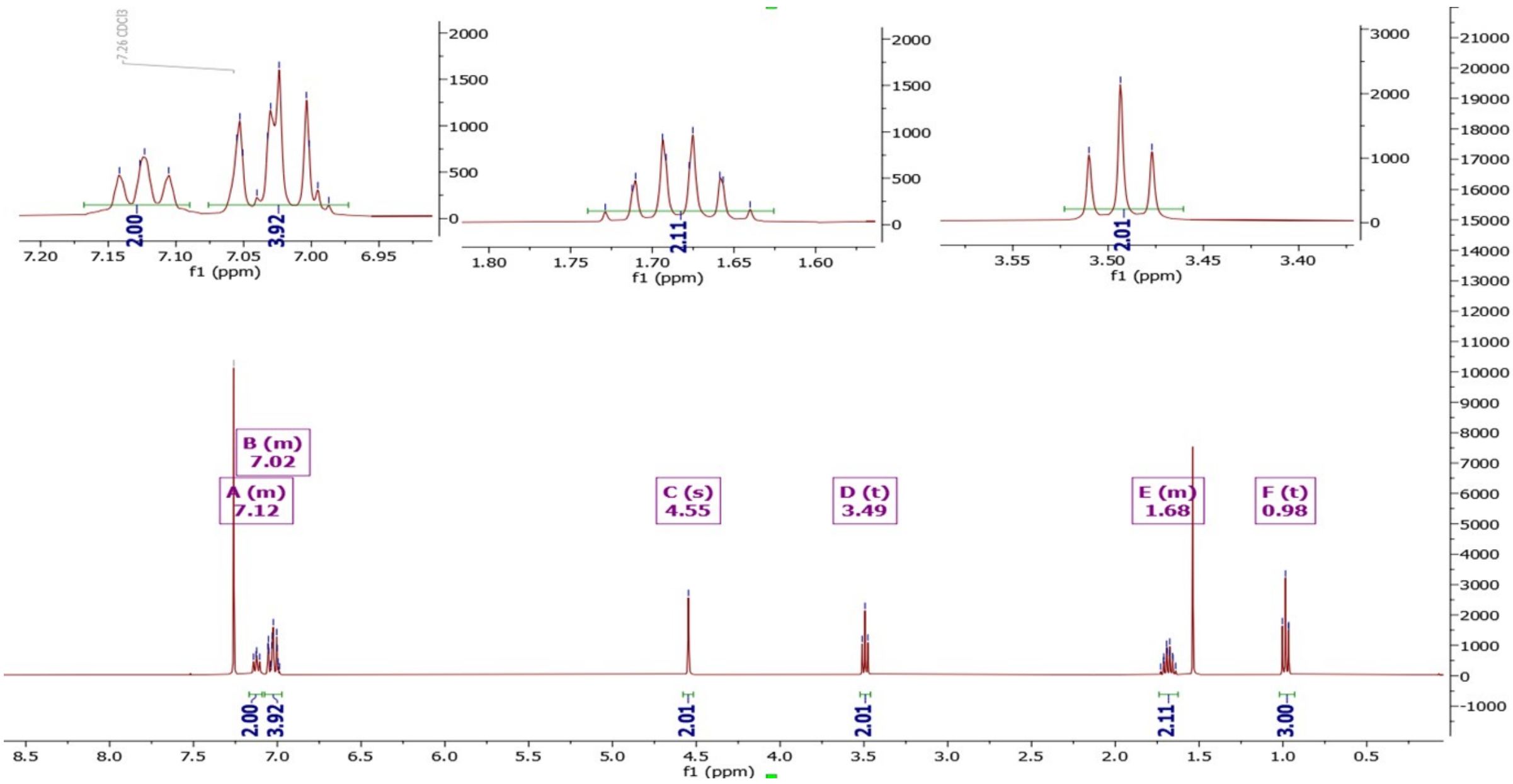


**Figure SI-32:** $^{1}$H NMR for (2,3',4',5',6-pentafluoro-[1,1'-biphenyl]-4-yl)methyl 2,6-difluoro-4-(propoxymethyl)benzoate

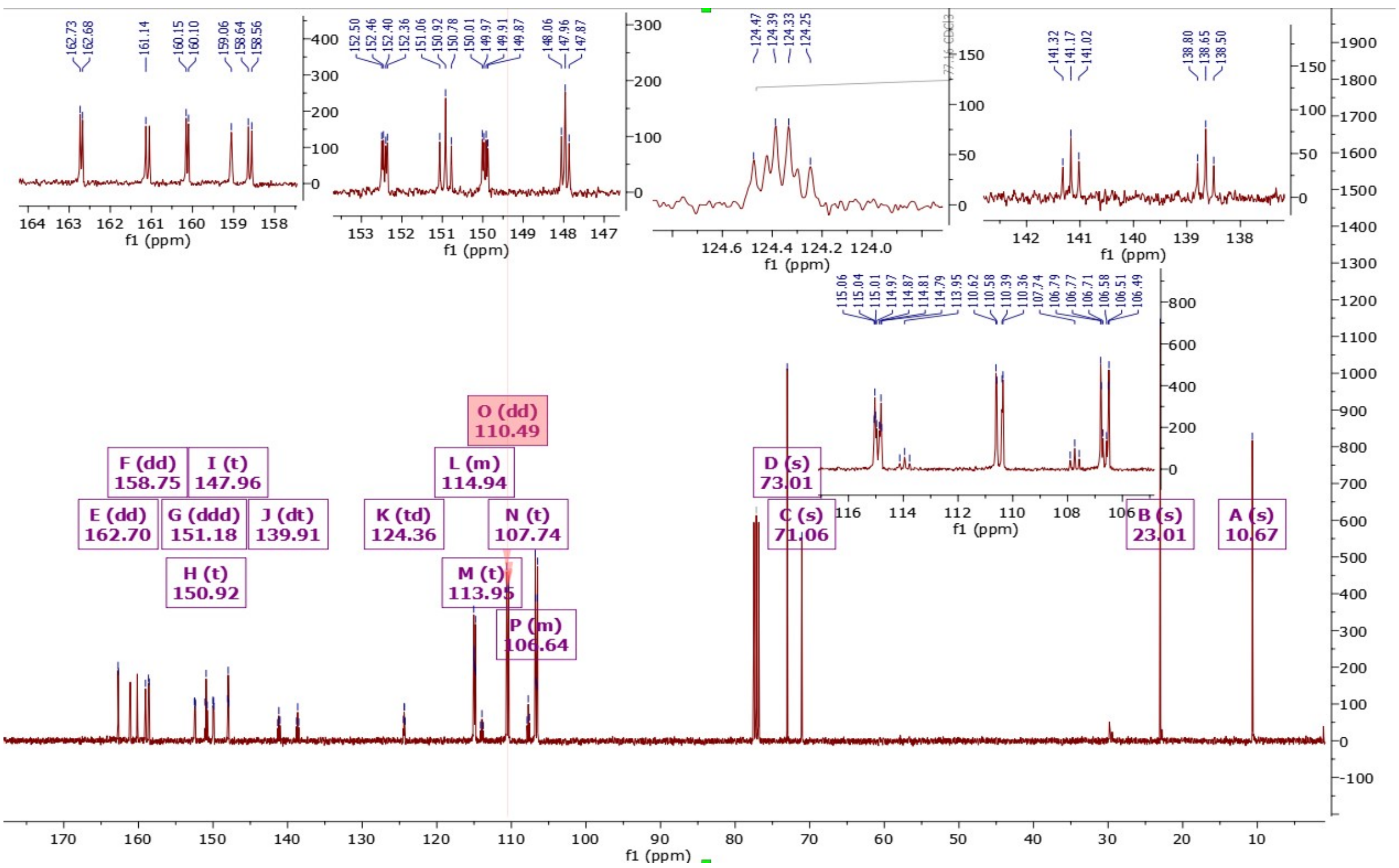


**Figure SI-33:** $^{13}$C{$^{1}$H} NMR for (2,3',4',5',6-pentafluoro-[1,1'-biphenyl]-4-yl)methyl 2,6-difluoro-4-(propoxymethyl)benzoate

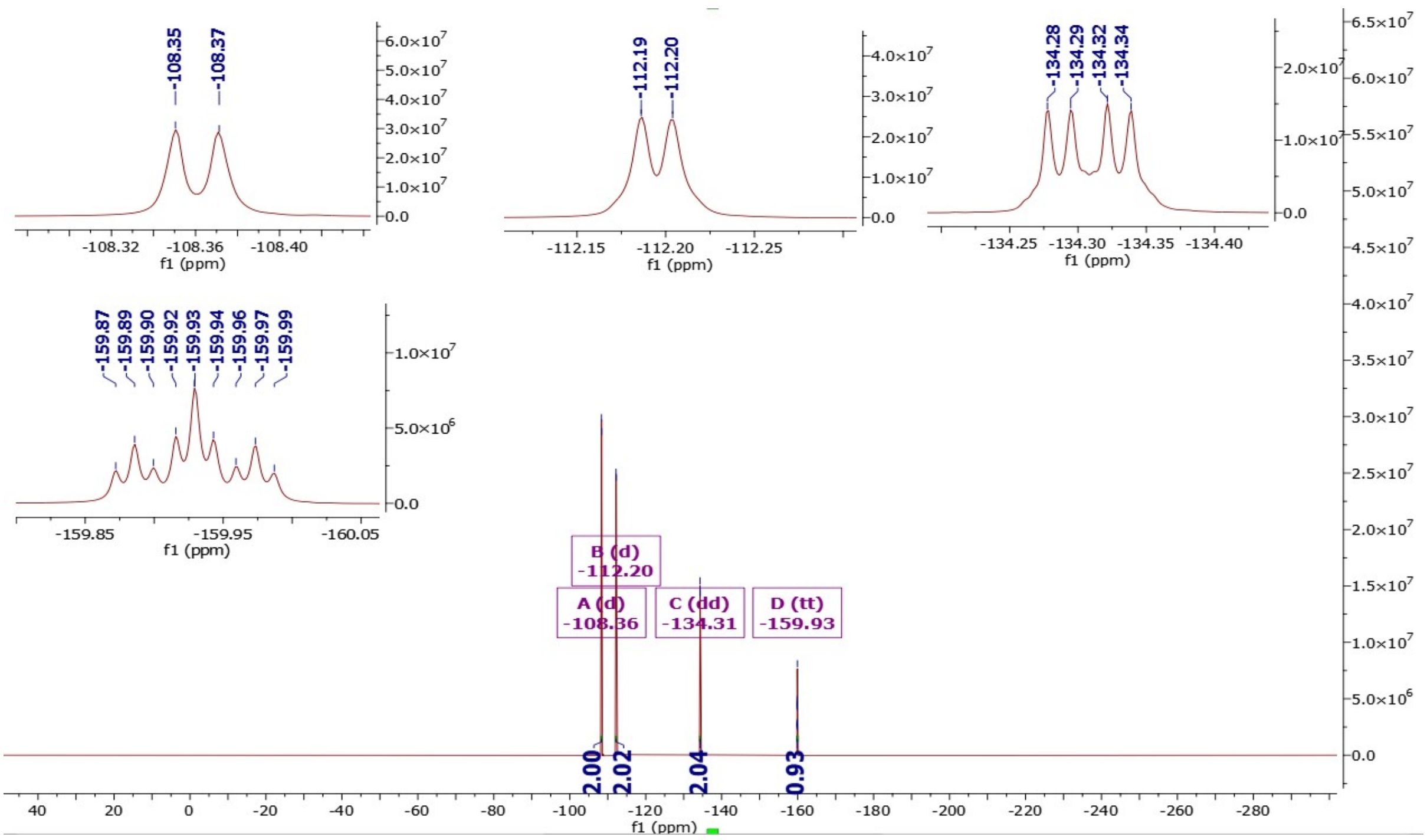


**Figure SI-34:** $^{19}F$ NMR for (2,3',4',5',6-pentafluoro-[1,1'-biphenyl]-4-yl)methyl 2,6-difluoro-4-(propoxymethyl)benzoate

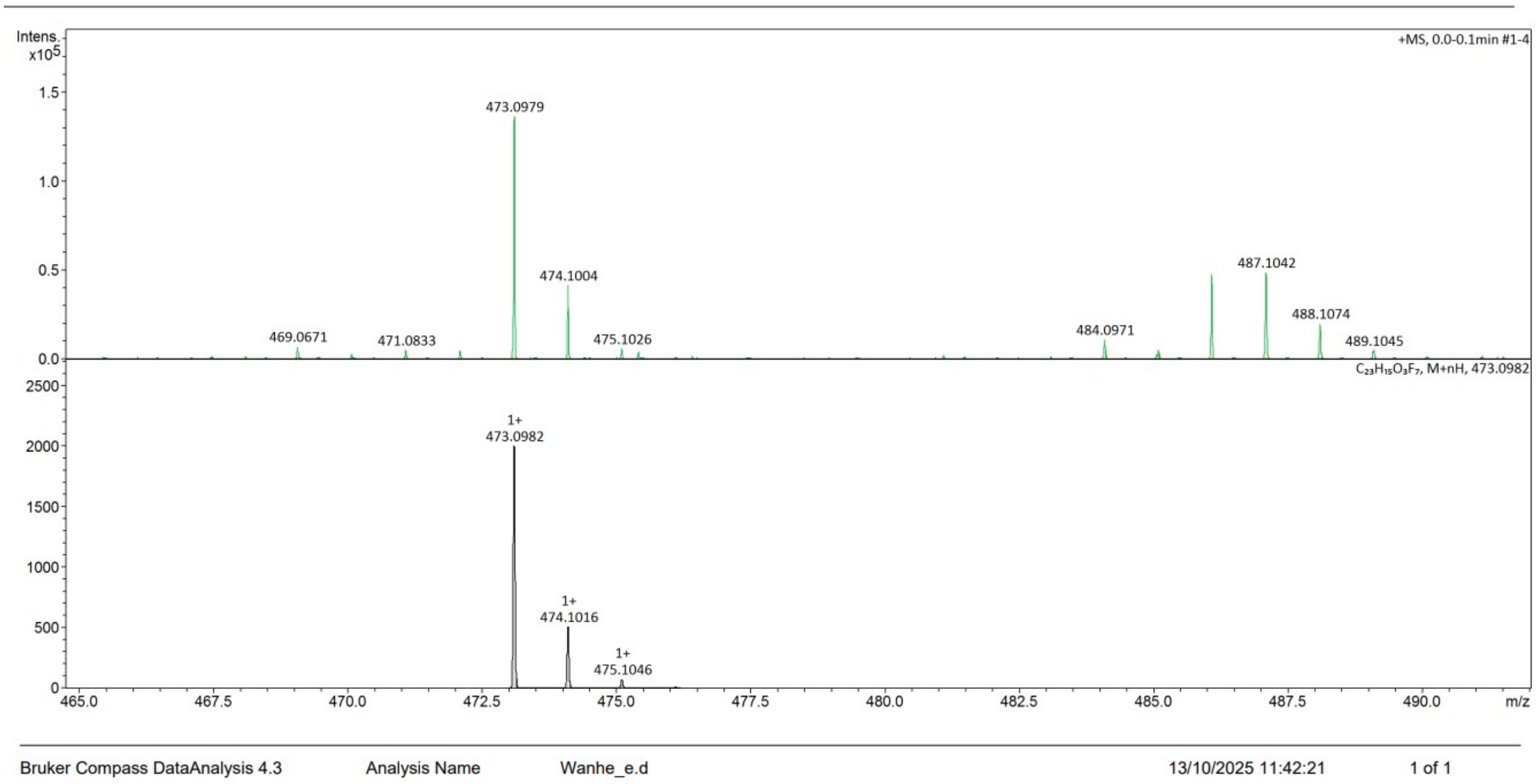


**Figure SI-35:** HRMS for (2,3',4',5',6-pentafluoro-[1,1'-biphenyl]-4-yl)methyl 2,6-difluoro-4-(propoxymethyl)benzoate

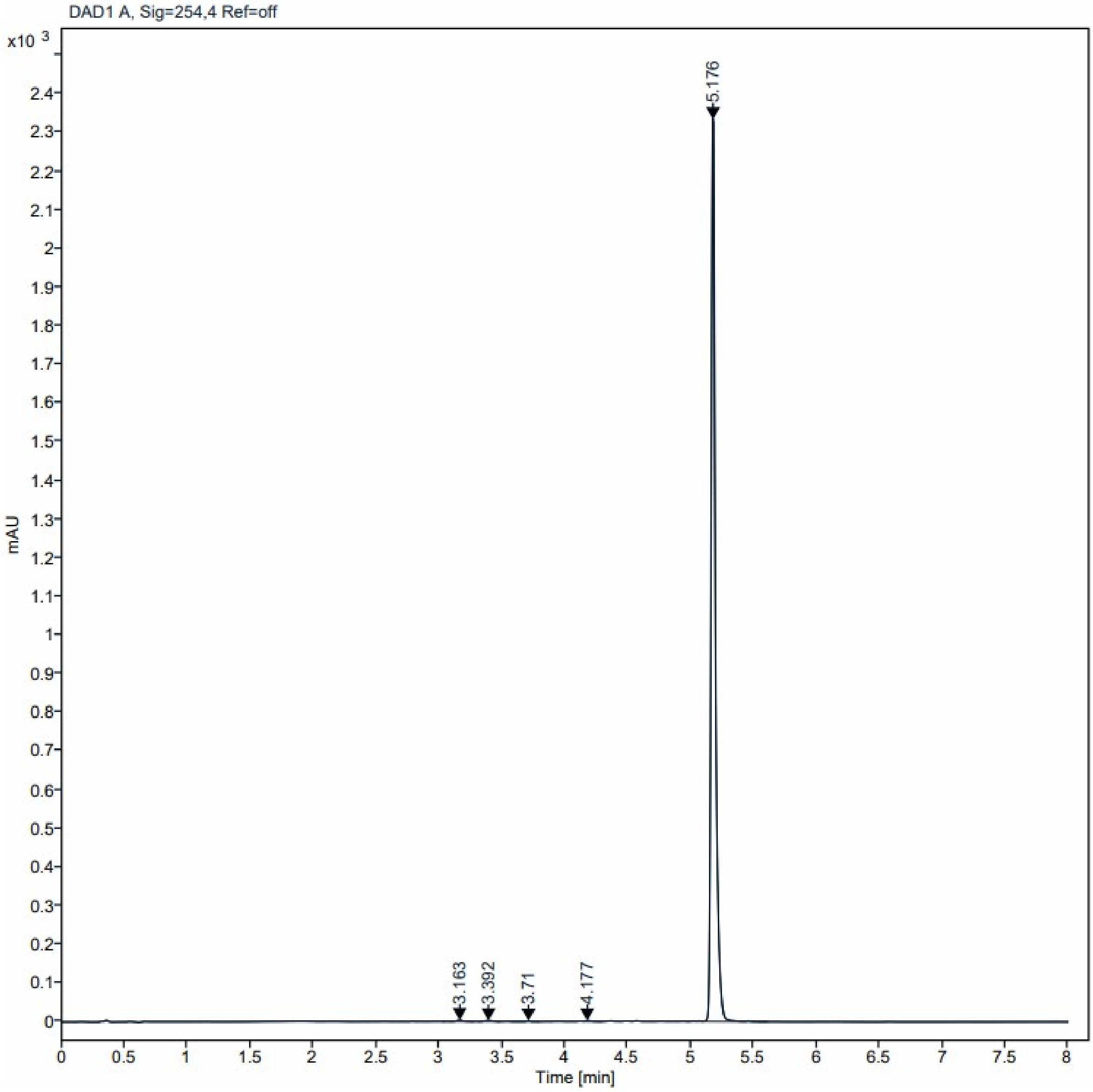


**Figure SI-36:** RP-HPLC for (2,3',4',5',6-pentafluoro-[1,1'-biphenyl]-4-yl)methyl 2,6-difluoro-4-(propoxymethyl)benzoate

### 10.2.1. Analytical data for IDX2538

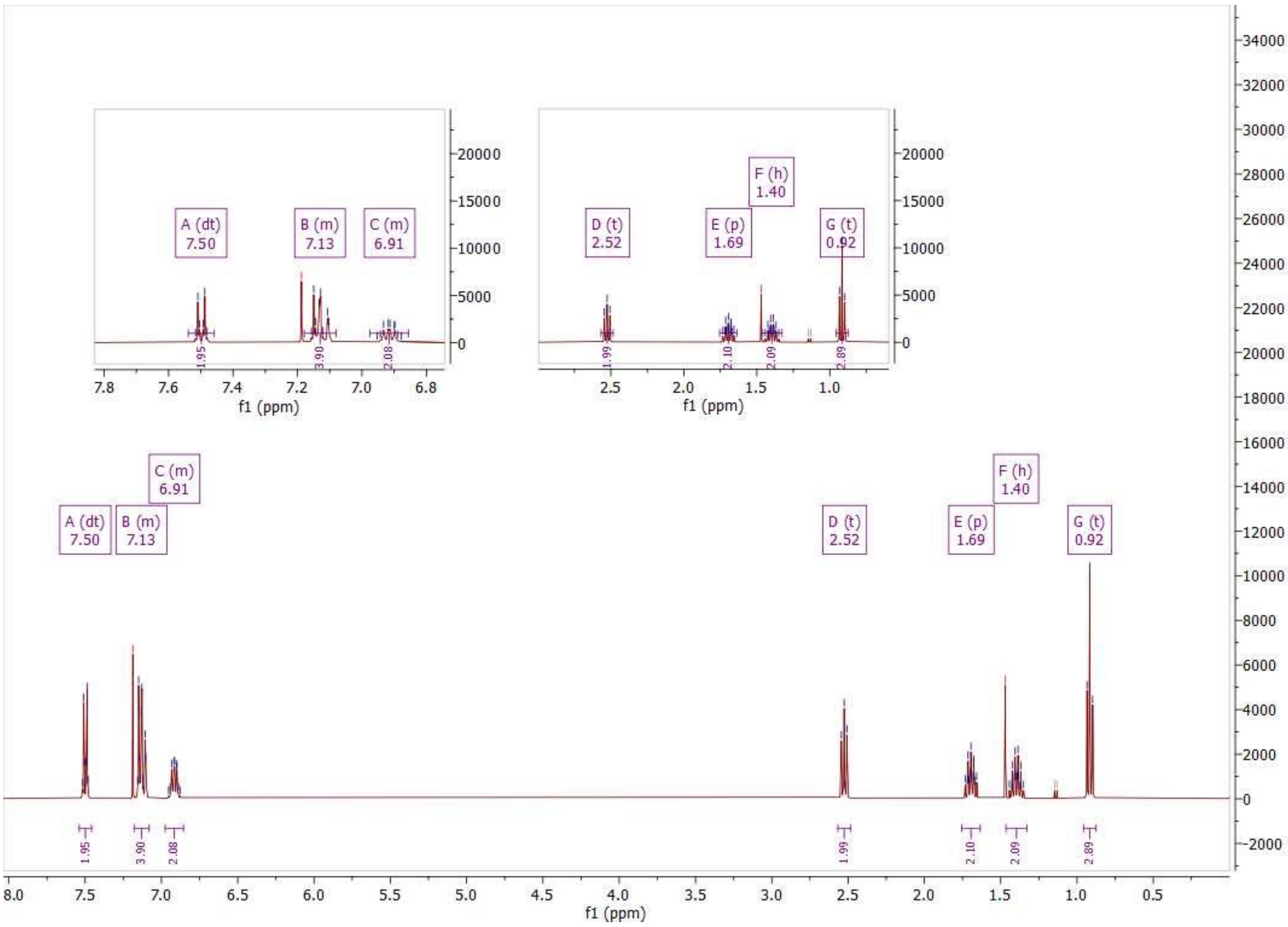


**Figure SI-37:** $^1$H NMR for IDX2538

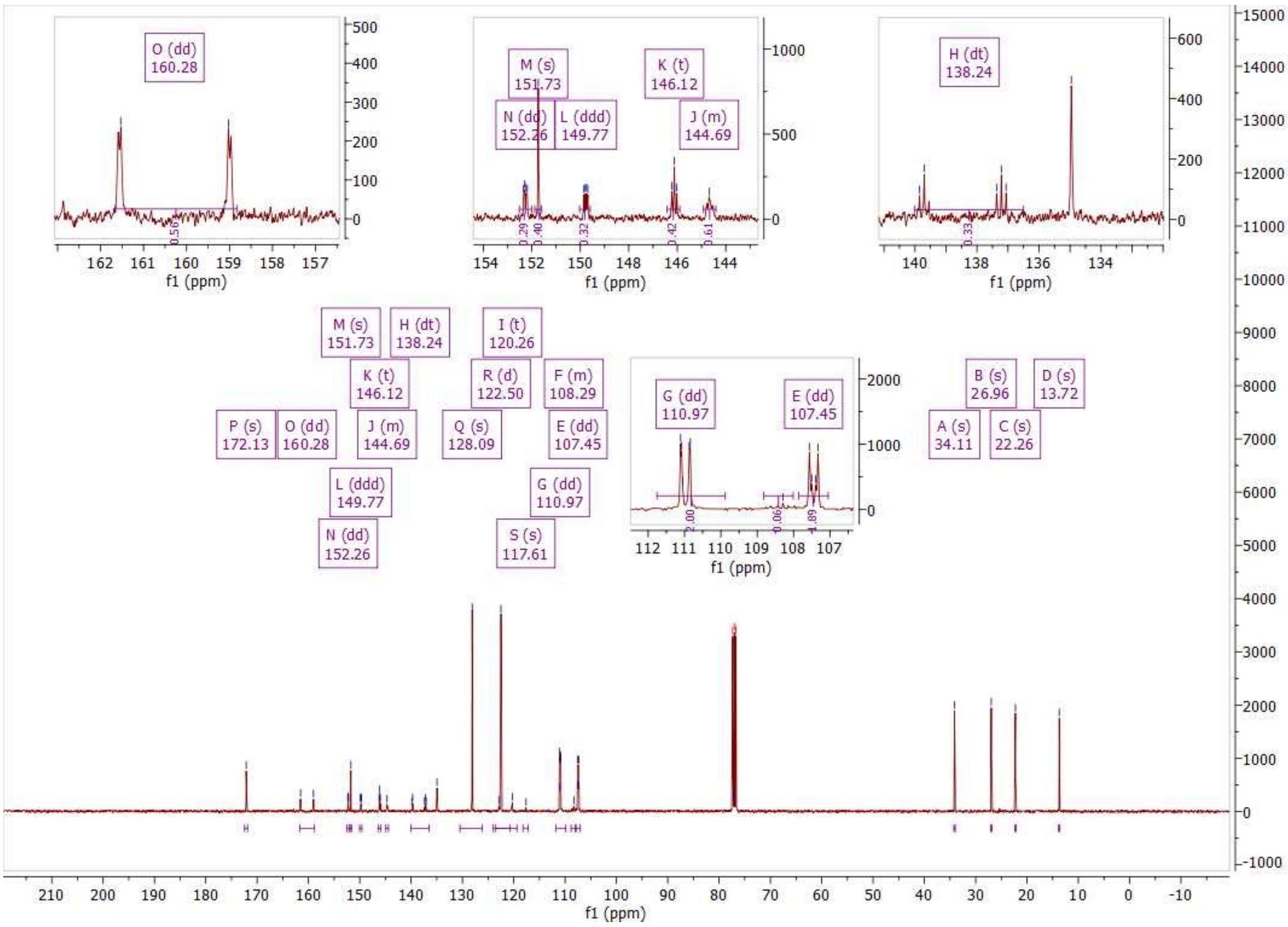


**Figure SI-38:** $^{13}C\{^{1}H\}$ NMR for IDX2538

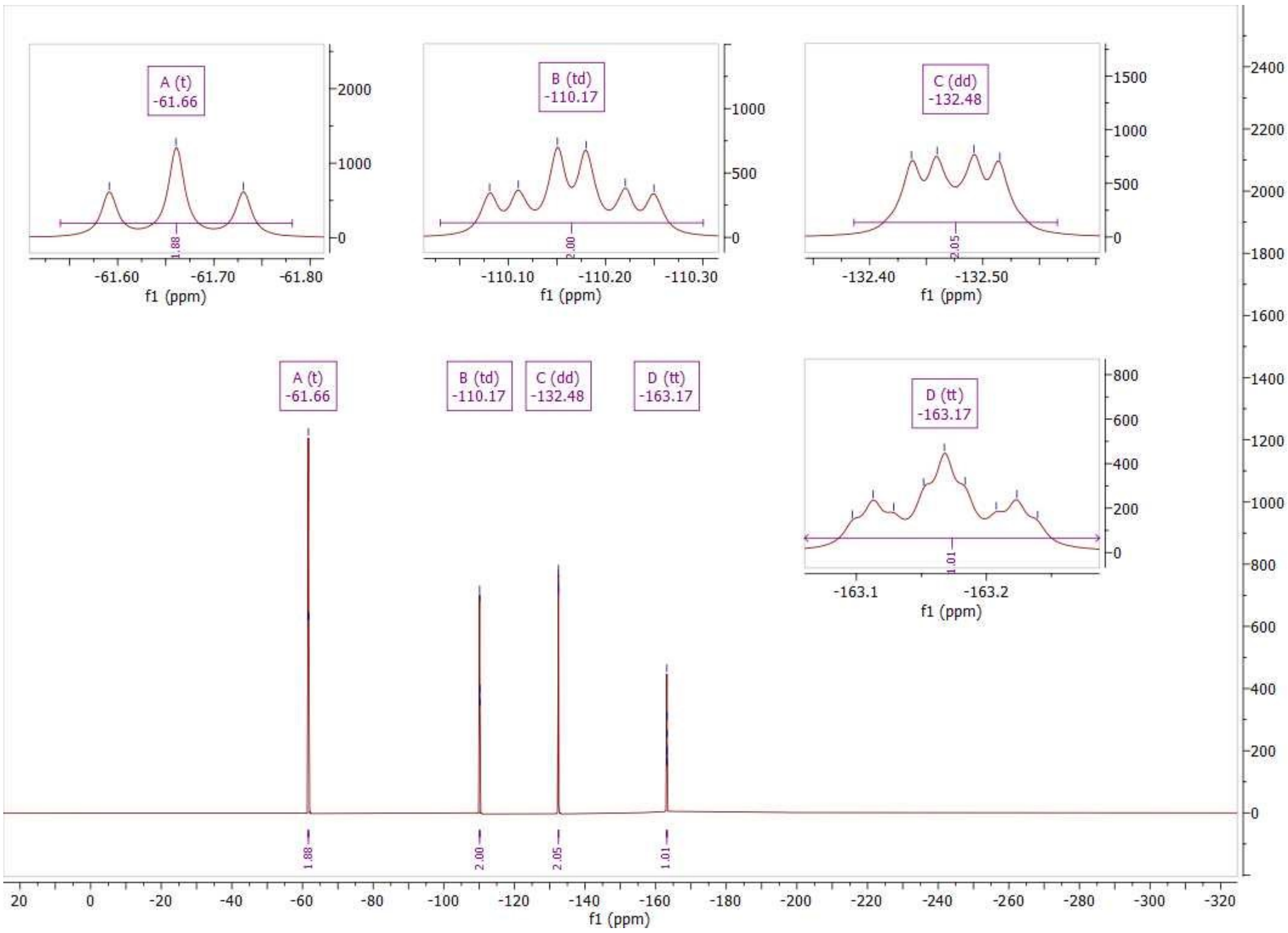


**Figure SI-39:** $^{19}$F NMR for IDX2538

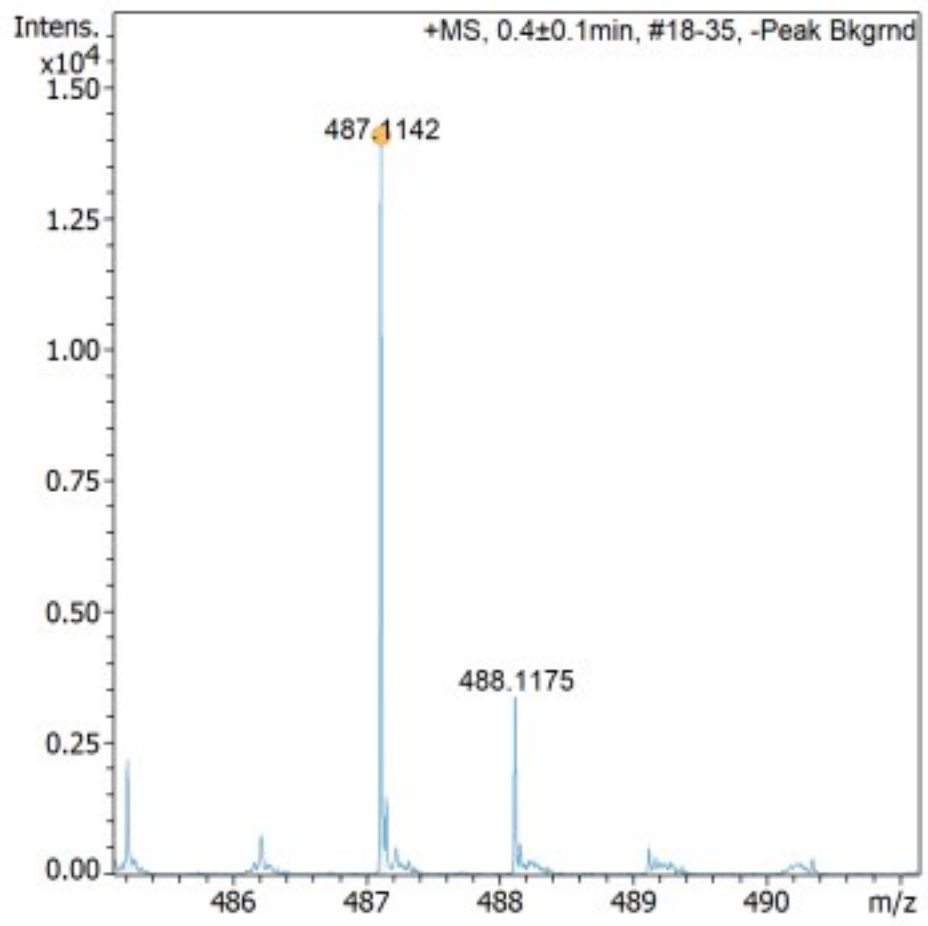


**Figure SI-40:** HRMS for IDX2538

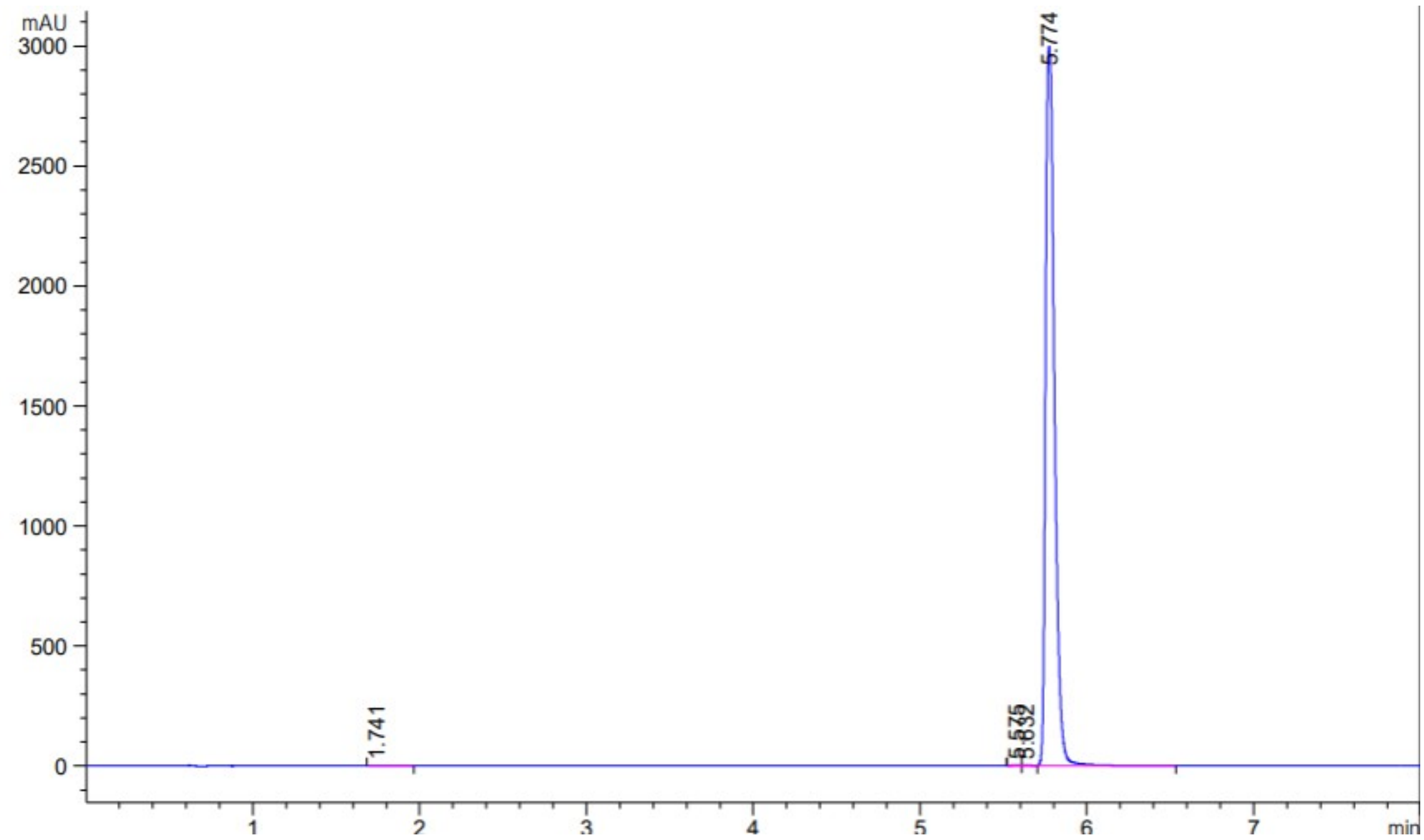


**Figure SI-41:** RP-HPLC for IDX2538

### 10.2.2. Analytical data for IDX1646

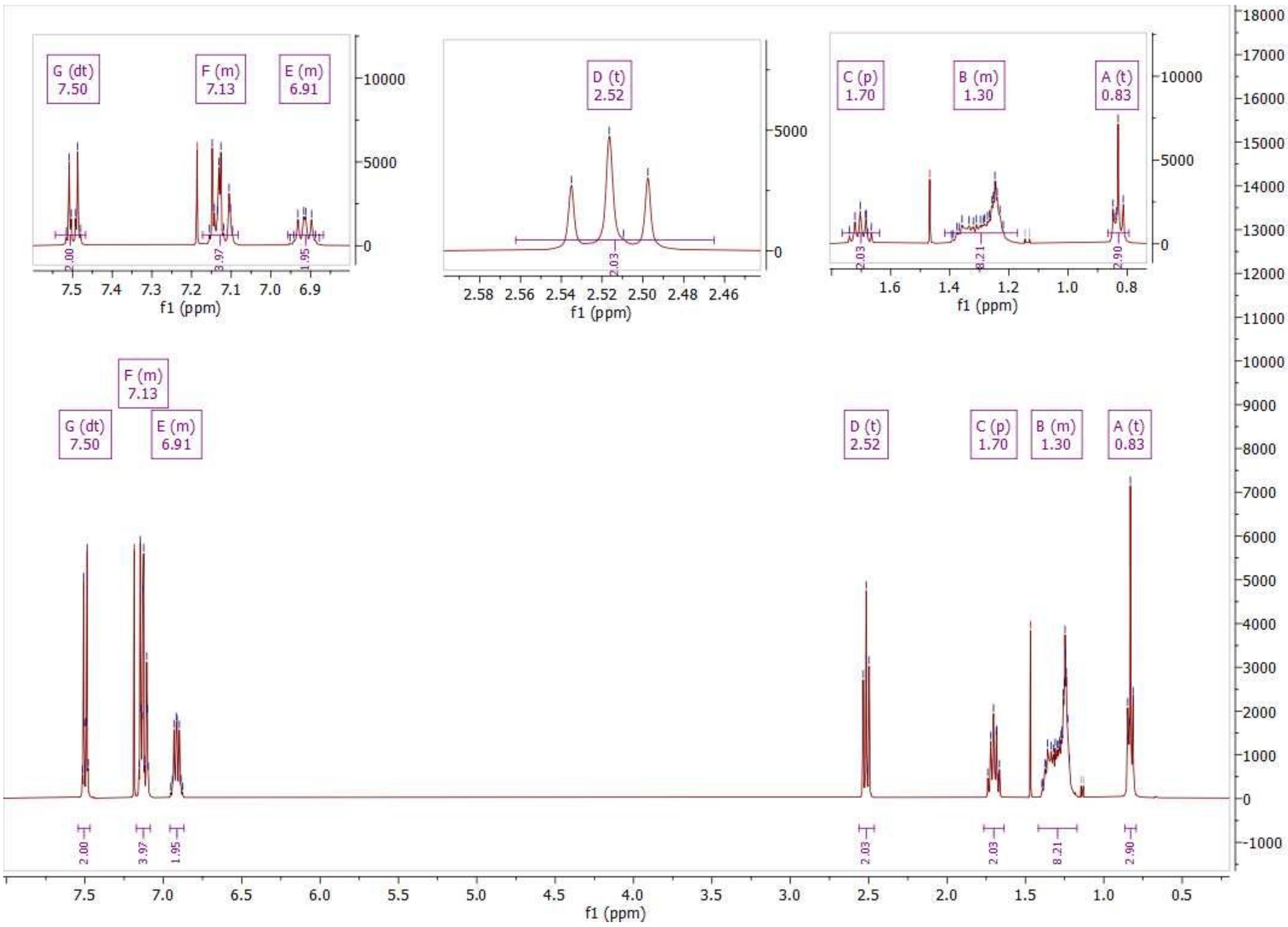


**Figure SI-42:** $^{1}H$ NMR for IDX1646

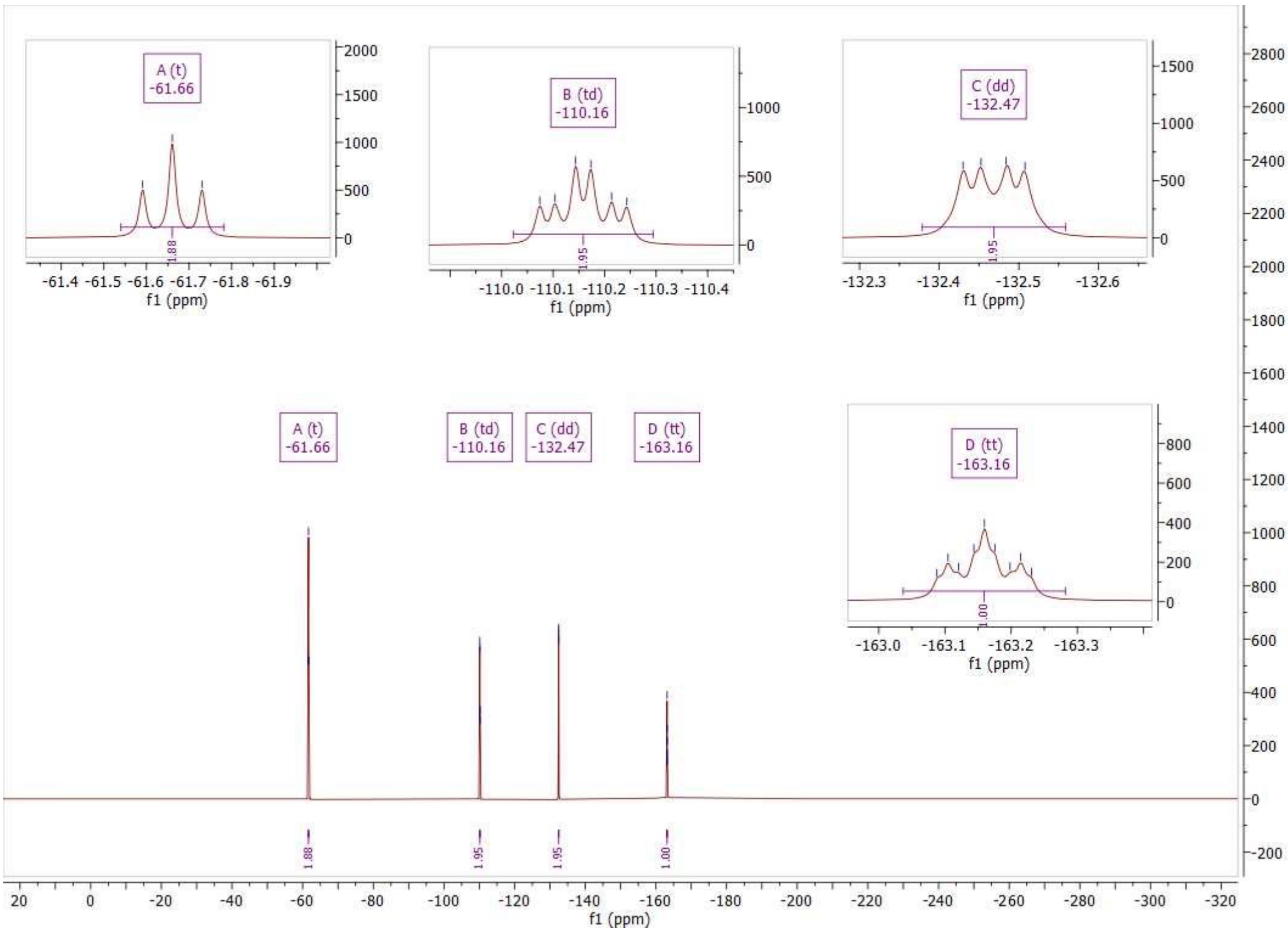


**Figure SI-43:** $^{19}F$ NMR for IDX1646

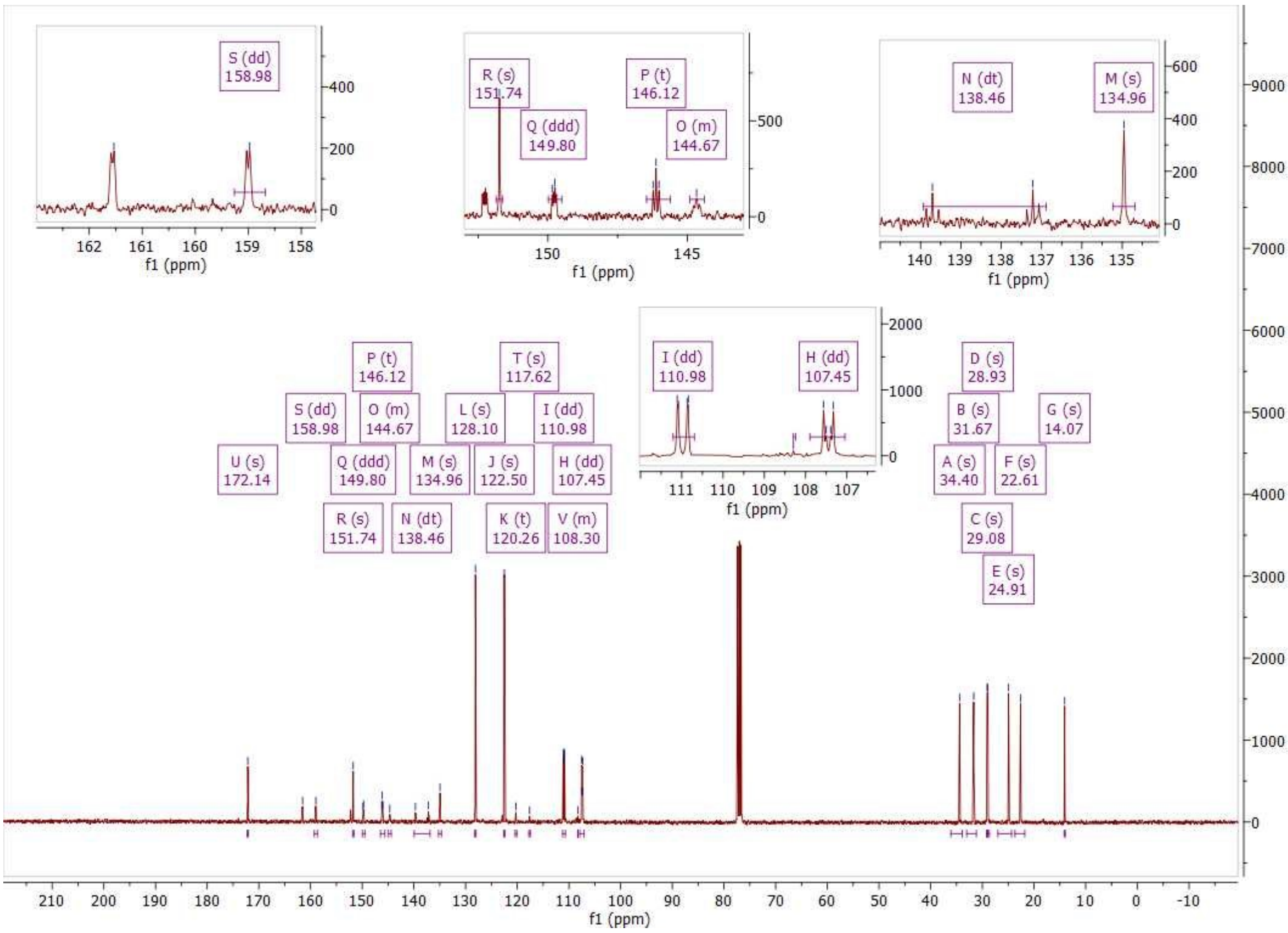


**Figure SI-44:** $^{13}C\{^{1}H\}$ NMR for IDX1646

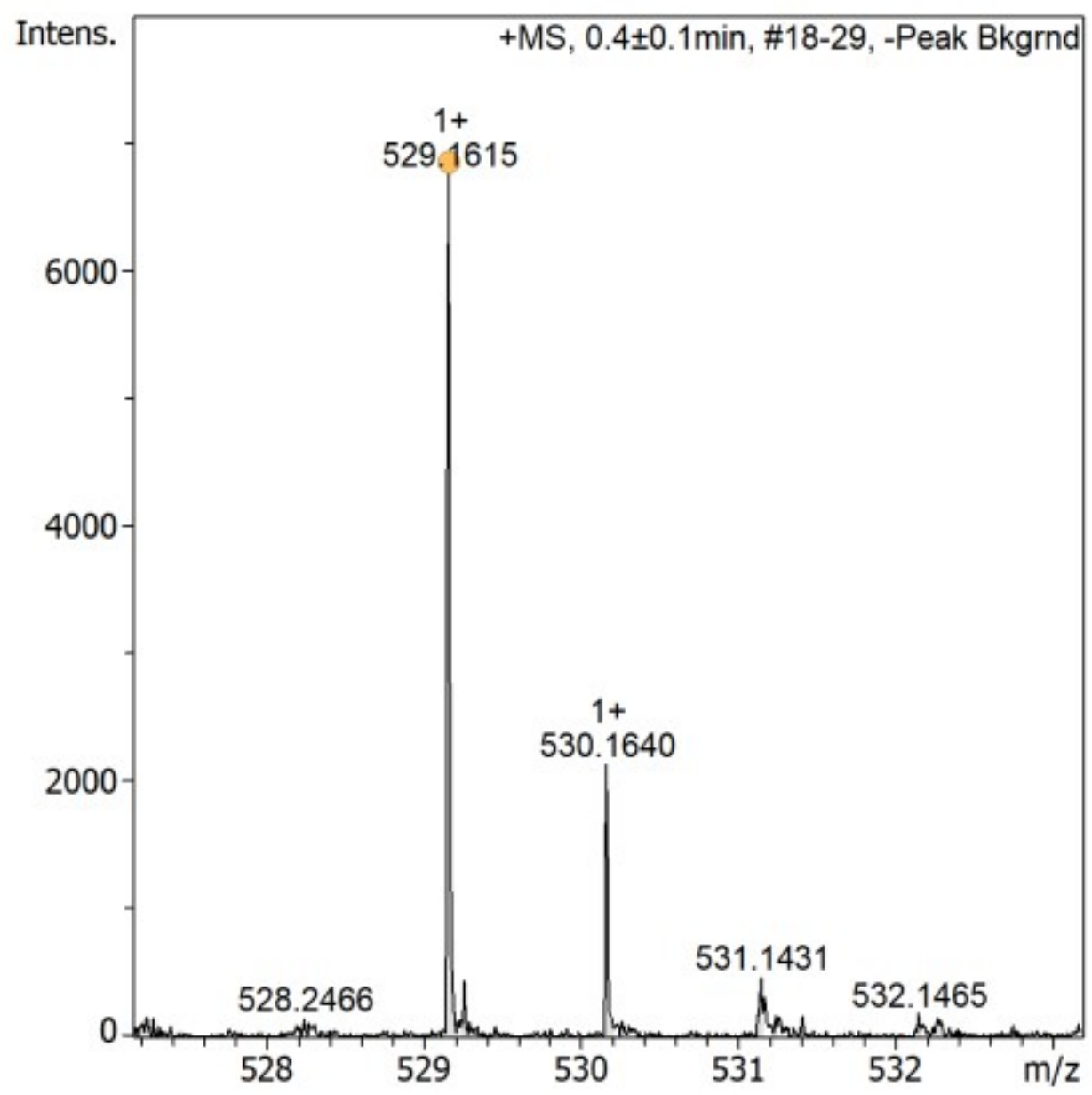


**Figure SI-45:** HRMS for IDX1646

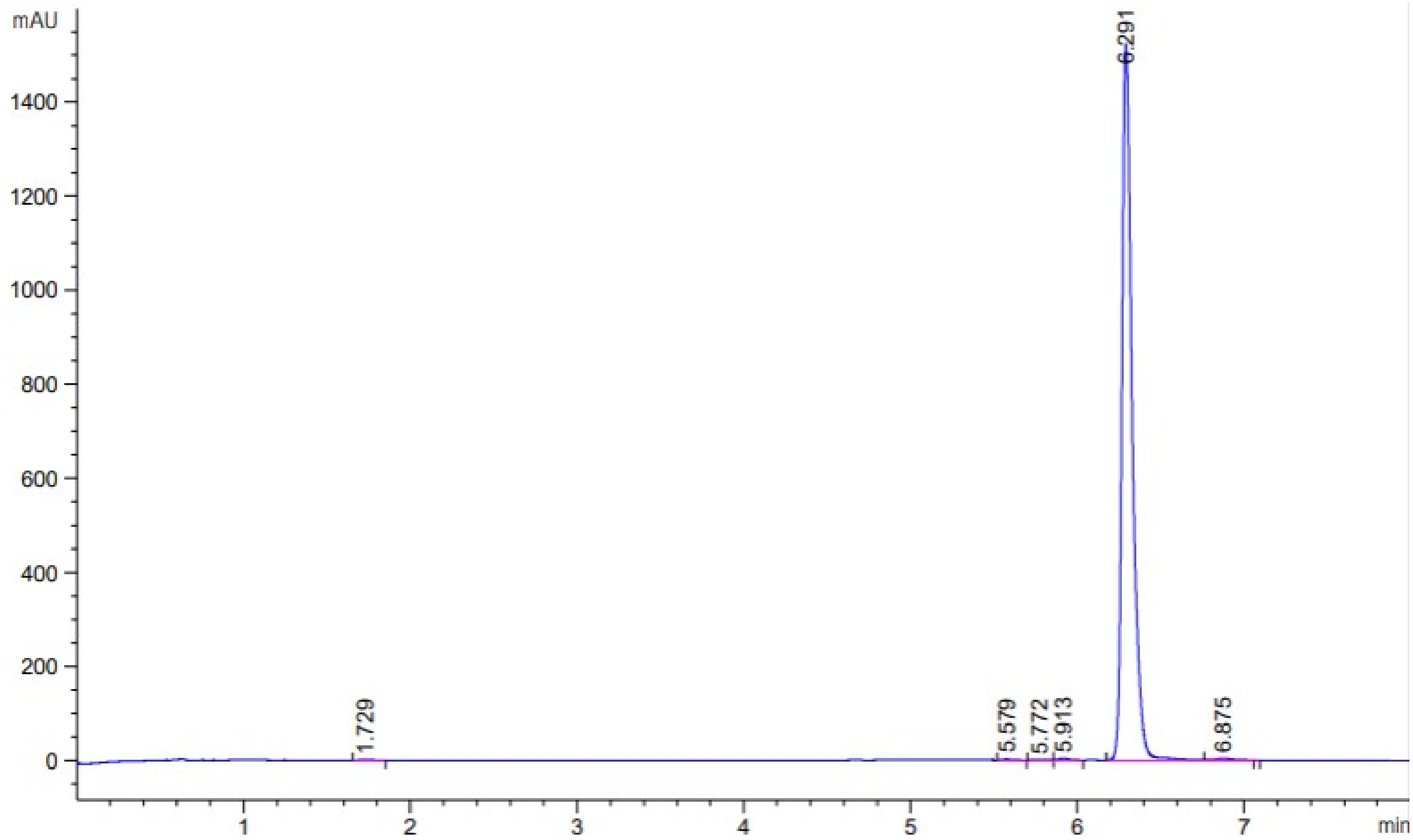


**Figure SI-46:** RP-HPLC for IDX1646

## 10.3. Analytical data for Scheme 3

### 10.3.1. IDX323 / CJG 412 / 2-bromo-5-ethoxy-1,3-diflurobenzene

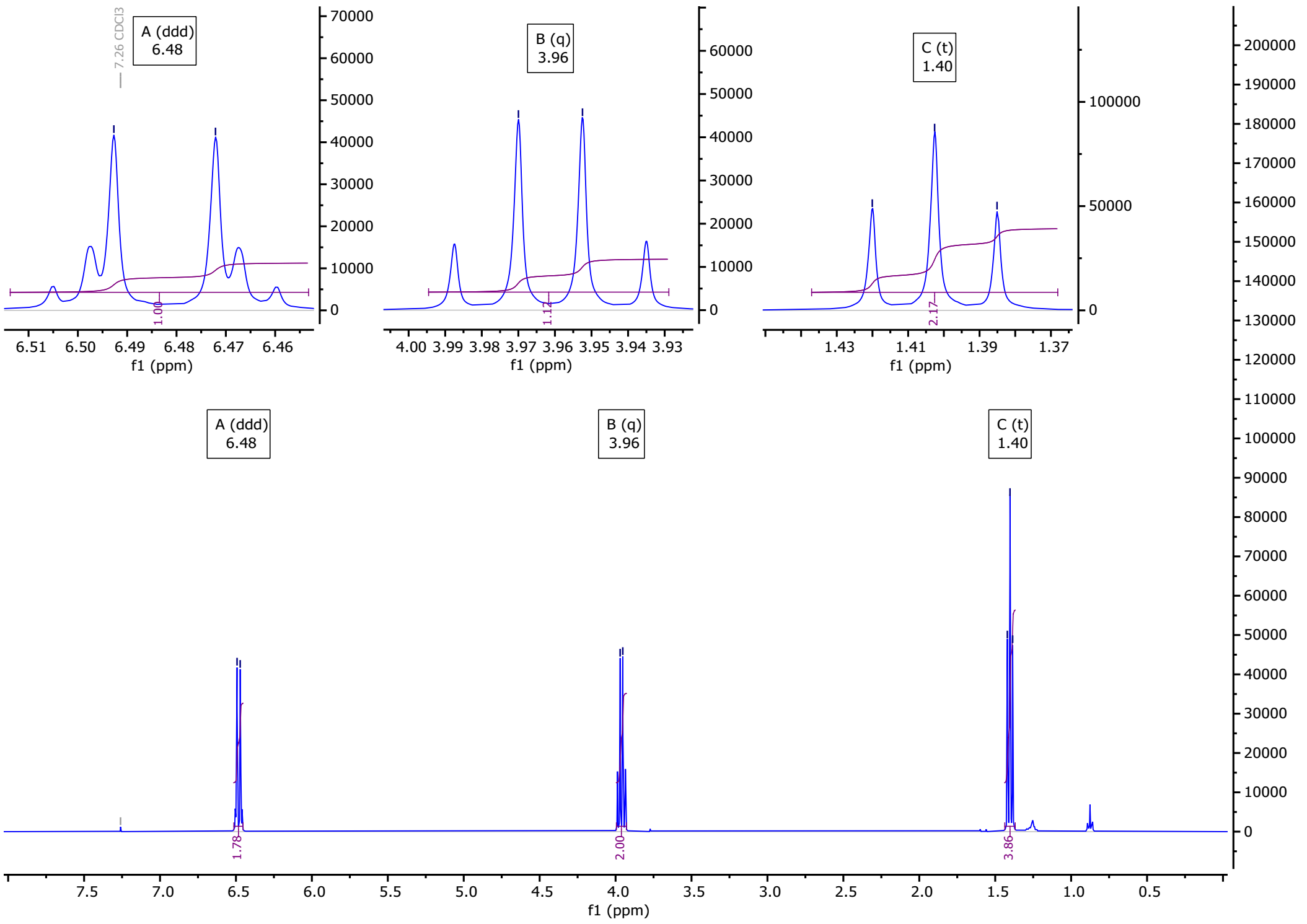


**Figure SI-47:** $^{1}$H NMR of 2-bromo-5-ethoxy-1,3-diflurobenzene

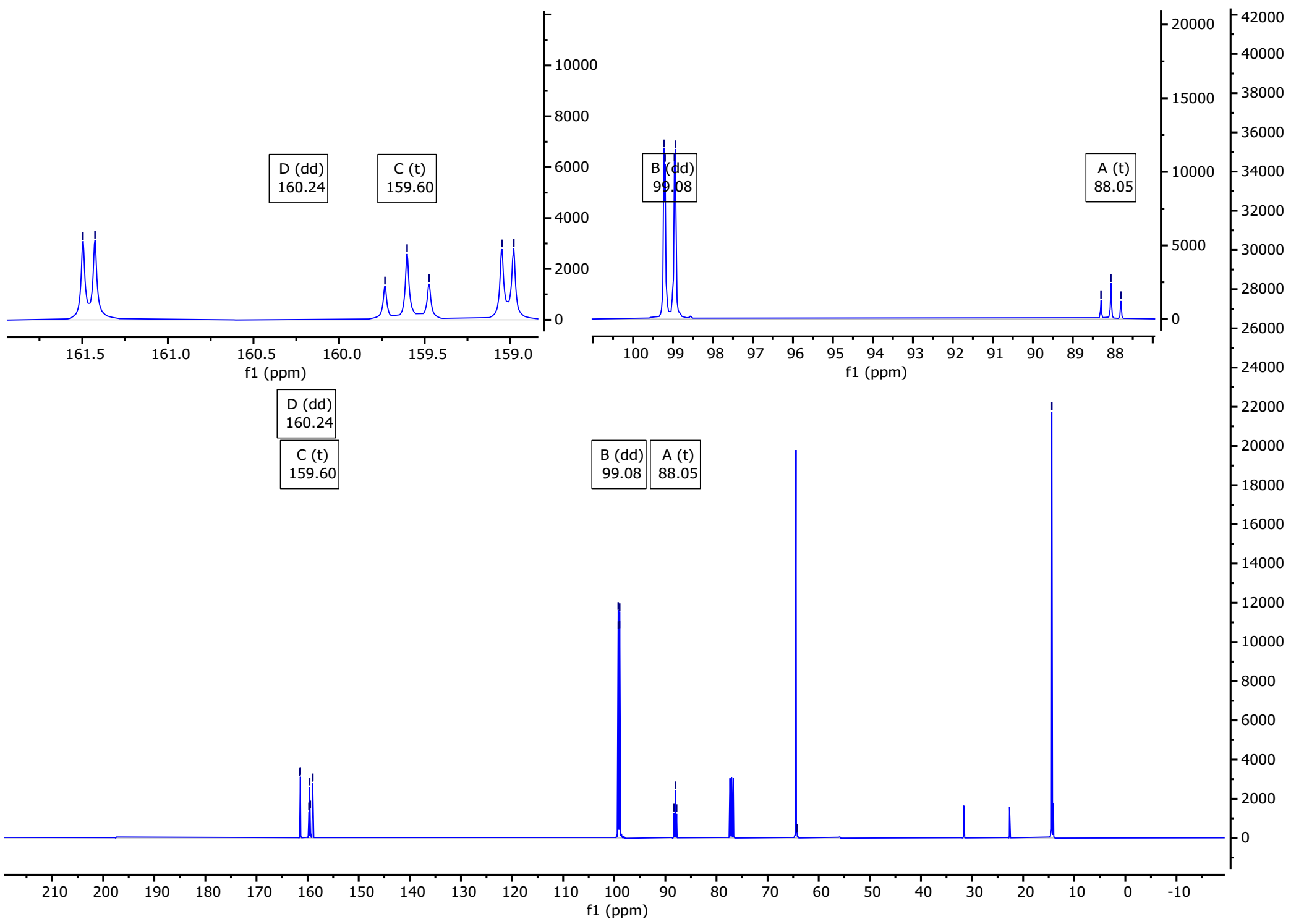


**Figure SI-48:** $^{13}$C{$^{1}$H} NMR of 2-bromo-5-ethoxy-1,3-diflurobenzene

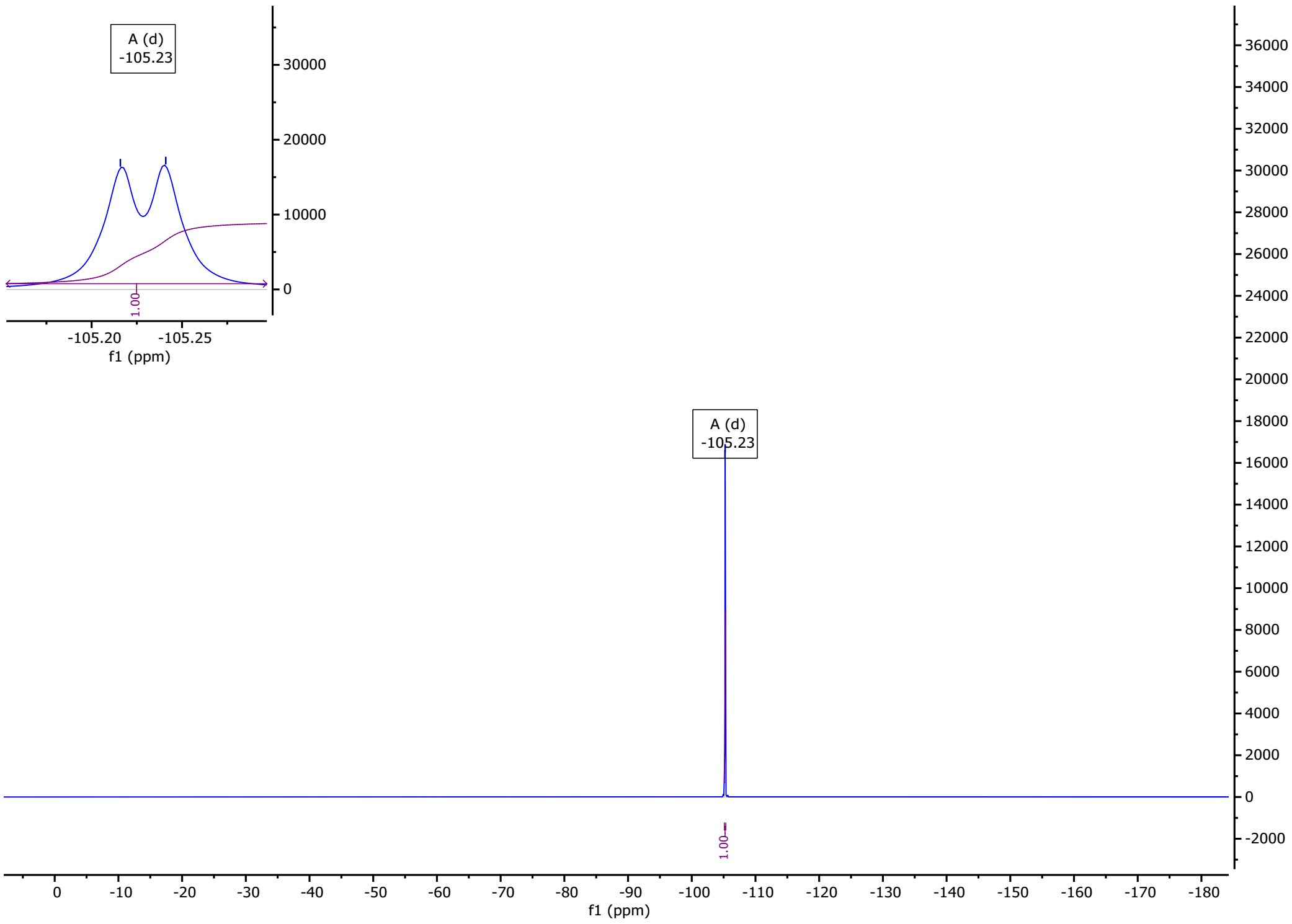


**Figure SI-49:** $^{19}$F NMR of 2-bromo-5-ethoxy-1,3-diflurobenzene

### 10.3.2. Analytical Data for IDX??? / CJG 483 / 4-ethoxy-2,6-diflurobenzoic acid

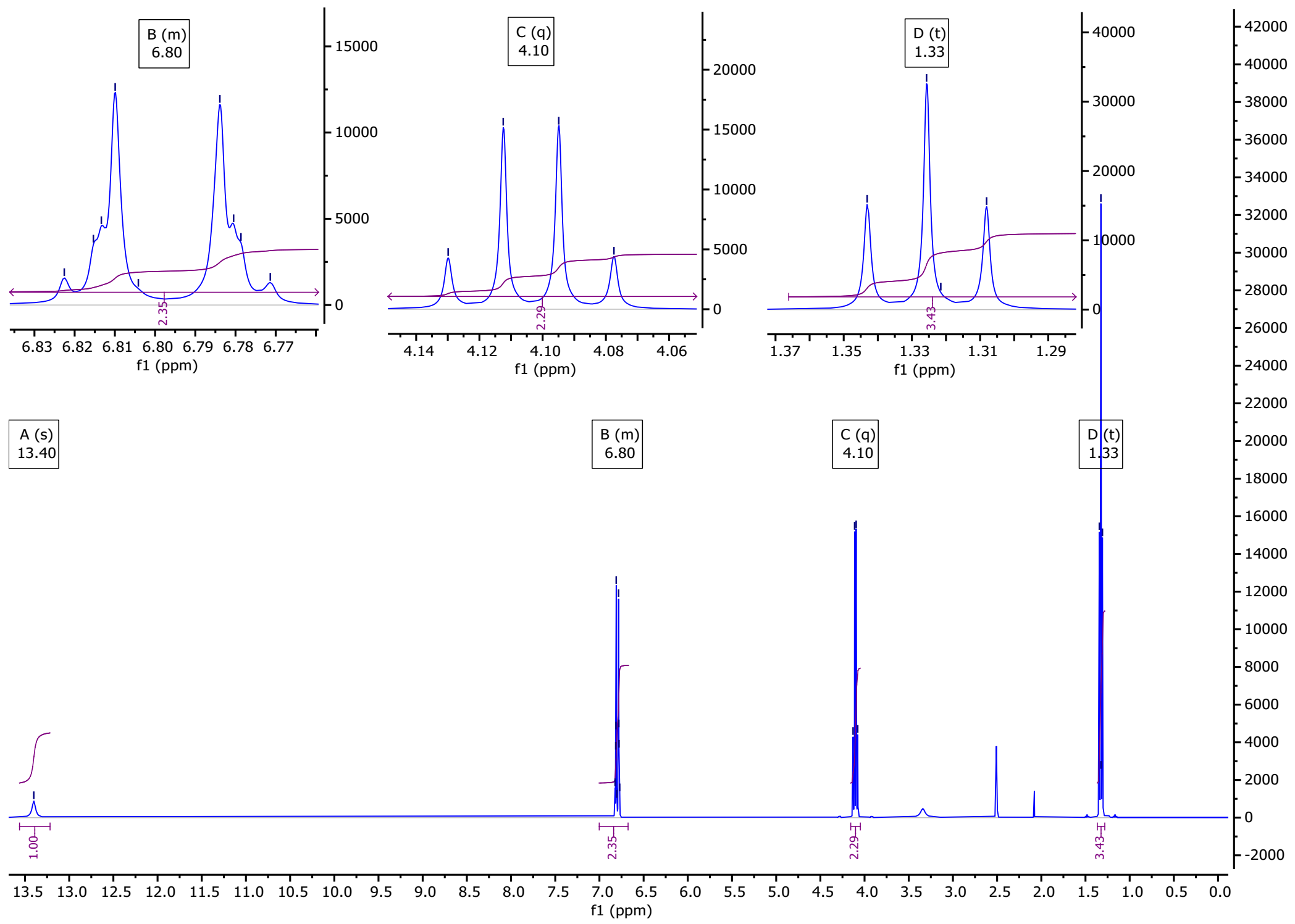


**Figure SI-50:** $^{1}H$ NMR of 4-ethoxy-2,6-diflurobenzoic acid

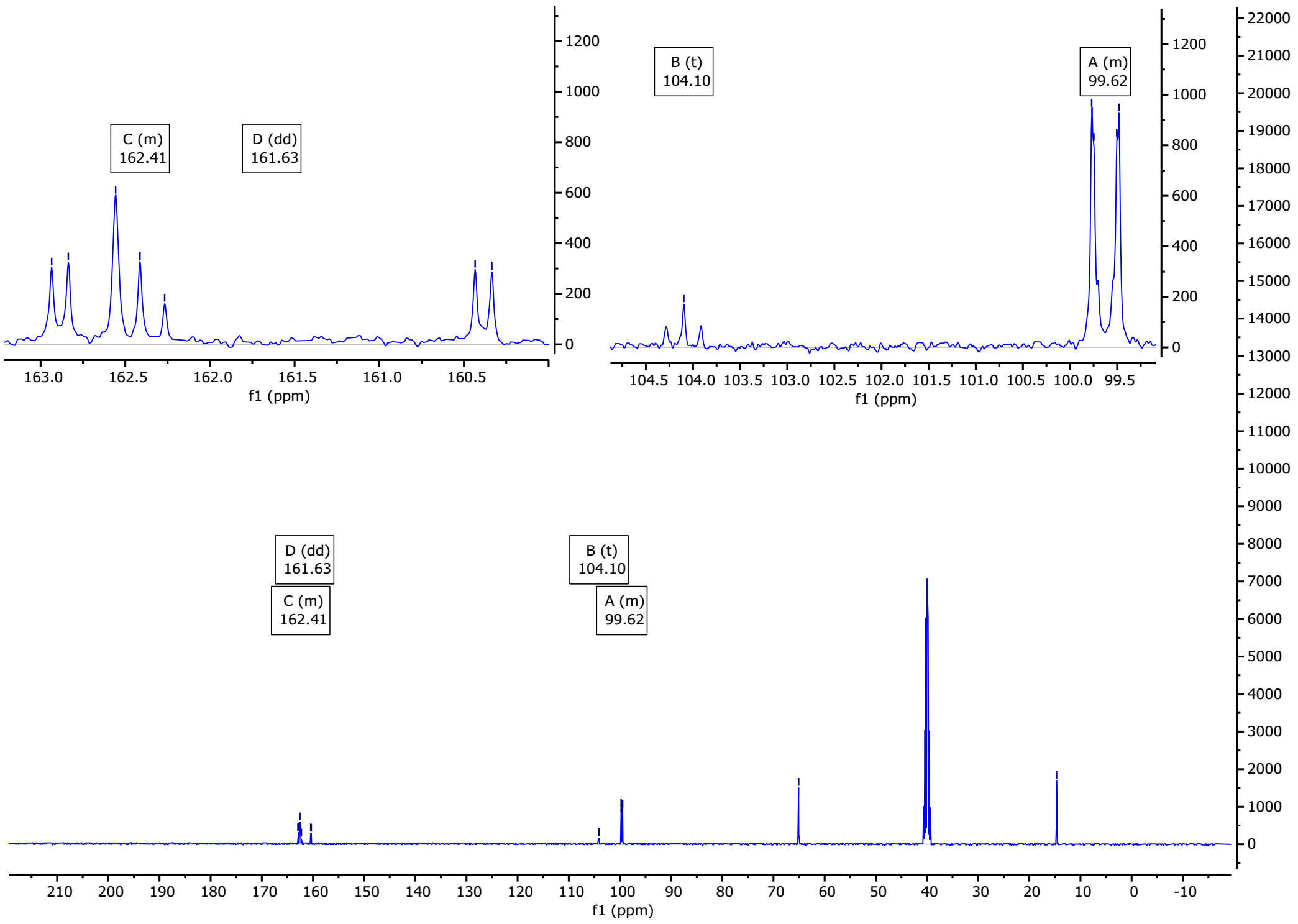


**Figure SI-51:** $^{13}C\{^{1}H\}$ NMR of 4-ethoxy-2,6-diflurobenzoic acid

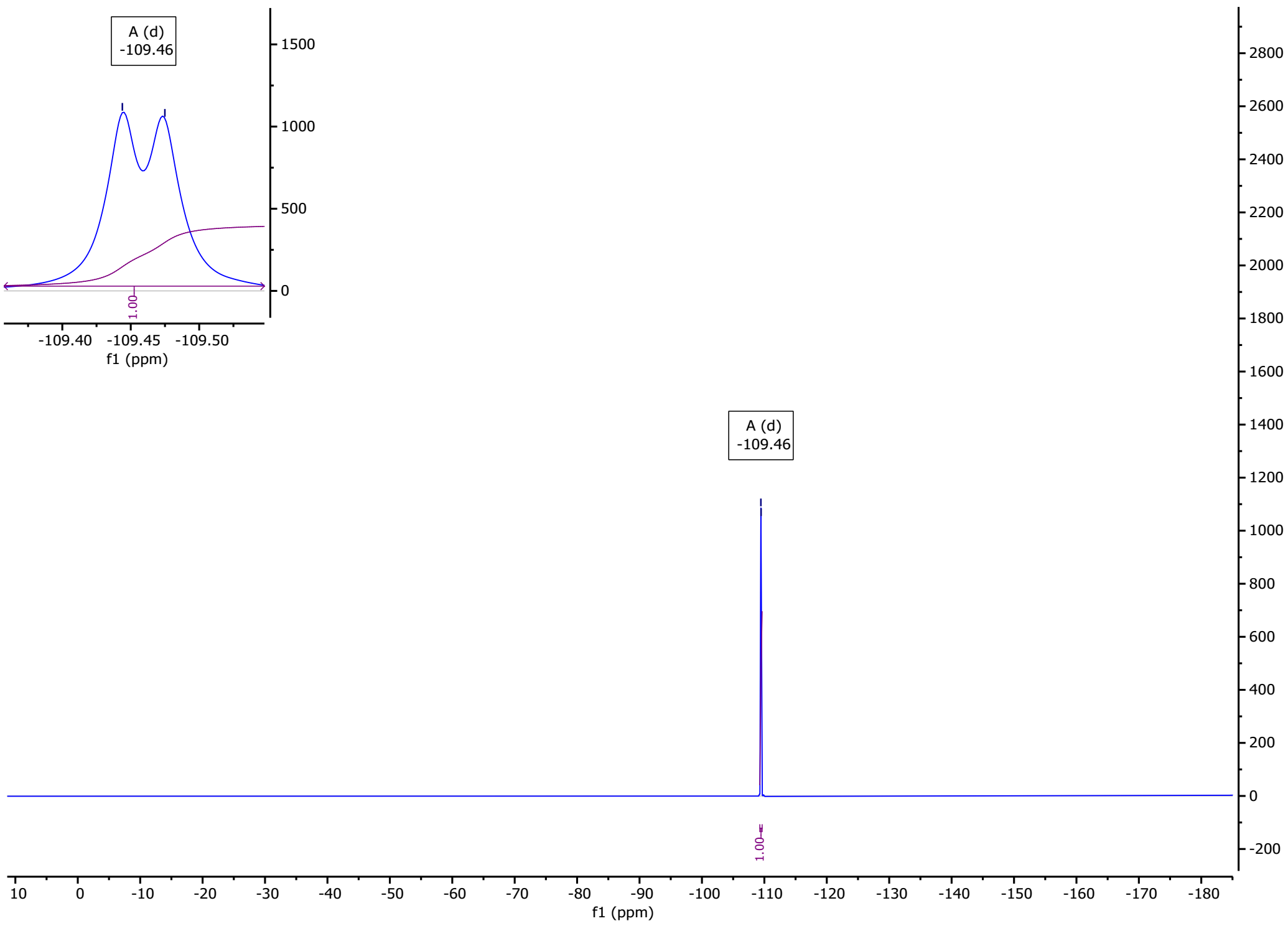


**Figure SI-52:** $^{19}F$ NMR of 4-ethoxy-2,6-diflurobenzoic acid

### 7.3.4. Analytical data for IDX 232 / CJG 484 / 2,3',4',5'-tetrafluoro-[1,1'-biphenyl]-4-yl 4-ethoxy-2,6-difluorobenzoate:

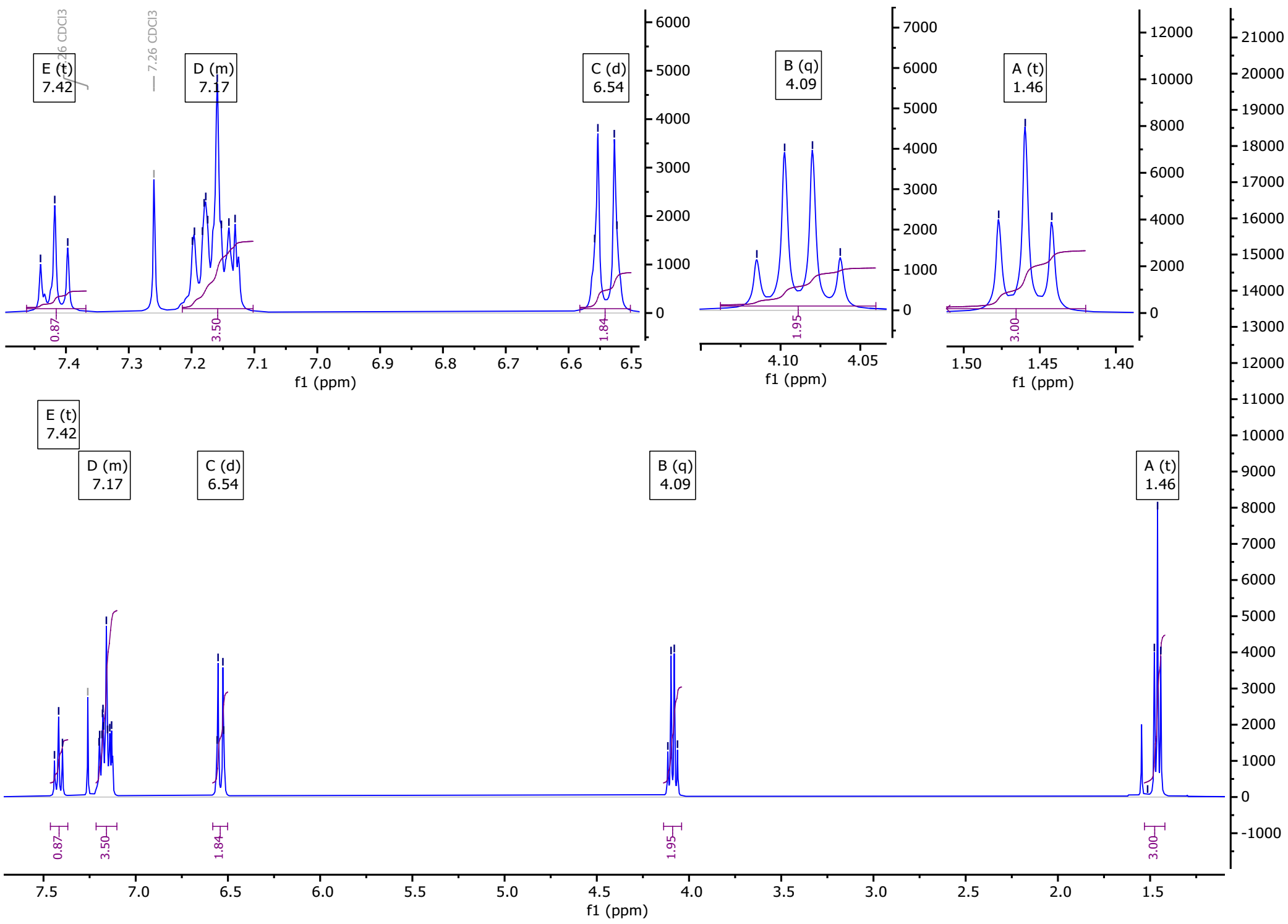

**Figure SI-53:** $^{1}H$ NMR for 2,3',4',5'-tetrafluoro-[1,1'-biphenyl]-4-yl 4-ethoxy-2,6-difluorobenzoate

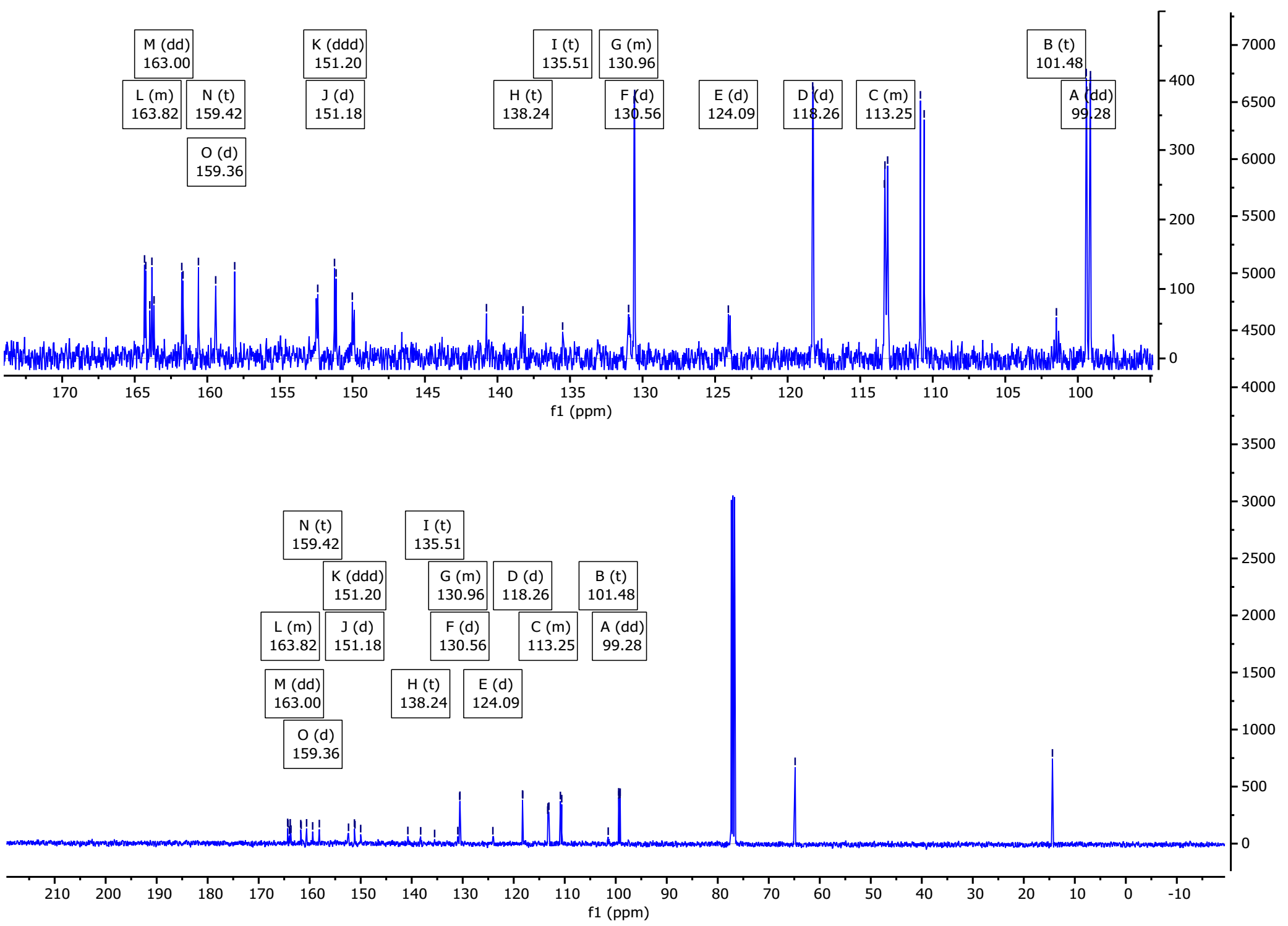

**Figure SI-54:** $^{13}C\{^{1}H\}$ NMR for 2,3',4',5'-tetrafluoro-[1,1'-biphenyl]-4-yl 4-ethoxy-2,6-difluorobenzoate

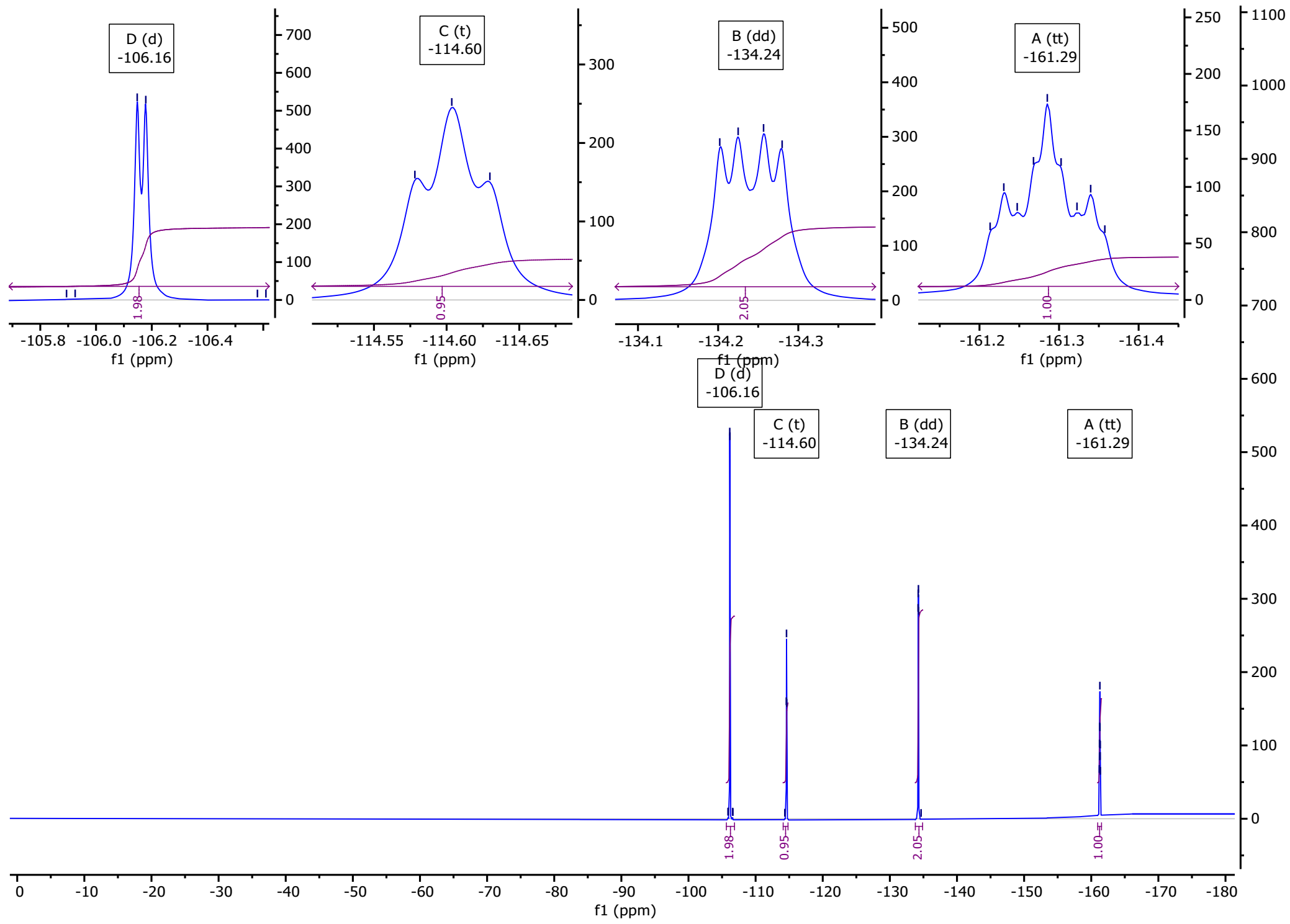


**Figure SI-55:** $^{19}F$ NMR 2,3',4',5'-tetrafluoro-[1,1'-biphenyl]-4-yl 4-ethoxy-2,6-difluorobenzoate

## 10.4. Scheme 4

### 10.4.1. 3,5-difluorobenzyl butyl ether

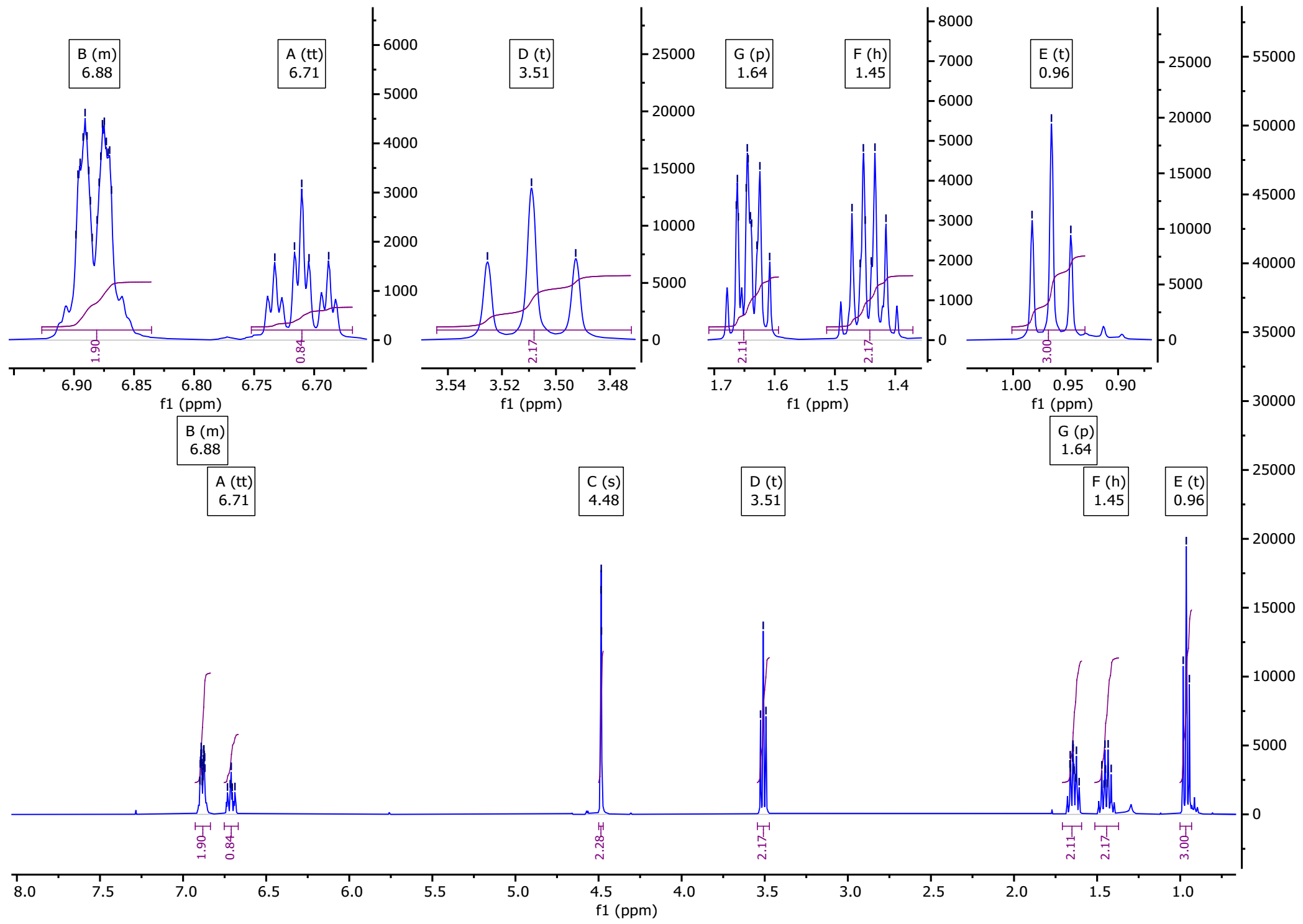

**Figure SI-56:** $^{1}H$ NMR for 3,5-difluorobenzyl butyl ether

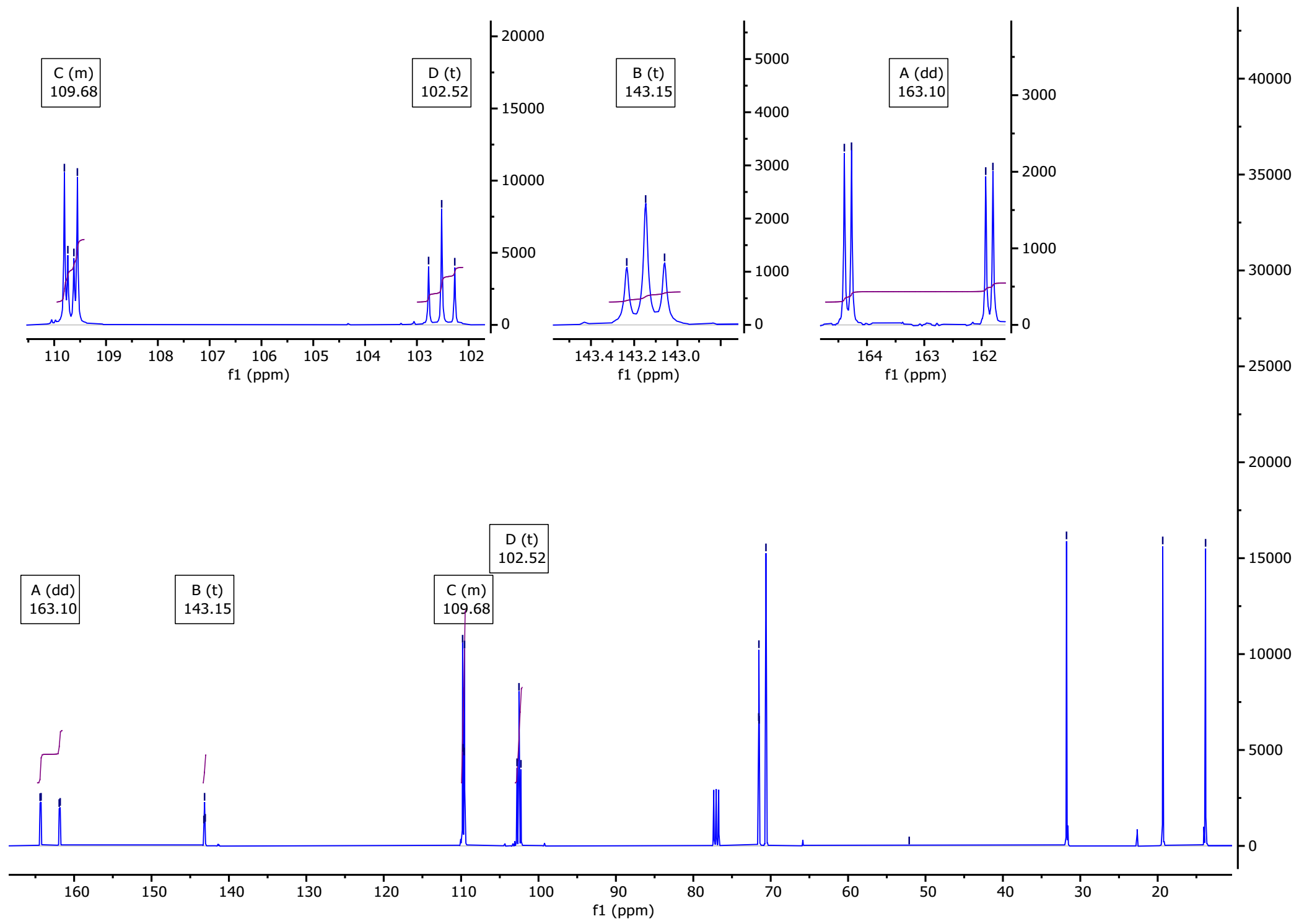

**Figure SI-57:** $^{13}C\{^{1}H\}$ NMR for 3,5-difluorobenzyl butyl ether

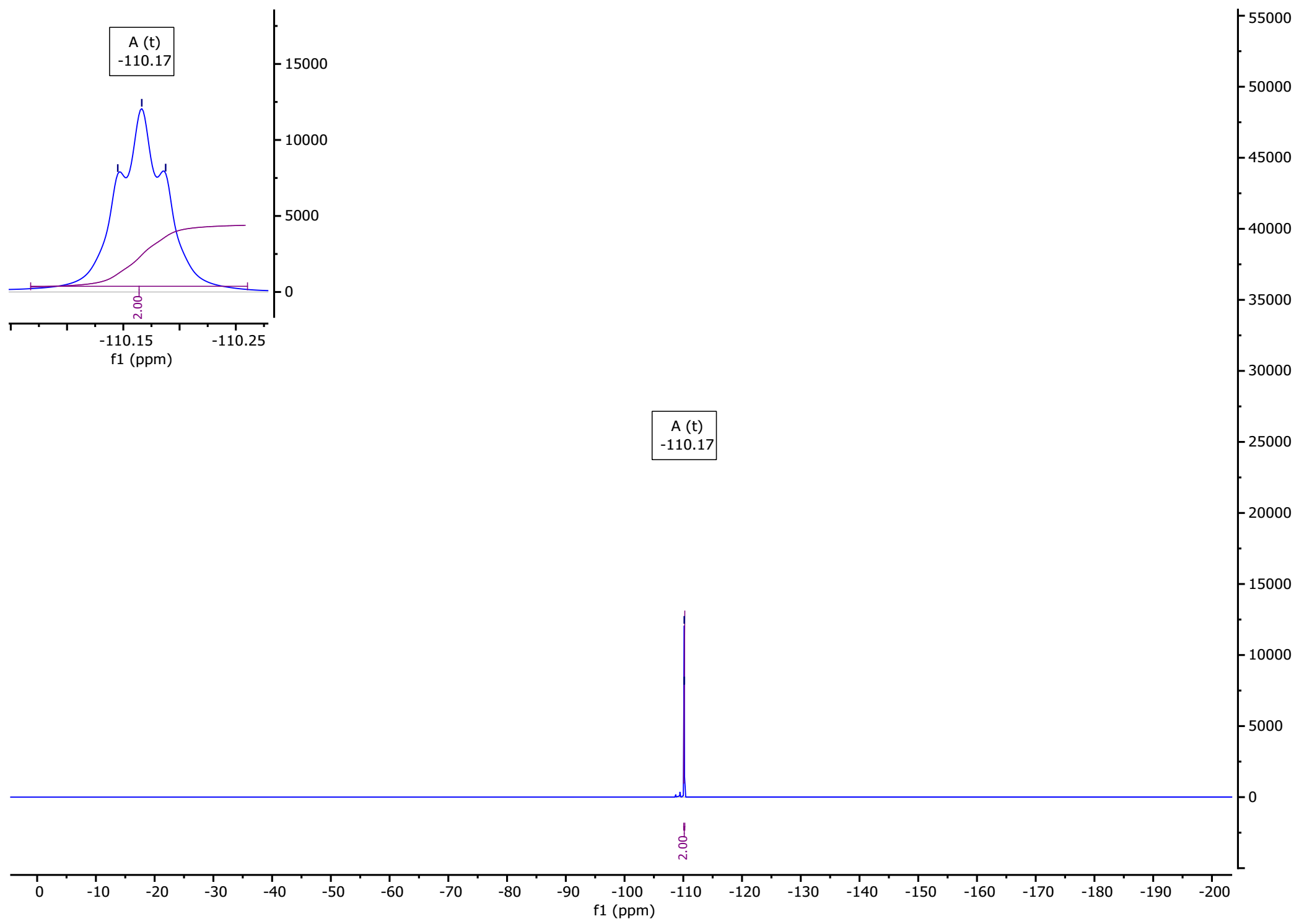


**Figure SI-58:** $^{19}F$ NMR for 3,5-difluorobenzyl butyl ether

### 10.4.2. 2,6-difluoro-2-(butyl-1-yloxy)benzoic acid

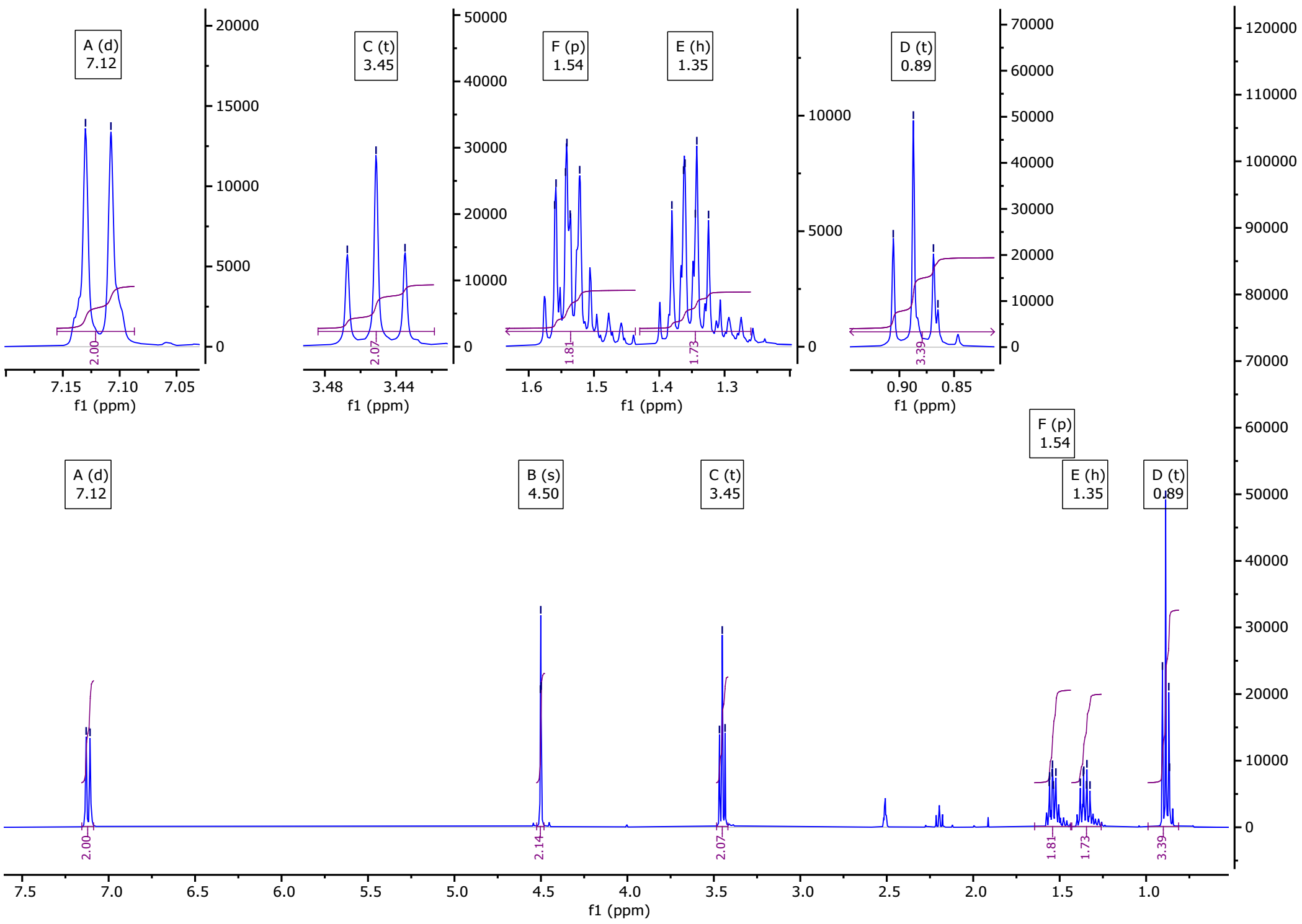

**Figure SI-59:** $^{1}H$ NMR for 2,6-difluoro-2-(butyl-1-yloxy)benzoic acid

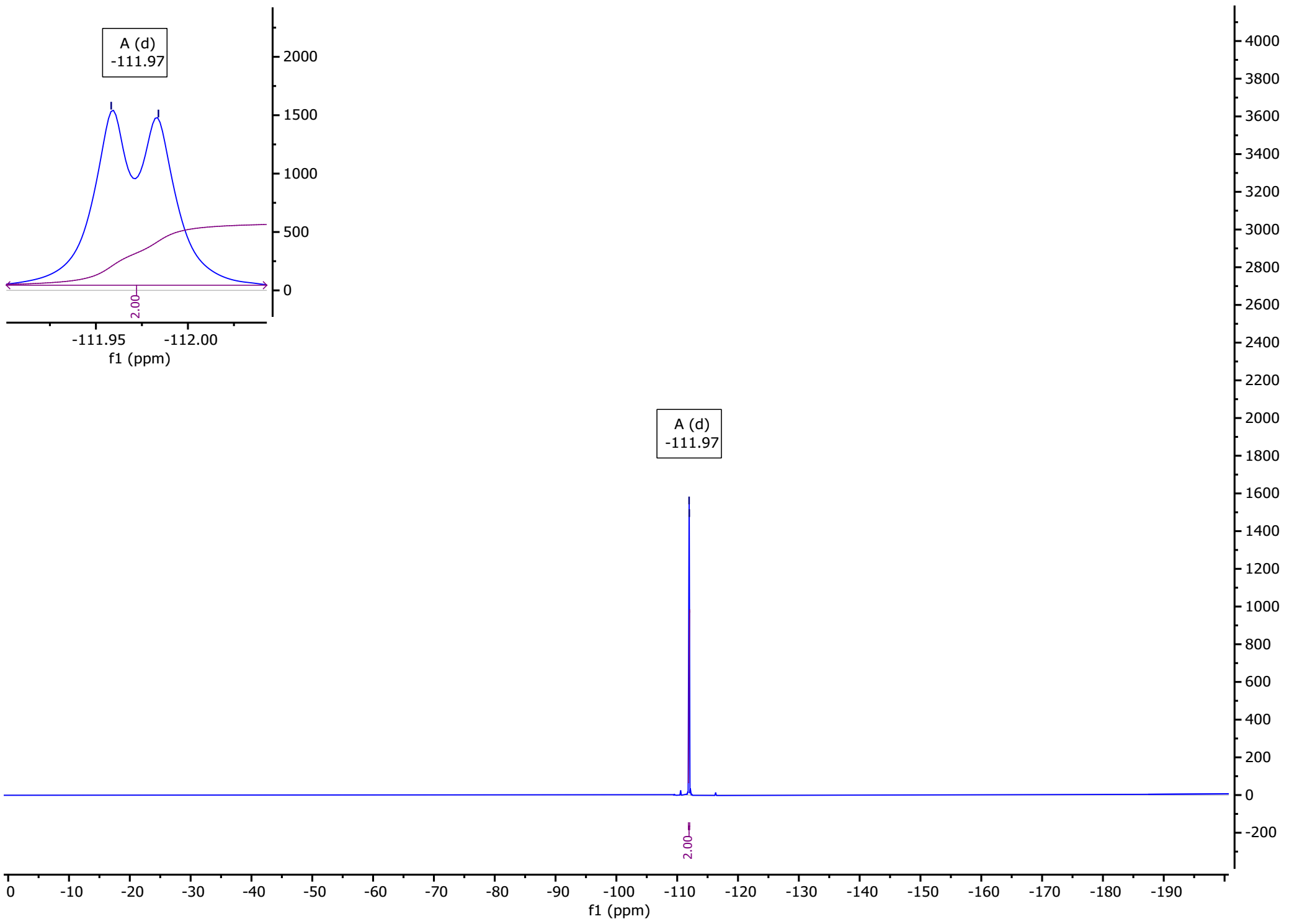

**Figure SI-60:** $^{19}F$ NMR for 2,6-difluoro-2-(butyl-1-yloxy)benzoic acid

### 7.4.3. IDX 59 / CJG 480 / 2,3',4',5',6-pentafluoro-[1,1'-biphenyl]-4-yl 4-(butoxymethyl)-2,6-difluorobenzoate

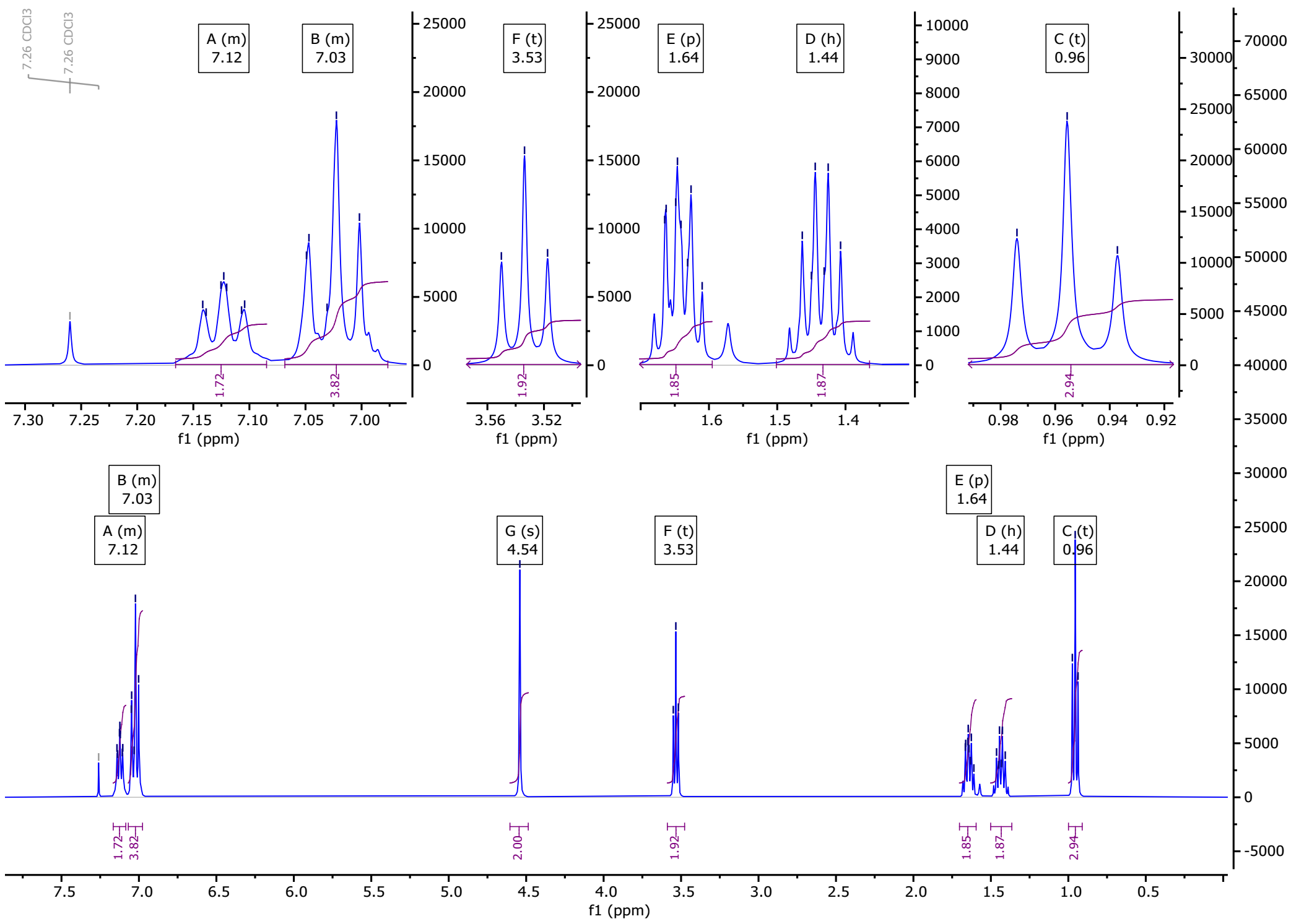


**Figure SI-61:** $^{1}$H NMR of 2,3',4',5',6-pentafluoro-[1,1'-biphenyl]-4-yl 4-(butoxymethyl)-2,6-difluorobenzoate.

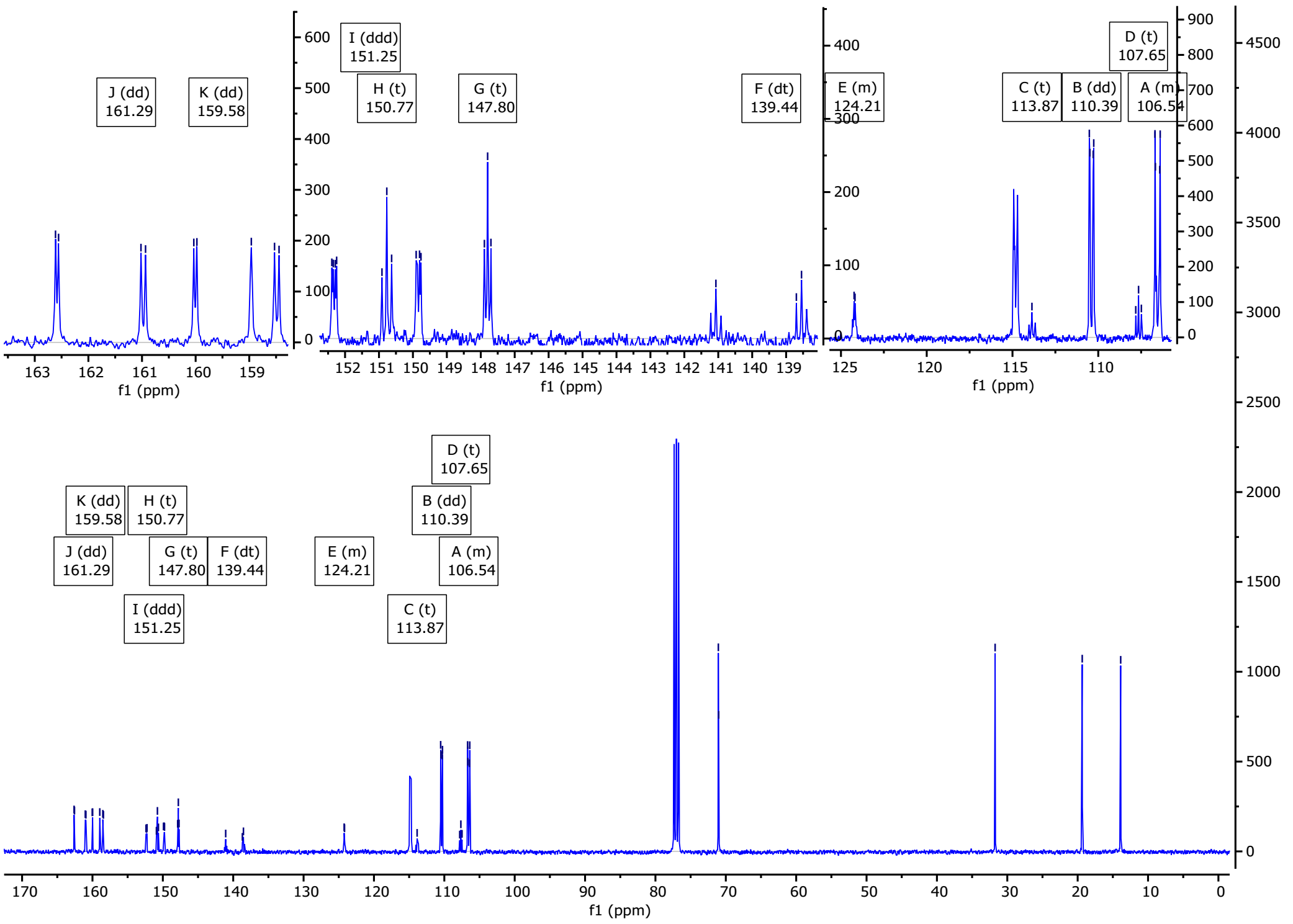


**Figure SI-62:** $^{13}C\{^{1}H\}$ NMR of 2,3',4',5',6-pentafluoro-[1,1'-biphenyl]-4-yl 4-(butoxymethyl)-2,6-difluorobenzoate.

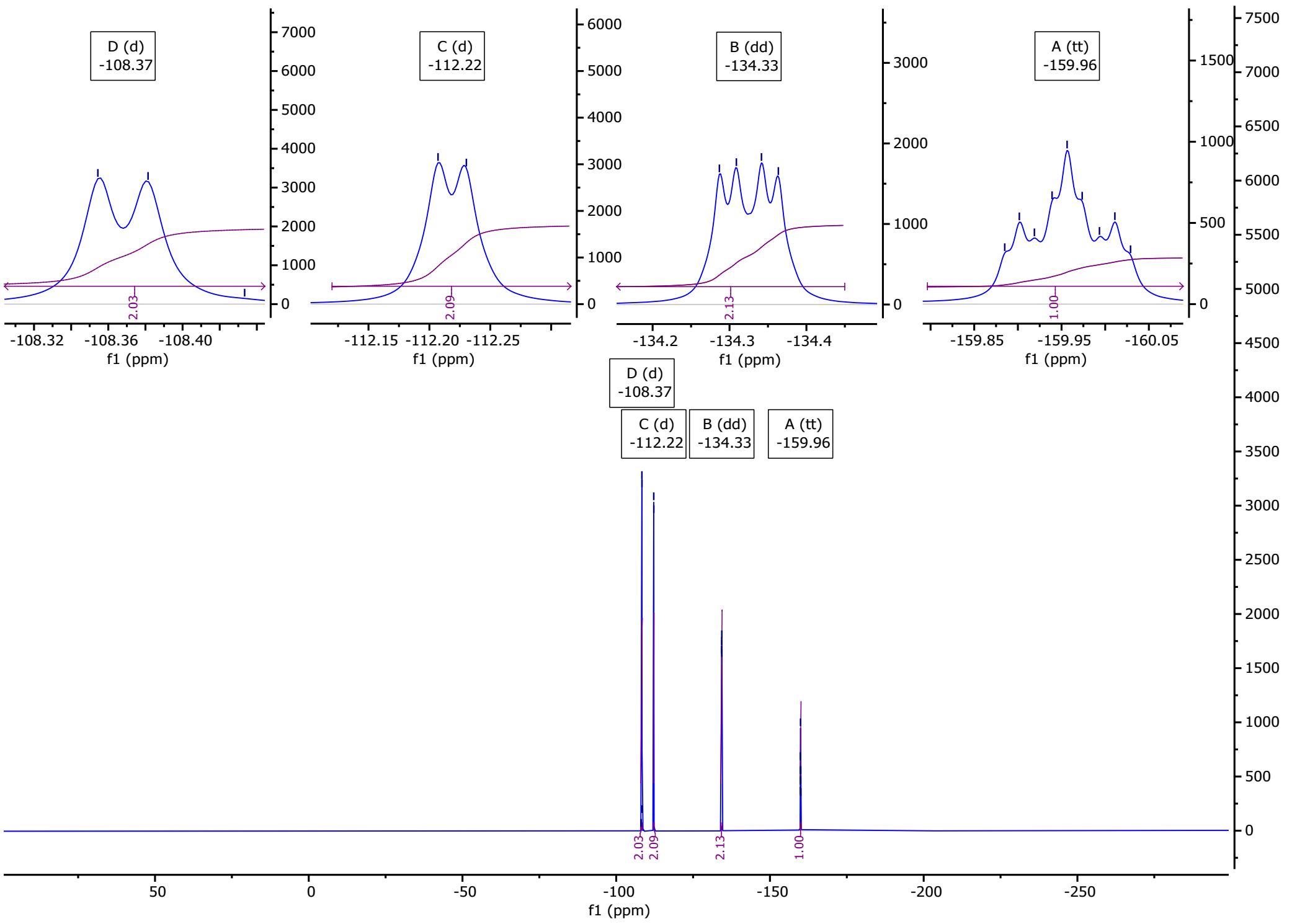


**Figure SI-63:** $^{19}F$ NMR of 2,3',4',5',6-pentafluoro-[1,1'-biphenyl]-4-yl 4-(butoxymethyl)-2,6-difluorobenzoate.

## 10.5. Scheme 5

### 10.5.1. IDX 519 / CJG 410 / 2,3',4',5',6-pentafluoro-[1,1'-biphenyl]-4-yl 2-fluoro-4-(5-propyl-1,3-dioxan-2-yl)benzoate

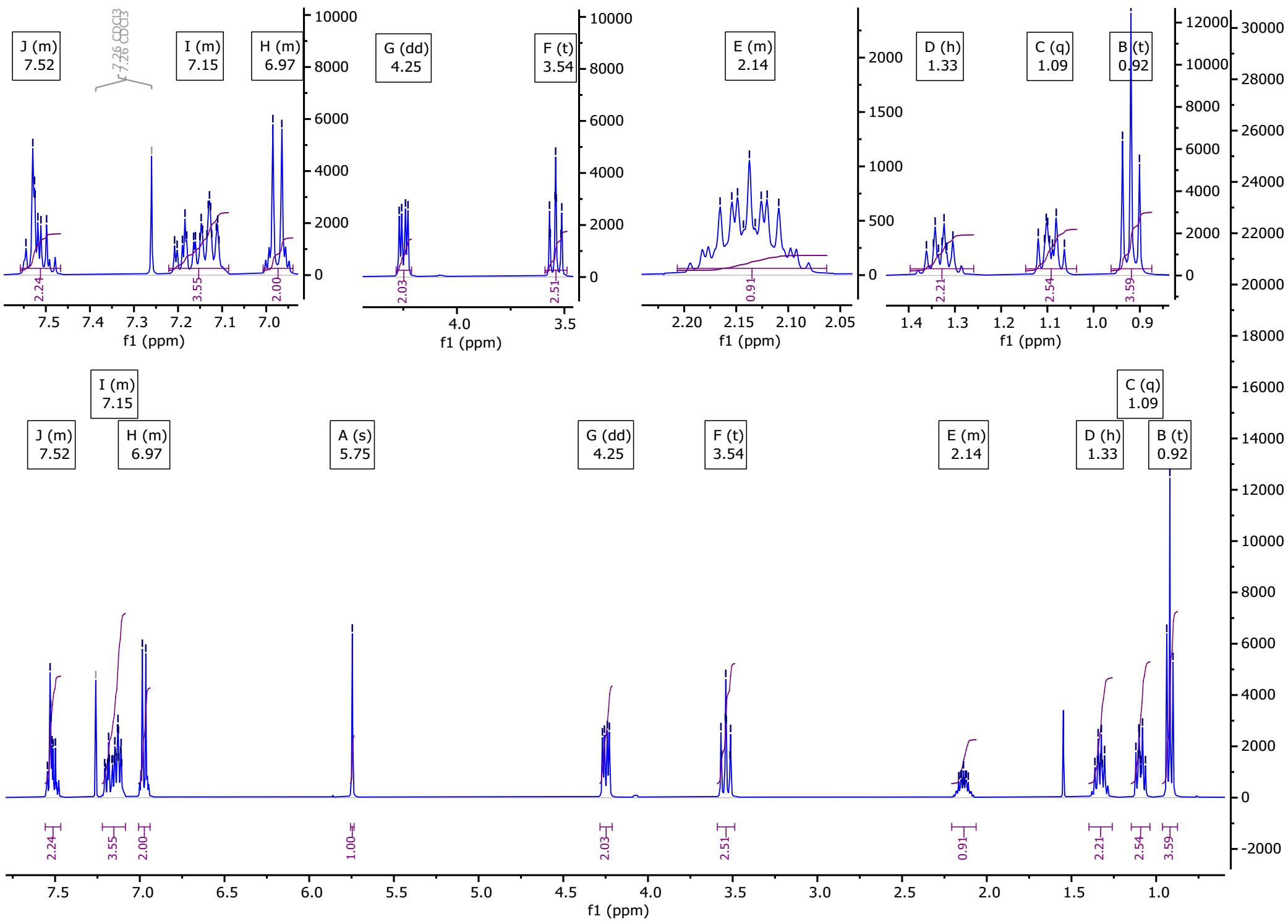


**Figure SI-64:** $^{1}H$ NMR of 2,3',4',5',6-pentafluoro-[1,1'-biphenyl]-4-yl 2-fluoro-4-(5-propyl-1,3-dioxan-2-yl)benzoate

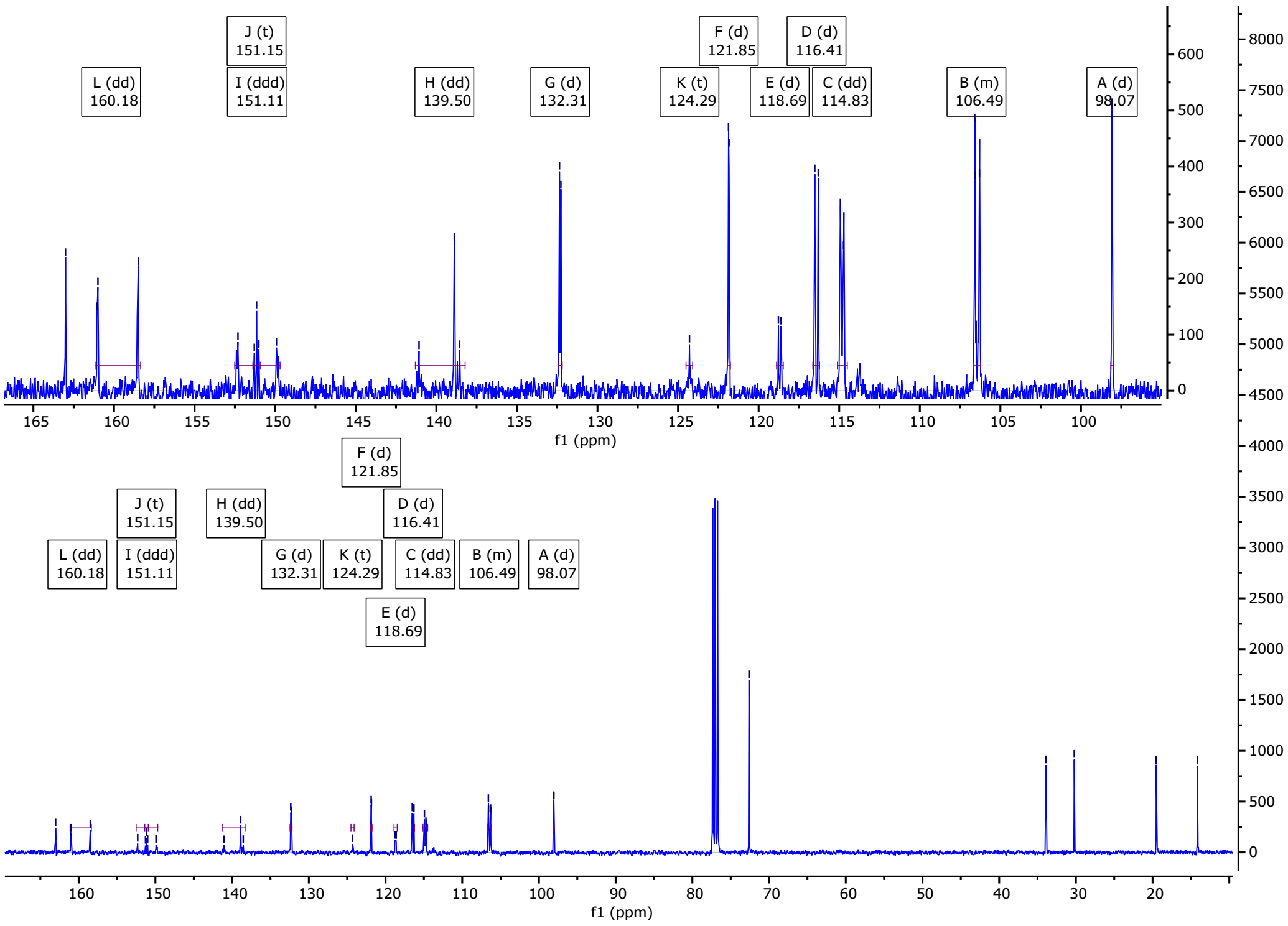


**Figure SI-65:** $^{13}C\{^{1}H\}$ NMR of 2,3',4',5',6-pentafluoro-[1,1'-biphenyl]-4-yl 2-fluoro-4-(5-propyl-1,3-dioxan-2-yl)benzoate

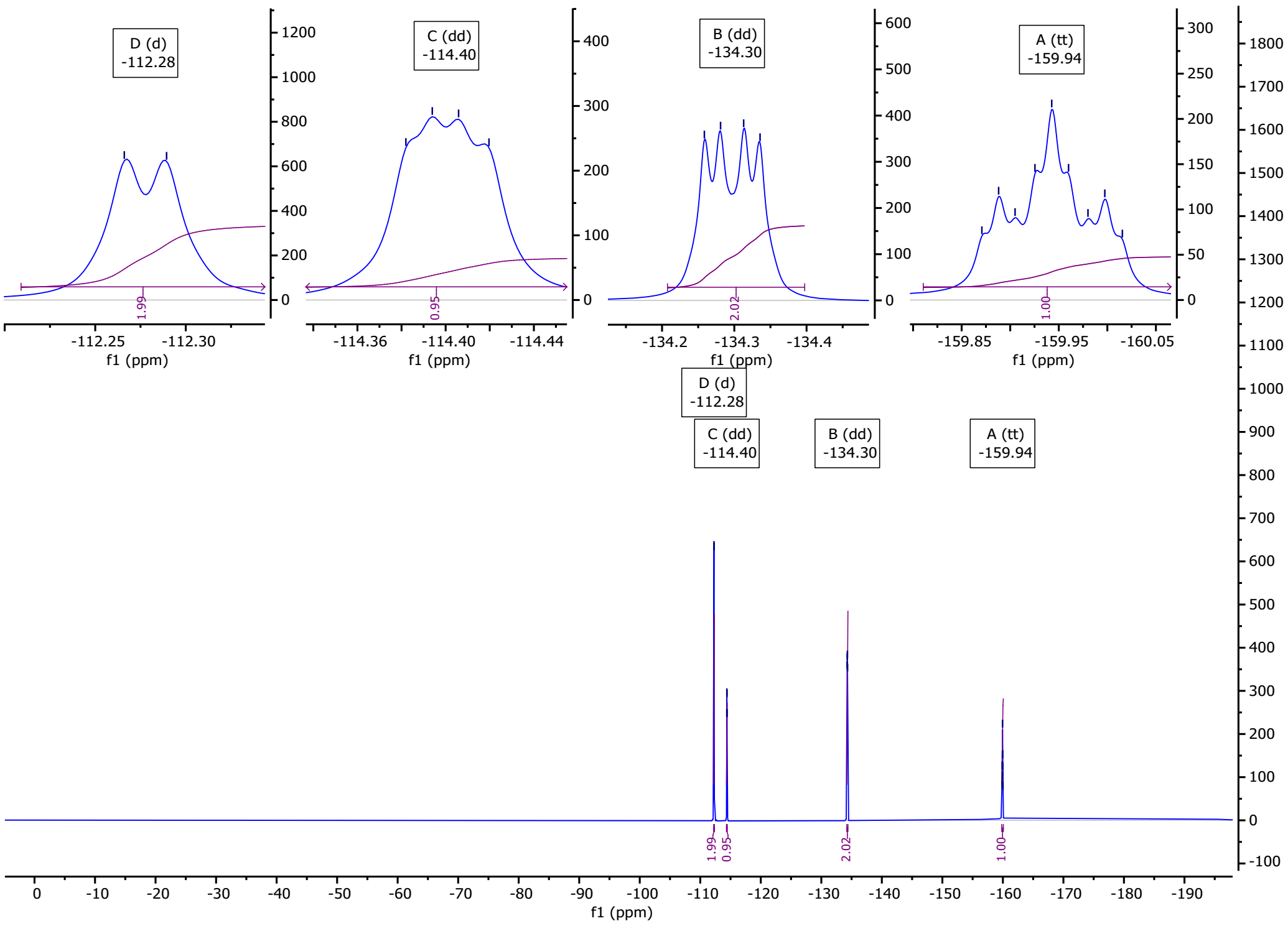


**Figure SI-66:** $^{19}F$ NMR of 2,3',4',5',6-pentafluoro-[1,1'-biphenyl]-4-yl 2-fluoro-4-(5-propyl-1,3-dioxan-2-yl)benzoate

## 10.6. Scheme 6

### 10.6.1. 4-(2,6-Difluoro-4-hydroxyphenyl)-3,4,5-trifluorobenzene

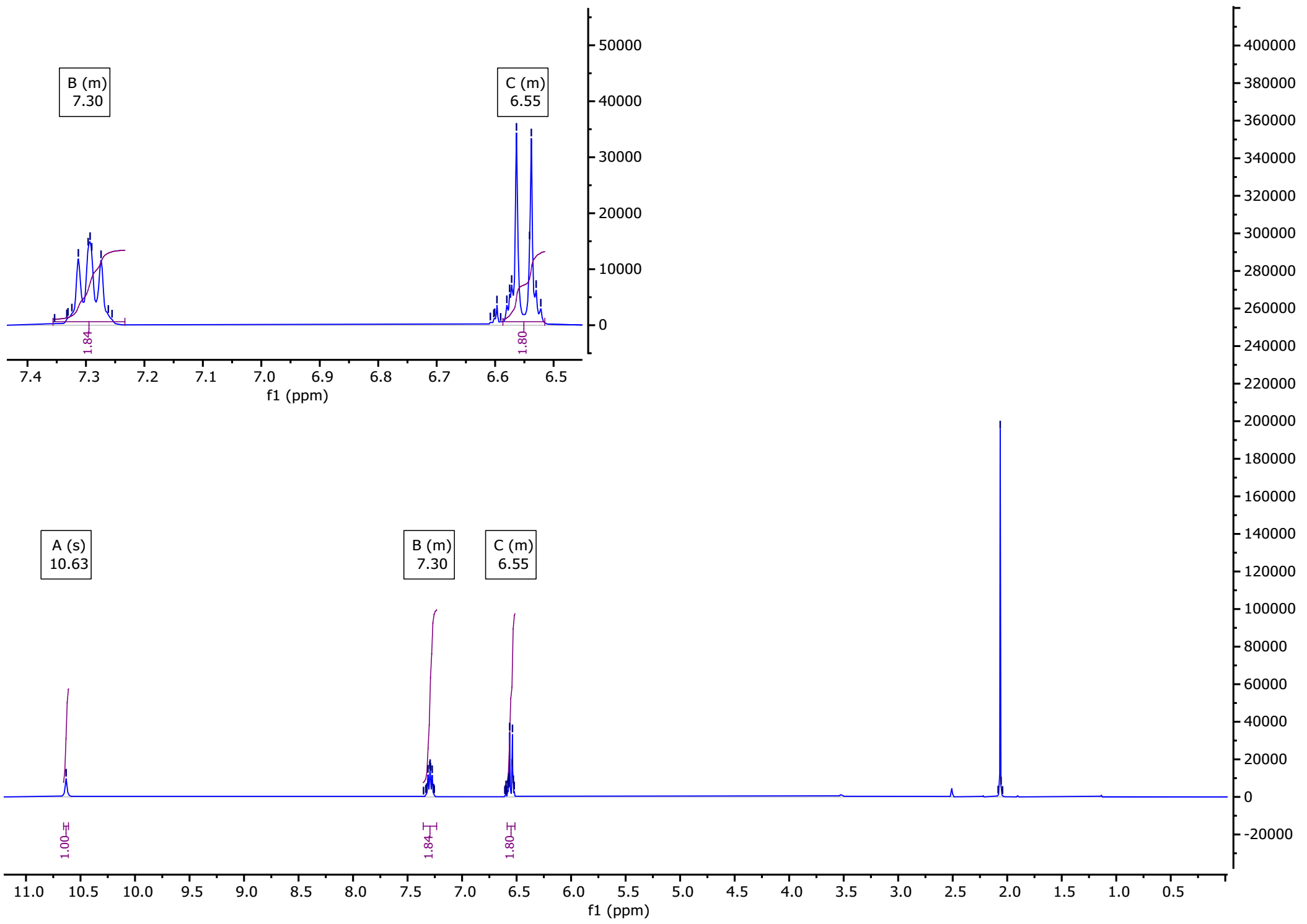


**Figure SI-67:** $^1$H NMR of 4-(2,6-Difluoro-4-hydroxyphenyl)-3,4,5-trifluorobenzene

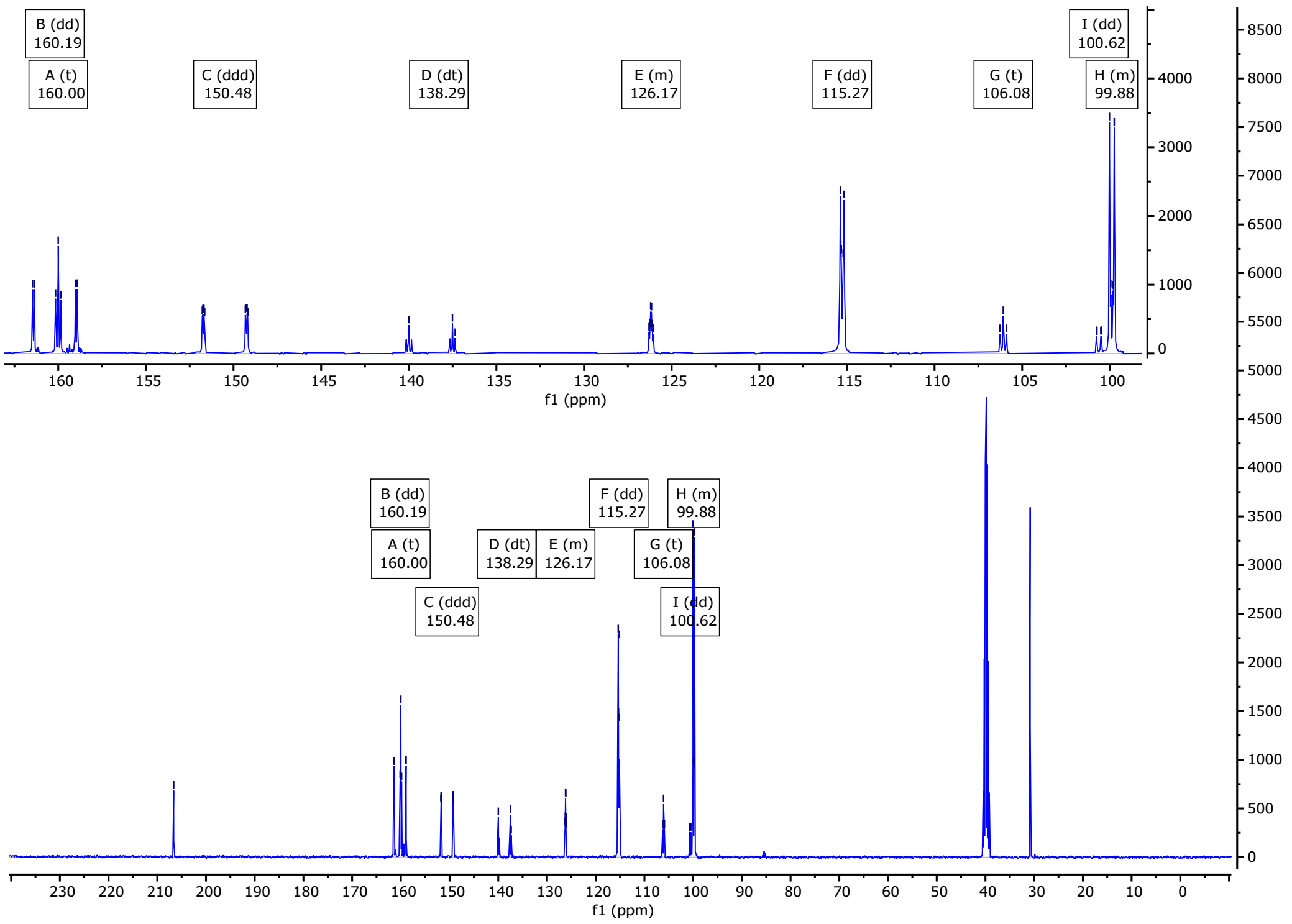


**Figure SI-68:** $^{13}$C{$^1$H} NMR of 4-(2,6-Difluoro-4-hydroxyphenyl)-3,4,5-trifluorobenzene

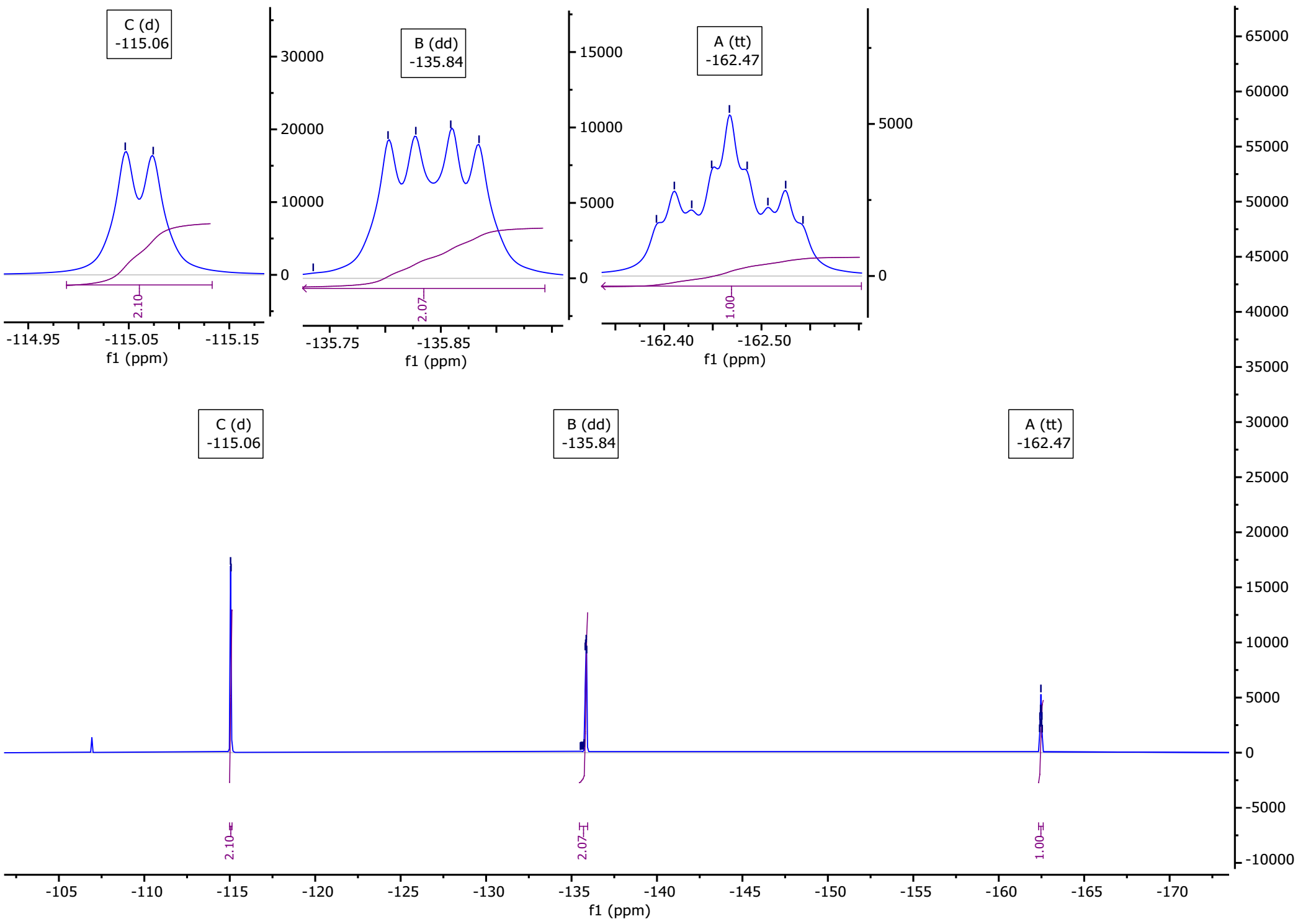


**Figure SI-69:** $^{19}F$ NMR of 4-(2,6-Difluoro-4-hydroxyphenyl)-3,4,5-trifluorobenzene

## 10.7. Scheme 7

### 10.7.1. 2,6-Difluoro-4-(4-hydroxyphenyl)toluene

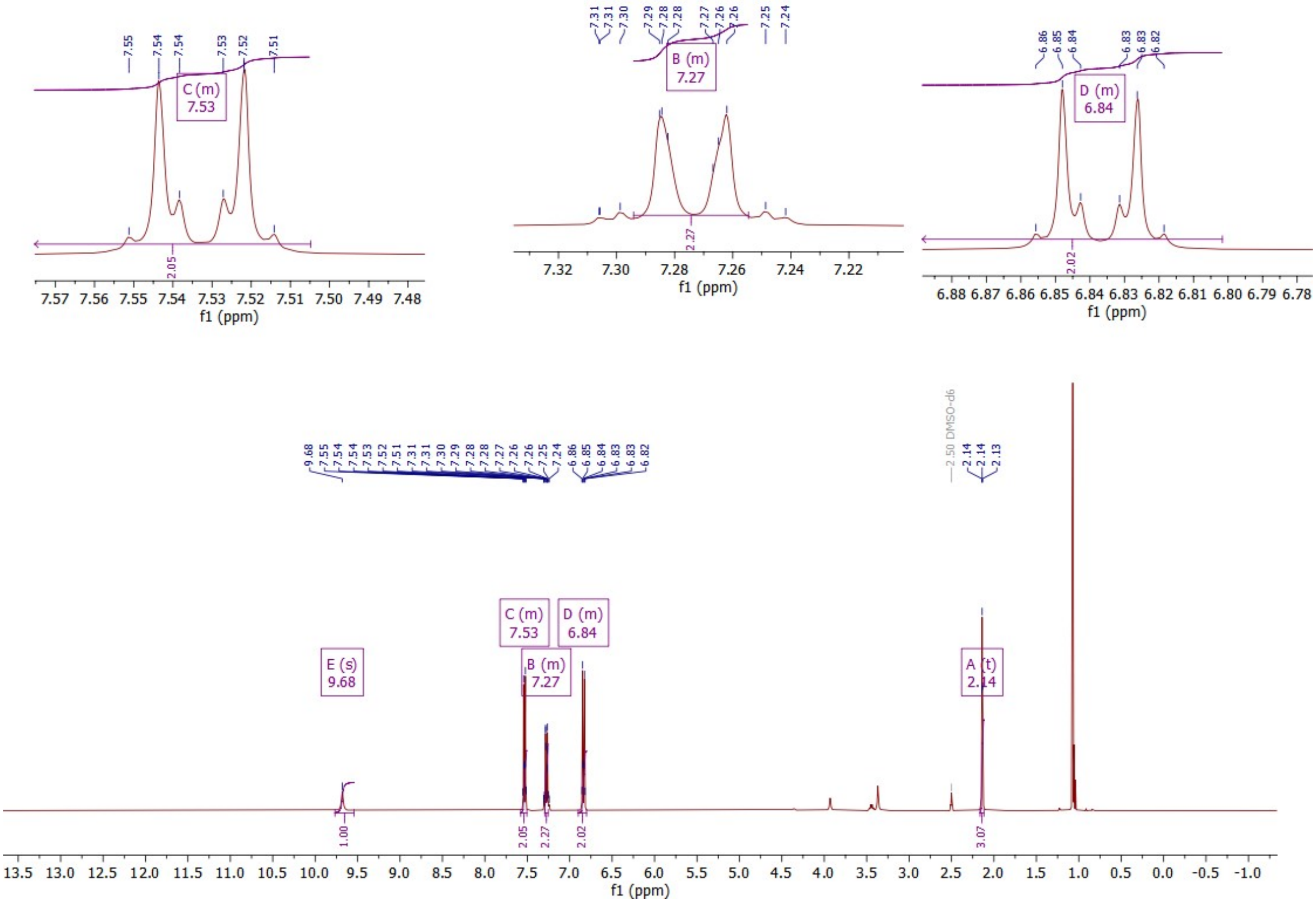


**Figure SI-70:** ¹H NMR of 2,6-Difluoro-4-(4-hydroxyphenyl)toluene)

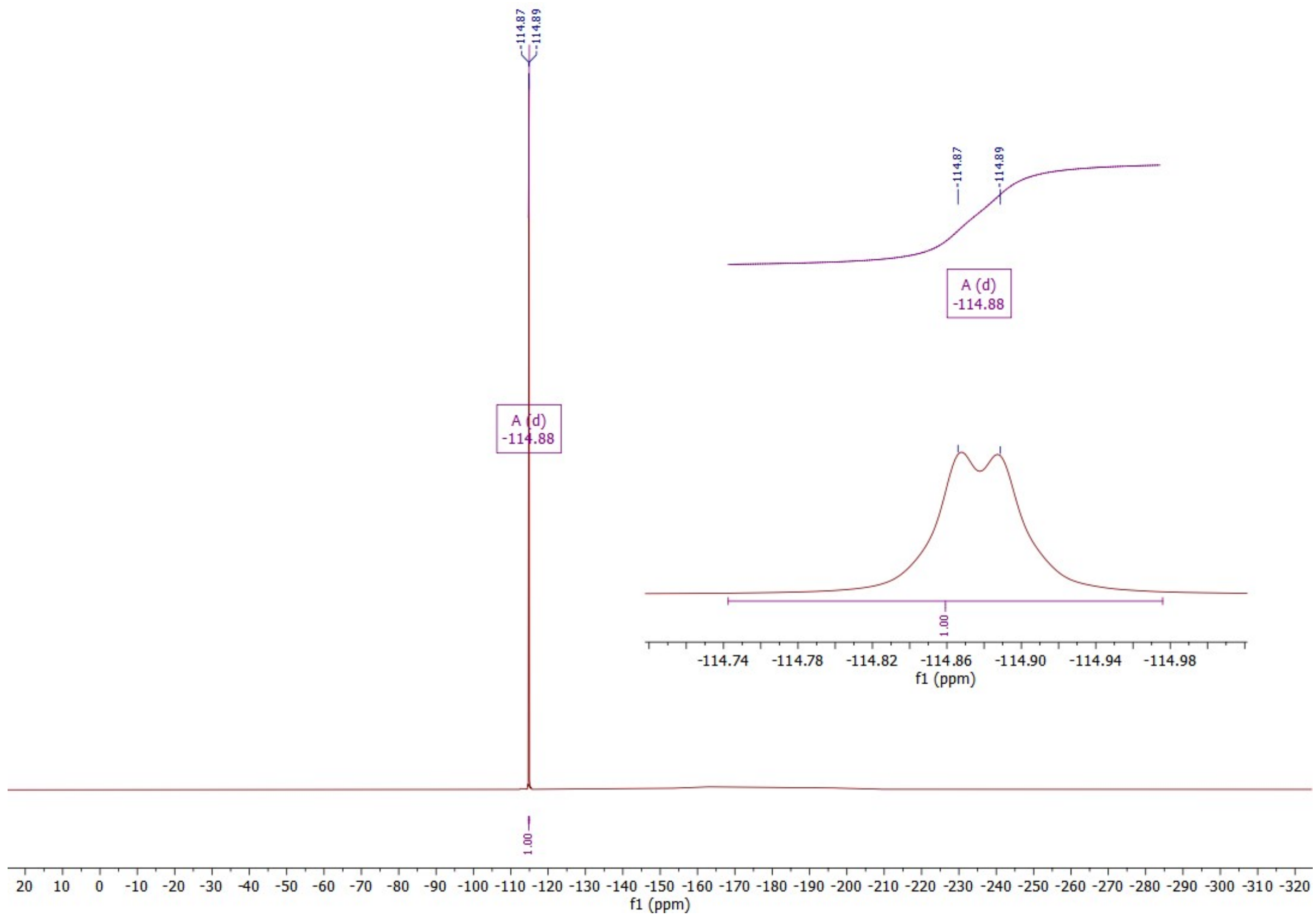


**Figure SI-71:** $^{19}$F NMR of 2,6-difluoro-4-(4-hydroxyphenyl)toluene)

### 10.7.2. 3',5'-difluoro-4'-methyl-[1,1'-biphenyl]-4-yl 2,6-difluoro-4-(5-propyl-1,3-dioxan-2-yl)benzoate

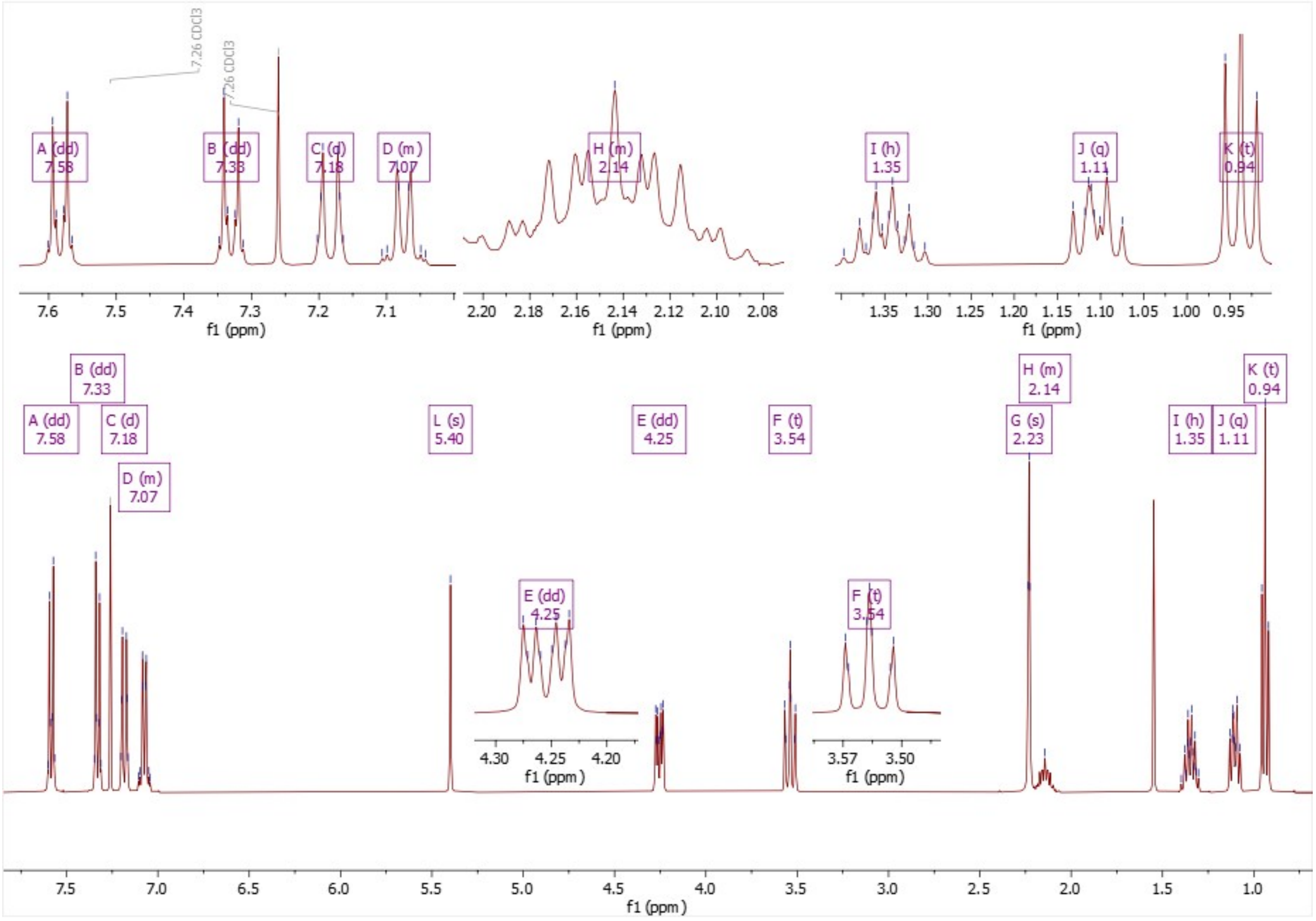


**Figure SI-72:** $^{1}H$ NMR for 3',5'-difluoro-4'-methyl-[1,1'-biphenyl]-4-yl 2,6-difluoro-4-(5-propyl-1,3-dioxan-2-yl)benzoate

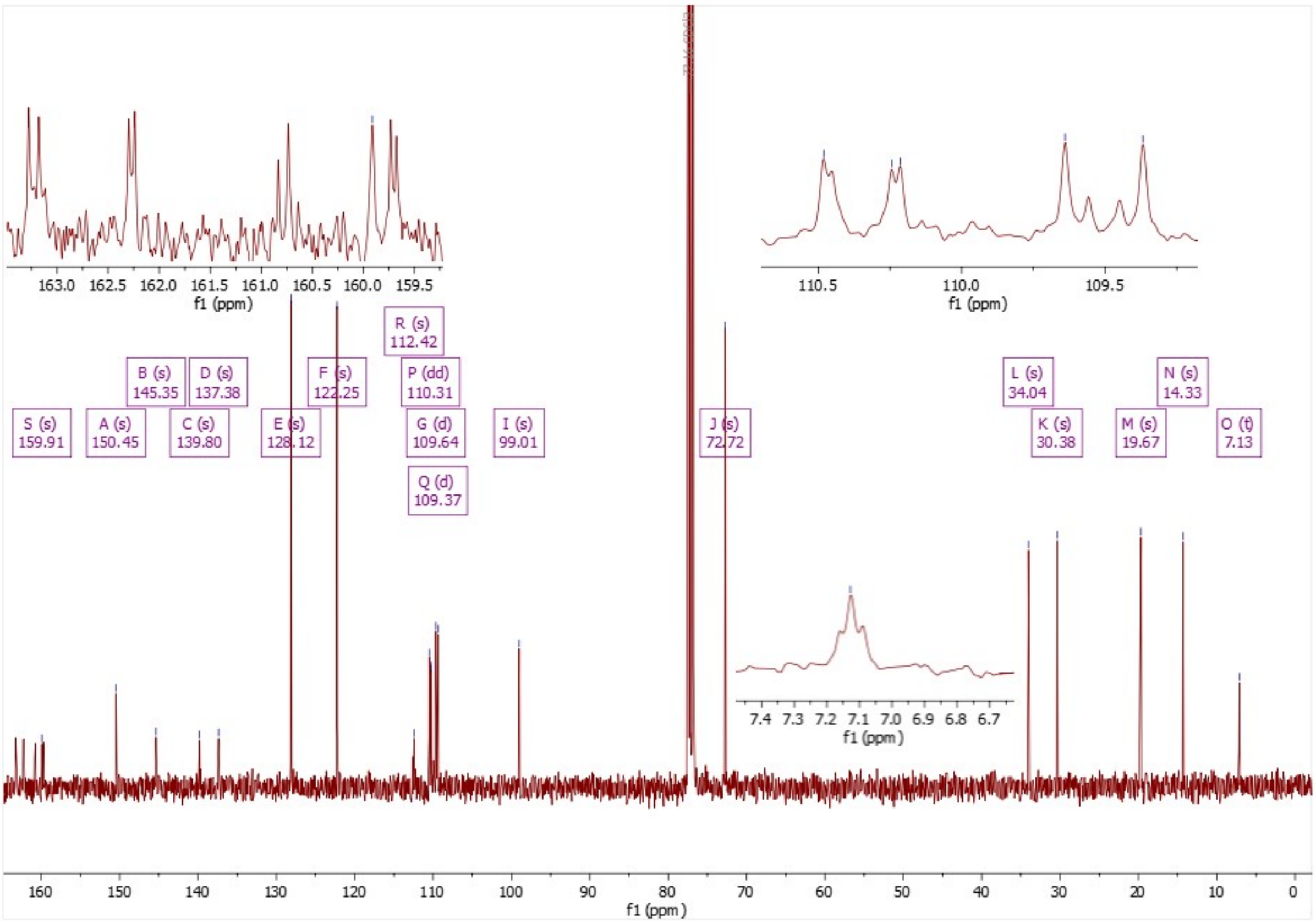


**Figure SI-73:** $^{13}C\{^{1}H\}$ NMR for 3',5'-difluoro-4'-methyl-[1,1'-biphenyl]-4-yl 2,6-difluoro-4-(5-propyl-1,3-dioxan-2-yl)benzoate

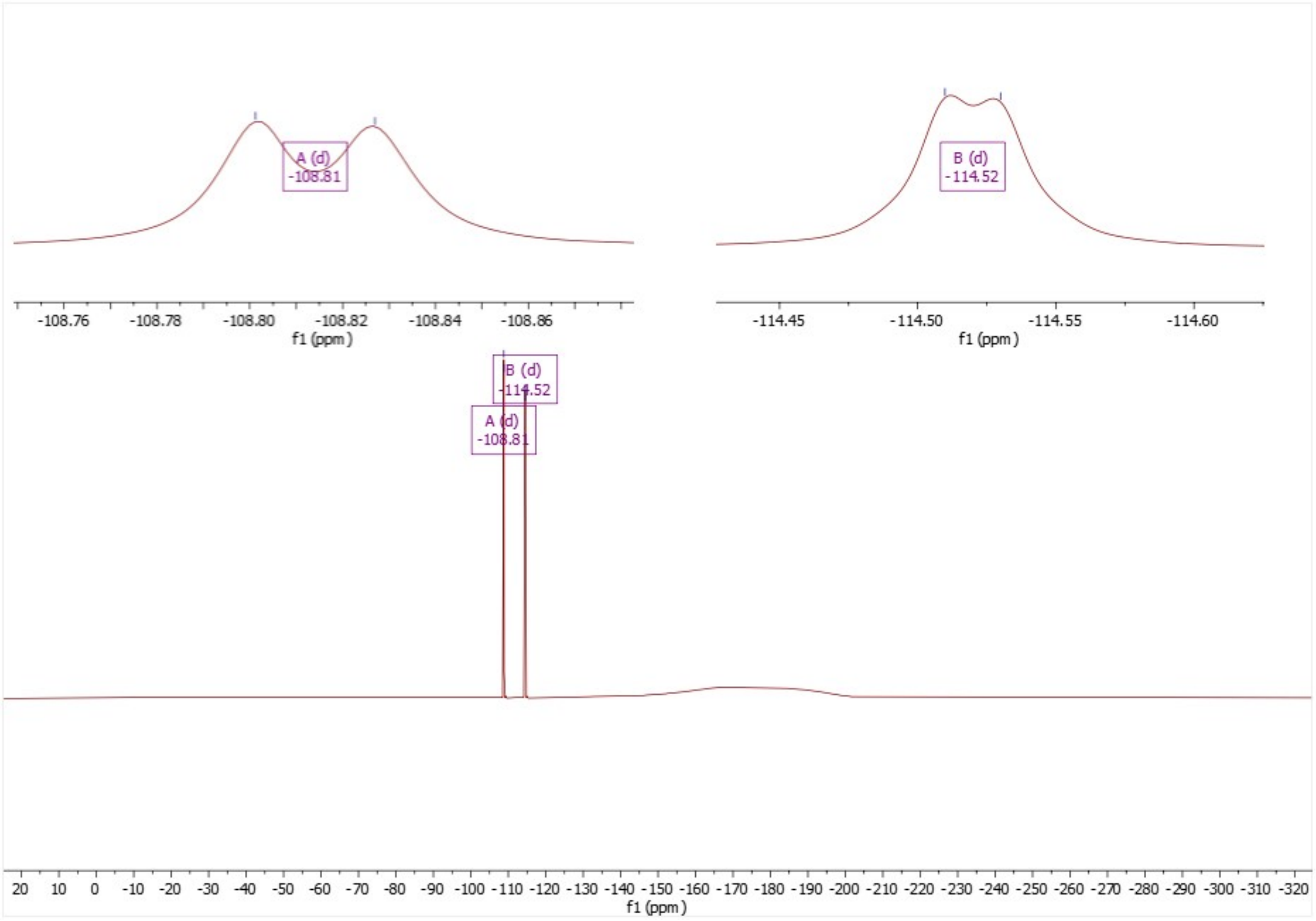


**Figure SI-74:** $^{19}F$ NMR for 3',5'-difluoro-4'-methyl-[1,1'-biphenyl]-4-yl 2,6-difluoro-4-(5-propyl-1,3-dioxan-2-yl)benzoate

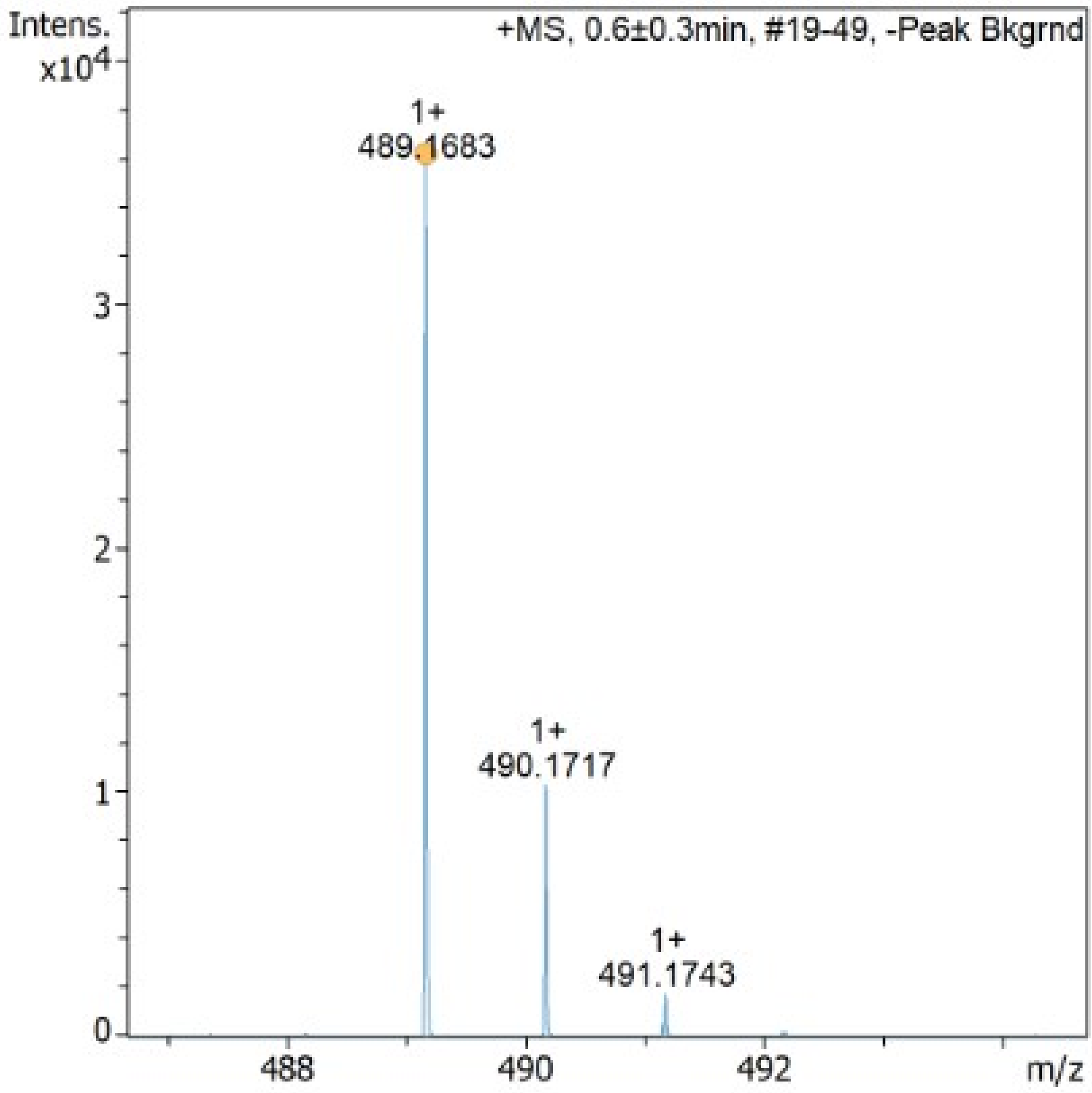


**Figure SI-75:** HRMS for 3',5'-difluoro-4'-methyl-[1,1'-biphenyl]-4-yl 2,6-difluoro-4-(5-propyl-1,3-dioxan-2-yl)benzoate

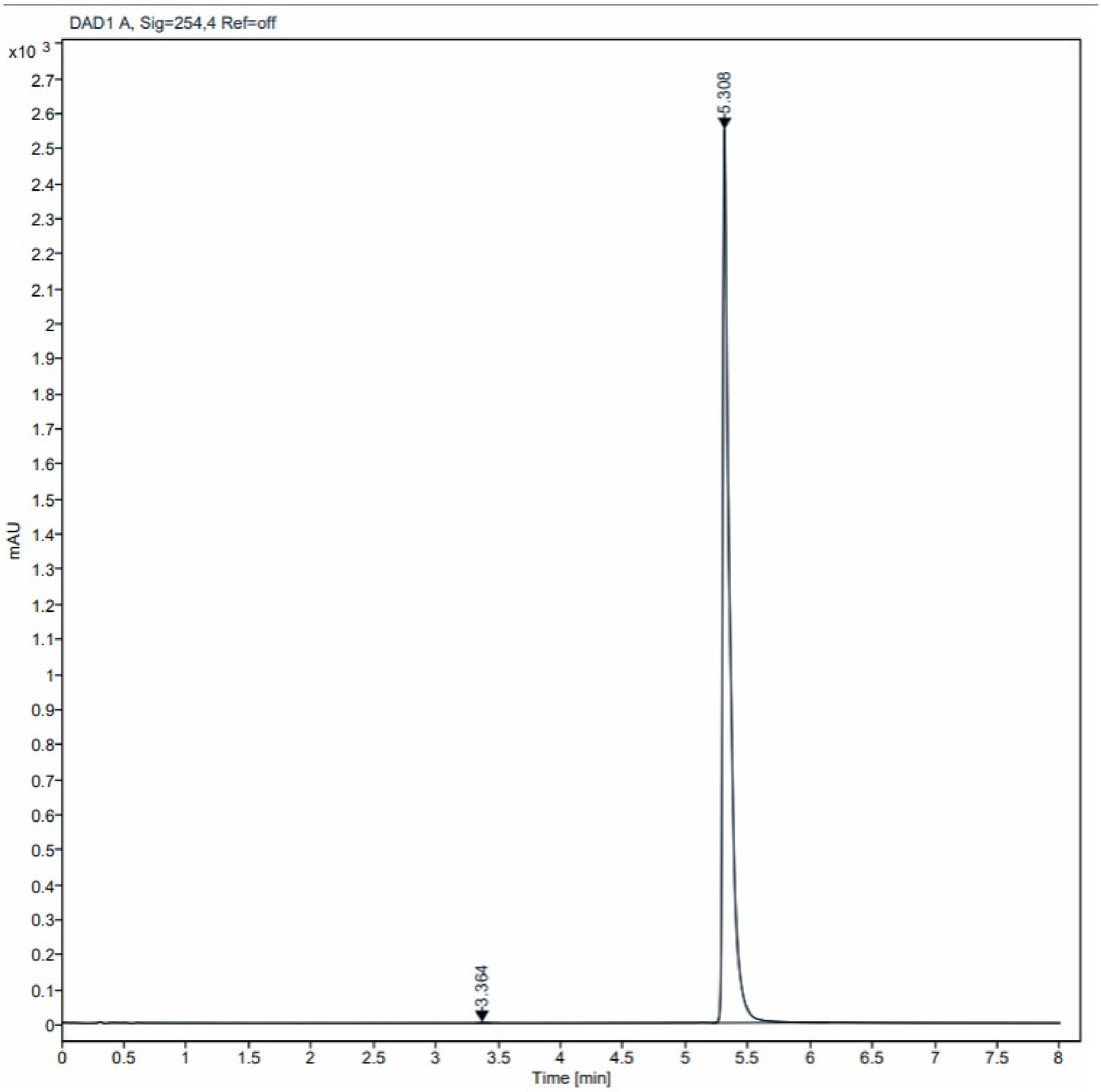


**Figure SI-76:** HPLC for 3',5'-difluoro-4'-methyl-[1,1'-biphenyl]-4-yl 2,6-difluoro-4-(5-propyl-1,3-dioxan-2-yl)benzoate

### 10.8.1. 4-((4-Ethylphenoxy)carbonyl)phenyl-2,4-dimethoxybenzoate (COB30 IDX 1014)

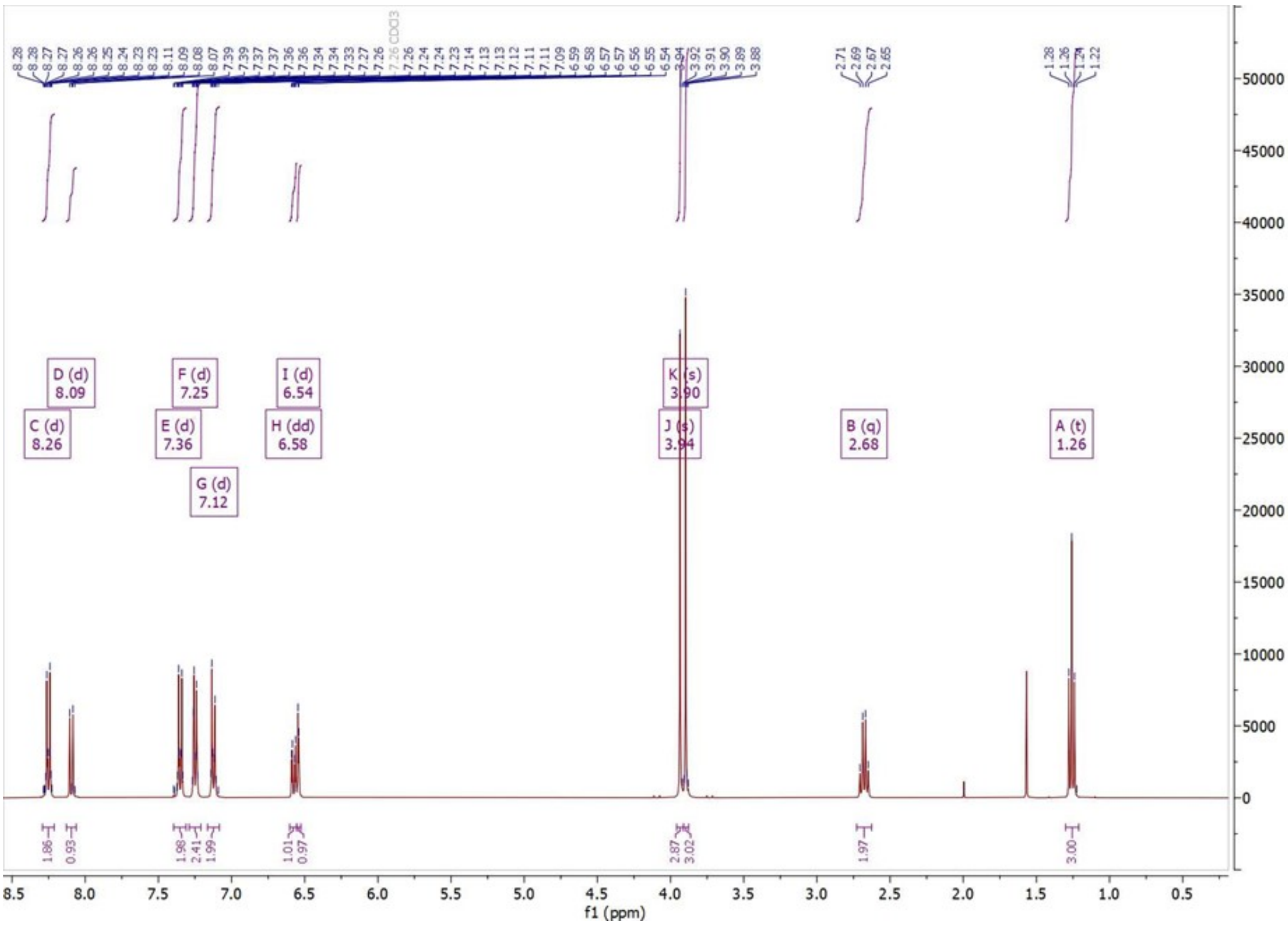


**Figure SI-77:** $^1$H NMR for 4-((4-Ethylphenoxy)carbonyl)phenyl-2,4-dimethoxybenzoate (COB30 IDX 1014)

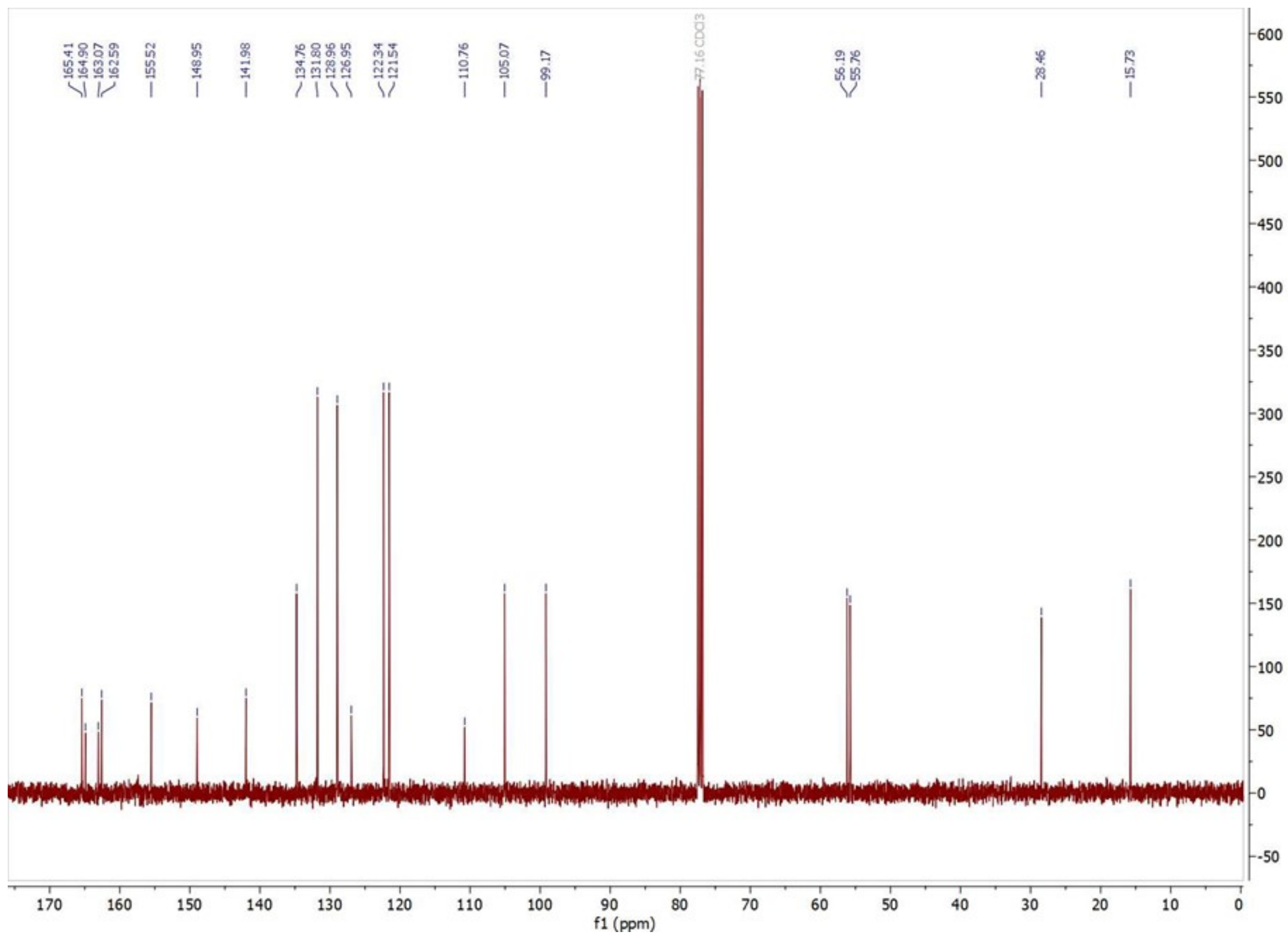


**Figure SI-78:** $^{13}C\{^{1}H\}$ NMR for 4-((4-Ethylphenoxy)carbonyl)phenyl-2,4-dimethoxybenzoate (COB30 IDX 1014)

### 7.8.2. 4-((4-formylphenyloxy)carbonyl)phenyl 2,4-dimethoxybenzoate (COB27 IDX 1407)

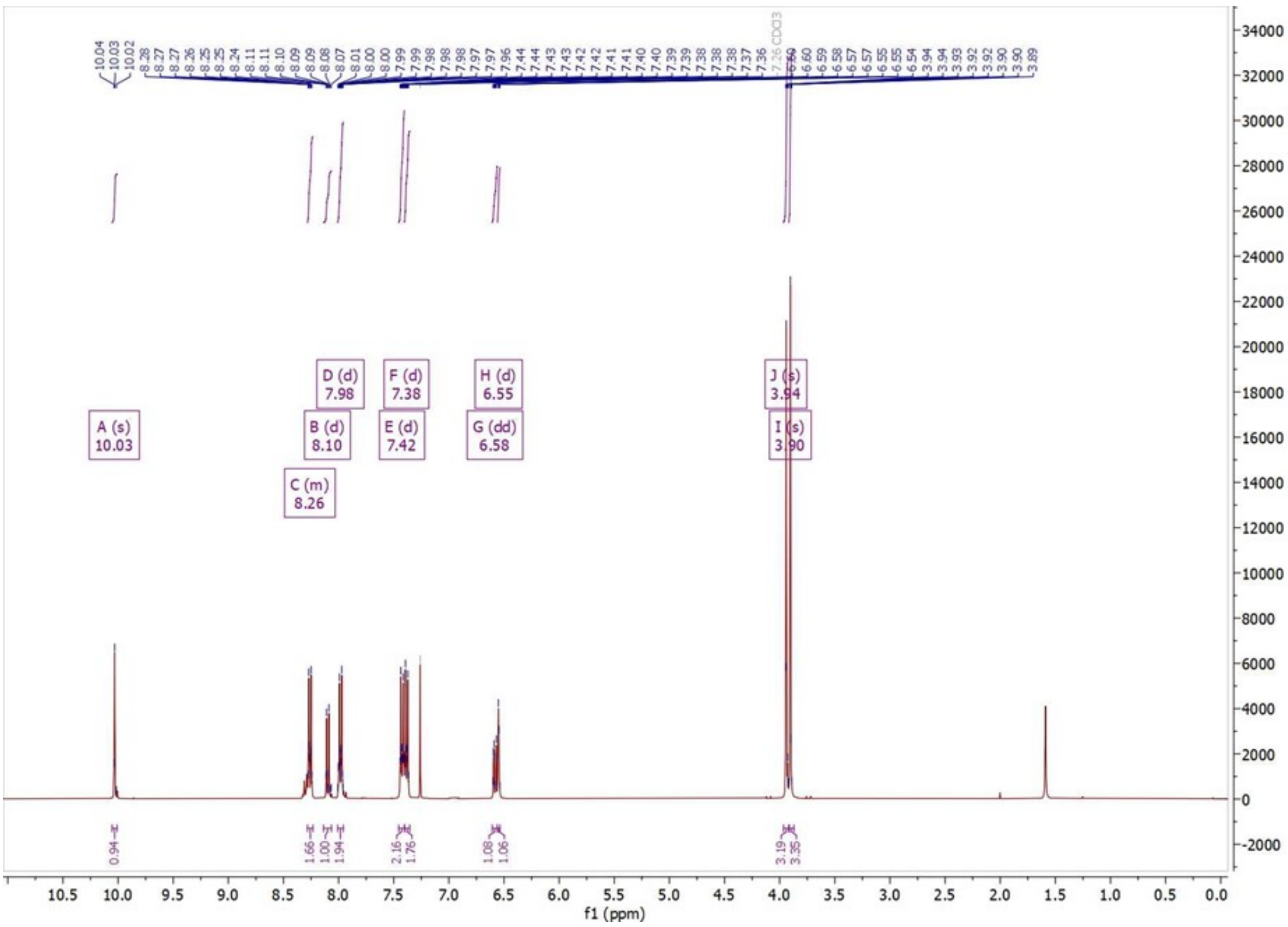

**Figure SI-79:** $^1$H NMR for IDX 4-((4-formylphenyloxy)carbonyl)phenyl 2,4-dimethoxybenzoate

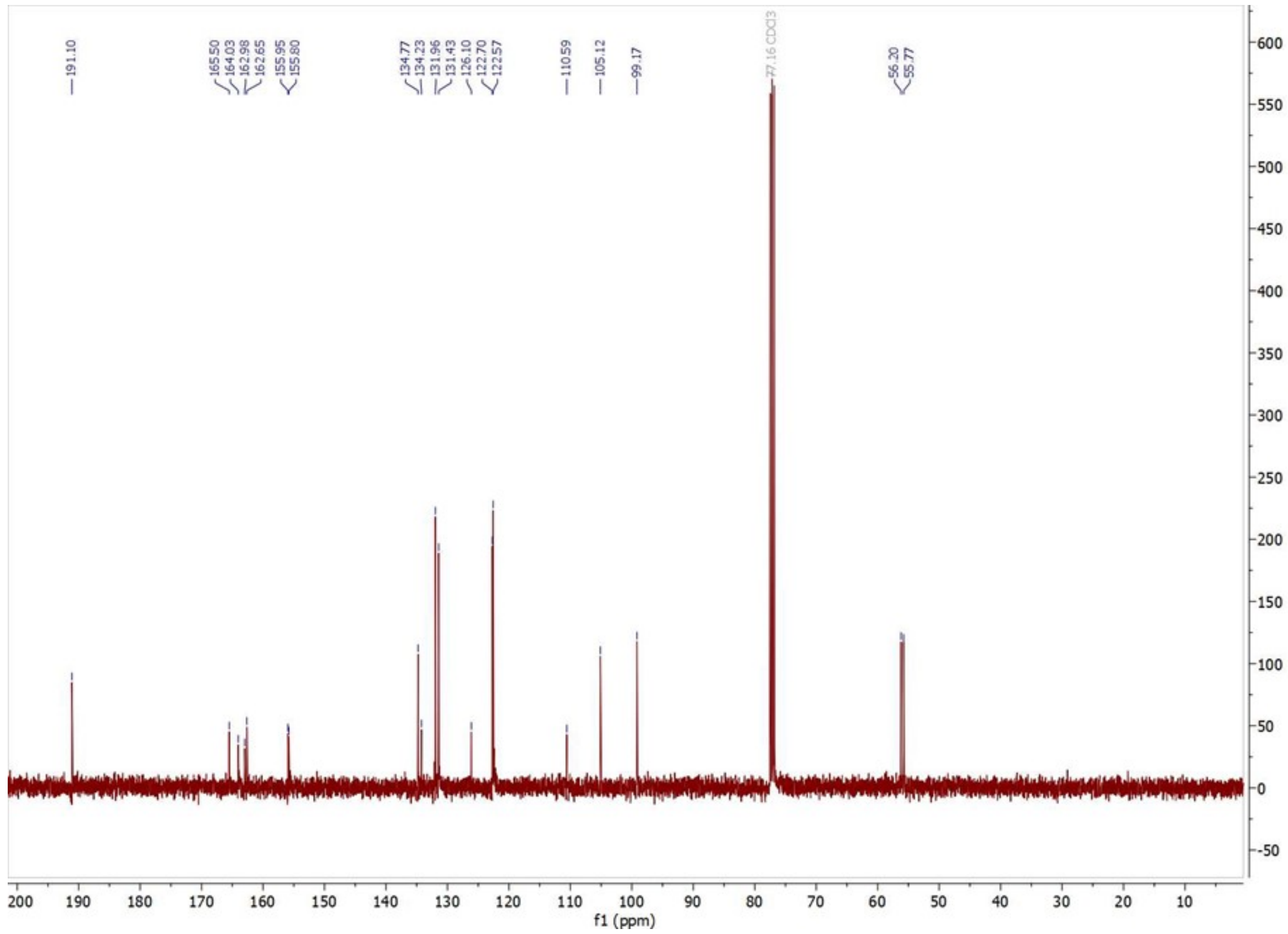


**Figure SI-80:** $^{13}C\{^{1}H\}$ NMR for 4-((4-formylphenyloxy)carbonyl)phenyl 2,4-dimethoxybenzoate

### 7.9.1. 3',5'-difluoro-4'-cyano-[1,1'-biphenyl]-4-yl 2,6-difluoro-4-(-5-propyl-1,3-dioxan-2-yl)benzoate (RM2926 / IDX2012):

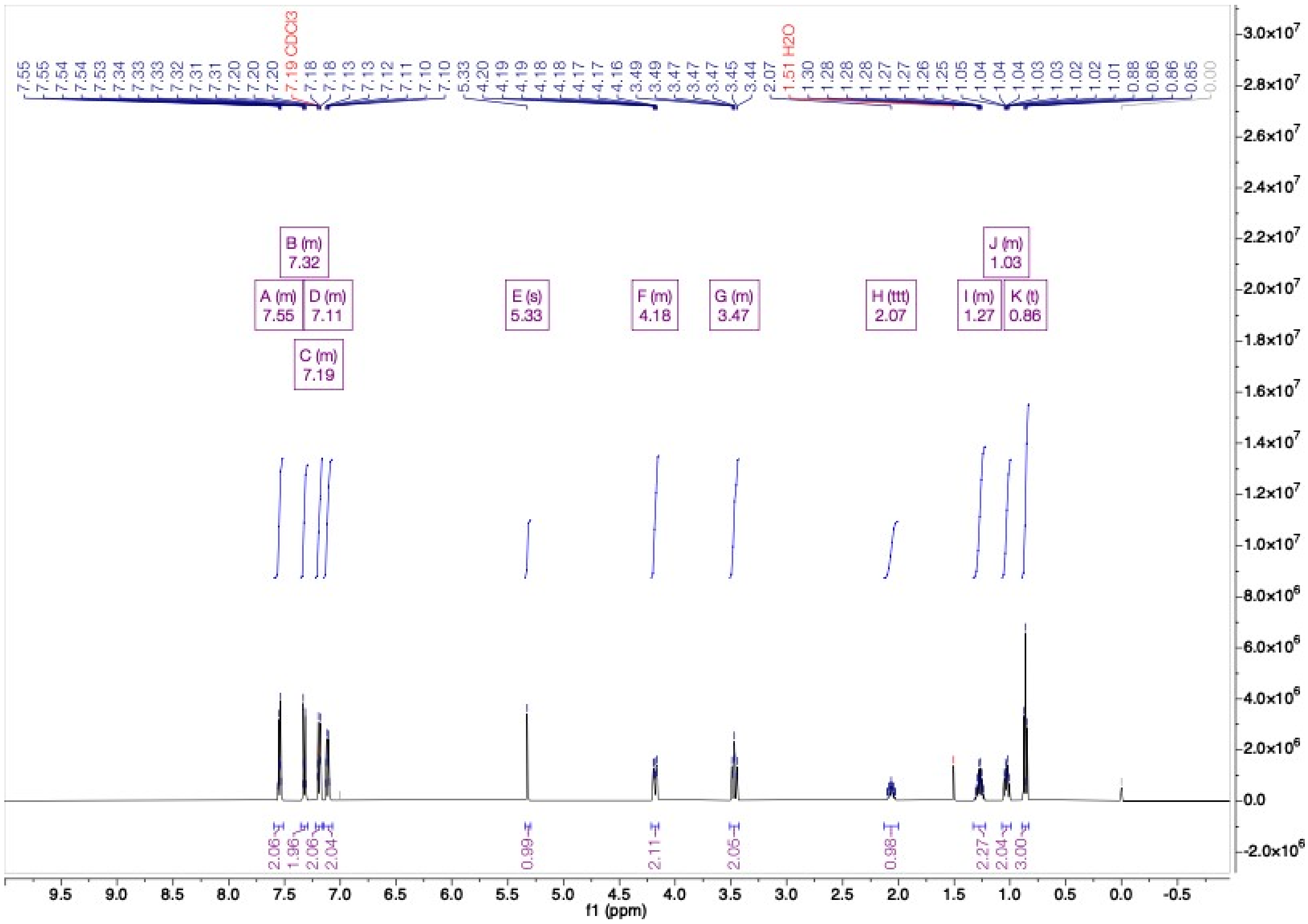

**Figure SI-81:** $^1$H NMR for RM2926

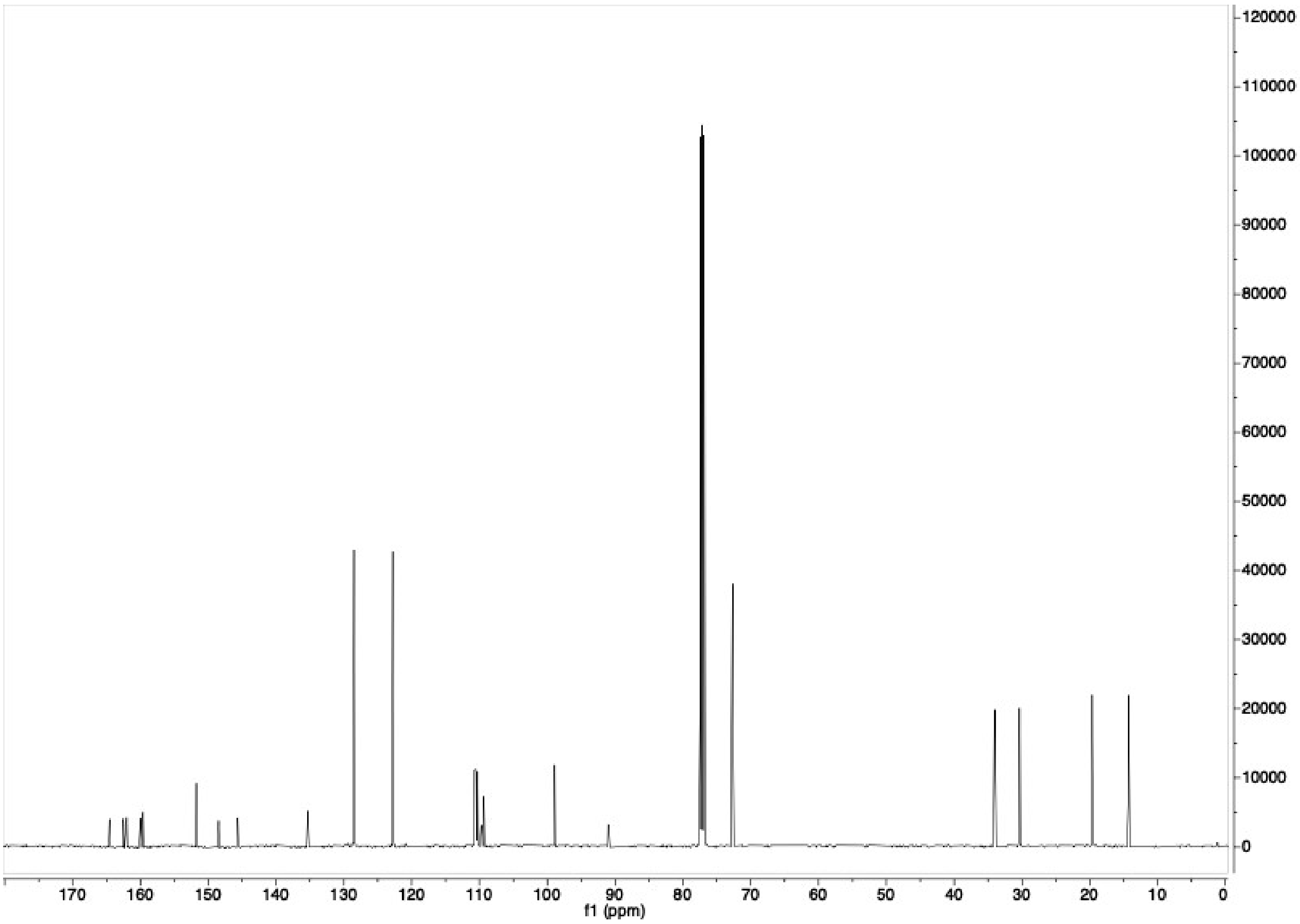


**Figure SI-82:** $^{13}C\{^{1}H\}$ NMR for RM2926

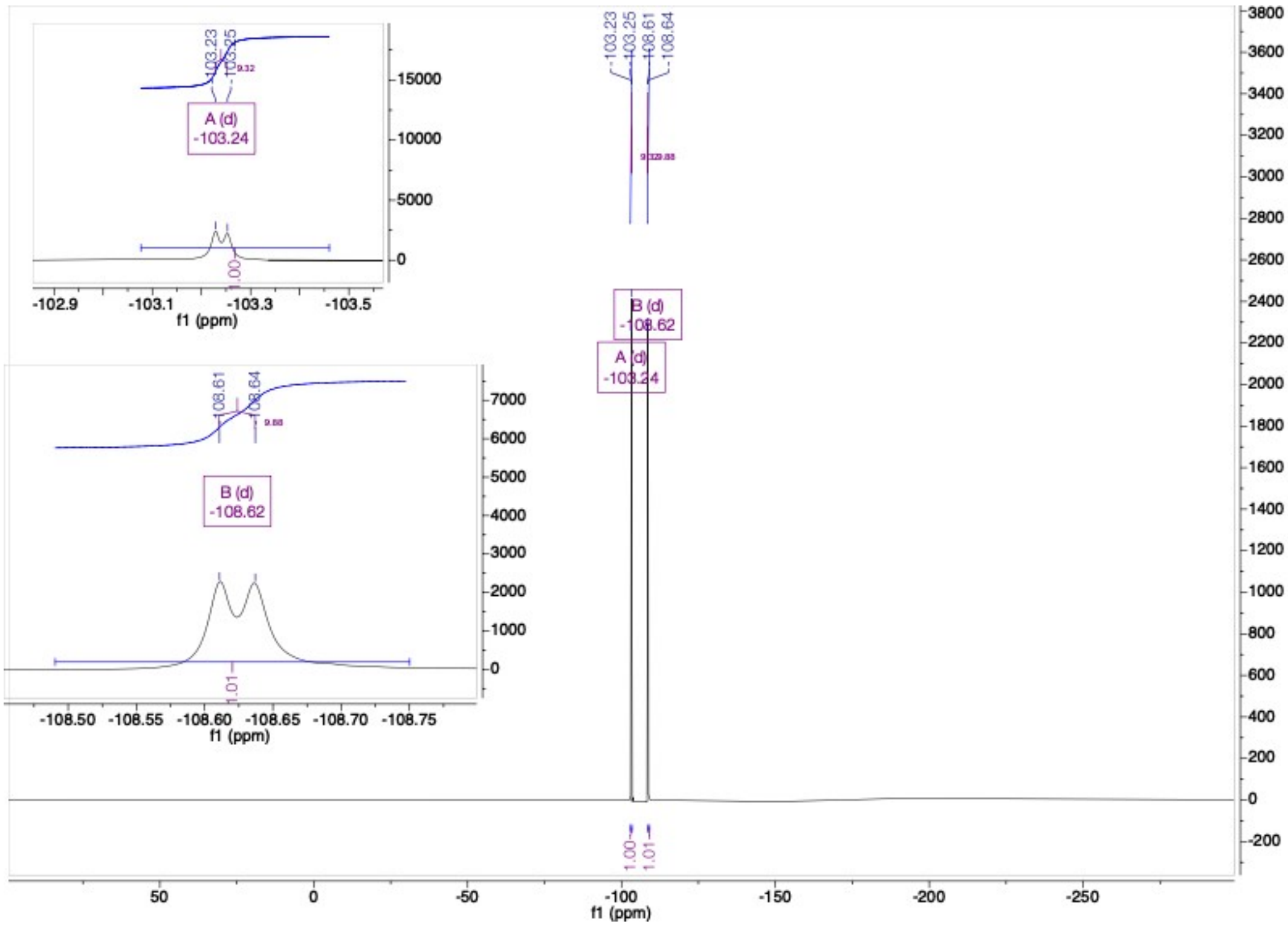


**Figure SI-83:** $^{19}F$ NMR for RM2926

**7.10.1. 4'-cyano-2,3',5',6-tetrafluoro-[1,1'-biphenyl]-4-yl 2,6-difluoro-4-(propoxymethyl)benzoate (SRB-CHEM-163 / IDX2.0713)):**

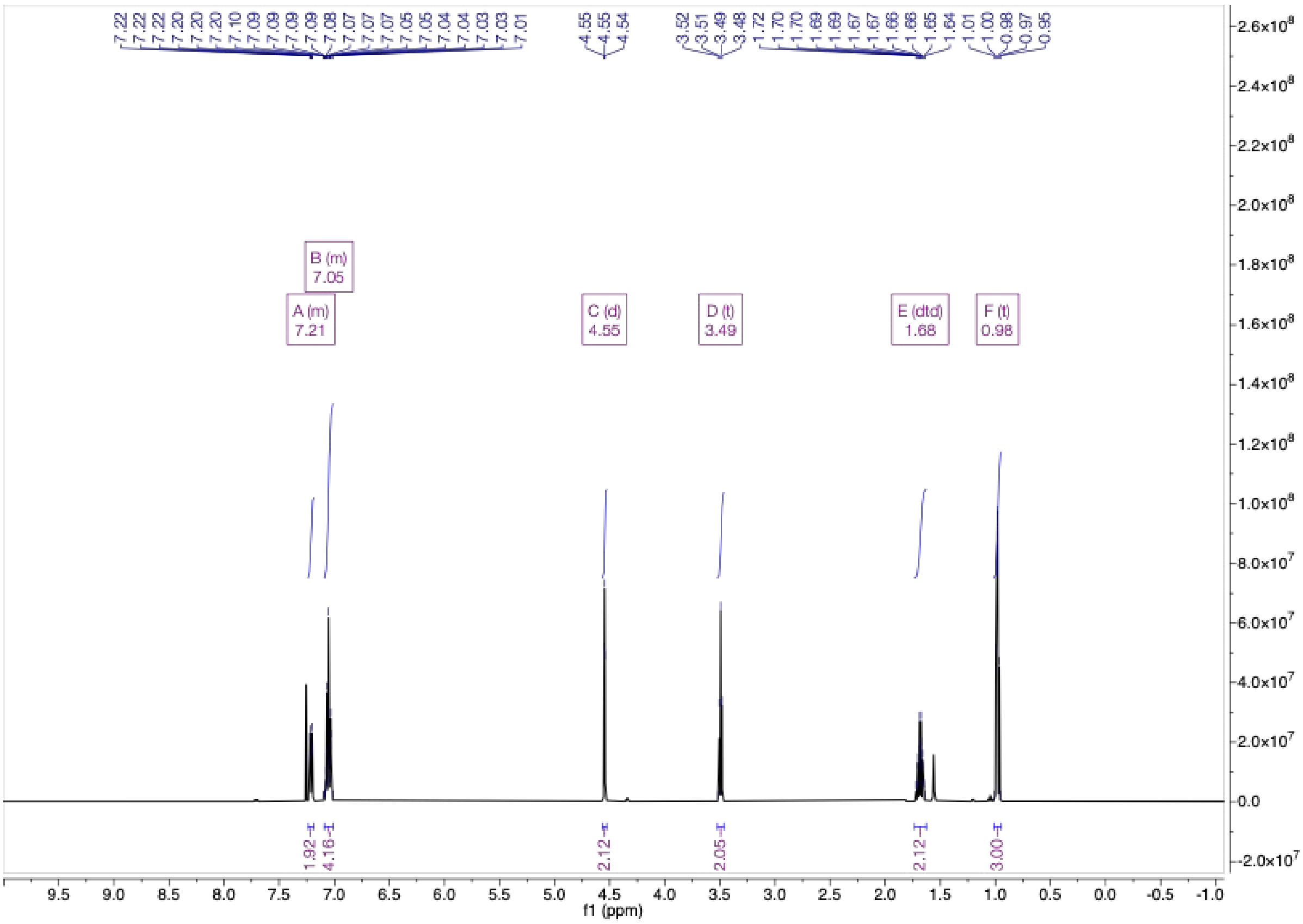


**Figure SI-84:** $^{1}$H NMR of 4'-cyano-2,3',5',6-tetrafluoro-[1,1'-biphenyl]-4-yl 2,6-difluoro-4-(propoxymethyl)benzoate (SRB-CHEM-163 / IDX2.0713)

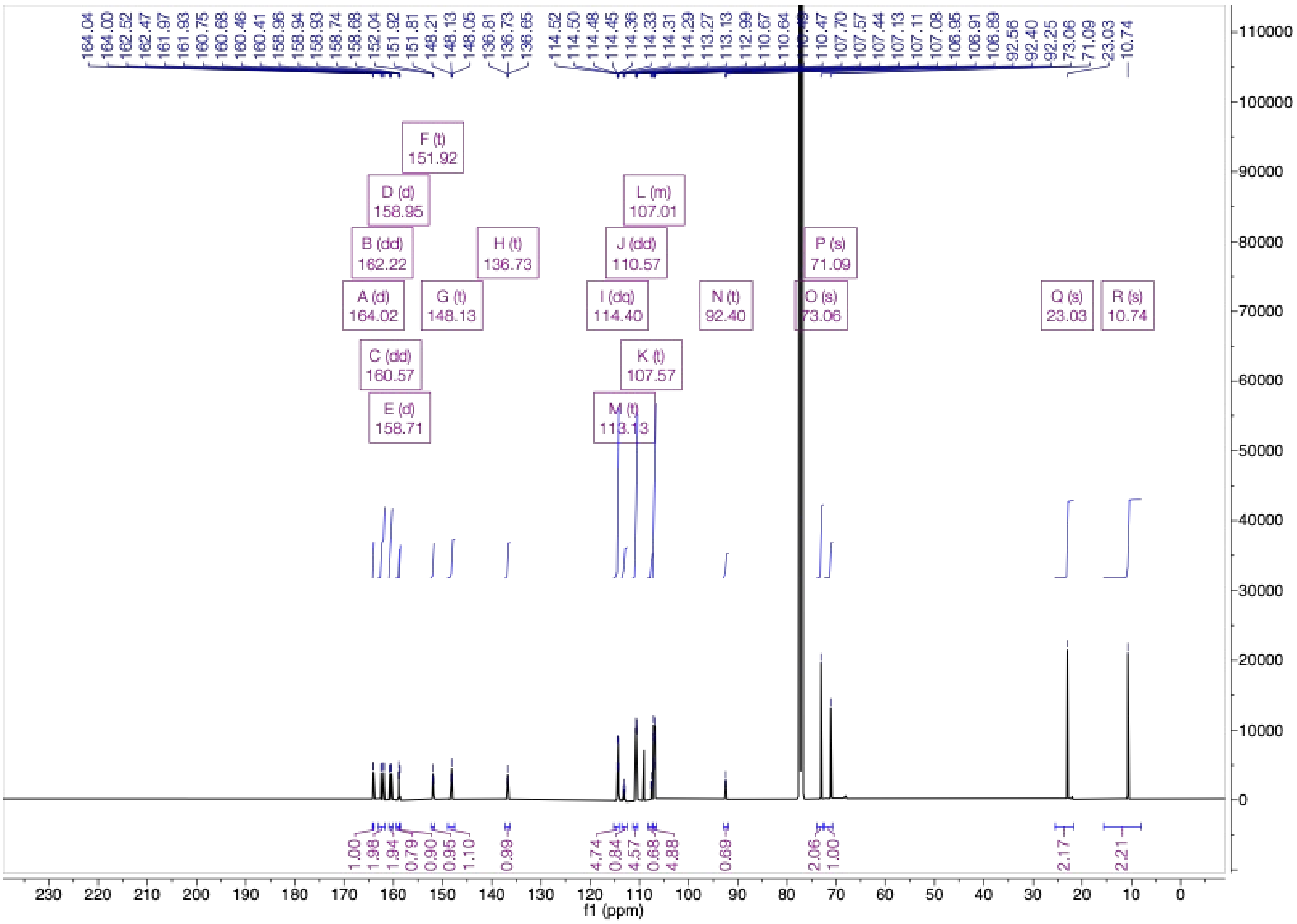

**Figure SI-85:** $^{13}C\{^{1}H\}$ NMR of 4'-cyano-2,3',5',6-tetrafluoro-[1,1'-biphenyl]-4-yl 2,6-difluoro-4-(propoxymethyl)benzoate (SRB-CHEM-163 / IDX2.0713)

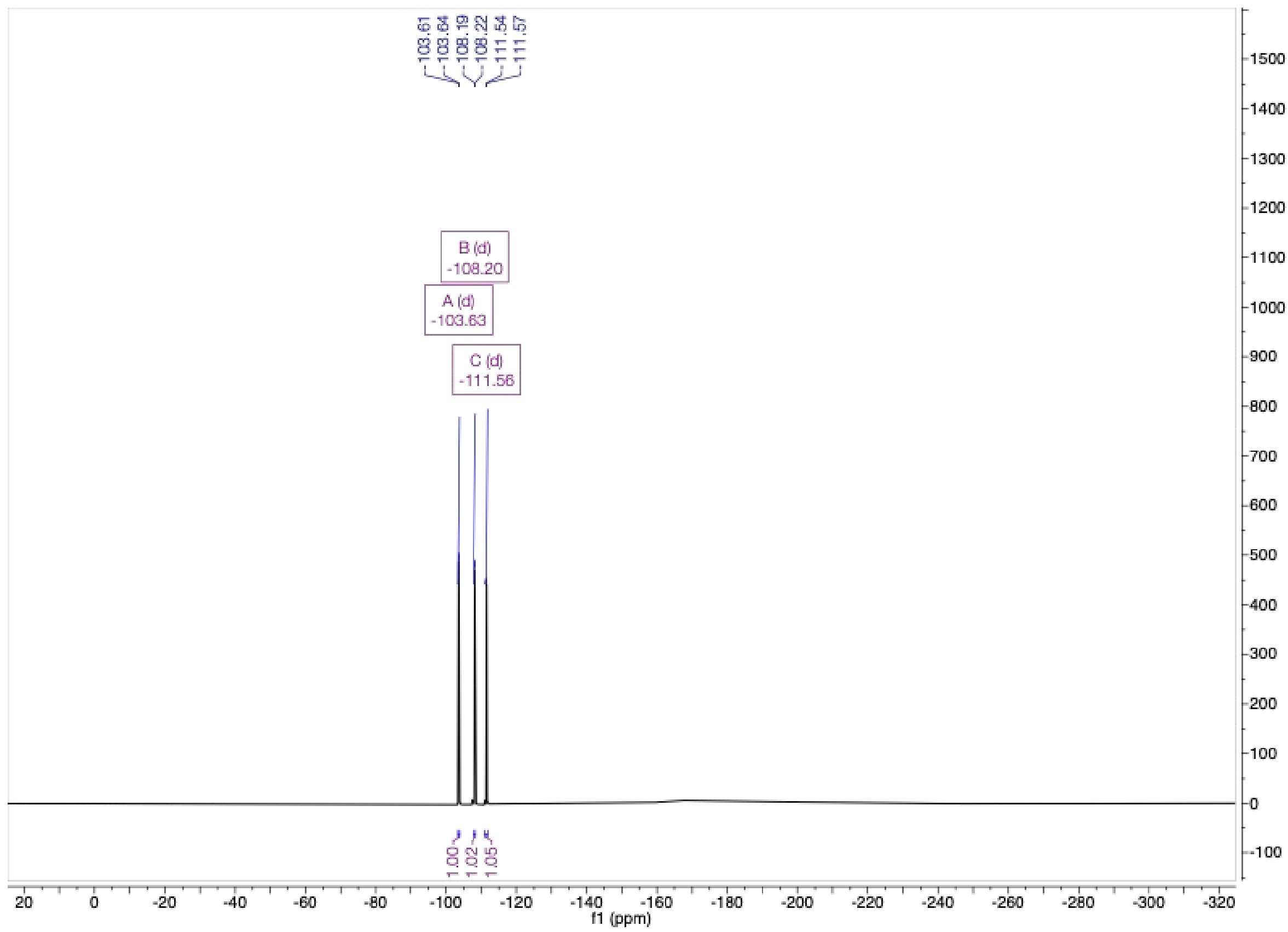


**Figure SI-86:** $^{19}F$ NMR of 4'-cyano-2,3',5',6-tetrafluoro-[1,1'-biphenyl]-4-yl 2,6-difluoro-4-(propoxymethyl)benzoate (SRB-CHEM-163 / IDX2.0713)

### 7.10.1. 4'-cyano-2,3',5',6-tetrafluoro-[1,1'-biphenyl]-4-yl 2,6-difluoro-4-(4-propylcyclohexyl)benzoate (WCO 4.19 / IDX2.1471)

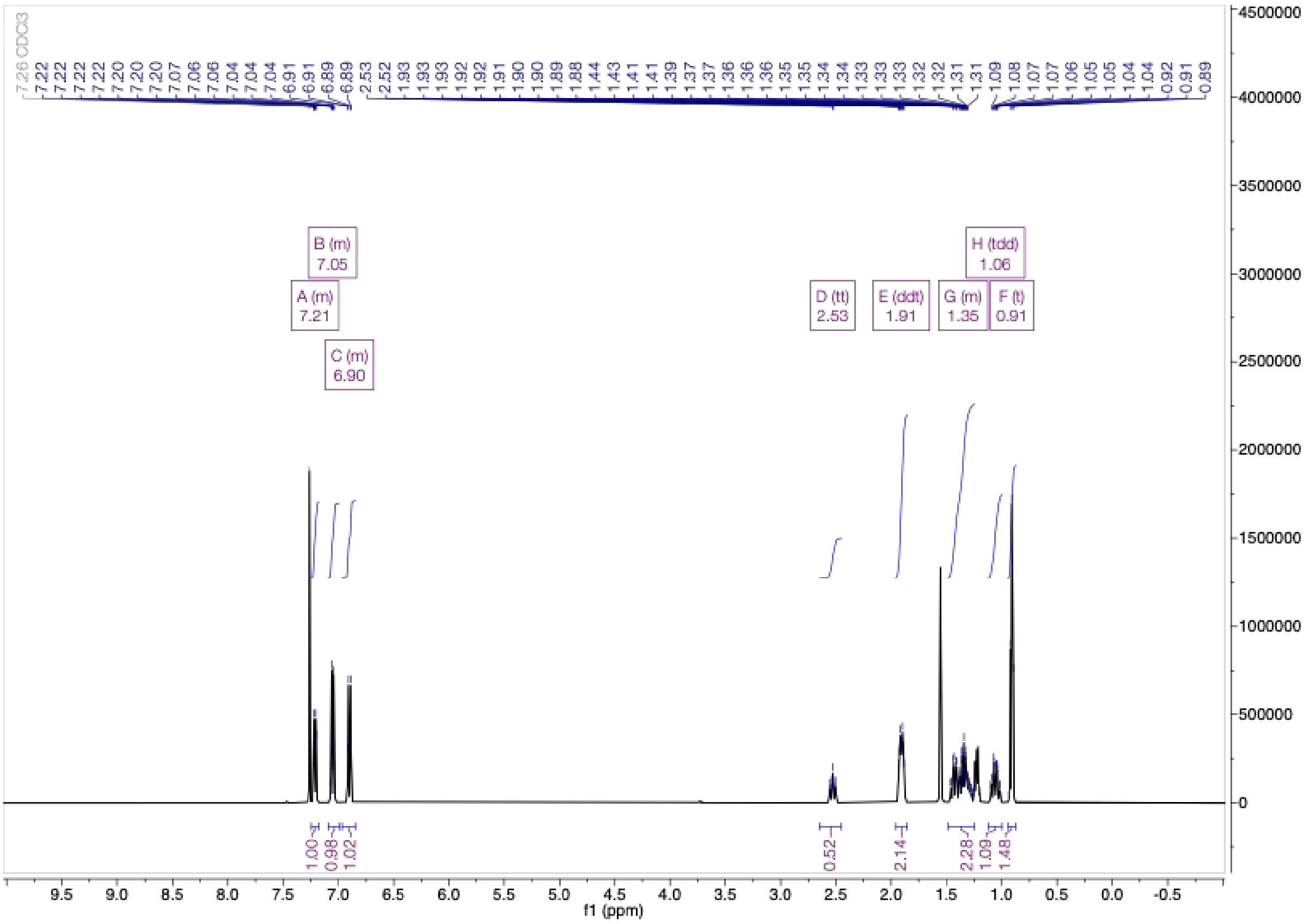


**Figure SI-87:** $^1$H NMR for 4'-cyano-2,3',5',6-tetrafluoro-[1,1'-biphenyl]-4-yl 2,6-difluoro-4-(4-propylcyclohexyl)benzoate (WCO 4.19 / IDX2.1471)

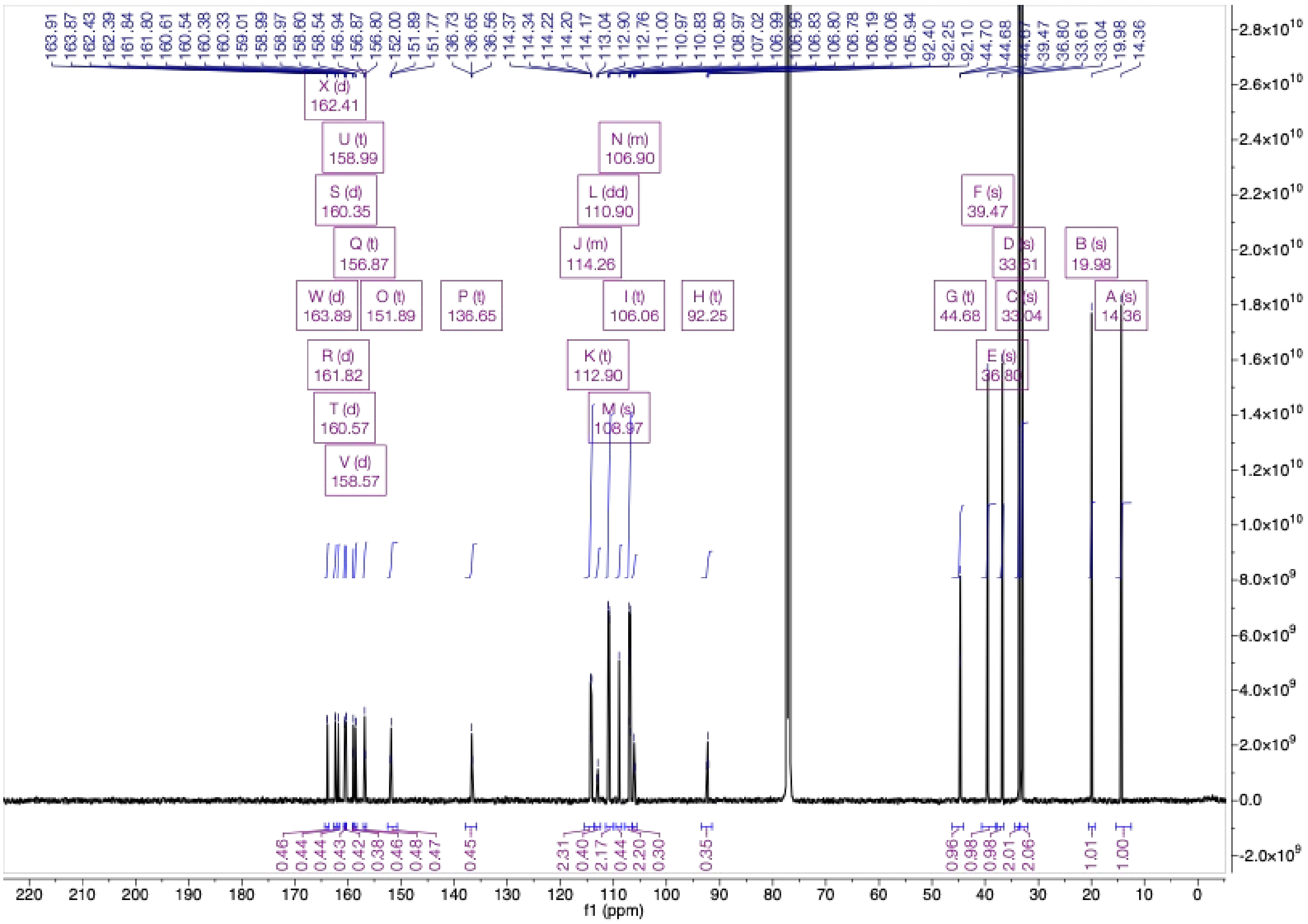

**Figure SI-88:** $^{13}C\{^1H\}$ NMR for 4'-cyano-2,3',5',6-tetrafluoro-[1,1'-biphenyl]-4-yl 2,6-difluoro-4-(4-propylcyclohexyl)benzoate (WCO 4.19 / IDX2.1471)

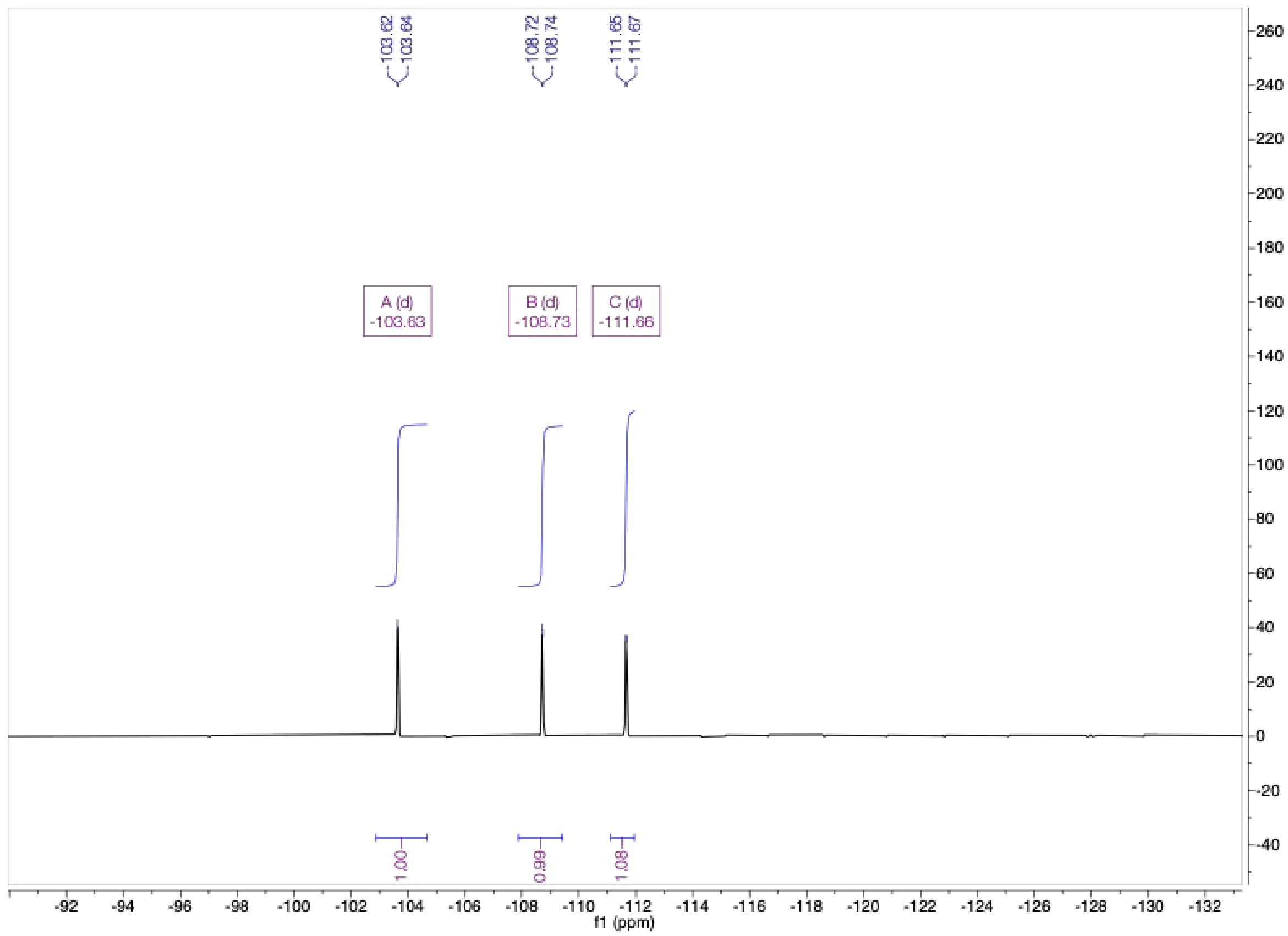


**Figure SI-89:** $^{19}F$ NMR for 4'-cyano-2,3',5',6-tetrafluoro-[1,1'-biphenyl]-4-yl 2,6-difluoro-4-(4-propylcyclohexyl)benzoate (WCO 4.19 / IDX2.1471)

## 12. Synthesised Candidate Materials

***Cycle One***

***DIO-like***

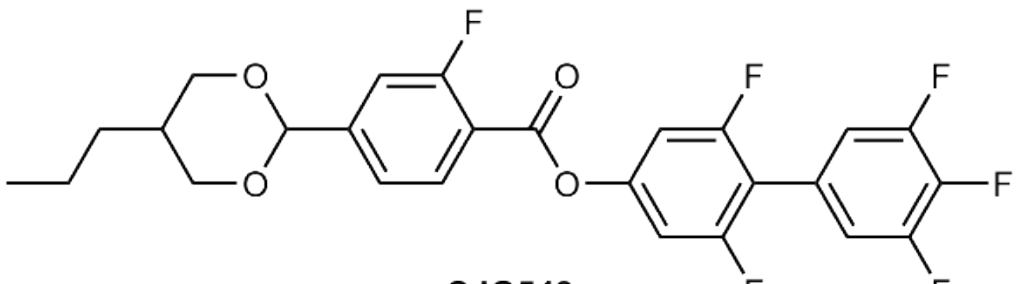

**CJG548**
K 145 $N_S$ 155 N 190 I

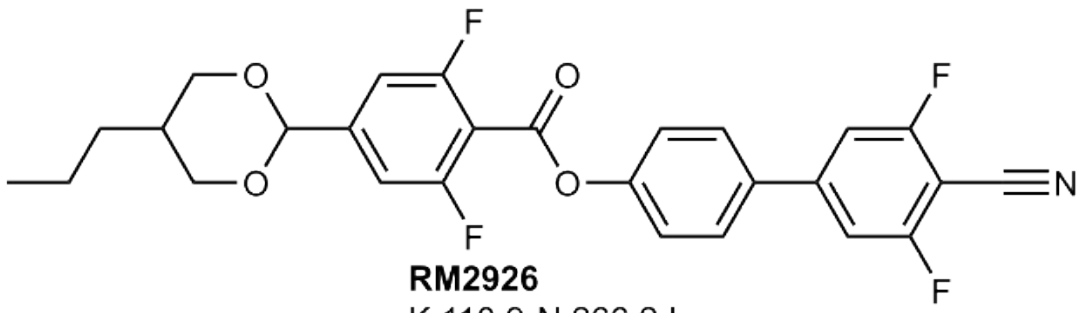

**RM2926**
K 110.9 N 266.2 I

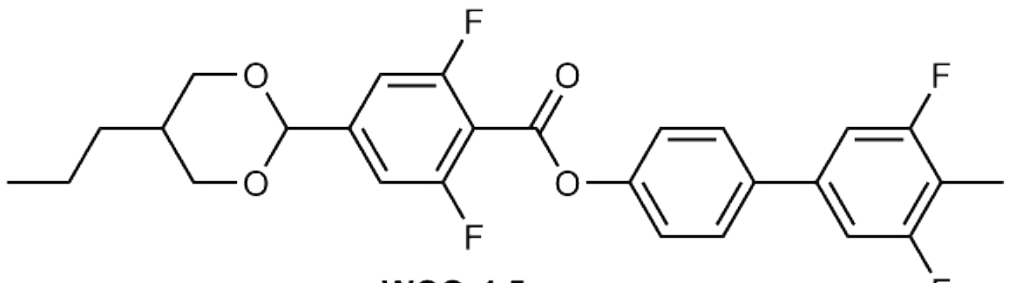

**WCO-4.5**
K 96.7 N 218.7 I

***RM734-like***

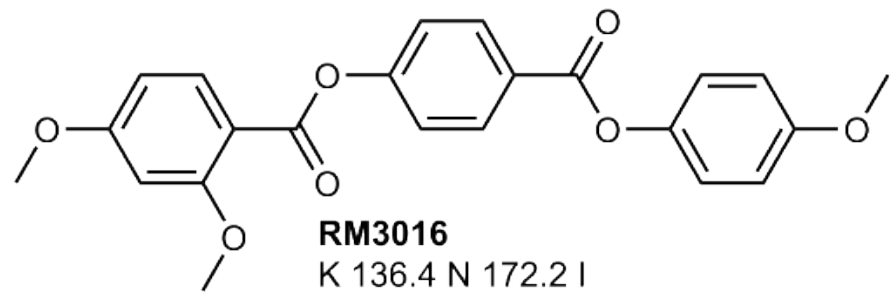

**RM3016**
K 136.4 N 172.2 I

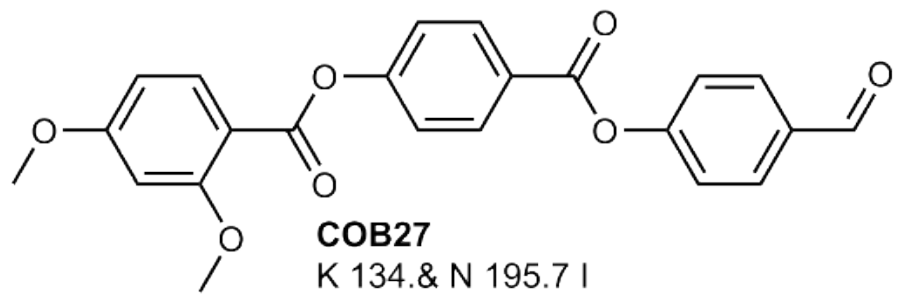

**COB27**
K 134.& N 195.7 I

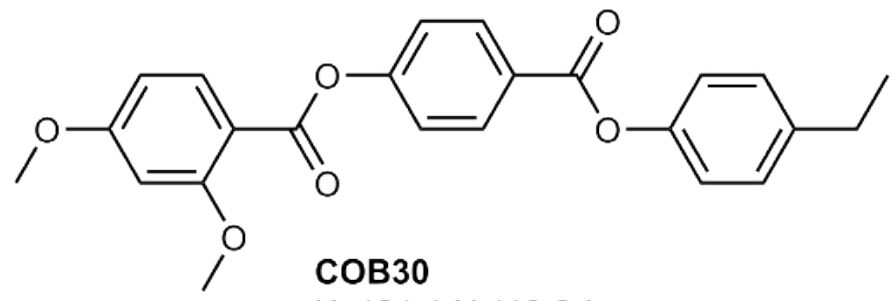

**COB30**
K 131.1 N 110.6 I

***UQU-like***

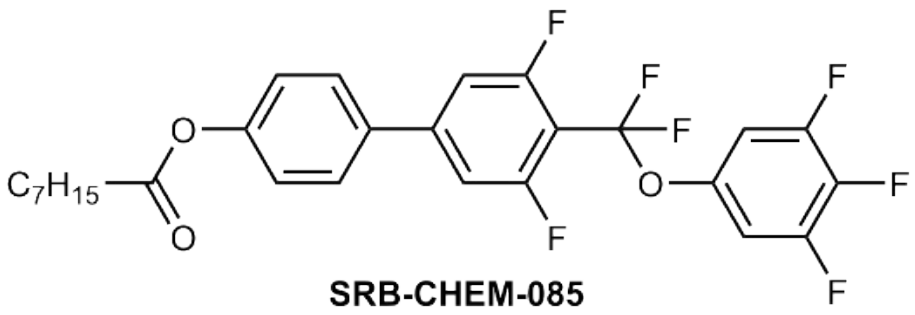

**SRB-CHEM-085**
K 64.8 N 33.6 I

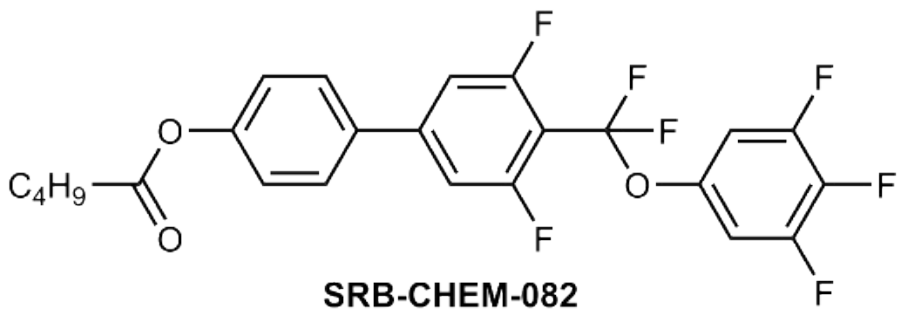

**SRB-CHEM-082**
K 63.1 I

***UZUU-like***

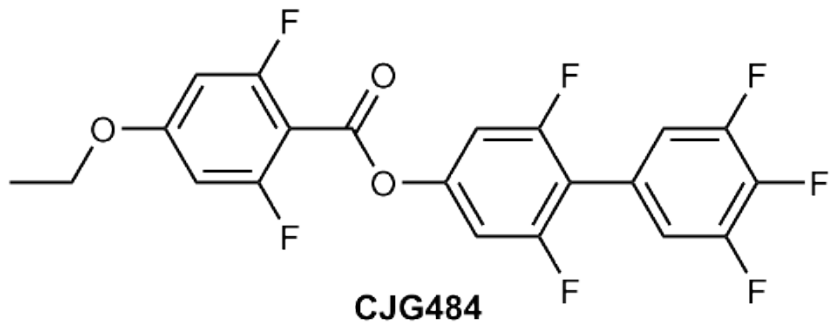

**CJG484**
K 140 I

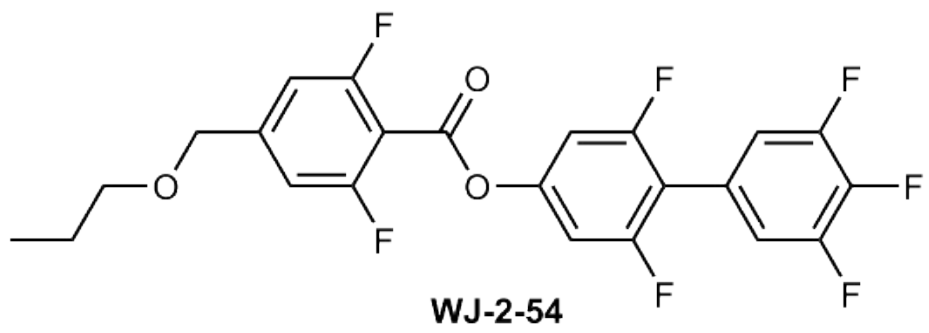

**WJ-2-54**
K 111.9 I

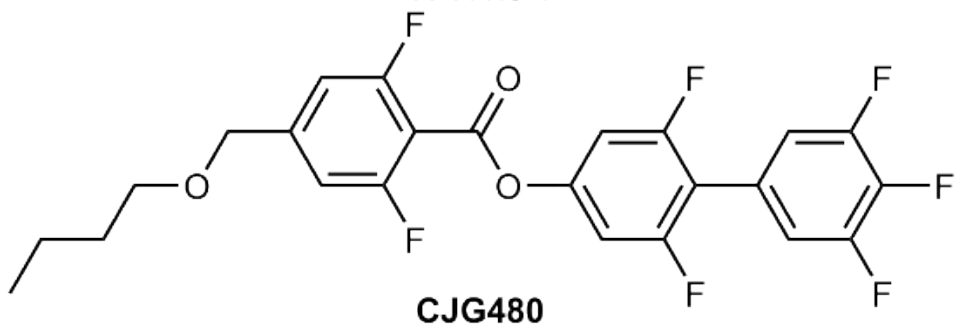

**CJG480**
K 90 I

***Cycle Two***

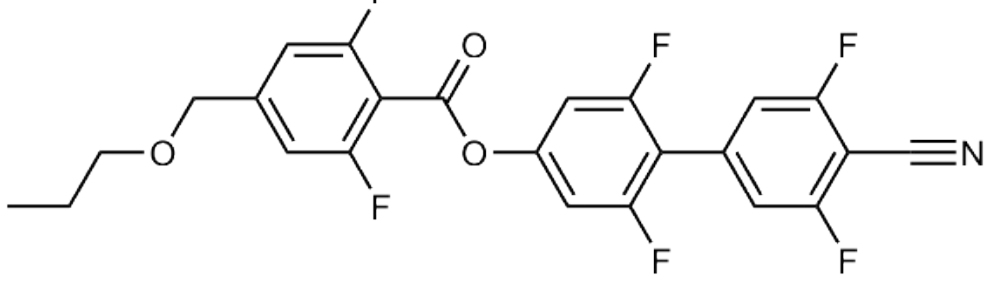

**SRB-CHEM-163**
K 120 ($N_F$ 110) I

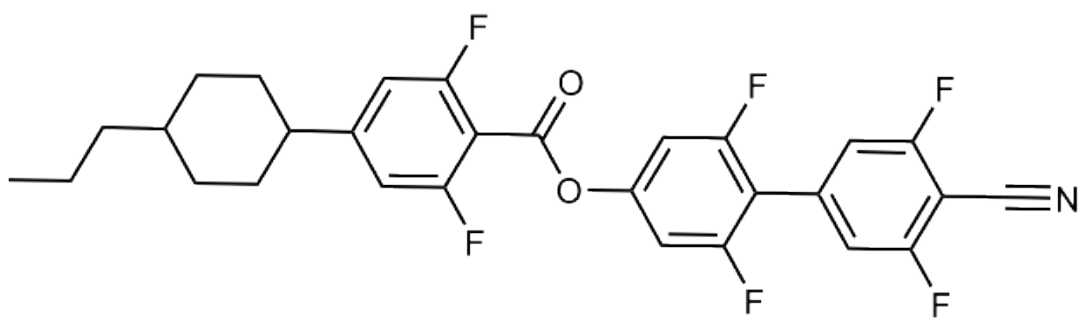

**WCO 4.19**
K 138.1 ($N_F$ 61 $N_X$ 72 N 250.9) I

**Figure SI-90.** Molecular structures of the synthesised candidate materials and their experimental phase sequences as neat materials.

## 13. Current response

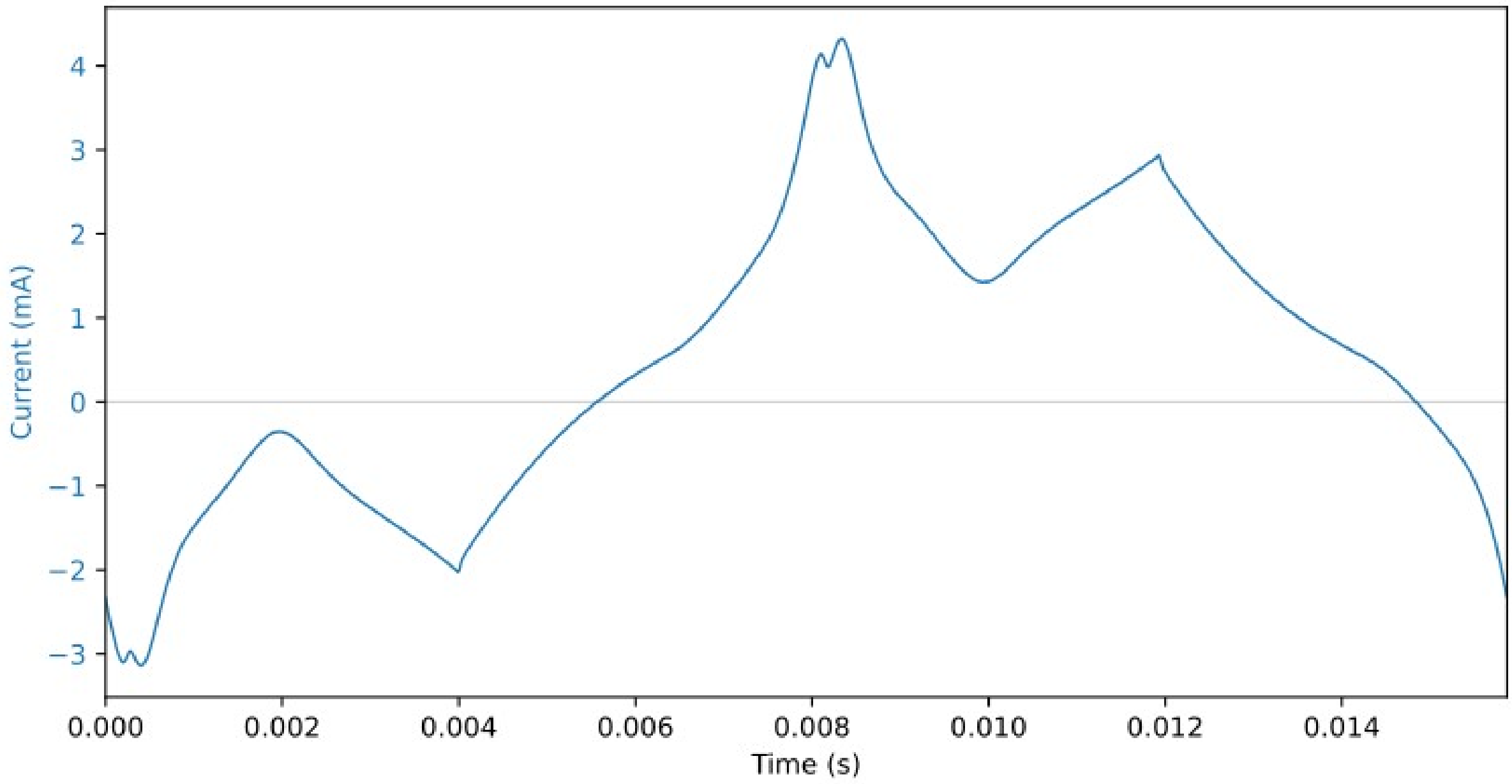


**Figure SI-91.** Current response profile of CJG548 indicating the presence of an $N_X$ phase. The measurement was performed at 417K, 63HZ and 3V.

## References


1. Adamczyk, J. and P. Ludynia, *Scikit-fingerprints: Easy and efficient computation of molecular fingerprints in Python.* SoftwareX, 2024. **28**: p. 101944.
2. Lee, M. and K. Min, *MGCVAE: Multi-Objective Inverse Design via Molecular Graph Conditional Variational Autoencoder.* Journal of Chemical Information and Modeling, 2022. **62**(12): p. 2943-2950.
3. Gibb, C.J., et al., *Spontaneous symmetry breaking in polar fluids.* Nature Communications, 2024. **15**(1): p. 5845.
4. Kinose, Y., et al., *An Unprecedented Impulse-Type Response of Ferroelectric Nematic Liquid Crystals under In-Plane Switching.* Advanced Electronic Materials, 2025. **11**(14): p. 2500153.
5. Kikuchi, H., et al., *Fluid Layered Ferroelectrics with Global C∞v Symmetry.* Advanced Science, 2022. **9**(26): p. 2202048.
